# Chiral Recognition of Carboxylate Anions by (*R*)-BINOL-Based Macrocyclic Receptors

**DOI:** 10.3390/molecules24142635

**Published:** 2019-07-19

**Authors:** Agata Tyszka-Gumkowska, Grzegorz Pikus, Janusz Jurczak

**Affiliations:** Institute of Organic Chemistry Polish Academy of Sciences, Kasprzaka 44/52, 01-224 Warsaw, Poland

**Keywords:** chiral recognition, macrocyclic receptors, BINOL, chiral anions, coalescence

## Abstract

Three (*R*)-BINOL-based macrocyclic receptors obtained via double-amidation reaction were used for chiral recognition of four anions derived from α-hydroxy and α-amino acids. The structural factors of hosts and guests that affect chiral recognition processes were also investigated, indicating that the proper geometry of both receptor and guest molecules plays a crucial role in effective enantio-discrimination.

## 1. Introduction

Molecular recognition has been a subject of intensive study for three decades now, including research into the synthesis and application of neutral receptors able to recognize neutral molecules as well as ions [1,2]. While biological systems are able to effectively distinguish between stereoisomers of guests in water, as is demonstrated for instance by recognition of naproxen enantiomers in the human body [3], the rational design of artificial receptors capable of recognizing chiral anions still remains a great challenge. Chiral recognition is one of the least understood processes in supramolecular chemistry, driven by hard-to-predict effects on the stability of diastereomeric host–guest complexes. Differences in the binding of guest enantiomers are driven by the enthalpy and entropy effects, mainly attributed to the existence of numerous attractive and repulsive noncovalent interactions as well as distinct conformations of guest and host [4,5]. These phenomena can be better understood when host–guest interactions are investigated by means of model chiral recognition of hydroxy and amino acids, which are widely prevalent across various pharmaceuticals, and play crucial roles in numerous biological systems.

Given the importance of chirality and chiral recognition in nature, there is a great need for in-depth research clarifying the correlation between a receptor’s structure and its capability for efficient chiral differentiation. In seeking to elucidate the subtle interactions driving chiral recognition, one of the most common approaches involves the combinatorial evaluation of series of receptors in combination with a wide range of guests [4,6]. Among the systems reported to date, macrocyclic chiral hosts have proved to have favorable enantio-discrimination properties as compared to their acyclic analogues due to increased steric repulsion, which allows for more efficient differentiation of enantiomers [7,8]. It is noteworthy, however, that preparation of macrocyclic compounds, in particular those bearing a chiral moiety, is often tedious, owing to an unfavorable entropy effect during the macrocyclization step [9]. In tackling this issue researchers have often adopted low-cost structural motifs, such as carbohydrates [10] or α-amino acids [11], broadly found in other areas of asymmetric chemistry, making the synthesis of macrocyclic receptors much more affordable in terms of its future practical applications.

Cram and co-workers first reported BINOL-based crown ethers for enantio-selective binding of chiral ammonium salts [12]. Since then, BINOL has been extensively used in chiral recognition [13] and exhibit excellent chiral induction in asymmetric reactions [14]. In recent years many scientists have employed this chiral molecule to create a new group of receptors, which have turned out to be appropriate for effective chiral recognition of anions [15,16,17], cations [18,19] and neutral molecules [20,21].

**Figure 1:**
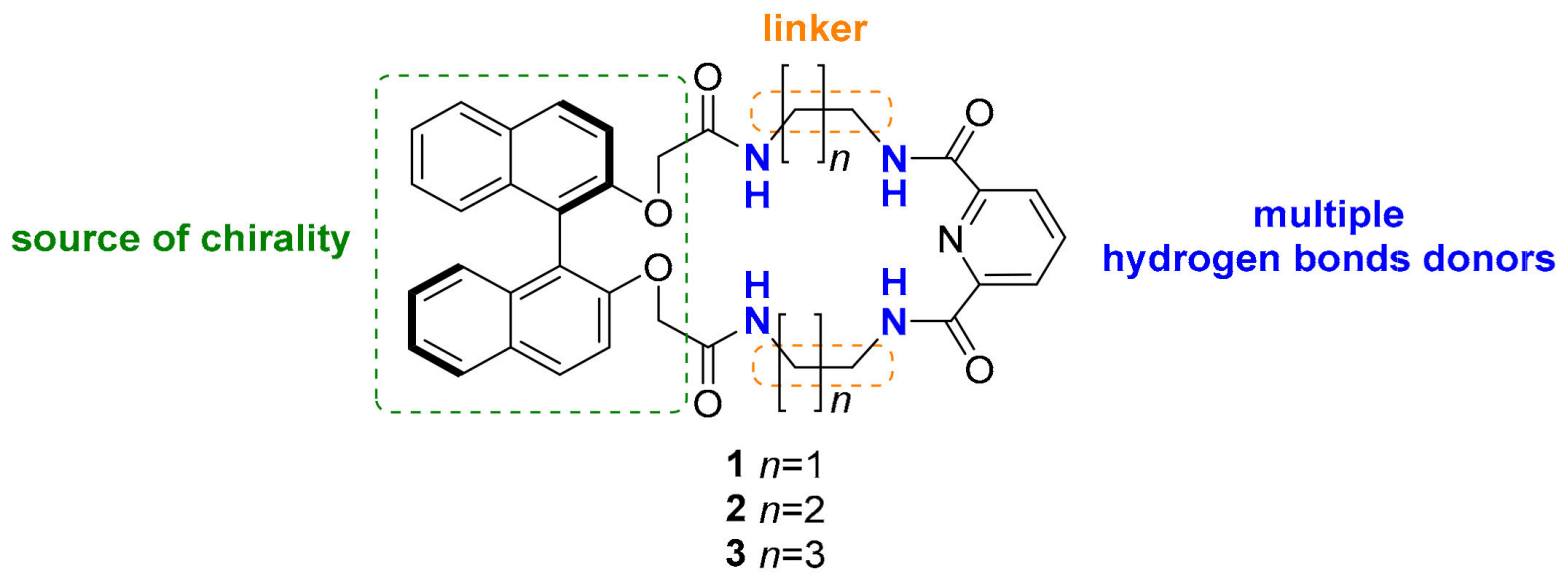
Structure of BINOL–based macrocyclic receptors (*R*)-1–3 investigated herein.

Recently building on our previous experience [18], herein we report on the synthesis of putative macrocyclic receptors (*R*)-**1**–**3**, featuring multiple hydrogen-bonding sites and varied aliphatic linker length (Figure 1). They are thus characterized by varying size and conformation of their macrocyclic pocket, which can translate into chiral recognition abilities towards α-hydroxy and α-amino acids.

## 2. Results and Discussion

**Scheme 1:**
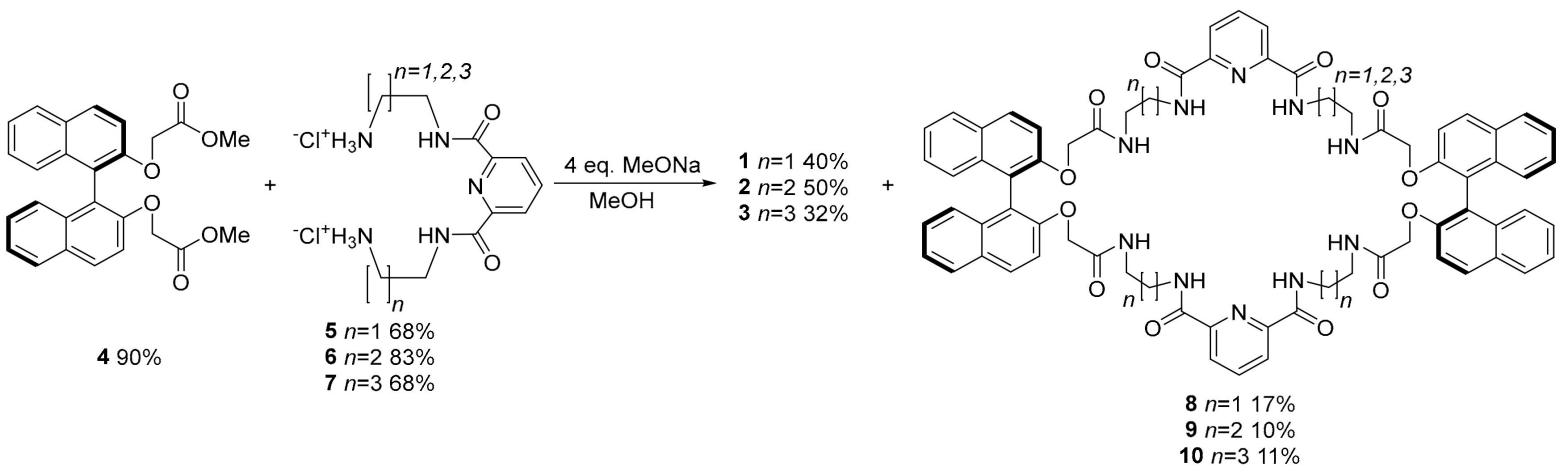
Synthesis of macrocyclic tetramides **1**–**3** and octaamides **8**–**10.**

Receptors (*R*)-**1**–**3** were obtained in a one-step synthetic protocol using **4** and **5**–**7**, readily available via procedures previously reported in our group, as starting materials [22]. The double-amidation reaction of chiral diester **4** with diamines **5**–**7**, catalyzed by sodium methoxide, resulted in the desired macrocyclic tetraamides (*R*)-**1**–**3** in reasonable yields of 40, 50, and 32%, respectively. Furthermore, side-products were identified as macrocyclic octaamides (*R*)-**8**–**10** (Scheme 1). We also synthesized enantiomeric hosts (*S*)-**1**–**3** and (*S*)-**8**–**10** in similar yields (see ESI).

We investigated the binding affinities of the chiral receptors (*R*)-**1**–**3** so obtained, via ^1^H-NMR-controlled titration with respect to model achiral anions of various geometries, such as chloride, dihydrogen phosphate, acetate and benzoate, carried out in the demanding solvent mixture DMSO–*d*_6_ + 0.5% H_2_O (Table 1). For all anions studied, taking into account analysis of the data points fitting to calculated curves and residual errors, we observed the formation of 1:1 complexes (for details see ESI). The values of binding constants were in agreement with the Hofmeister series [23]. Under these conditions, the observed binding constants were low. Furthermore, we carried out a set of additional titrations in a less competitive solvent mixture, namely acetone–*d*_6_ + 0.5% H_2_O, using benzoate as a guest ion. As expected, the values of stability constants increased as compared to former conditions therefore, we decided to perform all the following titrations of chiral carboxylates in this latter solvent mixture.

**Figure 2:**
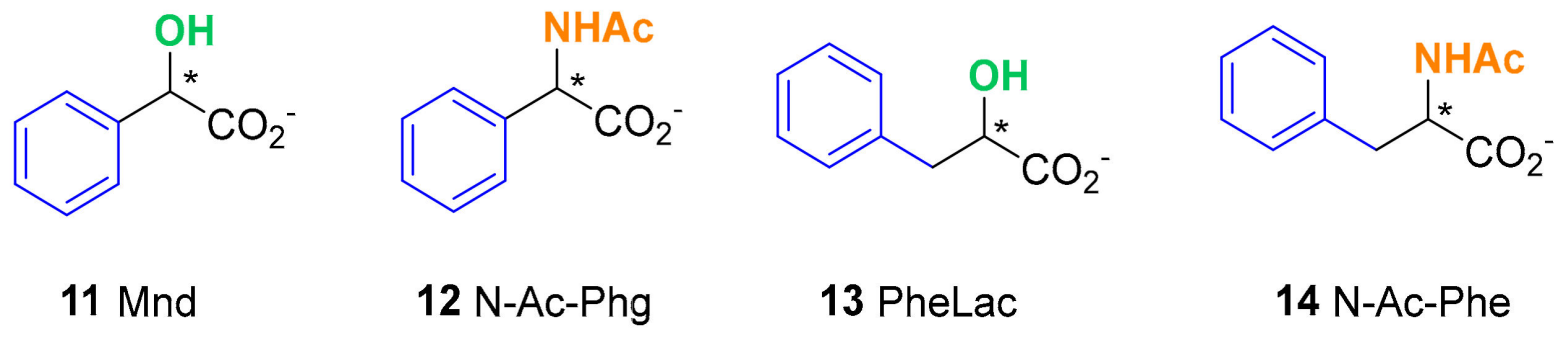
Structure of the anionic guests investigated in this study, used as TBA salts.

With this setup in hand, we were interested in gaining insight into the role played by the anion structure in the chiral recognition process, exploring the ability of macrocyclic receptors (*R*)-**1**–**3** to discriminate selected chiral anions. Inspired by natural compounds and synthetic drugs, we investigated anions possessing a stereogenic center in α-position. We used guest as TBA salts derived from chiral (*R*)/(*S*)-α-hydroxy acids: mandelic acid (**11**) and 3-phenyllactic acid (**13**), as well as from *N*-Ac-d/l-α-amino acids: phenylglycine (**12**) and phenylalanine (**14**) as shown in Figure 2.

The binding constants obtained for hosts (*R*)-**1**–**3** with chiral guests **11**–**14** were lower than those observed for structurally less sophisticated achiral benzoate. By analogy to achiral carboxylates, we observed that hosts (*R*)-**1**–**3** formed 1:1 complexes with chiral guests, and in all cases, the values of K_a_ lay in the convenient range for the ^1^H-NMR technique (91–329 M^−1^) (Table 2).

**Figure 3:**
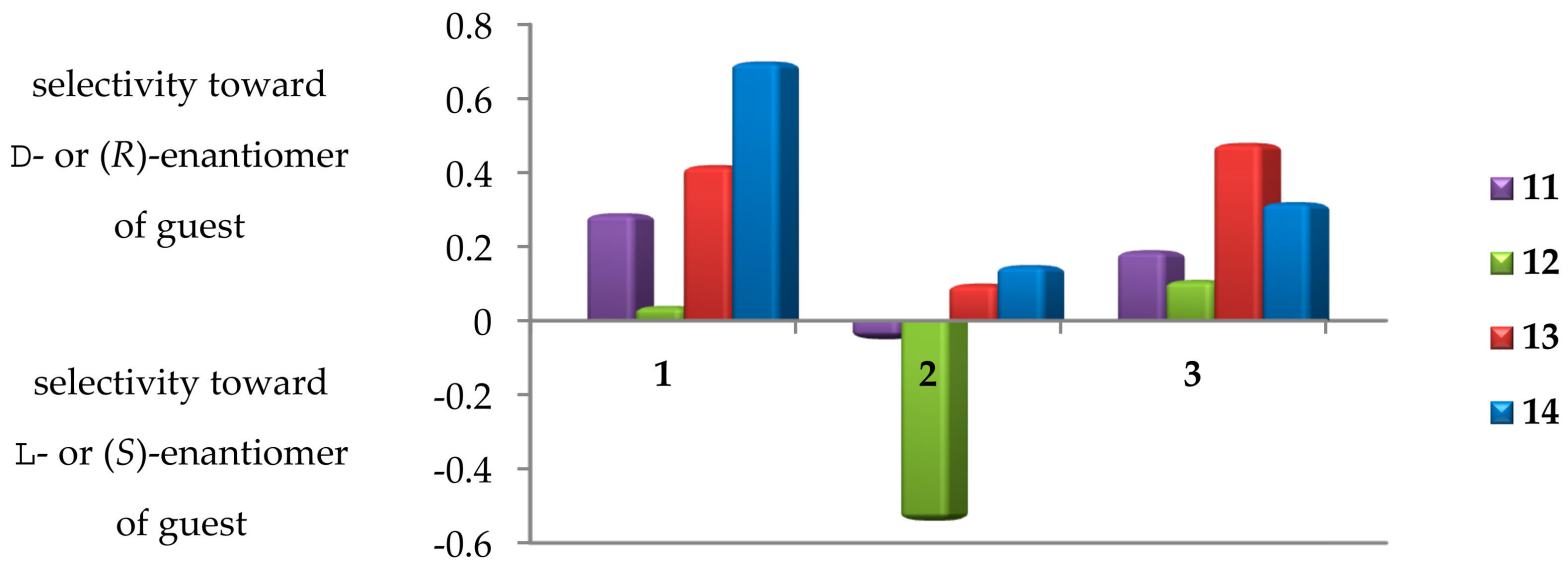
Plot of α_rel_ for receptors (*R*)-**1**–**3** with chiral carboxylates **11**–**14**.

To clarify the discussion below, Figure 3 shows a plot of the relative chiral recognition values (α_rel_ = α − 1 or α_rel_ = (1/α) − 1 in the case of host (*R*)-**2** with guests **11** and **12** when reverse enantioselectivity was observed). According to the estimated errors for stability constants (<10%), chiral recognition in the range of 0.9–1.1 obtained from direct, noncompatitive titrations did not allow us to elucidate the influence of the anion structure on enantio-discrimination.

Figure 3 indicates that in most cases, the explored receptors (*R*)-**1**–**3** display a preference for chiral guest with the (*R*) absolute configuration on stereogenic center, except for two cases involving host (*R*)-**2**. The combined outcomes of the experimental data clearly show pronounced enantio-discrimination properties on the part of macrocyclic host (*R*)-**1** towards three of the four examined anions (**11**, **13** and **14**), and a lack of chiral recognition toward **12** (α = 1.04). Interestingly, in the case of guest **12** and receptor (*R*)-**2**, which is bridged with a longer three-carbon linker, we observed reversed selectivity, manifested by a higher binding constant toward the l-isomer of **12**. Receptor (*R*)-**2** thereby exhibits a high level of chiral recognition for **12** (α = 0.65) in connection with its appropriately organized macro ring, providing space for more adequate binding of the anion than in the case of receptor (*R*)-**1**. On the other hand, the most flexible host (*R*)-**3**, equipped with a four-carbon linker, does show low chiral recognition of anion **12** (α = 1.11), due to the unmatched and large macro ring. Subsequently, we examined guest **11**, characterized by a geometry similar to that of anion **12**, but containing a free hydroxy group in its structure. We found slight changes in the stability constants determined for the complexes formed with enantiomers of **11** with hosts (*R*)-**1**–**3**, resulting in fairly low chiral recognition values (Table 2). These results can be rationalized in the terms of the additional solvation effect of anions **11**, owing to the presence of the free hydroxy group, interacting with both solvent and host molecule, not only as a hydrogen bond acceptor but also as a donor. Interaction with the solvent is responsible for the increased solvation of anion **11**, resulting in weaker binding and low enantio-discrimination of this guest by macrocyclic receptors (*R*)-**1**–**3**. Next, we performed titration experiments with another α-hydroxy acid anion **13**, more flexible than **11** owing to the presence of the additional methylene group. The replacement of the phenyl group by a benzyl substituent resulted in improved chiral recognition for all tested receptors (*R*)-**1**–**3** (Table 2). Afterwards, this small change in the guest structure had a significant impact on differences in binding geometry and strength of enantiomers through receptors (*R*)-**1** and (*R*)-**3**, and in consequence better chiral recognition of anion **13** than **11**. In the light of these results, we decided to incorporate anion **14** into our research. We noted an increase in chiral recognition for hosts (*R*)-**1** and (*R*)-**2**, and a slight decrease for host (*R*)-**3** (Table 2). This was due to the lack of the free hydroxy group in the structure of **14**, characterized by weaker solvation, which is responsible for its relative smaller size as compared with **13**. Therefore, receptors (*R*)-**2** and (*R*)-**3,** having spacious macrocyclic pockets (25- and 27-membered, respectively) can easily adopt their conformation, which leads to similar binding of both enantiomers. Only in the case of the 23-membered host (*R*)-**1** better chiral recognition toward **14** was observed (α = 1.70).

In contrast to the nonmacrocyclic receptors of anions previously reported by our group [24,25], the above-mentioned results led to the conclusion that the presence of the α-hydroxy group in the guest structure is not critical for fine-tuning the chiral recognition ability of macrocyclic hosts presented in this report. Therefore, hosts **1**–**3** demonstrate better enantio-discrimination for *N*-Ac-α-amino acid anions than for α-hydroxy acid ones.

**Figure 4:**
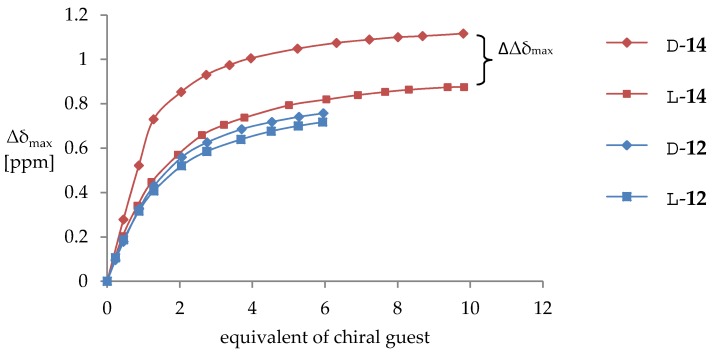
Comparison of the chemical shifts changes for the amide NH protons of receptor **1** (δ 9.05 ppm) upon addition both enantiomers of *N*-Ac-α-amino acids **12** and **14** (acetone–*d*_6_ + 0.5% H_2_O, T = 298 K, 400 MHz); points show experimental data, the line is the fitted chemical shift data.

The above results can also be visualized using chemical shift changes (Δδ_max_) of protons originating from the receptor amide groups, being donors of hydrogen bonds formed, as shown in Figure 4.

Figure 4. illustrates the representative, well-visible correlation between the changes of Δδ_max_ value for receptor (*R*)-**1** during titrations with pairs of enantiomeric anions **12** and **14**. When a significant chiral recognition was noted for guest **14** (labelled in red), a major ΔΔδ_max_ value was observed, whereas in the case of low chiral recognition for **12** (labelled in blue) only slight differences between Δδ_max_ for enantiomers was present. The details of such correlations for other hosts and guests are given in ESI.

**Figure 5:**
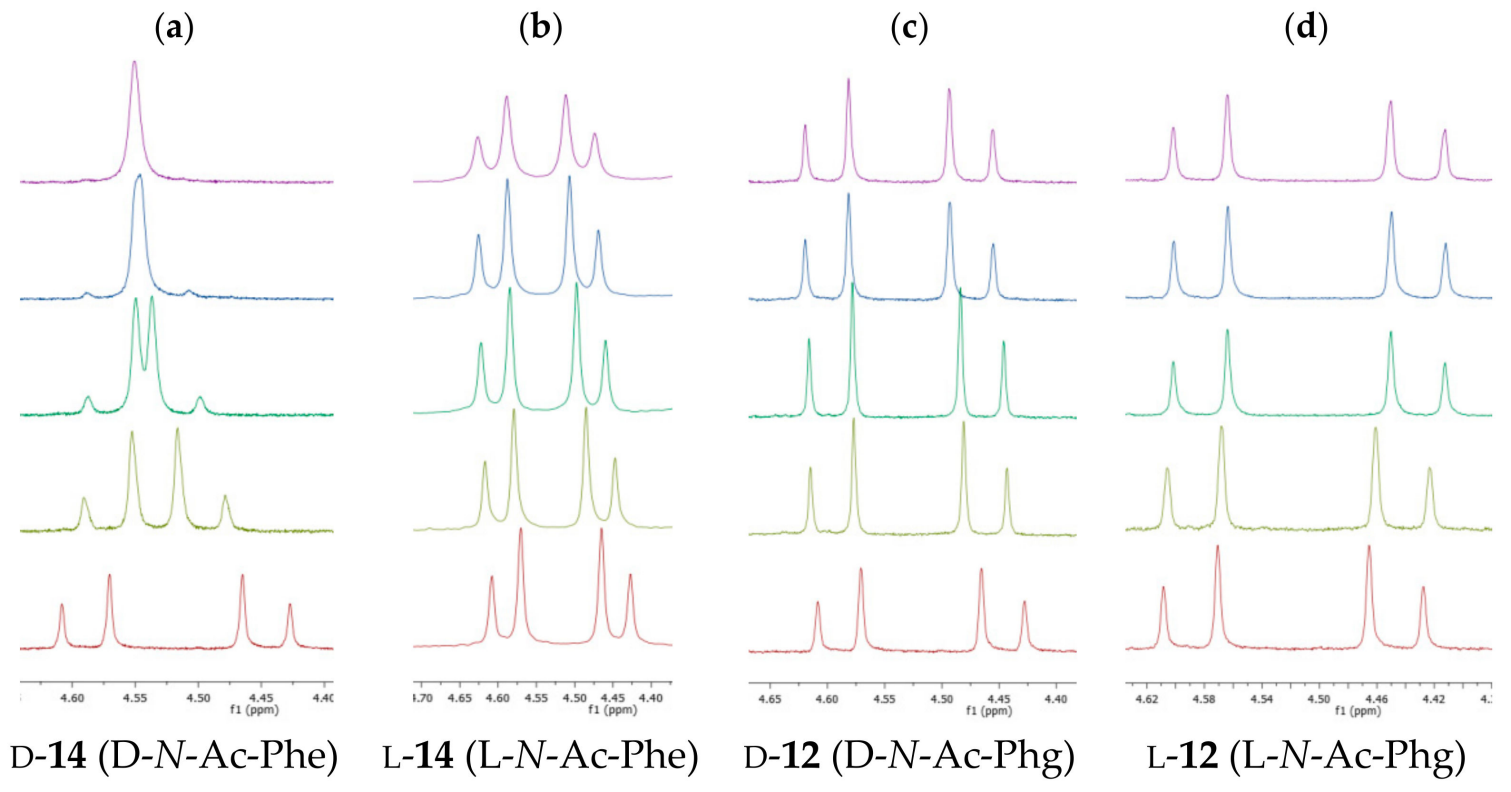
Fragments of stacked spectra from ^1^H-NMR titration of both enantiomers of guest **11** and **12** with host (*R*)-**1** (acetone–*d*_6_ + 0.5% H_2_O, T = 298 K, 400 MHz).

Strong evidence of stereoselective interactions of examined host (*R*)-**1** with enantiomeric guests **12** and **14** was also visible in the multiplicity of signals originating from the diastereotopic methylene protons, shown in Figure 5.

During the titration of macrocyclic host (*R*)-**1** with d-enantiomer of **14**, the coalescence of diastereotopic methylene group signals was noted, indicating a change in conformation of the macro ring (Figure 5a). When the host was titrated with l-enantiomer of **14**, the multiplicity of these signals stood intact and we noticed only slight changes in chemical shifts (Figure 5b). These observations suggest that binding of d-enantiomer is assisted by a favorable macro ring twist conformation, as reflected in the discrepancies in multiplicity of the appropriate protons and in the great enantio-discrimination properties of host (*R*)-**1**. On the other hand, when a low level of chiral recognition was found, like in the case of guest **12**, no similar effects were noted (Figure 5c,d). We can also observe dependencies of this type for other chiral guests (**13** and **14**) interacting with host (*R*)-**1** (for details see ESI). Interestingly, no identical difference in signal multiplicity was seen for receptors (*R*)-**2** and (*R*)-**3**, owing to their larger macro ring pockets.

## 3. Conclusions

In conclusion, we have presented a convenient and efficient synthesis of three BINOL-based macrocyclic receptors (*R*)-**1**–**3**, and demonstrated their chiral recognition ability toward important α-hydroxy and α-amino acid anions. The structural factors affecting chiral recognition were studied, and it was established that α-amino acid anion **14** was recognized better than **12**, and α-hydroxy acid anion **13** similarly prevailed over **11**. We also found that the optimum-sized host (*R*)-**1** can pre-organize its chiral pocket, interacting much more effectively with only one enantiomer of guest molecules. This was shown by the best chiral recognition ability of host (*R*)-**1**, and by the low enantioselectivity of host (*R*)-**2** and (*R*)-**3**, due to their overly large and flexible macrocyclic cavity. Investigation of methylene group signal changes also reveals that host (*R*)-**1** can serve as a chirality sensor for carboxylates. This transparently indicates that the proper geometry and the adoption of favored conformation for both receptor and guest molecules plays a crucial role in effective enantio-discrimination.

We anticipate that the findings reported herein will prove useful in the better design, synthesis, and use of new artificial sensors, responsive to chiral species.

## 4. Experimental Section

### 4.1. General Procedure of the Macrocyclization Reaction

One equivalent of an appropriate hydrochloride (**5**, **6** or **7**), one equivalent of (*R*)- or (*S*)-diester **1** and four equivalents of sodium methoxide were dissolved in dry methanol (concentration 0.02 M). The mixture was stirred at room temperature for 3 days (monitoring by TLC). After completion of the reaction, the solvent was evaporated and residue was purified by column chromatography (silica gel, MeOH in CH_2_Cl_2_ from 1 to 10%), obtaining white solids as products: macrocyclic tertaamides (**1**–**3**) and octaamides (**8**–**10**).

### 4.2. Characterization Data for Products 1–3 and 8–10

*Tetraamide***1**: White solid. Yield 40% (*R*), $\alpha_{D}^{rt}$ = +92.2 (c = 0.1, CH_2_Cl_2_); yield 48% (*S*), $\alpha_{D}^{rt}$ = −92.2 (c = 0.1, CH_2_Cl_2_); m.p. 201–202 °C; ^1^H-NMR (400 MHz, CDCl_3_) δ 9.05 (bt, *J* = 4.6 Hz, 2H), 8.30 (d, *J* = 7.8 Hz, 2H), 8.00 (t, *J* = 7.8 Hz, 1H), 7.91–7.77 (m, 4H), 7.40 (t, *J* = 7.2 Hz, 2H), 7.34–7.17 (m, 4H), 7.08 (d, *J* = 8.5 Hz, 2H), 6.87 (t, *J* = 5.8 Hz, 2H), 4.44 (ABq, *J* = 15.3 Hz, 4H), 3.75–3.58 (m, 2H), 3.51–3.36 (m, 2H), 3.33–3.08 (m, 4H); ^13^C-NMR (101 MHz, CDCl_3_) δ 170.6, 163.8, 154.0, 148.4, 138.8, 133.6, 130.5, 130.4, 128.1, 127.2, 125.1, 125.0, 124.3, 121.5, 117.8, 71.4, 40.8, 38.7; HRMS (*m*/*z*): cacld. for C_35_H_31_N_5_O_6_Na [MNa+]: 640.2172, found 640.2170.

**Figure 6:**
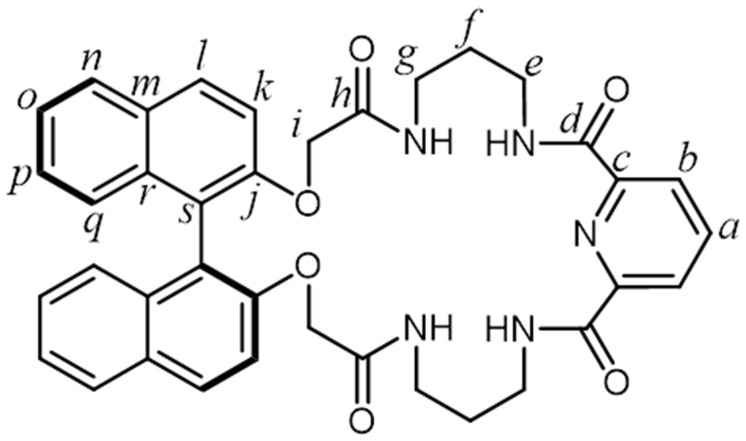
The structure of the receptor **2** with the designations of hydrogen and carbon atoms.

*Tetraamide***2**: White solid. Yield 50% (*R*), $\alpha_{D}^{rt}$ = +50.5 (c = 0.1, CH_2_Cl_2_); yield 60% (*S*), $\alpha_{D}^{rt}$ = −50.5 (c = 0.1, CH_2_Cl_2_), m.p. 244.5–245 °C; ^1^H-NMR (600 MHz, DMSO-d_6_) δ 9.28 (t, *J* = 6.1 Hz, 2H), 8.21–8.14 (m, 3H*^a^*^,*b*^), 8.08 (d, *J* = 9.1 Hz, 2H*^l^*), 7.96 (d, *J* = 8.1 Hz, 2H^n^), 7.89 (t, *J* = 5.9 Hz, 2H), 7.56 (d, *J* = 9.1 Hz, 2H*^k^*), 7.41–7.32 (m, 2H*^o^*), 7.29–7.20 (m, 2H*^p^*), 6.93 (d, *J* = 8.5 Hz, 2H*^q^*), 4.49 (ABq, *J* = 14.8 Hz, 4H*^i^*), 3.35–3.22 (m, 4H*^e^*), 3.21–3.12 (m, 4H*^g^*), 1.62 (m, 4H*^f^*); ^13^C-NMR (150 MHz, DMSO-d_6_) δ 168.7*^h^*, 162.7*^d^*, 153.6*^j^*, 148.5*^c^*, 139.6*^a^*, 133.2*^m^*, 129.7*^l^*, 129.3*^r^*, 128.1*^n^*, 126.6*^p^*, 124.7*^q^*, 124.0*^o^*, 123.9*^b^*, 119.2*^s^*, 116.2*^k^*, 68.9*^i^*, 35.7*^g^*, 35.5*^e^*, 28.6*^f^*; HRMS (*m*/*z*): cacld. for C_37_H_35_N_5_O_6_Na [MNa^+^]: 668.2485, found 668.2488. The structure was interpreted by extra COSY, HSQC and HMBC spectra (details in ESI). Designations of hydrogen and carbon atoms are labeled in Figure 6.

*Tetraamide***3**: White solid. Yield 32% (*R*), $\alpha_{D}^{rt}$ = +73.5 (c = 0.1, CH_2_Cl_2_); yield 35% (*S*), $\alpha_{D}^{rt}$ = −73.5 (c = 0.1, CH_2_Cl_2_), m.p. 143–144 °C; ^1^H-NMR (400 MHz, CDCl_3_) δ 9.01 (bs, 2H), 8.34 (d, *J* = 7.7 Hz, 2H), 8.09 (d, *J* = 8.9 Hz, 2H), 7.97 (t, *J* = 7.2 Hz, 3H), 7.55–7.29 (m, 6H), 7.21 (d, *J* = 8.4 Hz, 2H), 5.91 (bs, 2H), 4.55 (s, 4H), 3.83–3.63 (m, 2H), 3.40–3.23 (m, 2H), 3.22–2.91 (m, 4H), 1.59–1.27 (m, 9H); ^13^C-NMR (101 MHz, CDCl_3_) δ 168.5, 163.8, 152.4, 149.0, 138.4, 133.5, 130.6, 129.8, 128.2, 127.5, 125.2, 124.9, 124.6, 119.7, 114.3, 68.7, 39.6, 38.1, 27.9, 24.9; HRMS (*m*/*z*): cacld. for C_39_H_39_N_5_O_6_Na [MNa^+^]: 696.2798, found 696.2799.

*Octaamide***8**: White solid. Yield 17% (*R*), $\alpha_{D}^{rt}$ = +95.1 (c = 0.1, CH_2_Cl_2_); yield 5% (*S*), $\alpha_{D}^{rt}$ = −95.1 (c = 0.1, CH_2_Cl_2_); m.p. 186–188 °C; ^1^H-NMR (400 MHz, CDCl_3_) δ 9.08 (bs, 4H), 8.32 (d, *J* = 7.7 Hz, 4H), 8.03 (t, *J* = 7.7 Hz, 2H), 7.93–7.77 (m, 8H), 7.43 (t, *J* = 7.3 Hz, 4H), 7.36–7.24 (m, 8H), 7.09 (d, *J* = 8.4 Hz, 4H), 6.87 (bs, 4H), 4.46 (ABq, *J* = 38.2, 15.3 Hz, 8H), 3.74–3.59 (m, 4H), 3.53–3.40 (m, 4H), 3.35–3.12 (m, 8H); ^13^C-NMR (101 MHz, CDCl_3_) δ 170.6, 163.9, 154.0, 148.3, 138.9, 133.6, 130.5, 130.4, 128.1, 127.2, 125.2, 125.0, 124.4, 121.6, 117.9, 71.4, 40.8, 38.7; HRMS (*m*/*z*): cacld. for C_70_H_62_N_10_O_12_Na [MNa^+^]: 1257.4446, found 1257.4442.

*Octaamide***9**: White solid. Yield 10% (*R*), $\alpha_{D}^{rt}$ = +96.3 (c = 0.1, CH_2_Cl_2_); yield 12% (*S*), $\alpha_{D}^{rt}$ = −96.3 (c = 0.1, CH_2_Cl_2_); m.p. 175–177 °C; ^1^H-NMR (400 MHz, CDCl_3_) δ 9.02 (t, *J* = 6.4 Hz, 4H), 8.25 (d, *J* = 7.8 Hz, 4H), 8.07–7.88 (m, 10H), 7.48–7.21 (m, 16H), 5.68 (t, *J* = 6.2 Hz, 4H), 4.40 (qAB, *J* = 14.6 Hz, 8H), 3.34–3.19 (m, 4H), 3.15–2.95 (m, 8H), 2.86–2.71 (m, 4H), 1.42–1.27 (m, 4H), 1.25–1.08 (m, 4H); ^13^C-NMR (101 MHz, CDCl_3_) δ 168.5, 163.6, 152.5, 148.7, 138.8, 133.4, 130.5, 129.9, 128.4, 127.5, 125.0, 124.9, 124.4, 119.8, 114.9, 68.5, 35.9, 35.5, 29.0; HRMS (*m*/*z*): cacld. for C_74_H_70_N_10_O_12_Na [MNa^+^]: 1313.5072, found 1313.5098.

*Octaamide***10**: White solid. Yield 11% (*R*), $\alpha_{D}^{rt}$ = +96.6 (c = 0.1, CH_2_Cl_2_); 10% (*S*), $\alpha_{D}^{rt}$ = −96.6 (c = 0.1, CH_2_Cl_2_); m.p. 159–160 °C; ^1^H-NMR (400 MHz, CDCl_3_) δ 8.92 (bs, 4H), 8.45 (d, *J* = 7.7 Hz, 4H), 8.11 (t, *J* = 7.7 Hz, 2H), 8.02 (d, *J* = 8.9 Hz, 4H), 7.91 (d, *J* = 8.1 Hz, 4H), 7.42 (t, *J* = 7.3 Hz, 4H), 7.35–7.22 (m, 9H), 7.14 (d, *J* = 8.4 Hz, 4H), 5.85 (bs, 4H), 4.51 (d, *J* = 14.9 Hz, 4H), 4.31 (d, *J* = 14.9 Hz, 4H), 3.52–3.35 (m, 4H), 3.31–3.12 (m, 4H), 3.07–2.73 (m, 9H), 1.35–1.08 (m, 16H); ^13^C-NMR (101 MHz, CDCl_3_) δ 168.1, 163. 9, 152.0, 149.0, 138.8, 133.4, 1306, 129.7, 128.2, 127.5, 125.0, 124.8, 119.4, 114.0, 68.1, 39.2, 38.3, 26.7, 26.4; HRMS (*m*/*z* cacld. for C_78_H_78_N_10_O_12_Na [MNa^+^]: 1369.5698, found 1369.5720.

### 4.3. Titration Experiments

Tetrabutylammonium (TBA) salts of examinate anions were prepared before every titration experiments, namely commercially available carboxylic acid (from Sigma Aldrich or TCI Europe) was dissolved in 0.5 mL of dry methanol and one equivalent of TBAOH (solution in methanol, c = 1.21 M) was added. Prior to the experiment, the salts were pre-dried overnight under high vacuum at 60 °C. To obtain the appropriate water concentration distilled water was added to the commercially available DMSO-*d*_6_ or acetone-*d*_6_ of 99.9% isotopic purity. All titration experiments was performed on Bruker (400 MHz) at 298K.

### 4.4. ^1^H NMR Titration Procedure

The solution of a receptor (~10^−3^ M) was titrated in NMR tube with the 0.1–0.3 M solution of a respective TBA salt. The solution of the salt contained a certain amount of the receptor to keep receptor concentration constant during titration experiments. It was important to choose such volumes of aliquots so that most of the data points could occur in close proximity of the inflection point of the respective titration curve; 11 to 23 data points were recorded. Such procedure allows for more precise calculation of binding constants. A nonlinear curve fitting for the 1:1 binding model was carried out with the HypNMR2008 Software [26,27,28] (Version 4.0.71) and allows the determination of the global association constant. The details are given in ESI Figures S27–S65 and Tables S1–S38.

## Figures and Tables

**Table 1:** Binding constants for the formation of 1:1 complexes of receptors (*R*)-**1**–**3** with various anions ^[a]^ determined by ^1^H–NMR titration experiments in DMSO–*d*_6_ + 0.5% H_2_O mixture and acetone–*d*_6_ + 0.5% H_2_O at 298 K, 400 MHz ^[b]^.

Host	Macro Ring Size	Binding Constants [M^−1^]				
		DMSO–*d*_6_ + 0.5% H_2_O				Acetone–*d*_6_ + 0.5% H_2_O
		Cl^−^	H_2_PO_4_^−^	AcO^−^	BzO^−^	BzO^−^
**1**	23	12	121	90	25	916
**2**	25	35	136	82	29	916
**3**	27	8	148	60	25	972

^[a]^ Tetrabutylammonium (TBA) salts were used as a source of anions. ^[b]^ Values determined with error of estimate <10% using HypNMR2008 Software.

**Table 2:** Binding constants for the formation of 1:1 complexes of receptors (*R*)-**1**–**3** with chiral anions determined by ^1^H-NMR titration experiments in acetone *d*_6_ + 0.5% H_2_O mixture at 298 K, 400 MHz ^(a)^.

Host	Chiral Guests ^(b)^							
	11 Mnd		12 *N*-Ac-Phg		13 Phelac		14 *N*-Ac-Phe	
	K_a_	α ^(c)^	K_a_	α	K_a_	α	K_a_	α
**1**	K_R_ = 215	1.29	K_D_ = 229	1.04	K_R_ = 233	1.42	K_D_ = 318	1.70
	K_S_ = 167		K_L_ = 221		K_S_ = 164		K_L_ = 187	
**2**	K_R_ = 96	0.95	K_D_ = 124	0.65	K_R_ = 100	1.10	K_D_ = 140	1.15
	K_S_ = 101		K_L_ = 192		K_S_ = 91		K_L_ = 122	
**3**	K_R_ = 227	1.19	K_D_ = 329	1.11	K_R_ = 169	1.48	K_D_ = 221	1.32
	K_S_ = 191		K_L_ = 297		K_S_ = 114		K_L_ = 167	

^(a)^ Values determined with errors of estimation <10% using HypNMR2008 Software. ^(b)^ Used as tetrabutylammonium (TBA) salts. ^(c)^ α = K_R_/K_S_ (for **11** and **13**) or α = K_D_/K_L_ (for **12** and **14**).

## Supplementary Materials

The following are available online: synthetic procedures, ^1^H and ^13^C-NMR spectral data for all compounds, ^1^H NMR titration experiments details.
