# Supplementary material for: Chiral Recognition of Carboxylate Anions by (R)-BINOL-Based Macrocyclic Receptors
Source: Molecules. 2019 Jul 19;24(14):2635. doi: 10.3390/molecules24142635 (PMC6680683; doi:10.3390/molecules24142635)
Supplement: Supplementary file 1 [file molecules-24-02635-s001.pdf]

Supporting Information

Chiral recognition of carboxylate anions by (*R*)-BINOL-based macrocyclic receptors

Agata Tyszka-Gumkowska, Grzegorz Pikus, and Janusz Jurczak *

Institute of Organic Chemistry Polish Academy of Sciences, Kasprzaka 44/52, 01-224 Warsaw, Poland;

jurczak_group@icho.edu.pl

Table of content

1. Synthetic Procedures S2

1.1. General Remarks S2

1.2. Synthetic pathways for obtaining substrates to macrocyclization step S2

1.3. General procedures S2

1.4. Substance analysis S3

3. Copies of the NMR spectra S6

2. Binding Studies S31

2.1. General remarks S31

2.2. ^{1}H NMR titration experiments with achiral anions S32

2.3. ^{1}H NMR titration data from experiments with macrocyclic compound (*R*)-1 .... S60

2.4. ^{1}H NMR titration data from experiments with macrocyclic compound (*R*)-2 .... S75

2.5. ^{1}H NMR titration data from experiments with macrocyclic compound (*R*)-3 .... S92

1. Synthetic Procedures

1.1. General Remarks

All reagents were purchased from Sigma-Aldrich or TCI and used without further purification. The solvents were dried by distillation over the appropriate drying agents. Flash column chromatography was performed on silica gel (230–400 mesh), thin-layer chromatography (TLC) was carried out on aluminium sheets precoated with silica gel. ^{1}H NMR and ^{13}C NMR spectra were recorded on Varian 600 at 600 and 125 MHz, respectively, and on Bruker Mercury 400 instrument at 400 and 100 MHz, respectively. Proton and carbon chemical shifts are reported in ppm (δ) ($CDCl_3$: ^{1}H NMR δ = 7.26 and ^{13}C NMR δ = 77.26, DMSO-d_6: ^{1}H NMR δ = 7.26 and ^{13}C NMR δ = 77.26). J coupling constants values are reported in Hz. Melting points are uncorrected. High resolution mass spectra (HRMS) were recorded using ESI-TOF technique. Specific rotations were measured using a JASCO P-2000 polarimeter. Melting points were measured with a Koefler type (Boetius M) hot-stage apparatus.

1.2. Synthetic pathways for obtaining substrates to macrocyclization step

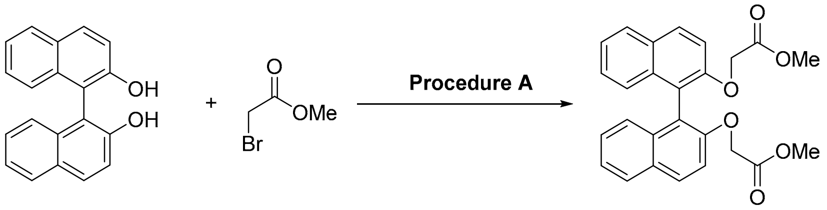

(*R*)-BINOL **(*R*)-4**
or (*S*)-BINOL **(*S*)-4**

Scheme S1. Synthesis of chiral esters based on BINOL moiety

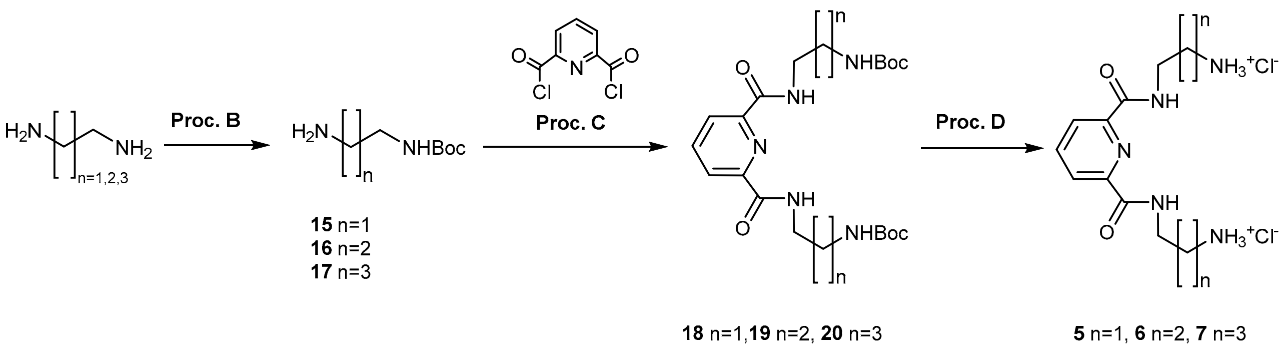

Scheme S2. Synthesis of diamines **5-7**

1.3. General procedures

Procedure A:

(*R*)-BINOL or (*S*)-BINOL (1.0 mmol, 286,3 mg) was dissolved in acetonitrile (20 mL), next anhydrous potassium carbonate (4.0 mmol. 552,8 mg) and methyl bromoacetate (2.5 mmol, 382.4 mg, 237.0 uL) were added. The mixture was stirring at reflux for 18 h. After cooling, the reaction mixture was filtrated and the solvent was removed under reduced pressure to give a colourless oil. Residue was purified by crystallization from methanol to gave product (*R*)-**4** or (*S*)-**4** as white crystals.

Procedure B:

The corresponding α,ω-diamine (30.0 mol) was dissolved in chloroform (500 mL) and a solution of di-*tert*-butyl dicarbonate (0.1 mol, 21.8 g) in chloroform (300 mL) was added dropwise at 0 °C within 3h.

The reaction mixture was stirred at room temperature for another 18h. Ethyl acetate (200 mL) and water (200 mL) were added and the mixture was stirred for 1h. The organic layer was separated, washed with 5% aqueous solution of sodium hydrocarbonate (2 x 200 mL) and dried over anhydrous disodium sulphate. The solvents were evaporated under reduced pressure. Residue was purified by distillation under reduce pressure to recovered starting α,ω-diamine, and to give products **15-17** as a colourless oils.

Procedure C:
The corresponding *N*-mono-Boc-substituted α,α-diamine (**15**-**17**) (22.0 mmol) and triethylamine (22.0 mmol, 2.22 g) were dissolved in dry DCM (150 mL) at 0 °C and a solution of 2,6-dipicolinic chloride (10.0 mmol, 2.04 g)) in dry DCM (100 mL) was added dropwise. After 2h the solvent was evaporated to give a yellow oily residue which was purified by column chromatography (silica gel, ethyl acetate). The final products **18**-**20** was obtained as a white solids.

Procedure D:
4M solution of hydrochloride in dioxane (20 mL) was added To the solution of corresponding derivative (**18**-**20**) (20.0 mmol) in dry DCM (20 mL). The reaction mixture was stirring at room temperature for 3hand the solvent was evaporated to give product as a white solid (**5**-**7**).

1.4. Substance analysis

**Compound (*S*)-4**<br>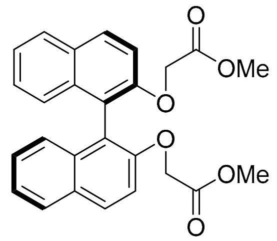<br>**(*R*)-4**<br>Mw= 430.46 g/mol	By starting from (*R*)-BINOL and following the procedure , 0.387 g (90%) of diester (*R*)-4 was isolated as a white crystals (mp 134 °C; $[\alpha]^{25}_{D}$ +26.5 (c=0.1, CH_2Cl_2).<br>**^{1}H NMR** (400 MHz, $CDCl_3$) δ 7.94 (d, *J*=8.9 Hz, 2H), 7.87-7.85 (m, 2H), 7.36-7.32 (m, 4H), 7.26-7.21 (m, 2H), 7.18-7.17 (m, 1H), 7.17-7.16 (m, 1H), 4.55 (s, 4H), 3.62 (s, 6H).<br>**^{13}C NMR** (100 MHz, $CDCl_3$) δ 169.8, 153.8, 134.0, 129.9, 129.7, 127.9, 126.5, 125.7, 124.2, 120.6, 115.8, 67.3, 51.9.
**Compound (*R*)-4**<br>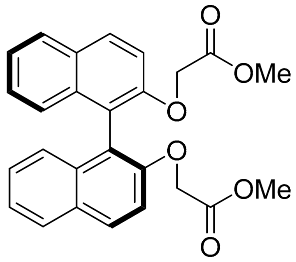<br>**(*S*)-4**<br>Mw= 430.46 g/mol	By starting from (*S*)-BINOL and following the procedure , 0.388 g (91%) of diester (*S*)-4 was isolated as a white crystals (mp 134 °C; $[\alpha]^{25}_{D}$ -26.5 (c=0.1, CH_2Cl_2).<br>**^{1}H NMR** (400 MHz, $CDCl_3$) δ 7.94 (d, *J*=8.9 Hz, 2H), 7.87-7.85 (m, 2H), 7.36-7.32 (m, 4H), 7.26-7.21 (m, 2H), 7.18-7.17 (m, 1H), 7.17-7.16 (m, 1H), 4.55 (s, 4H), 3.62 (s, 6H).<br>**^{13}C NMR** (100 MHz, $CDCl_3$) δ 169.8, 153.8, 134.0, 129.9, 129.7, 127.9, 126.5, 125.7, 124.2, 120.6, 115.8, 67.3, 51.9.

**Compound 5**<br>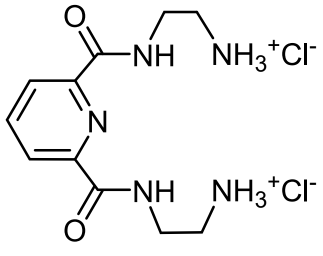<br>Mw=324,21 g/mol	By starting from **18** and following the procedure D, 6.160 g (95 %) of **5** was synthesized as white solid m.p. 205 ^{o}C.<br>**^{1}H NMR** (400 MHz, DMSO-d$_6$) δ 10.03 (t, J=5.97 Hz, 2H), 8.26 (bs, 6H), 8.22 - 8.18 (m, 3H), 3.65 (q, J=6.04 Hz, 4H), 3.09 (t, J=6.04, 4H).<br>**^{13}C NMR** (100 MHz, CDCl$_3$) δ 163.6, 148. 5, 139.4, 124.4, 38.7, 36.6.
**Compound 6**<br>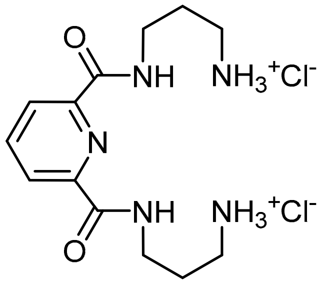<br>Mw=352,26 g/mol	By starting from **19** and following the procedure D, 6.622 g (94 %) of **6** was synthesized as white solid m.p. 199-200 ^{o}C.<br>**^{1}H NMR** (400 MHz, DMSO-d$_6$) δ 9.95 (t, J=6,2 Hz, 2H), 8.20 - 8.14 (m, 3H), 3.95 (bs, 6H), 3.44 (q, J=6.3 Hz, 4H), 2.86 (dt, J=6.2, J=6.3 Hz, 4H), 1.90 (p, J=6.8 Hz, 4H).<br>**^{13}C NMR** (100 MHz, CDCl$_3$) δ 163.3, 148.6, 139.4, 124.1, 36.5, 35.5, 27.0.
**Compound 7**<br>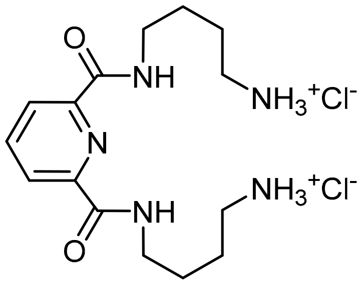<br>Mw=380,31 g/mol	By starting from **20** and following the procedure D, 7.226 g (95 %) of **7** was synthesized as white solid m.p. 174-175 ^{o}C.<br>**^{1}H NMR** (400 MHz, DMSO-d$_6$) δ 9.71 (t, J=6.1 Hz, 2H), 8.17 - 8.08 (m, 9H), 3.36 - 3.33 (m, 4H), 2.80 - 2.78 (m, 4H), 1.64 - 1.60 (m, 8H).<br>**^{13}C NMR** (100 MHz, CDCl$_3$) δ 163.1, 148.8, 139.2, 124.0, 38.4, 38.0, 26.2, 24.4.
**Compound 15**<br>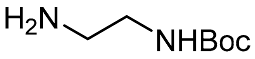<br>Mw=160,22 g/mol	By starting from ethane-1,2-diamine and following the procedure B, 10.890 g (68 %) of carbamate **15** was synthesized in the form of colorless oil. b.p. 88-89 ^{o}C (0.3 mmHg).<br>**^{1}H NMR** (400 MHz, CDCl$_3$) δ 4.94 (bs, 1H), 3.16 (q, 2H), 2.79 (t, 2H), 1.45 (s, 9H), 1.12 (bs, 2H).<br>**^{13}C NMR** (100 MHz, CDCl$_3$) δ 156.2, 79.1, 43.4, 41.9, 28.4.
**Compound 16**<br>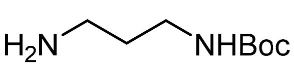<br>Mw=174,24 g/mol	By starting from propane-1,3-diamine and following the procedure B, 14.460 g (83 %) of carbamate **16** was synthesized in the form of colorless oil.b.p. 90-91 ^{o}C (0.3 mmHg).<br>**^{1}H NMR** (400 MHz, CDCl$_3$) δ 4.96 (bs, 1H), 3.21 (q, *J*=6.6 Hz, 2H), 2.76 (t, *J*=6,6 Hz, 2H), 1.61 (p, *J*=6.6 Hz, 2H), 1.44 (s, 9H), 1.17 (bs, 2H).<br>**^{13}C NMR** (100 MHz, CDCl$_3$) δ 156.1, 79.0, 39.7, 38.5, 33.5, 28.4.
**Compound 17**<br>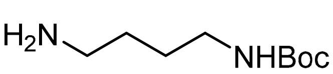<br>Mw=188,27 g/mol	By starting from buthane-1,4-diamine and following the procedure B, 12.802 g (68 %) of carbamate **17** was synthesized in the form of colorless oil.b.p. 95-96 ^{o}C (0.3 mmHg).<br>**^{1}H NMR** (400 MHz, CDCl$_3$) δ 4.73 (bs, 1H), 3.16-3.10 (m, 2H), 2.73- 2.69 (m, 2H), 1.54- 1.46 (m, 4H), 1.44 (s, 9H), 1.11 (bs, 2H).<br>**^{13}C NMR** (100 MHz, CDCl$_3$) δ 156.0, 79.0, 41.8, 40.4, 30.9, 28.4, 27.5.

Compound 18

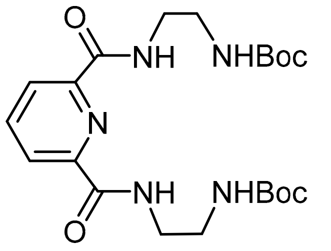

Mw=451,52 g/mol

By starting from **15** and following the procedure C, 3.432 g (76 %) of **18** was synthesized as white solid m.p. 200 °C.
^{1}H NMR (400 MHz, $CDCl_3$) δ 8.95 (s, 2H), 8.27 (d, J=7.75 Hz, 2H), 7.97 (t, J=7.75 Hz, 1H), 5.42 (s, 2H) 3.60-3.59 (m, 4H), 3.47 – 3.45(m, 4H), 1.38 (s, 18H).
^{13}C NMR (100 MHz, $CDCl_3$) δ 164.2, 148.5, 138.8, 124.4, 79.4, 45.8, 41.0, 40.2, 28.3.

Compound 19

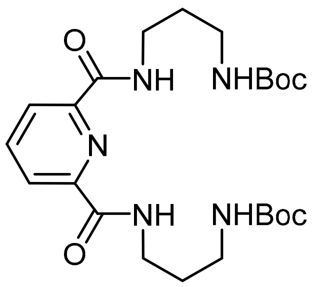

Mw= 479,58 g/mol

By starting from **16** and following the procedure C, 2.733 g (57 %) of **19** was synthesized as white solid m.p. 154 - 155 °C.
^{1}H NMR (400 MHz, $CDCl_3$) δ 8.95 (s, 2H), 8.33 (d, J = 7.7 Hz, 2H), 8.02 (t, J = 7.7 Hz, 1H), 5.08 (s, 2H), 3.57 (m, 4H), 3.29 (m, 4H), 1.74 (m, 4H), 1.45 (s, 18H).
^{13}C NMR (100 MHz, $CDCl_3$) δ 163.9, 156.8, 148.7, 138. 8, 124.5, 79.3, 38.8, 35.4, 30.1, 28.4.

Compound 20

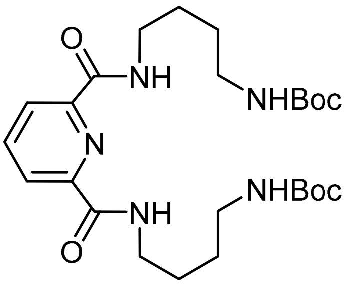

Mw= 507,63 g/mol

By starting from **17** and following the procedure C, 2.640 g (76 %) of **20** was synthesized as white solid m.p. 200 °C.
^{1}H NMR (400 MHz, $CDCl_3$) δ 8.34 (d, J=7.8 Hz, 2H), 8.24 (bs, 2H), 8.01 (t, J=7.8, 1H), 4.77 (bs, 2H), 3.51 - 3.48 (m, 4H), 3.18 - 3.16 (m, 4H), 1.69 - 1.65 (m, 4H), 1.59 - 1.56 (m, 4H), 1.42 (s, 18H).
^{13}C NMR (100 MHz, $CDCl_3$) δ 163.8, 156.2, 149.0, 138.8, 124.9, 79.2, 40.2, 39.4, 28.4, 27.7, 26.7.

3. Copies of the NMR spectra

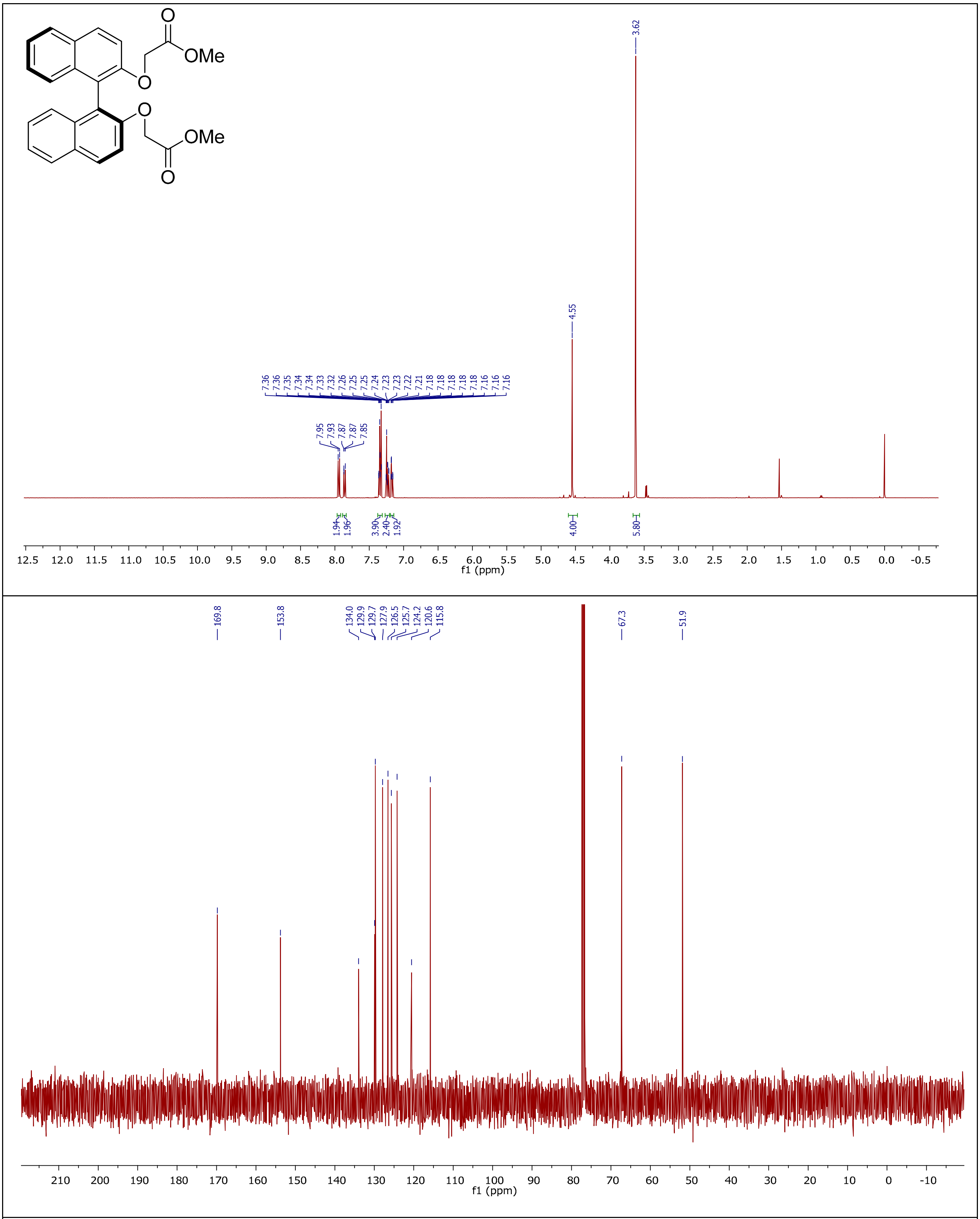

Fig. S1 ^{1}H NMR (400 MHz) and ^{13}C NMR (100 MHz) spectra of compound (*S*)-**4** in $CDCl_3$.

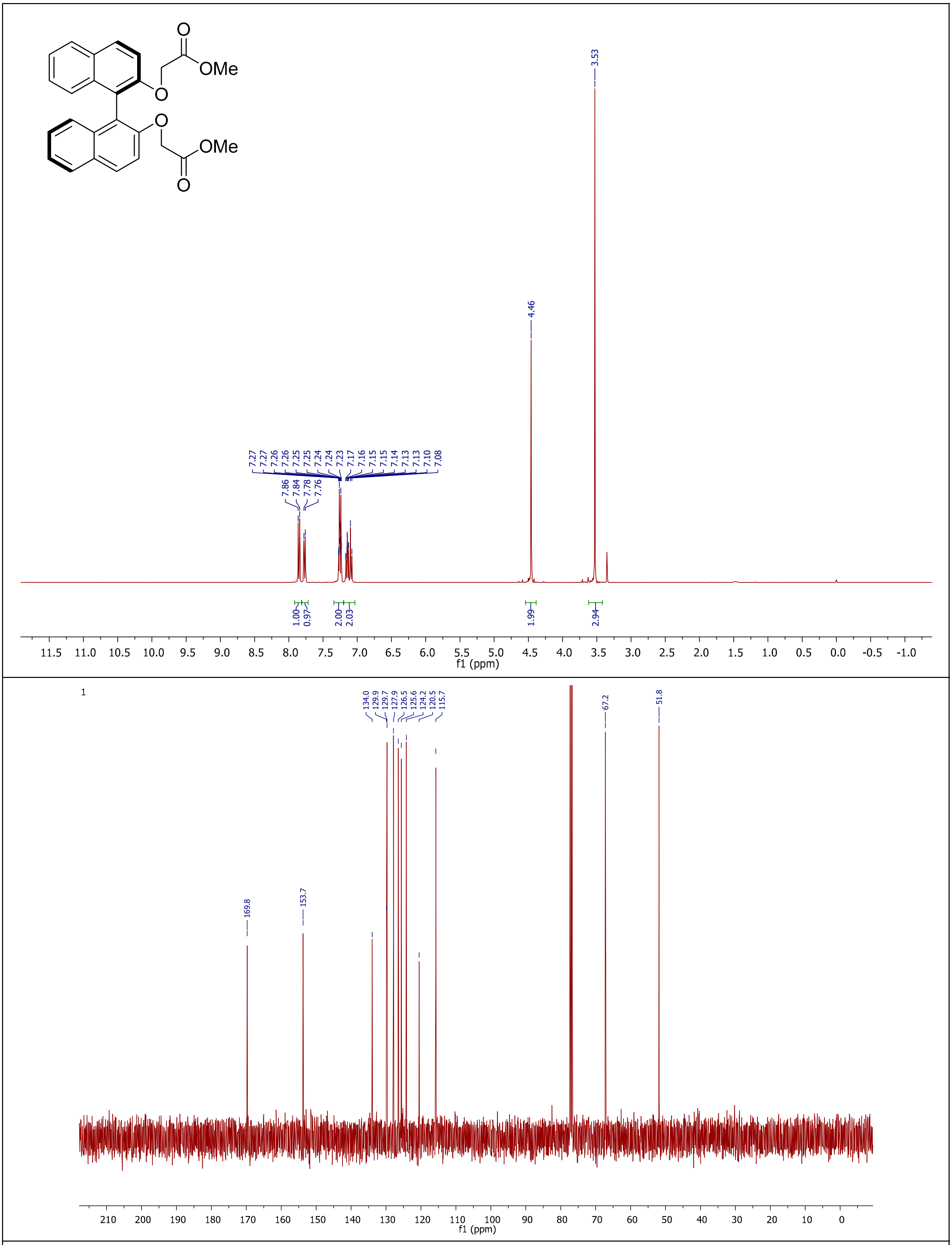

Fig. S2 ^{1}H NMR (400 MHz) and ^{13}C NMR (100 MHz) spectra of compound (*R*)-**4** in $CDCl_3$.

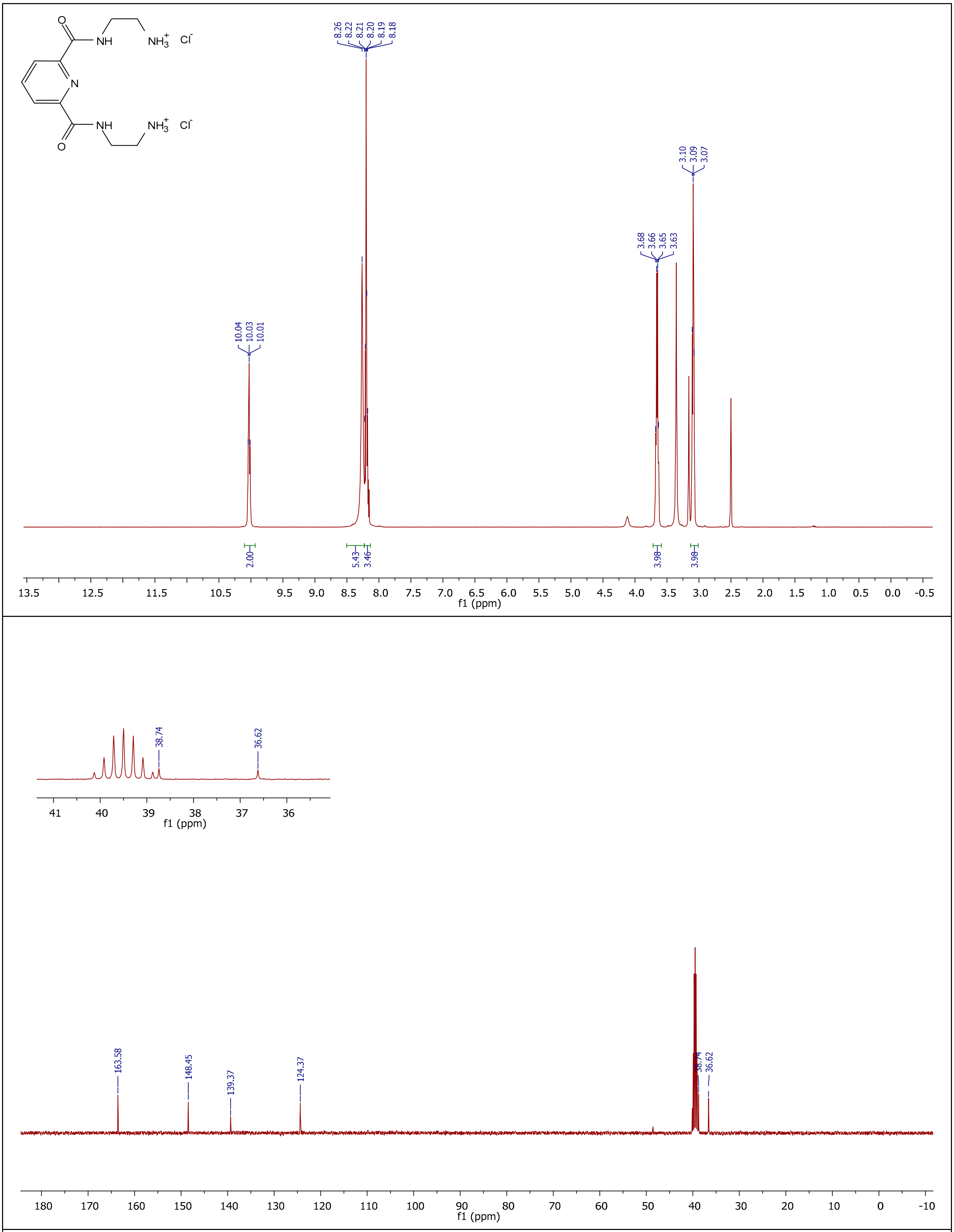

Fig. S3 ^{1}H NMR (400 MHz) and ^{13}C NMR (100 MHz) spectra of compound **5** in DMSO

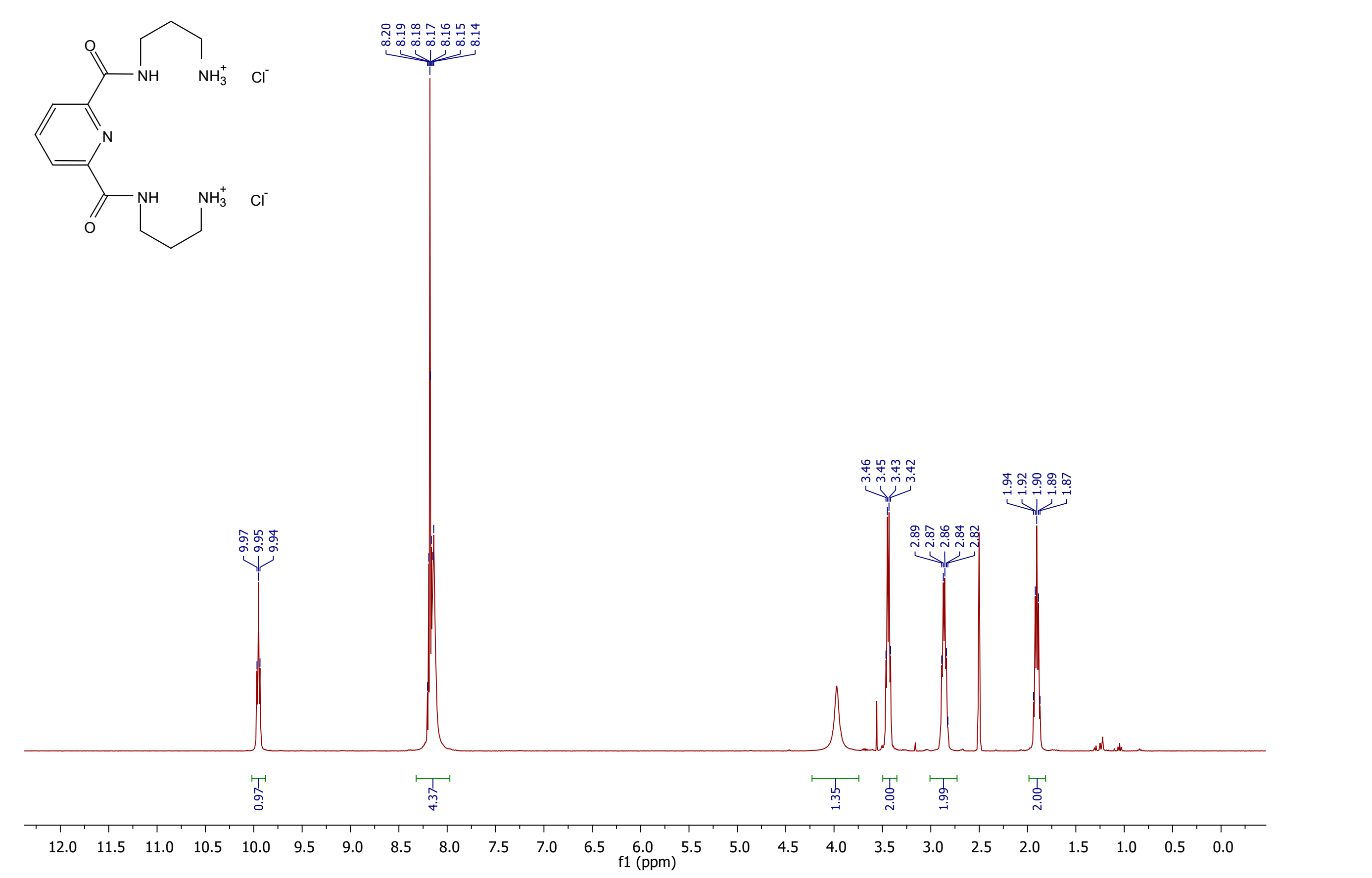

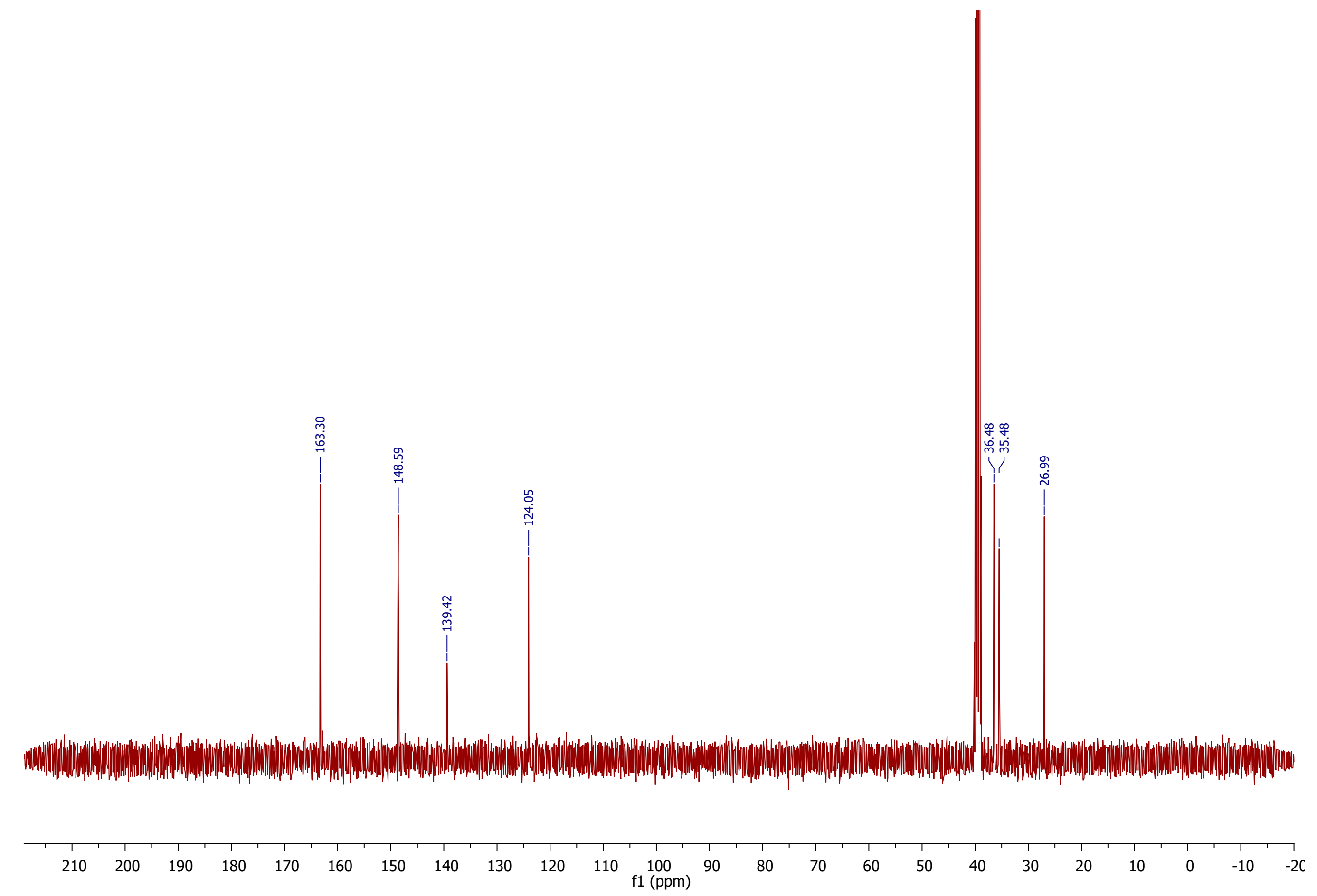

Fig. S4 ^{1}H NMR (400 MHz) and ^{13}C NMR (100 MHz) spectra of compound **6** in DMSO

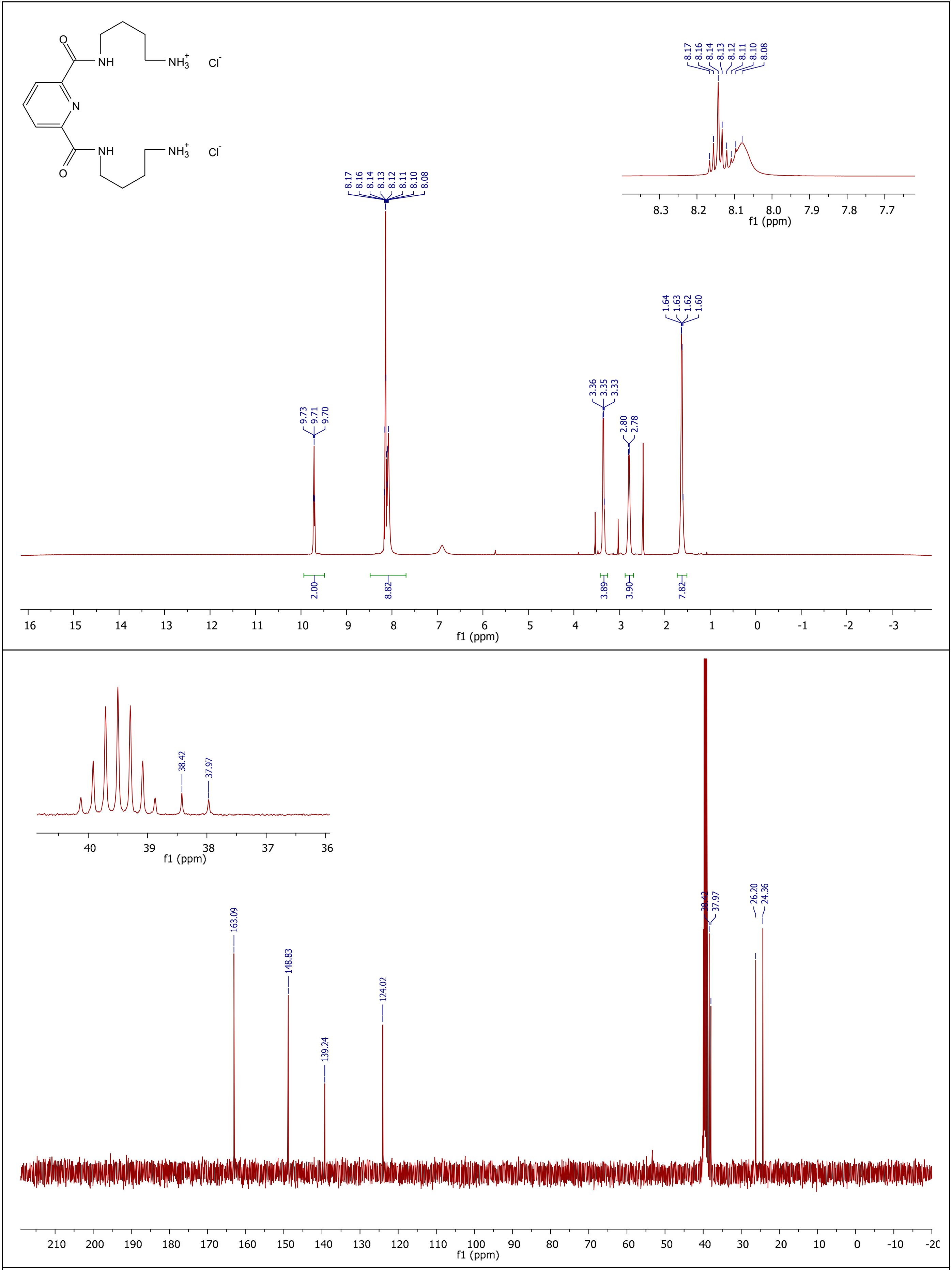

Fig. S5 ^{1}H NMR (400 MHz) and ^{13}C NMR (100 MHz) spectra of compound **7** in DMSO

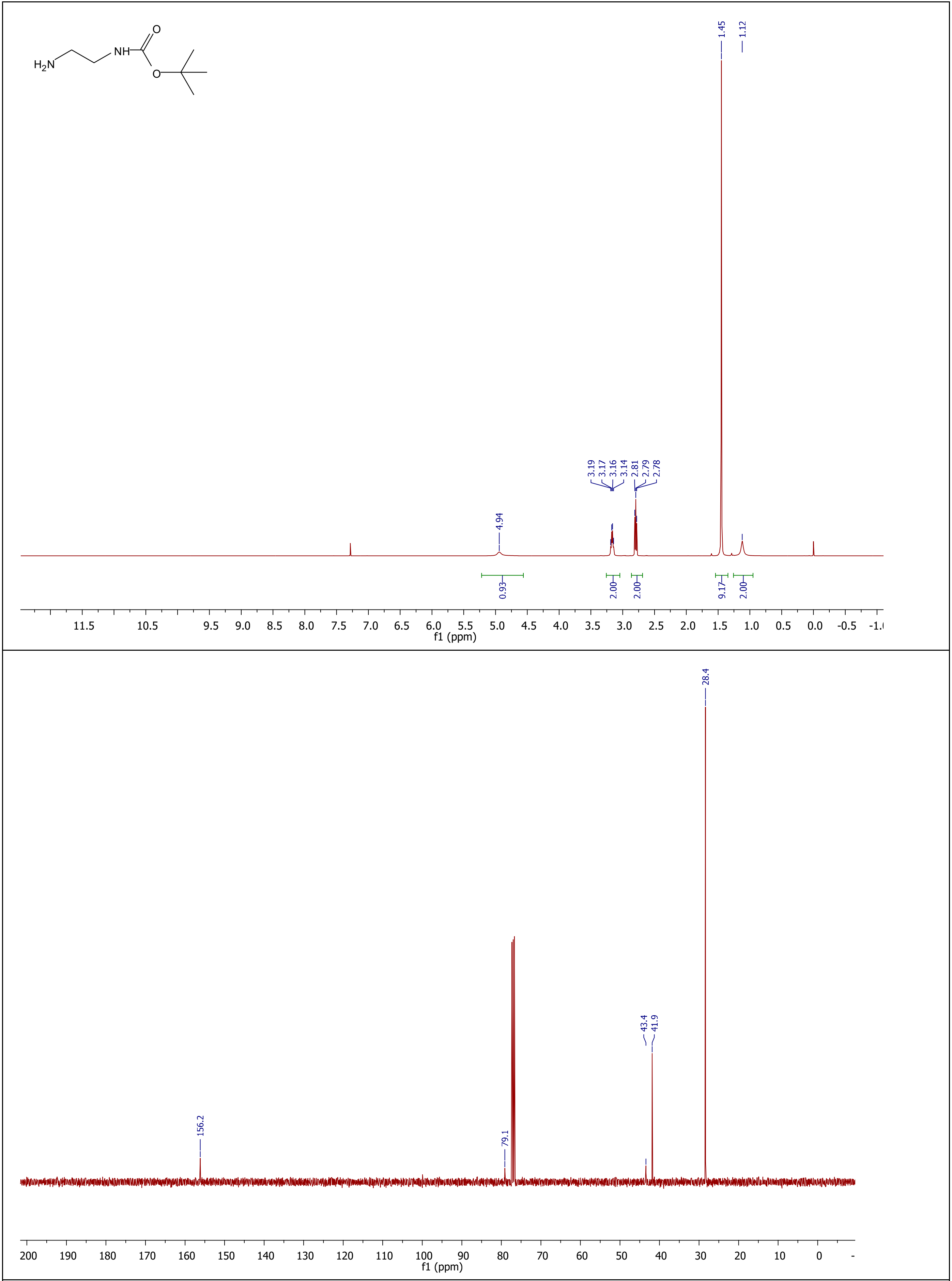

Fig. S6 ^{1}H NMR (400 MHz) and ^{13}C NMR (100 MHz) spectra of compound **15** in $CDCl_3$.

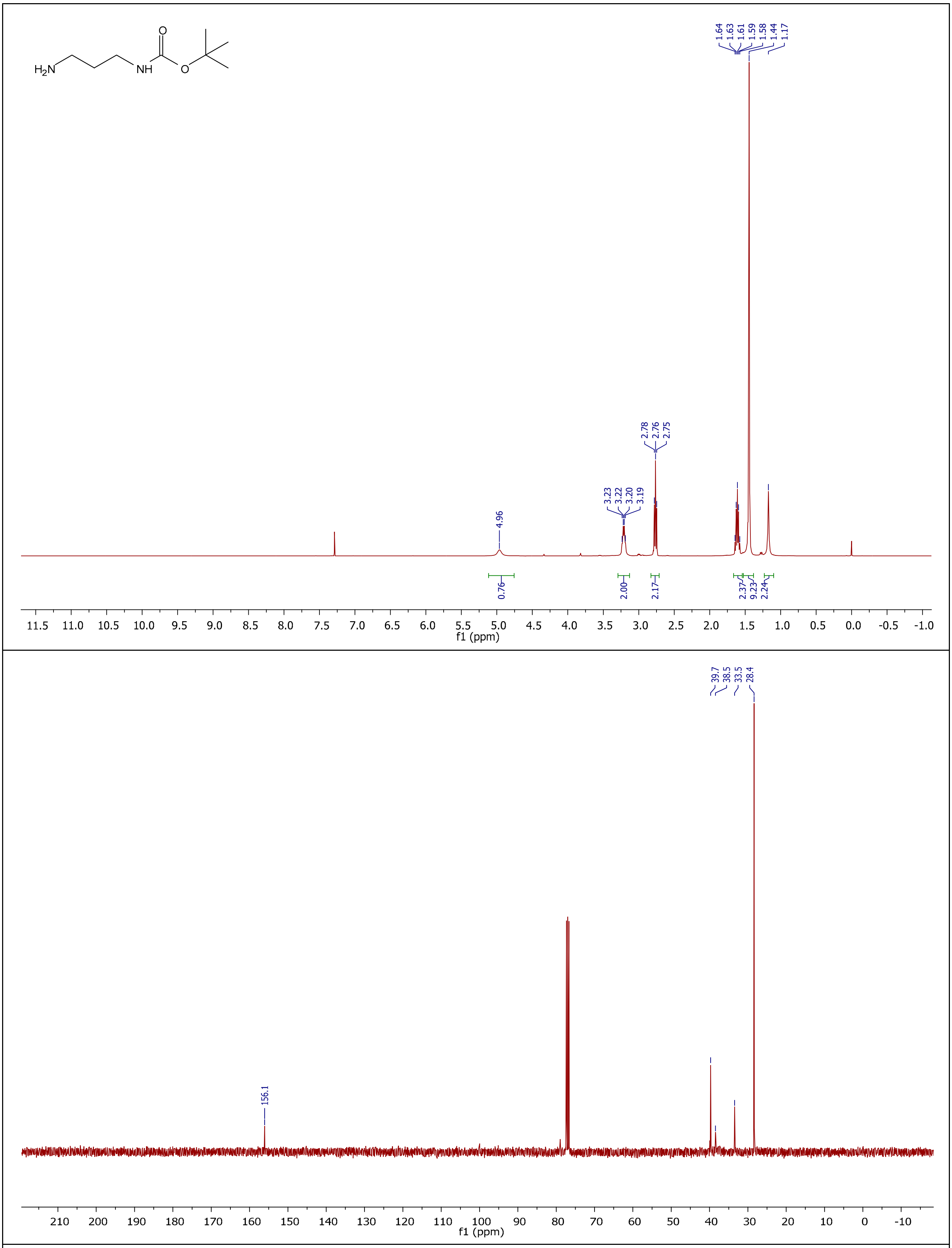

Fig. S7 ^{1}H NMR (400 MHz) and ^{13}C NMR (100 MHz) spectra of compound **16** in $CDCl_3$.

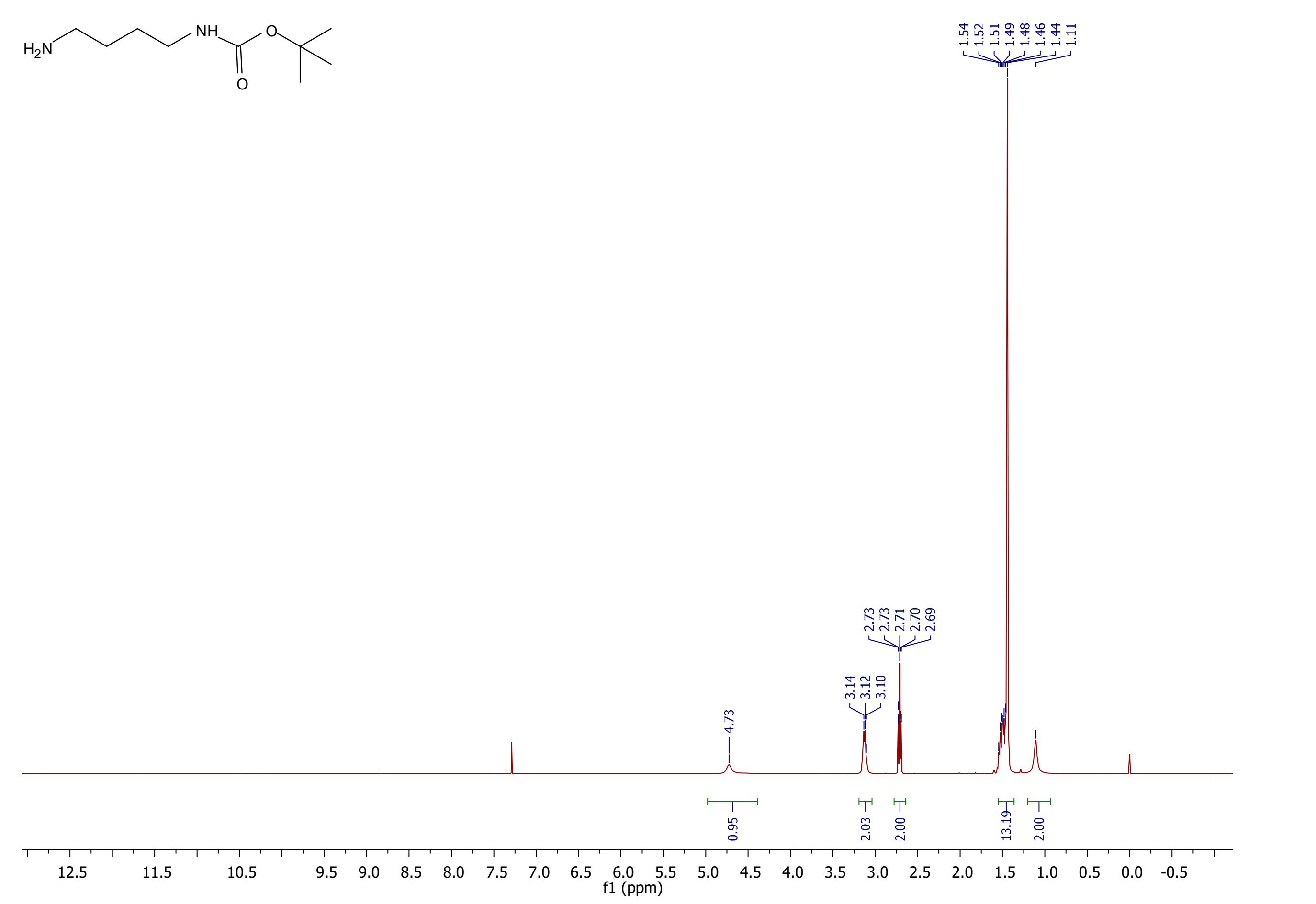

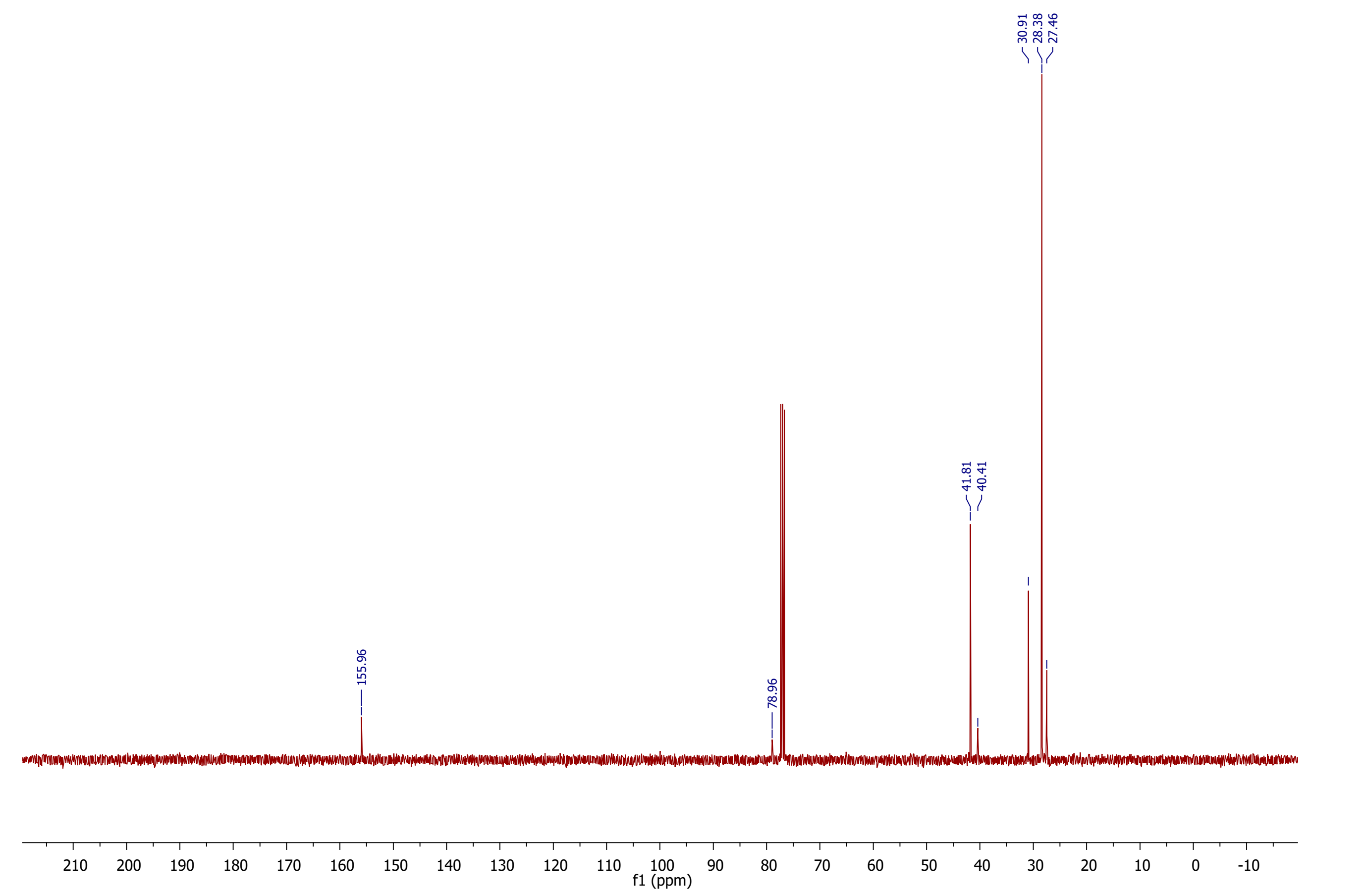

Fig. S8 ^{1}H NMR (400 MHz) and ^{13}C NMR (100 MHz) spectra of compound **17** in $CDCl_3$.

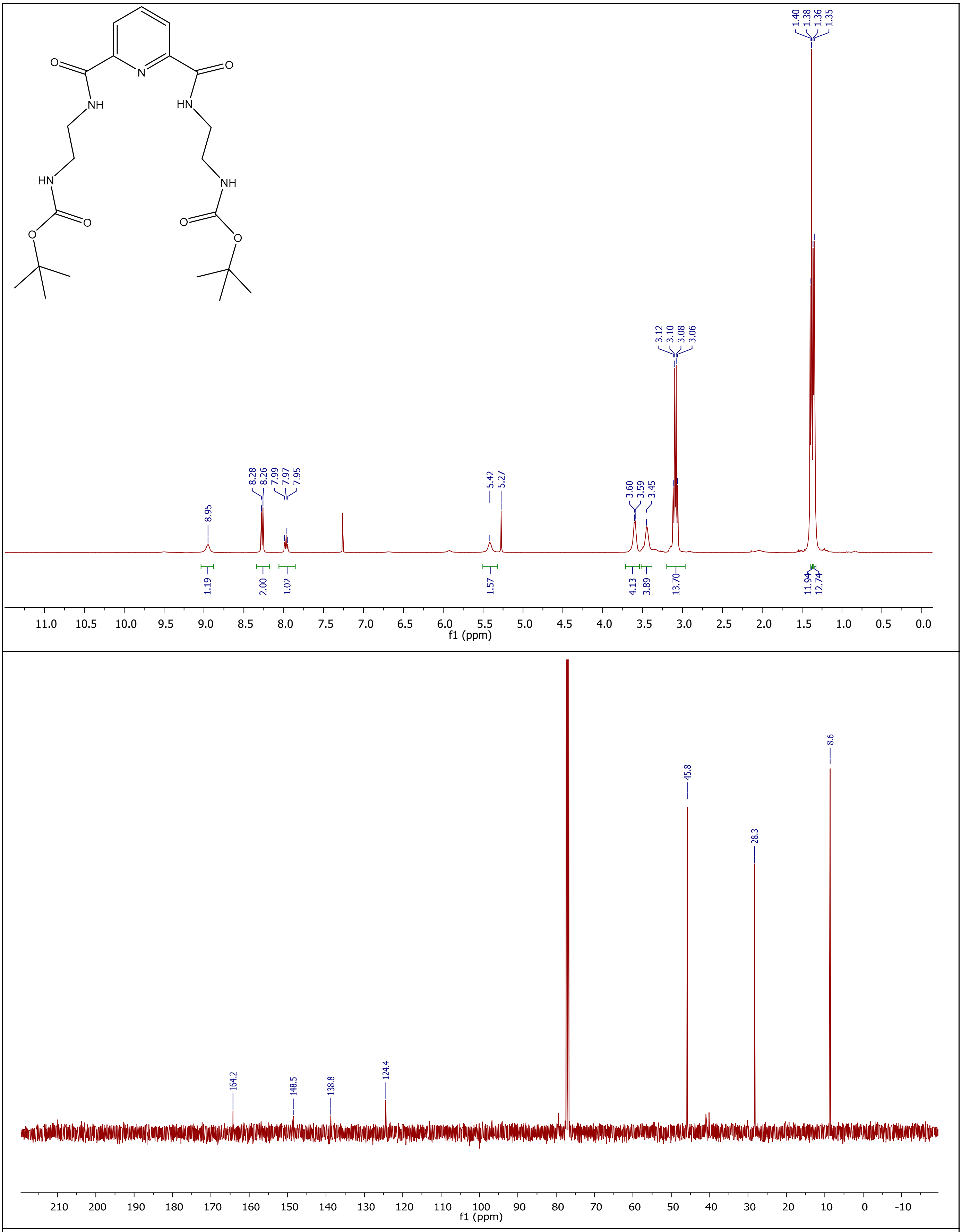

Fig. S9 ^{1}H NMR (400 MHz) and ^{13}C NMR (100 MHz) spectra of compound **18** in $CDCl_3$.

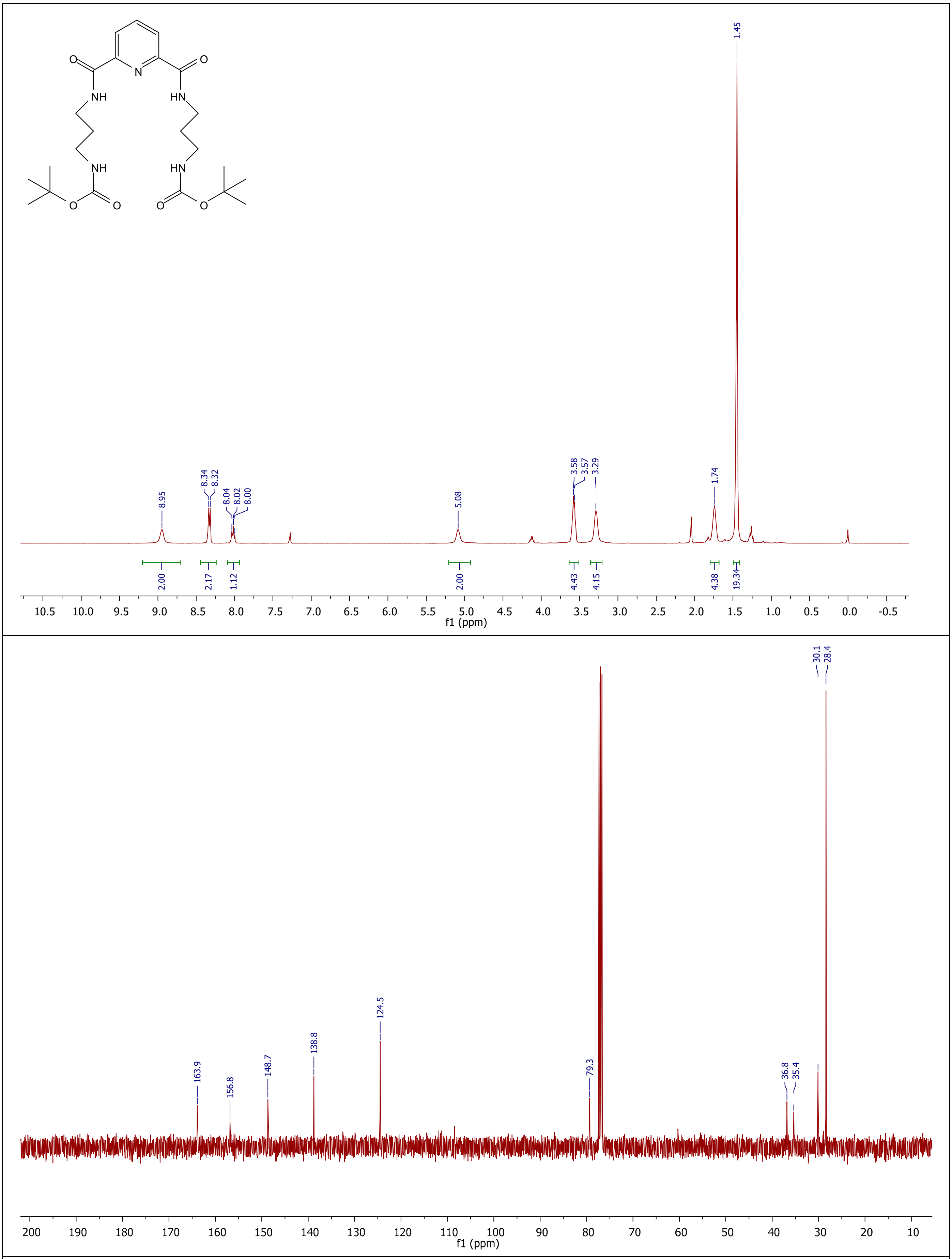

Fig. S10 ^{1}H NMR (400 MHz) and ^{13}C NMR (100 MHz) spectra of compound **19** in $CDCl_3$.

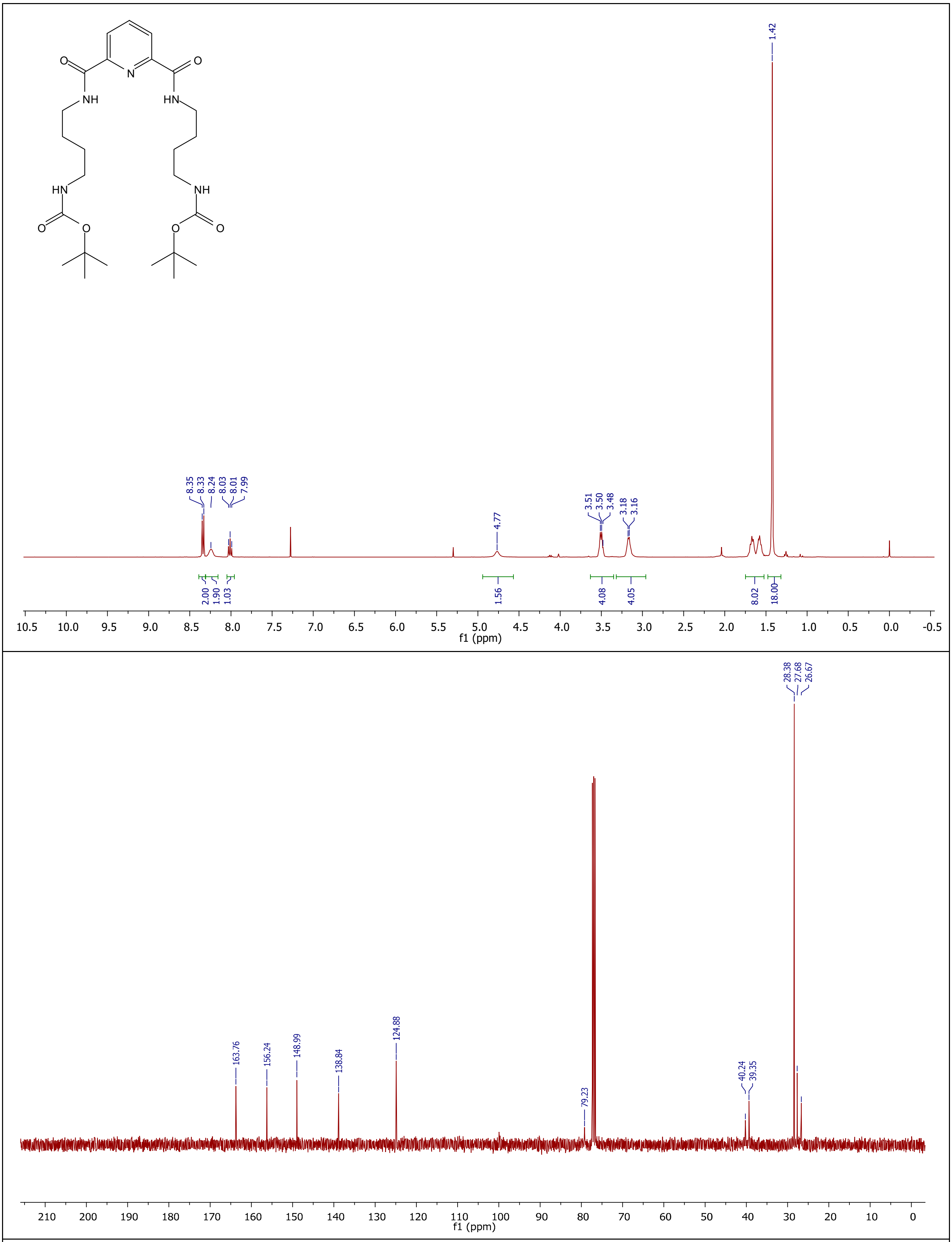

Fig. S11 ^{1}H NMR (400 MHz) and ^{13}C NMR (100 MHz) spectra of compound **20** in $CDCl_3$.

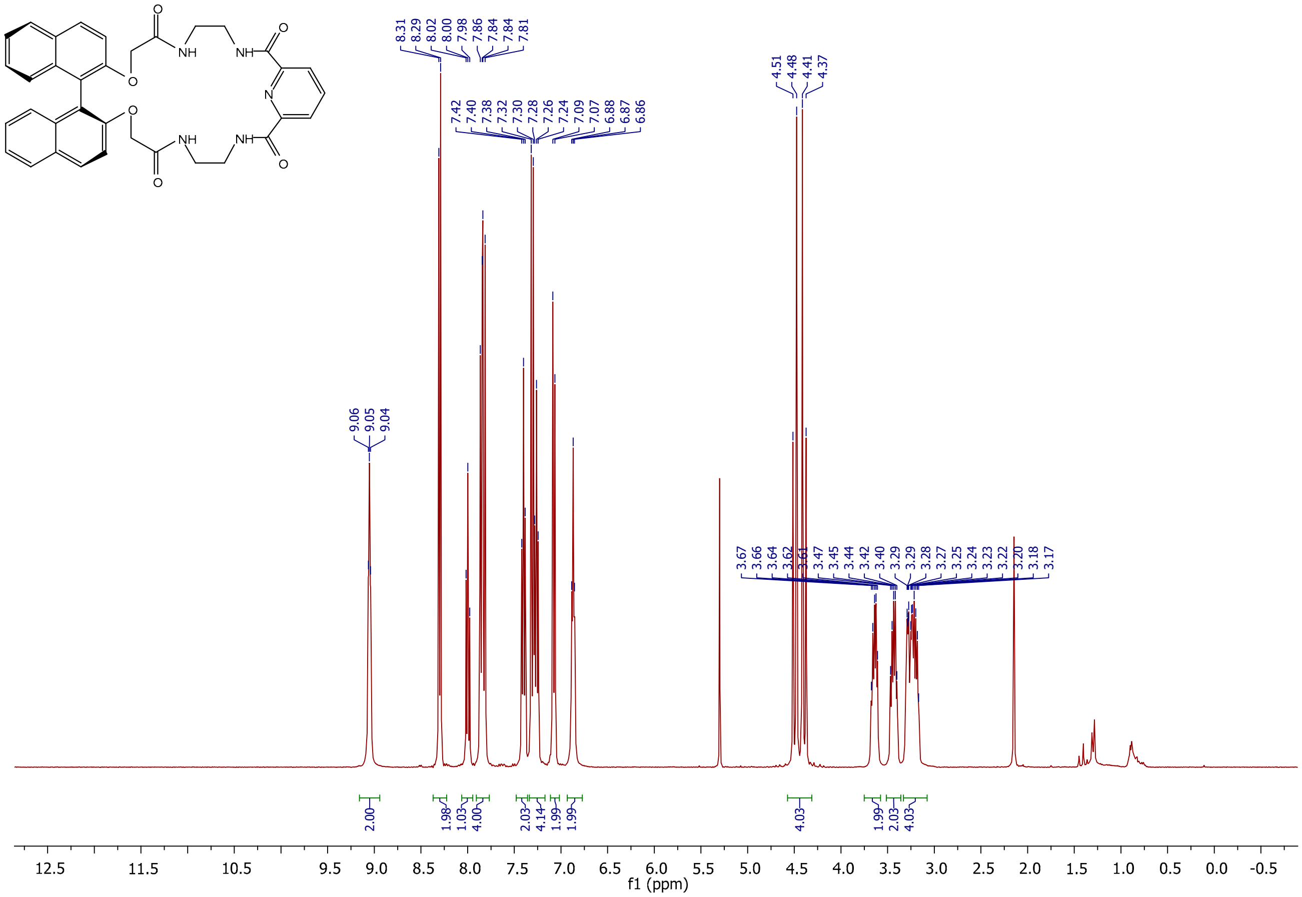

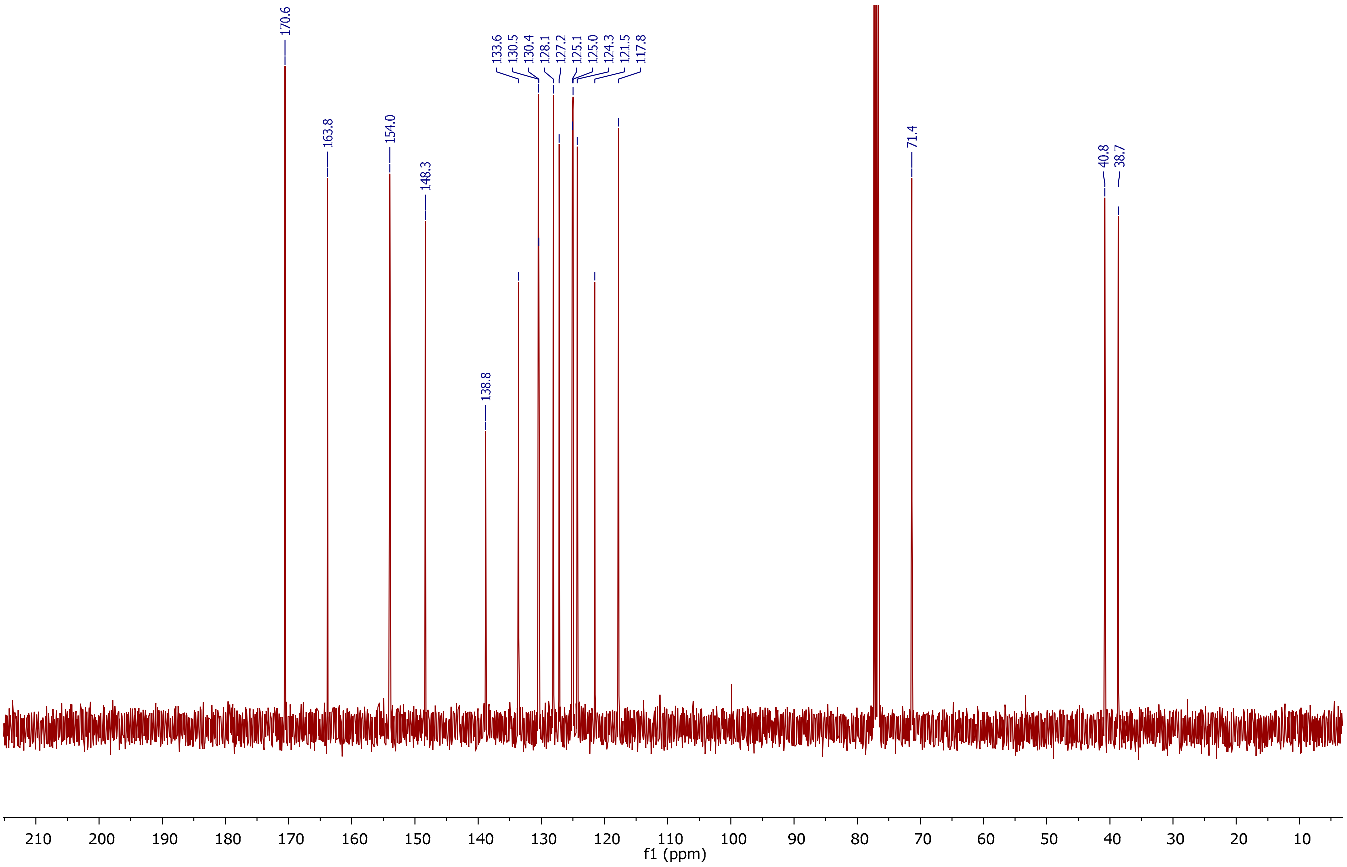

Fig. S12 ^{1}H NMR (400 MHz) and ^{13}C NMR (100 MHz) spectra of compound (*S*)-**1** in $CDCl_3$.

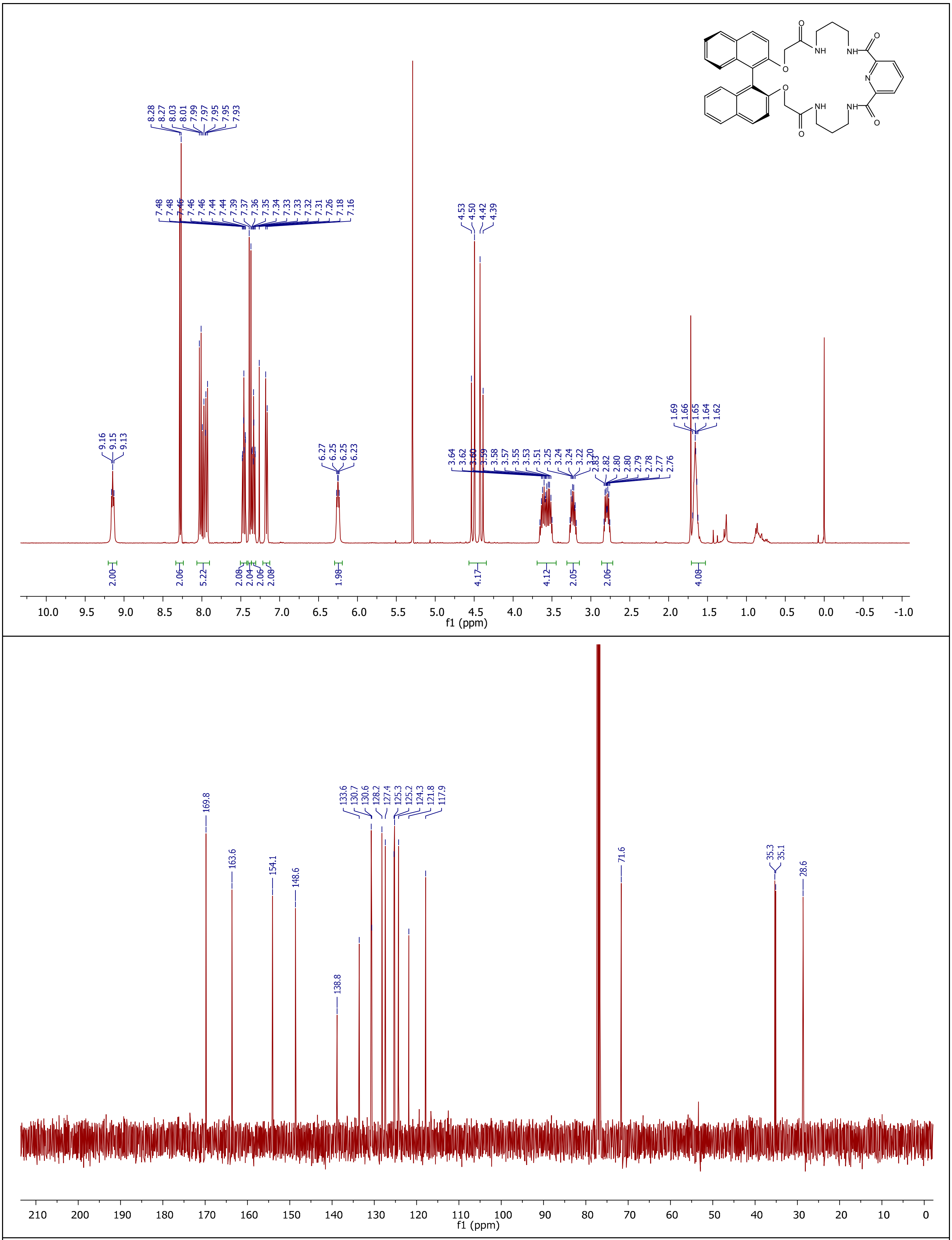

Fig. S13 ^{1}H NMR (400 MHz) and ^{13}C NMR (100 MHz) spectra of compound (*S*)-**2** in $CDCl_3$.

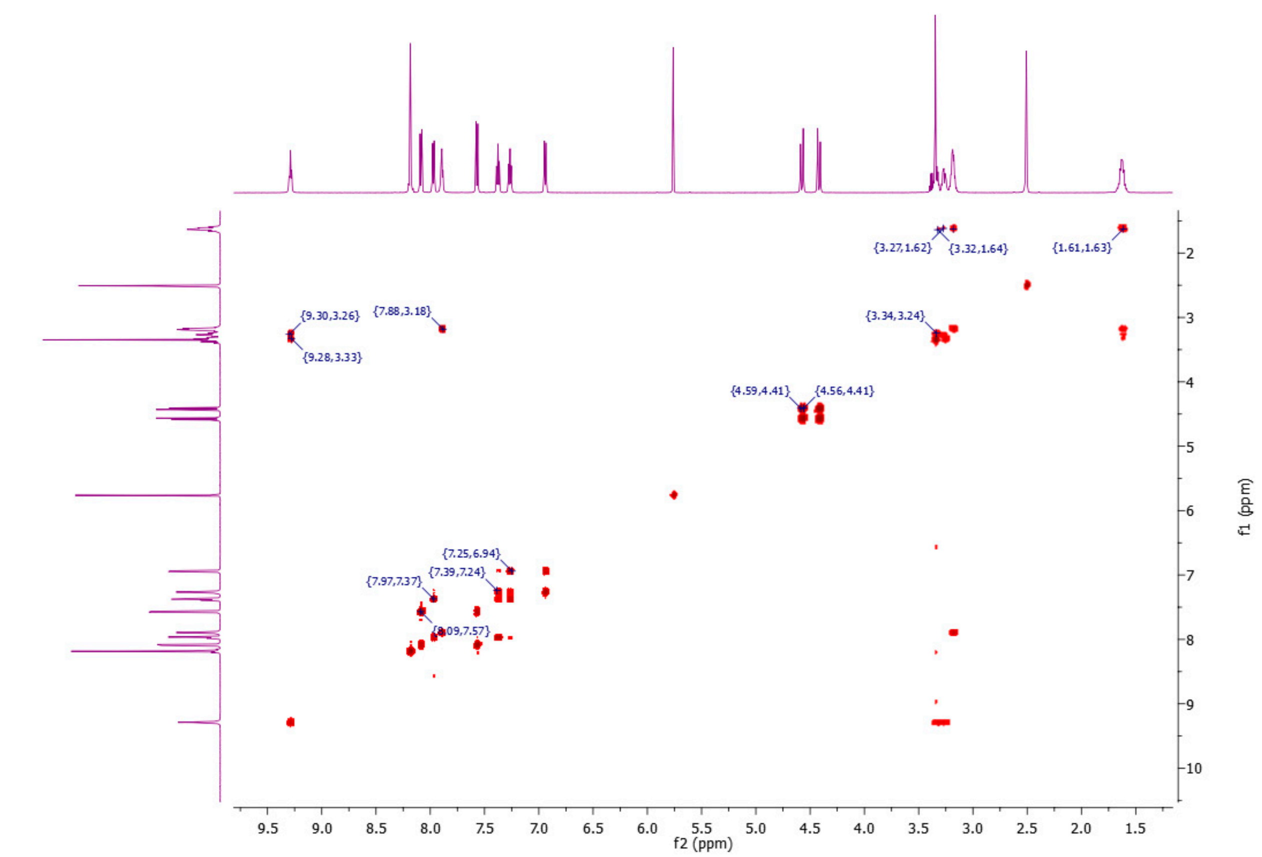

Fig. S14 COSY spectrum (600 MHz) of compound (*S*)-**2** in $CDCl_3$.

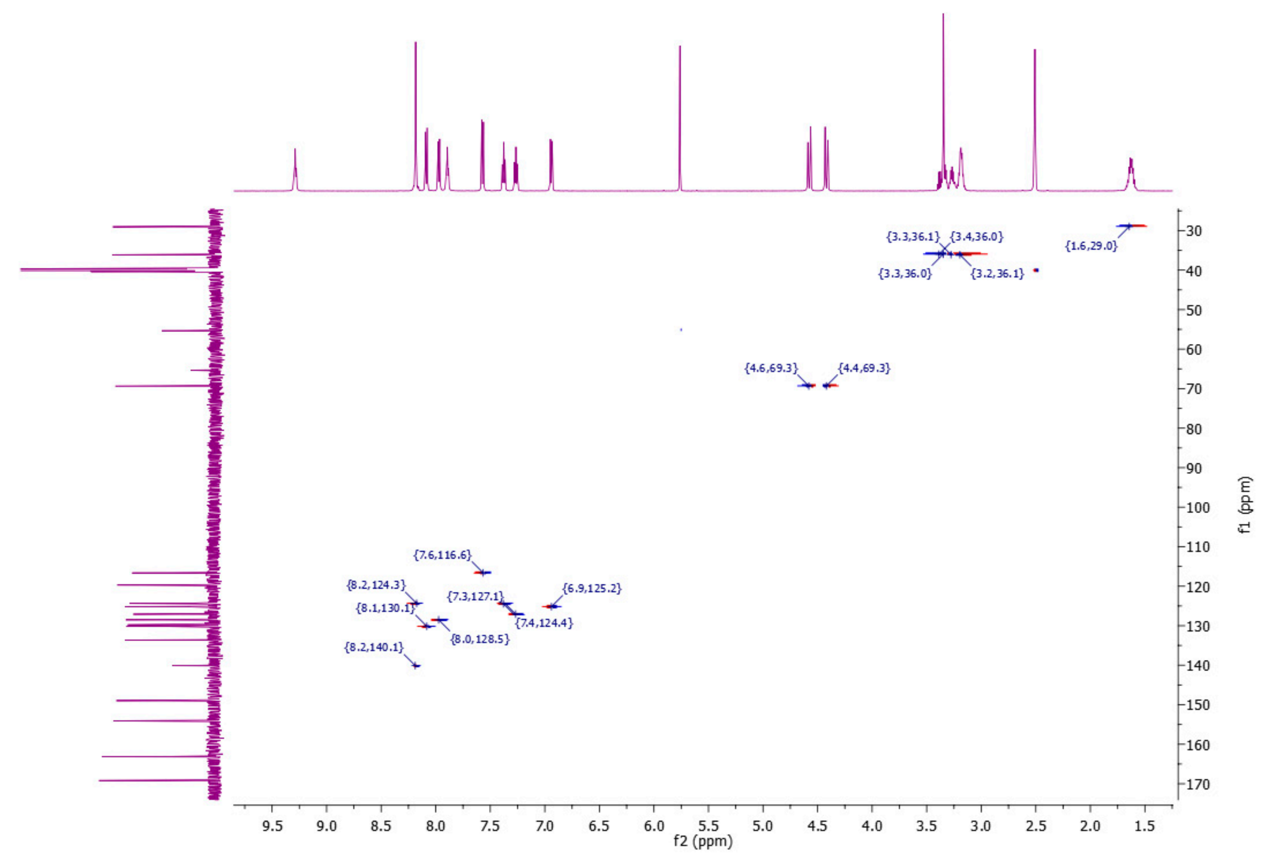

Fig. S15 HSQC spectrum (600 MHz) of compound (*S*)-**2** in $CDCl_3$.

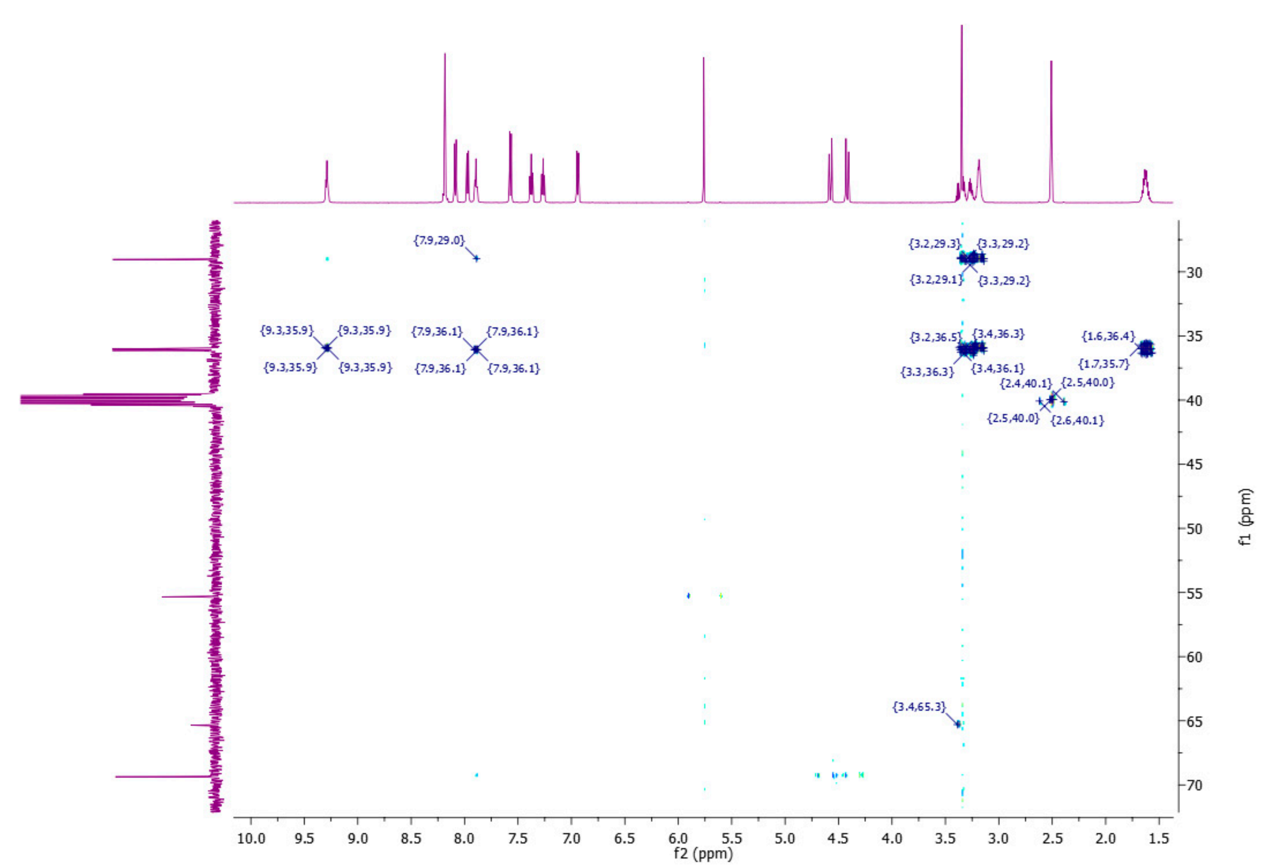

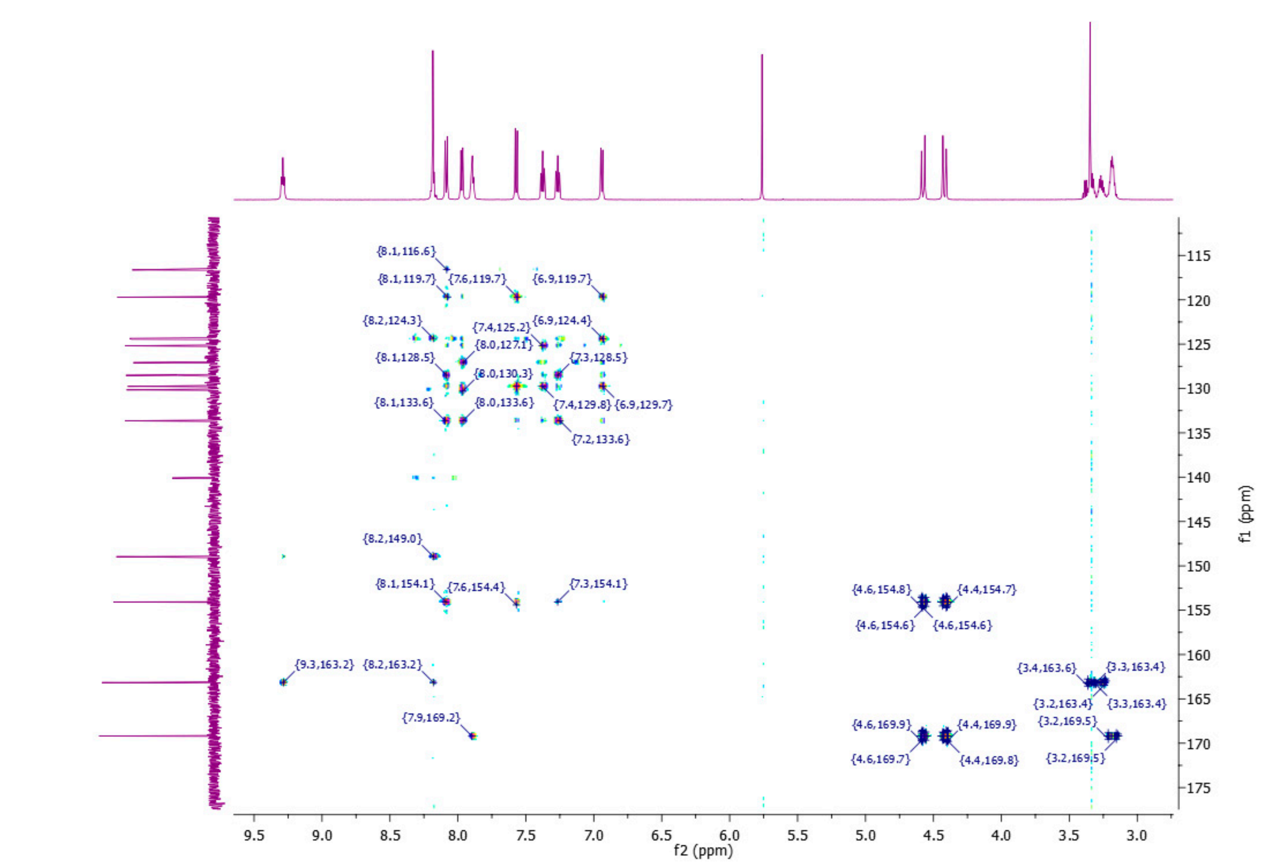

Fig. S16 HMBC spectrum (600 MHz) of compound (*S*)-**2** in $CDCl_3$.

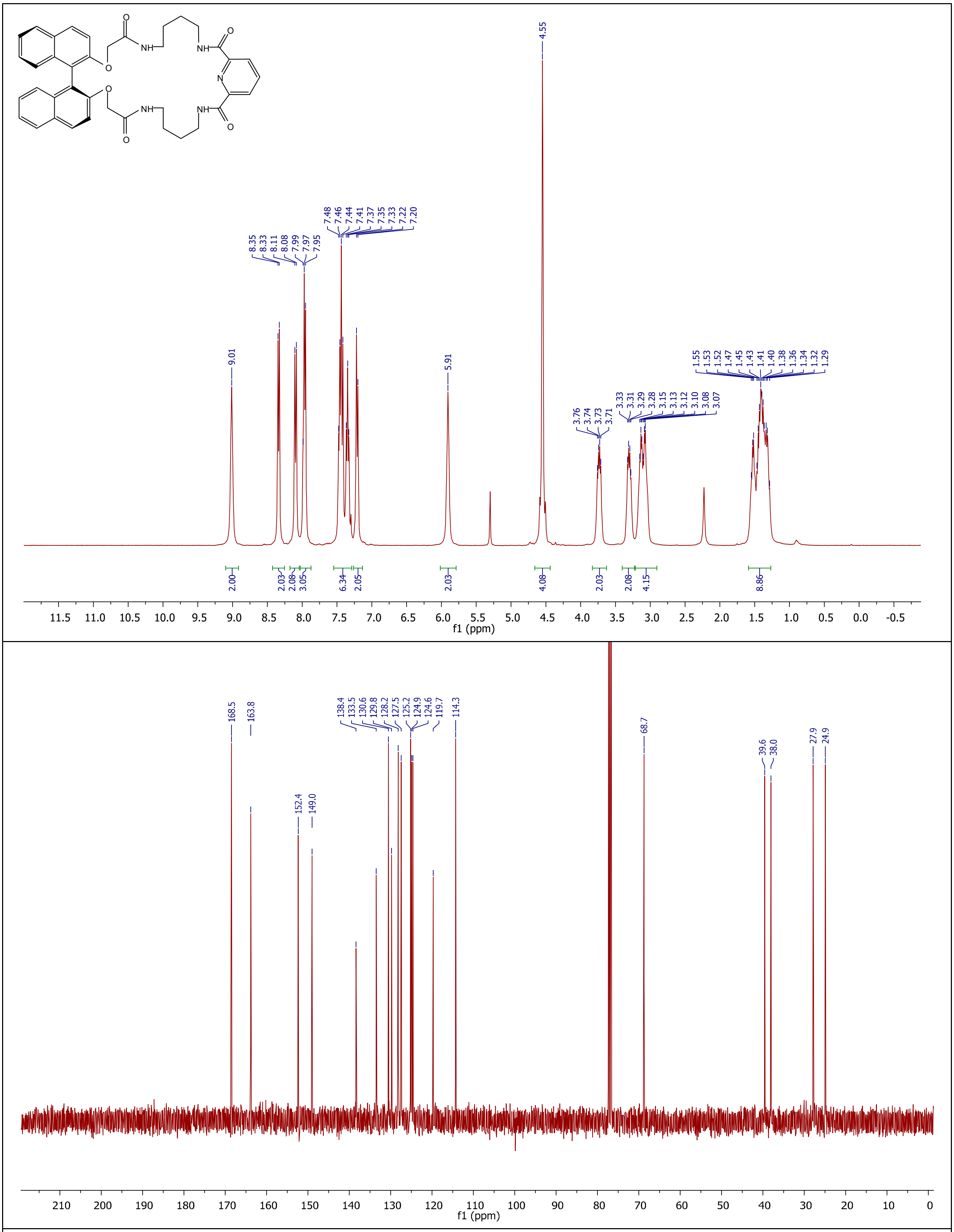

Fig. S17 ^{1}H NMR (400 MHz) and ^{13}C NMR (100 MHz) spectra of compound (*S*)-**3** in $CDCl_3$.

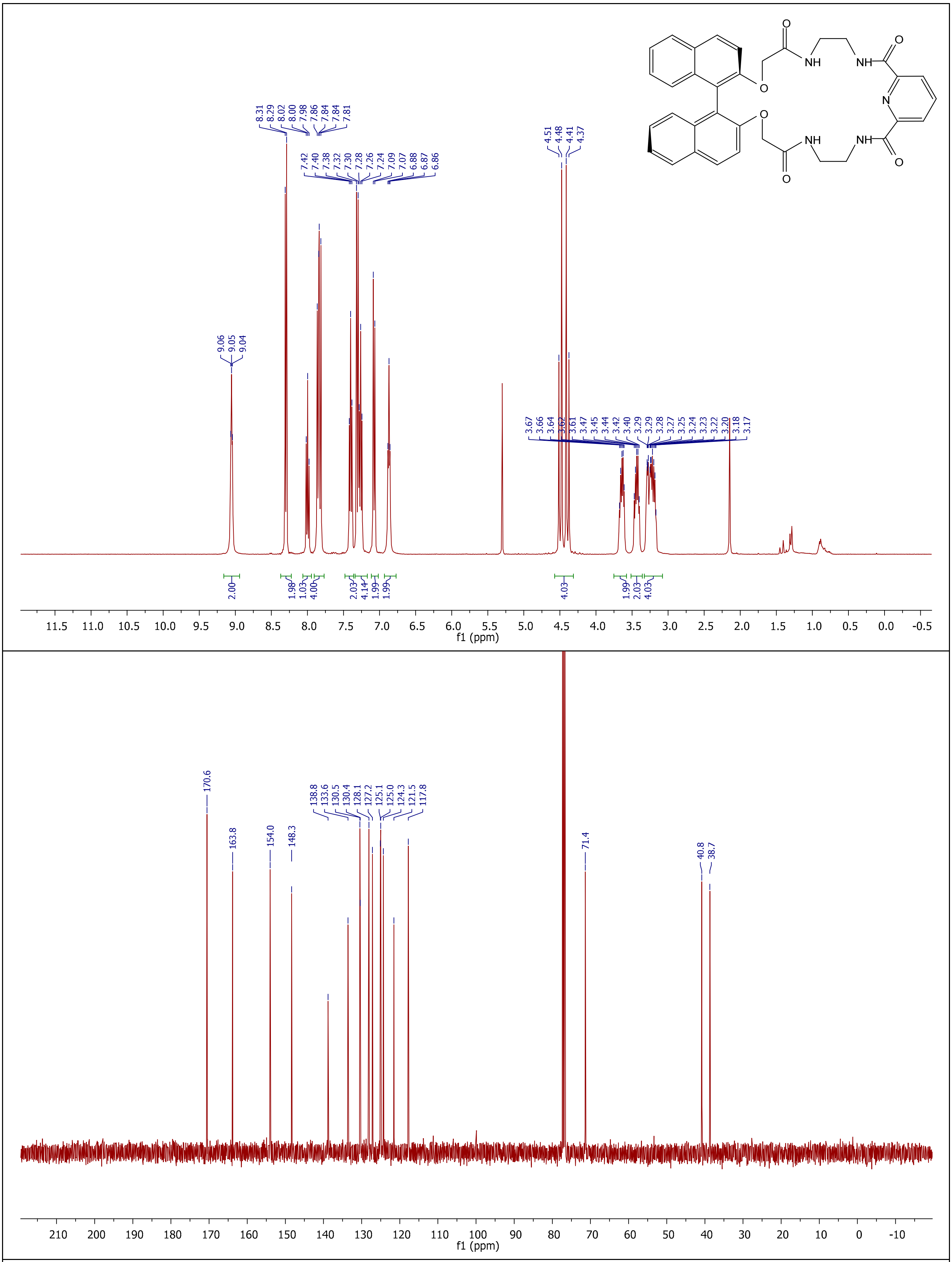

Fig. S18 ^{1}H NMR (400 MHz) and ^{13}C NMR (100 MHz) spectra of compound (*R*)-**1** in $CDCl_3$.

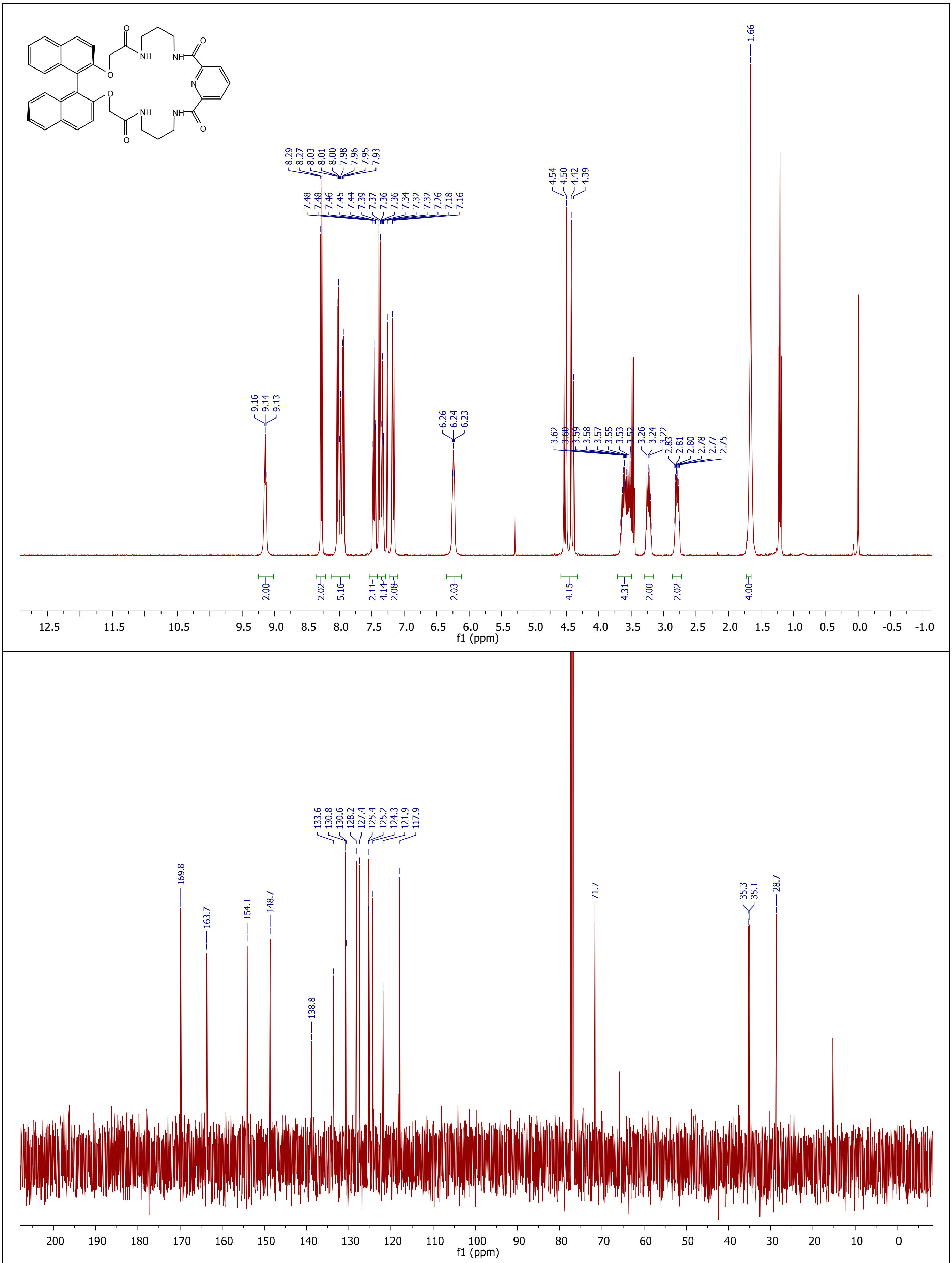

Fig. S19 ^{1}H NMR (400 MHz) and ^{13}C NMR (100 MHz) spectra of compound (*R*)-**2** in $CDCl_3$.

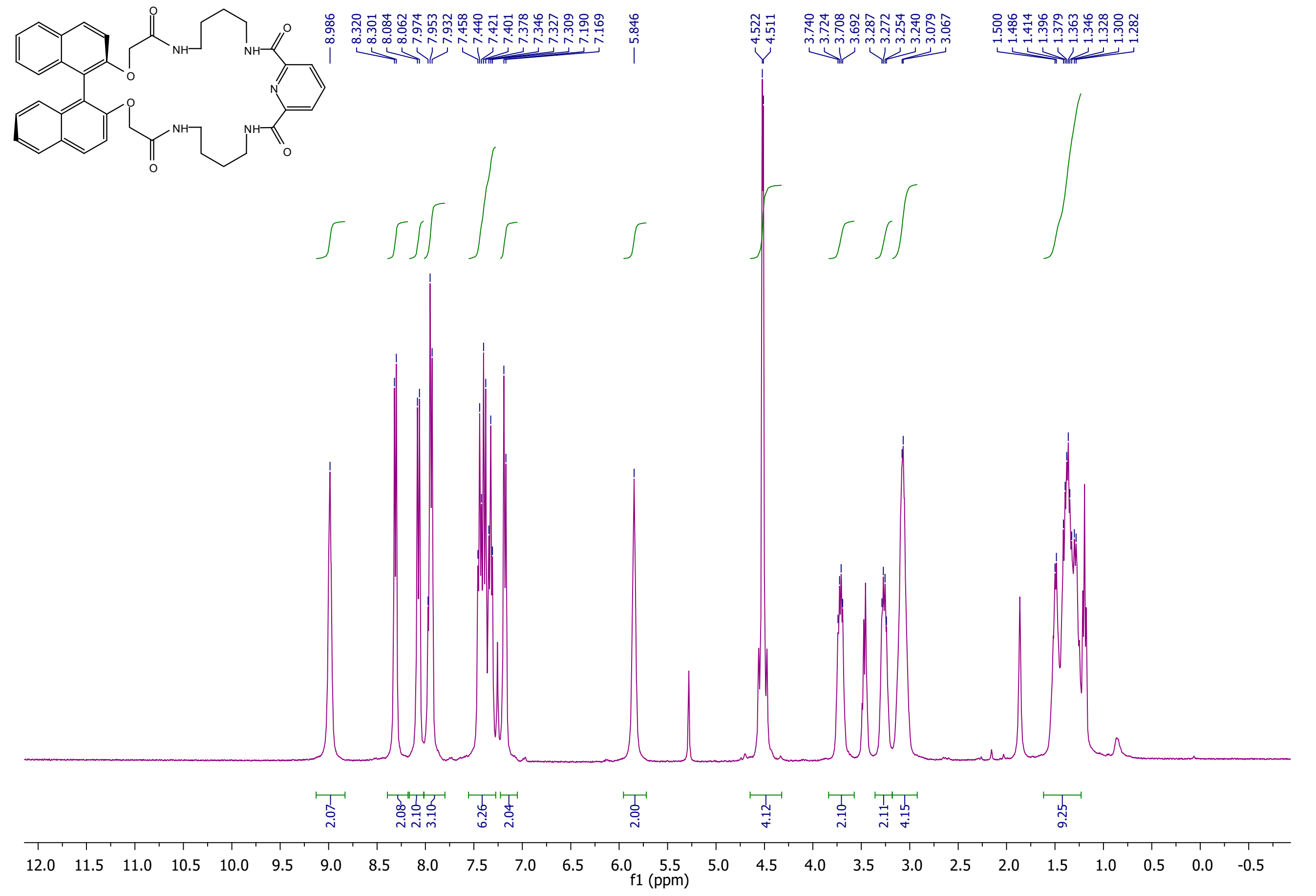

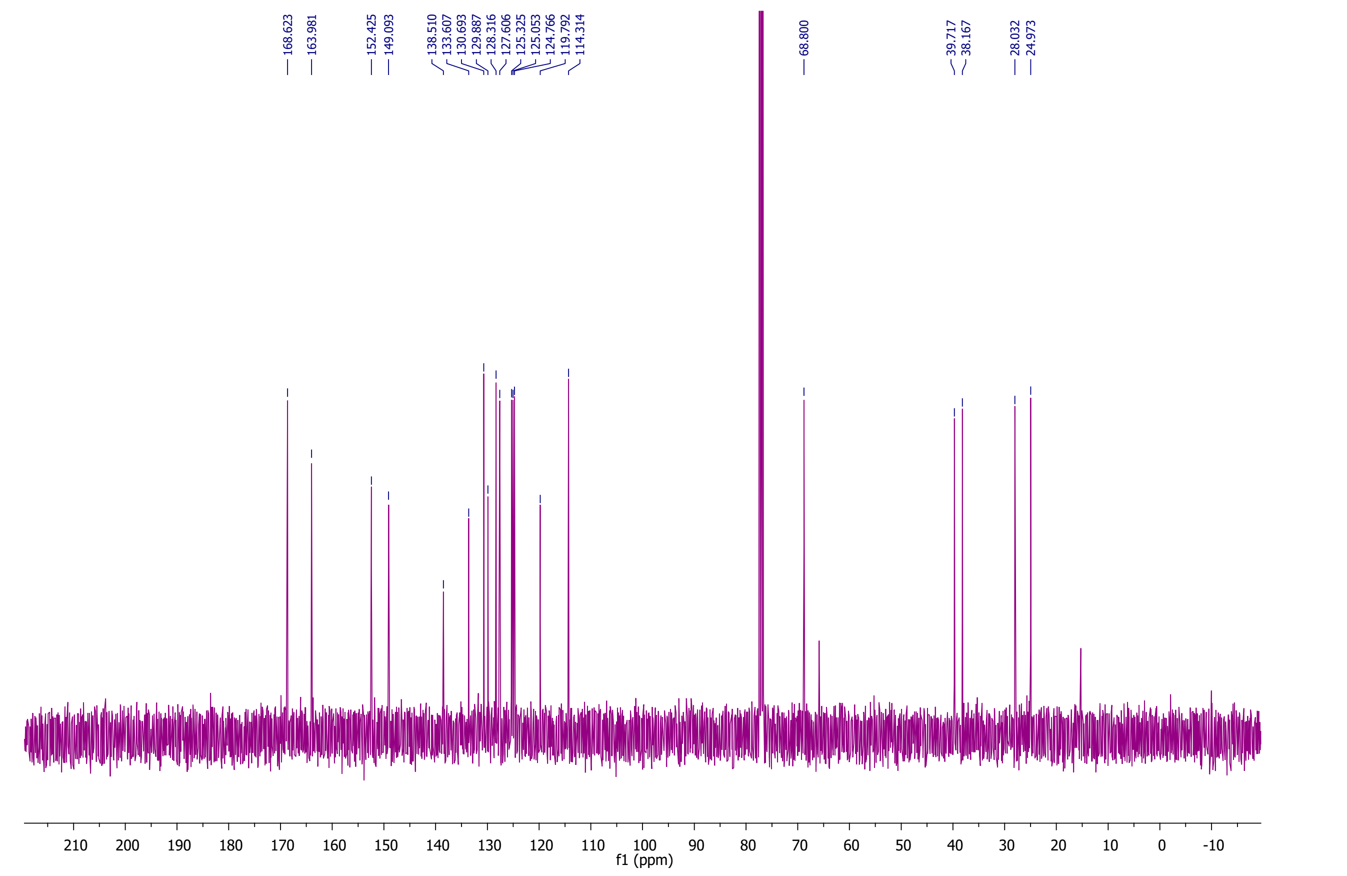

Fig. S20 ^{1}H NMR (400 MHz) and ^{13}C NMR (100 MHz) spectra of compound (*R*)-**3** in $CDCl_3$.

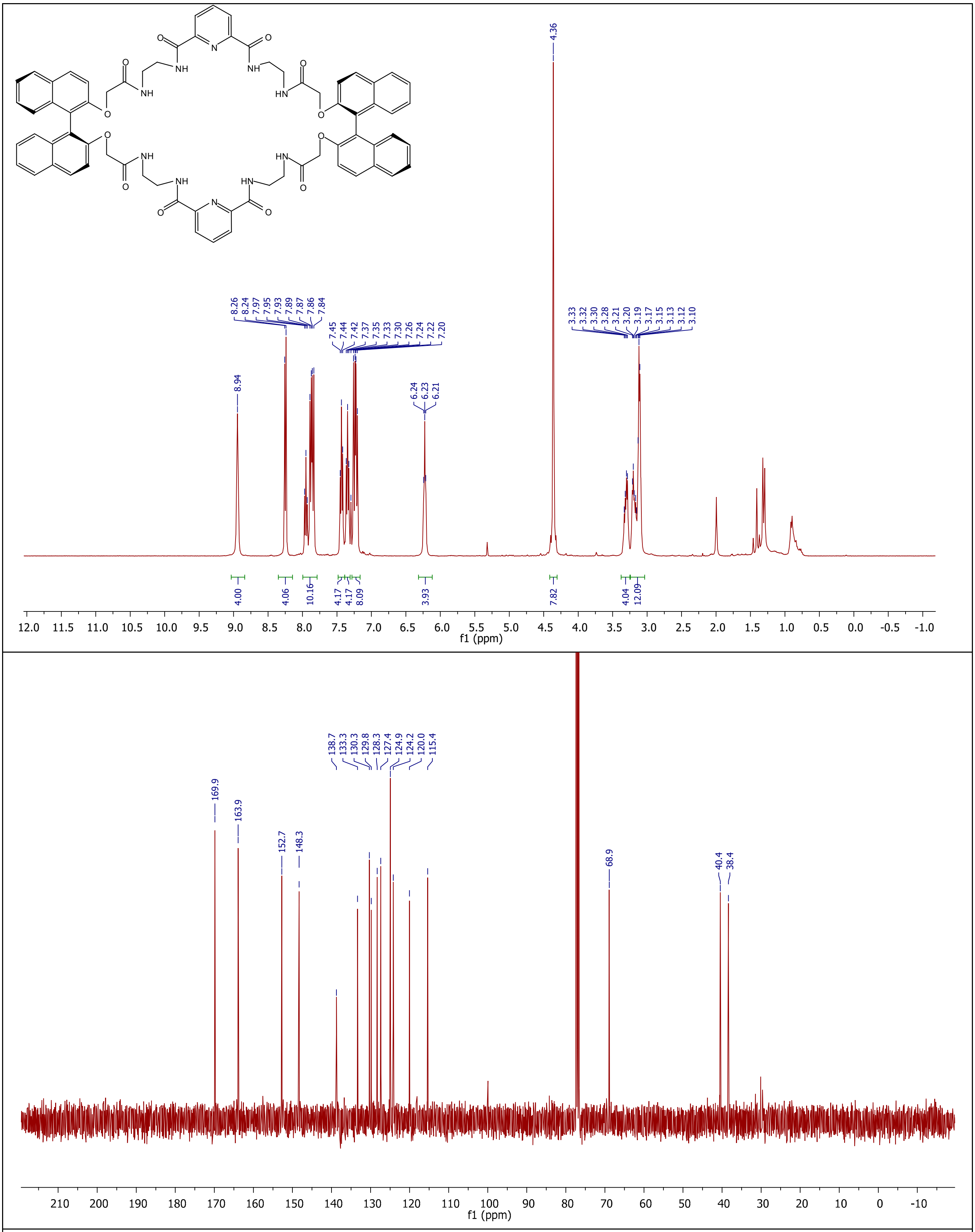

Fig. S21 ^{1}H NMR (400 MHz) and ^{13}C NMR (100 MHz) spectra of compound (*S*)-**8** in $CDCl_3$.

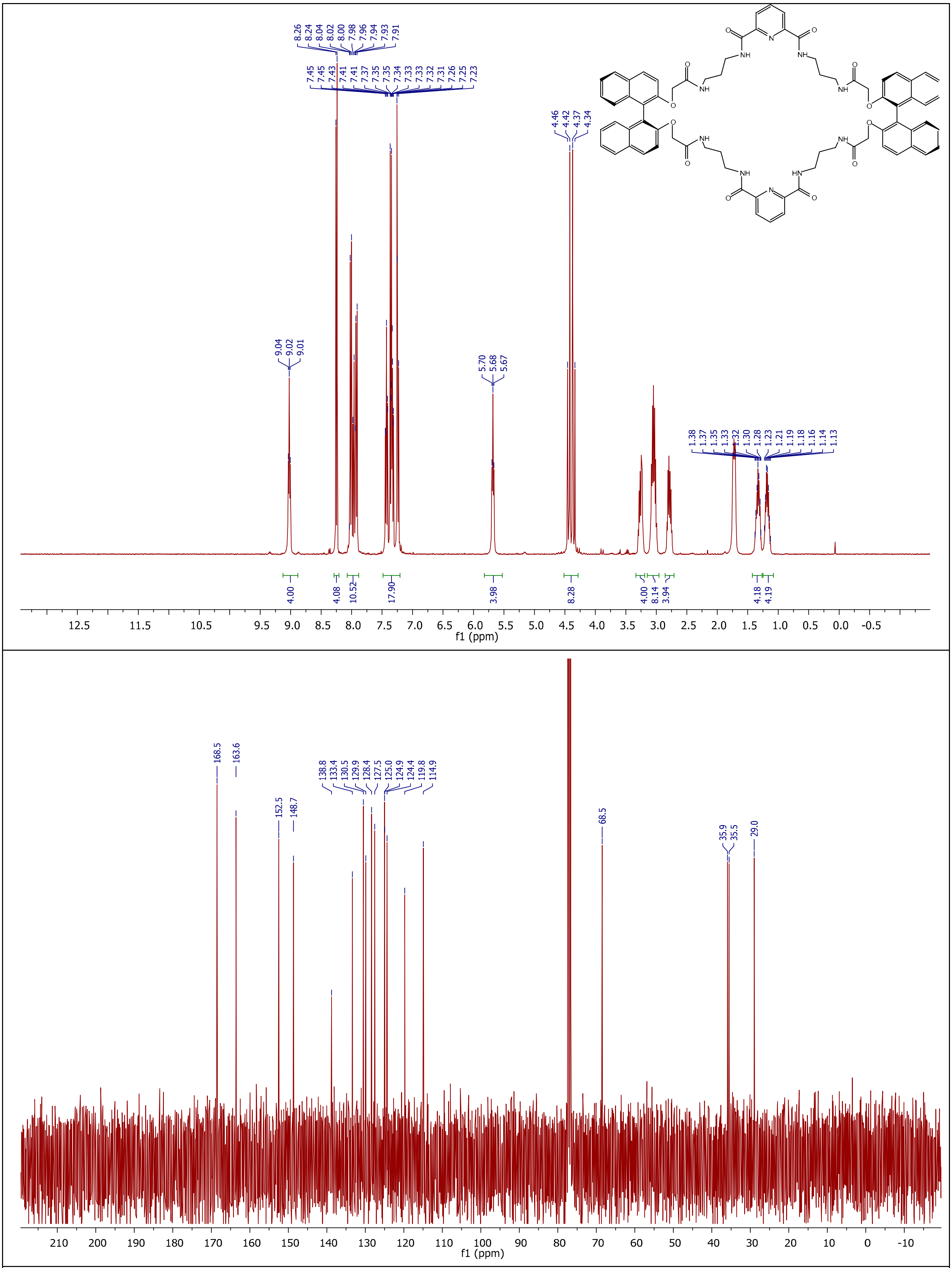

Fig. S22 ^{1}H NMR (400 MHz) and ^{13}C NMR (100 MHz) spectra of compound (*S*)-**9** in $CDCl_3$.

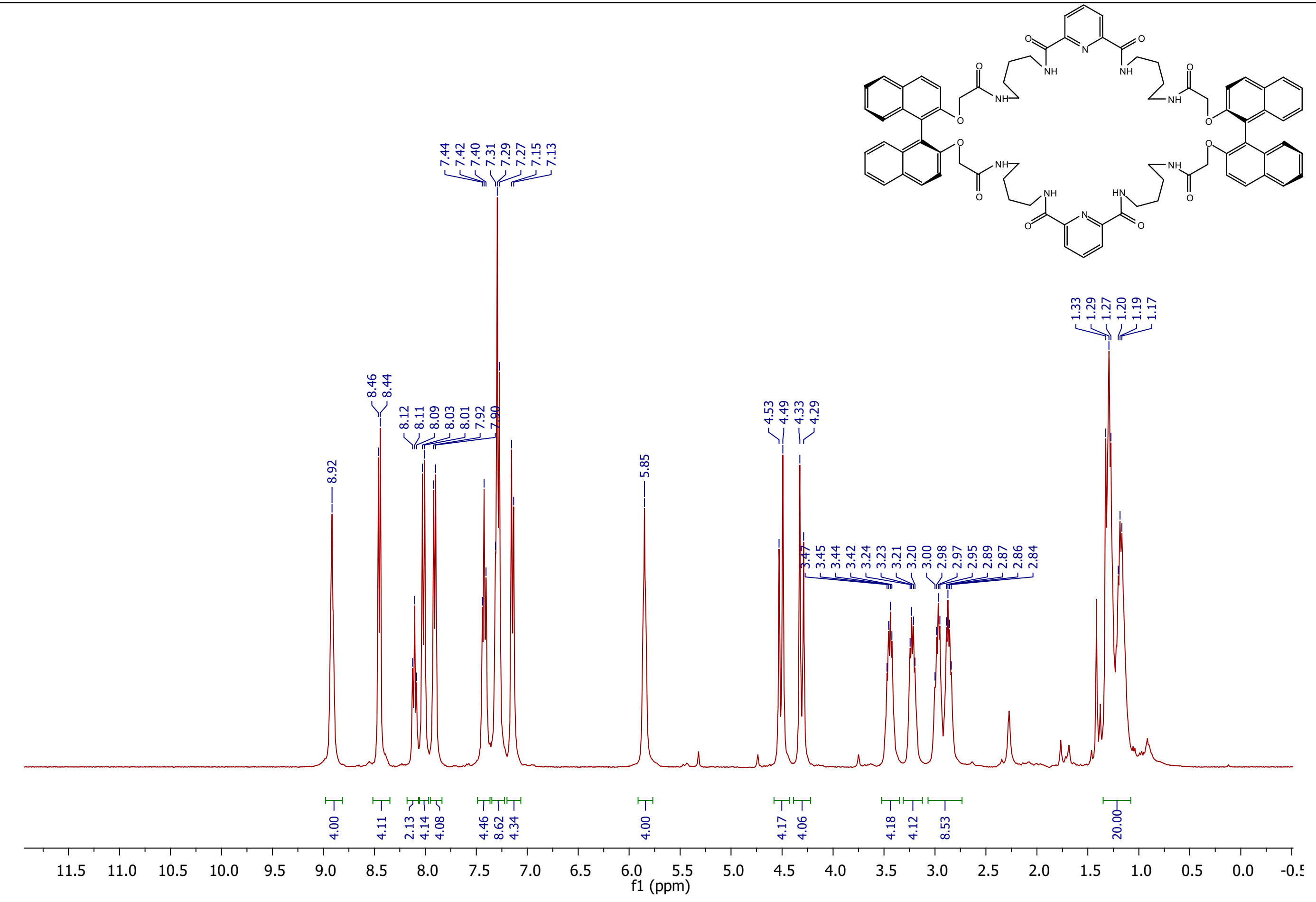

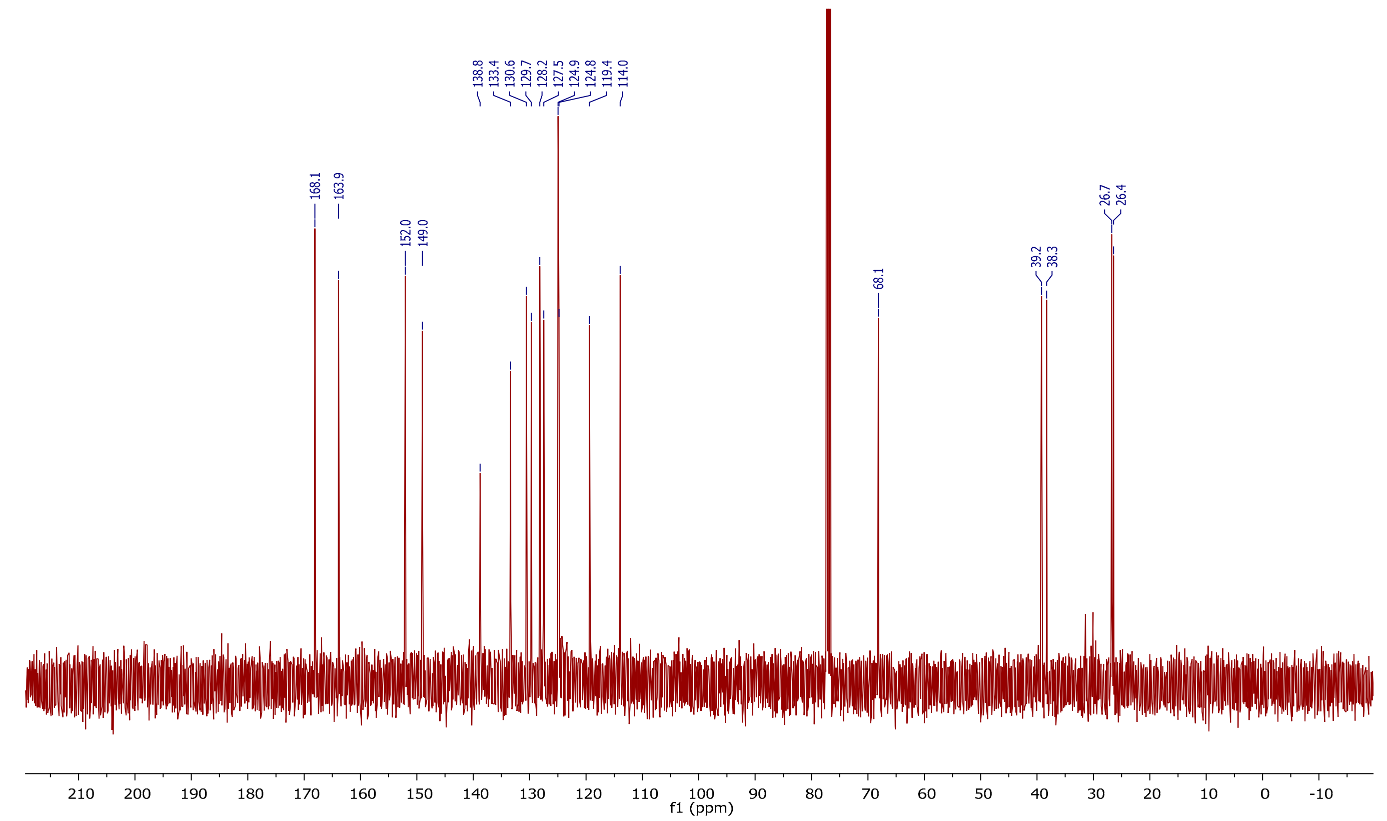

Fig. S23 ^{1}H NMR (400 MHz) and ^{13}C NMR (100 MHz) spectra of compound (*S*)-**10** in $CDCl_3$.

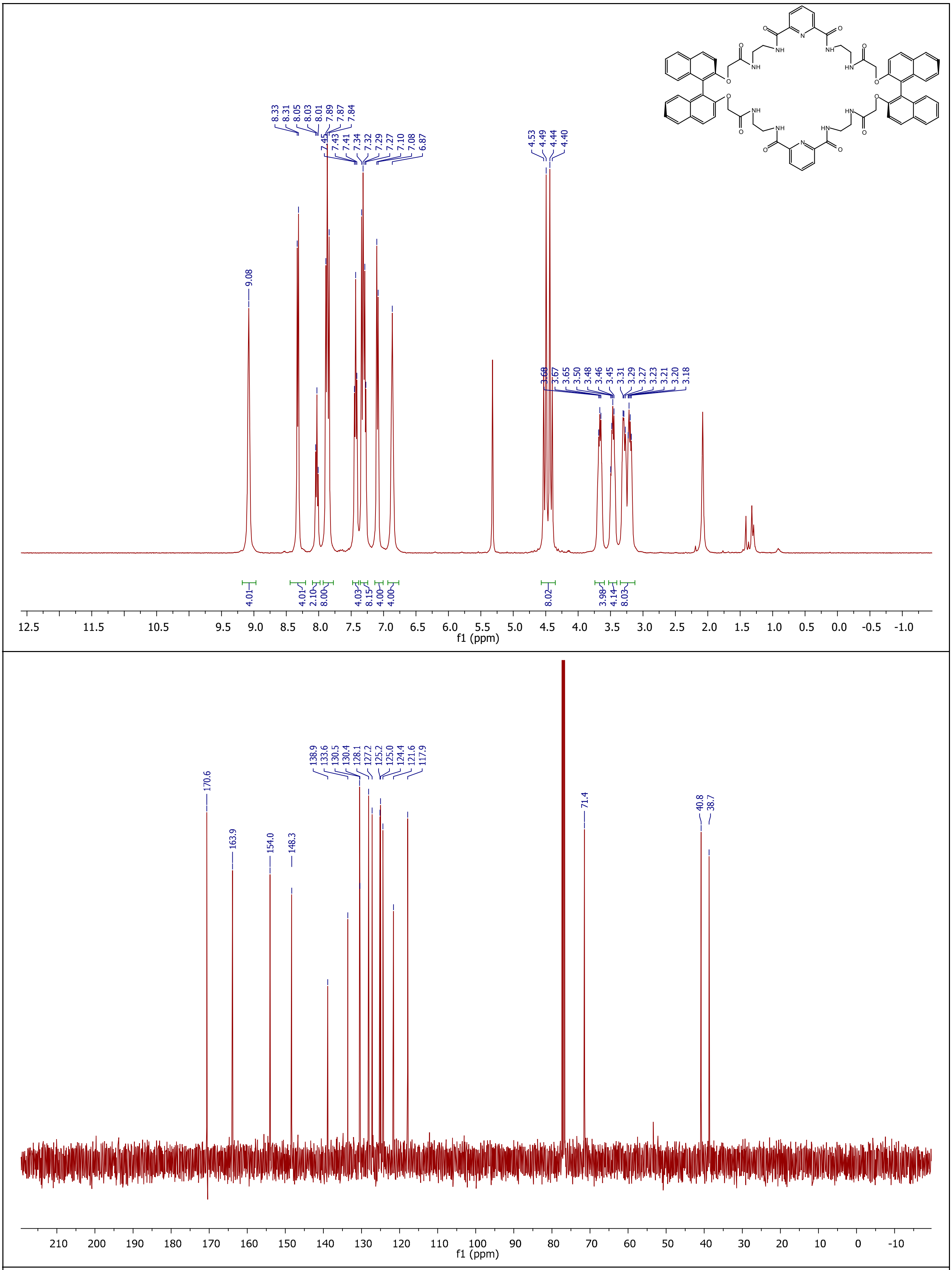

Fig. S24 ^{1}H NMR (400 MHz) and ^{13}C NMR (100 MHz) spectra of compound (*R*)-**8** in $CDCl_3$.

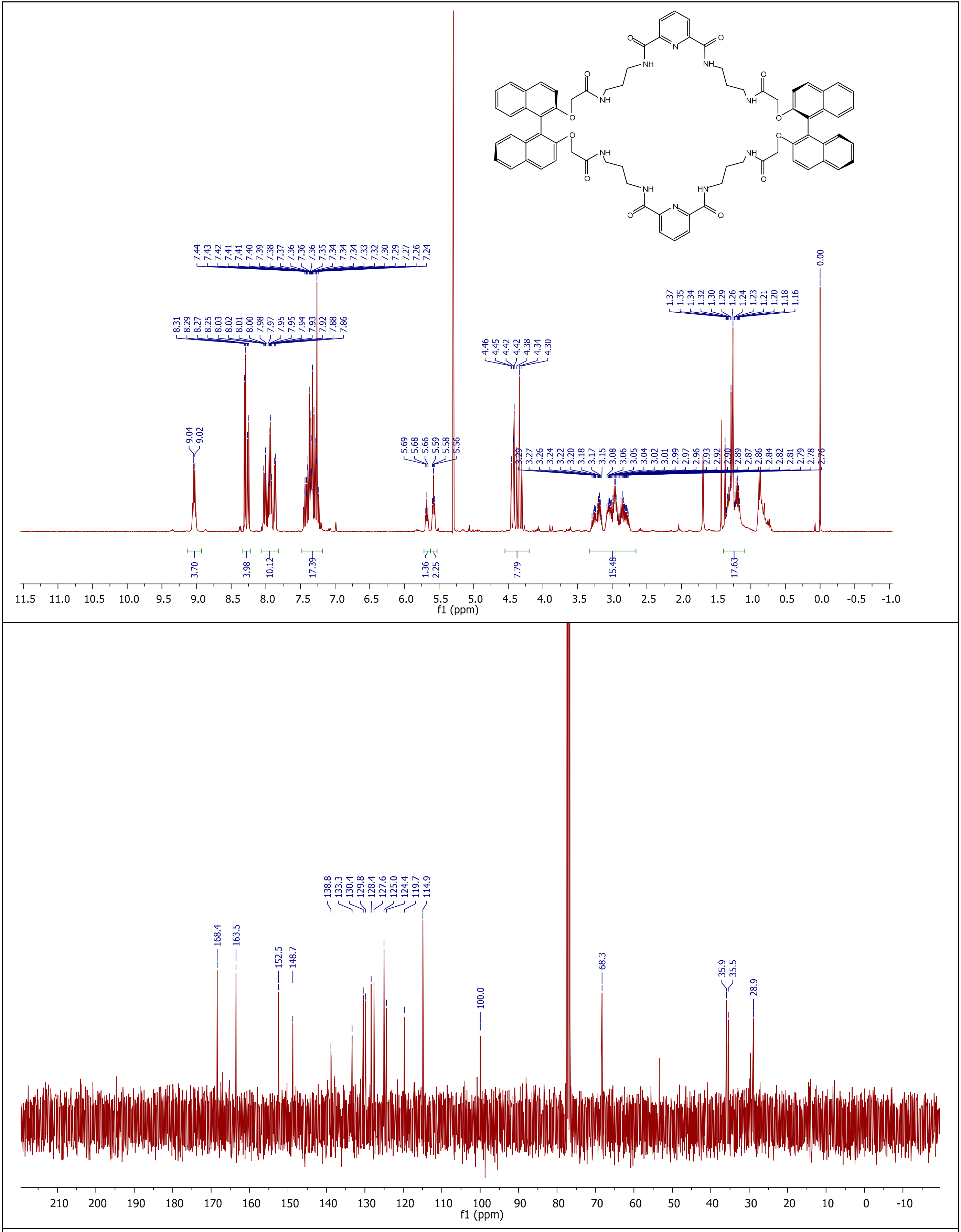

Fig. S25 ^{1}H NMR (400 MHz) and ^{13}C NMR (100 MHz) spectra of compound (*R*)-**9** in $CDCl_3$.

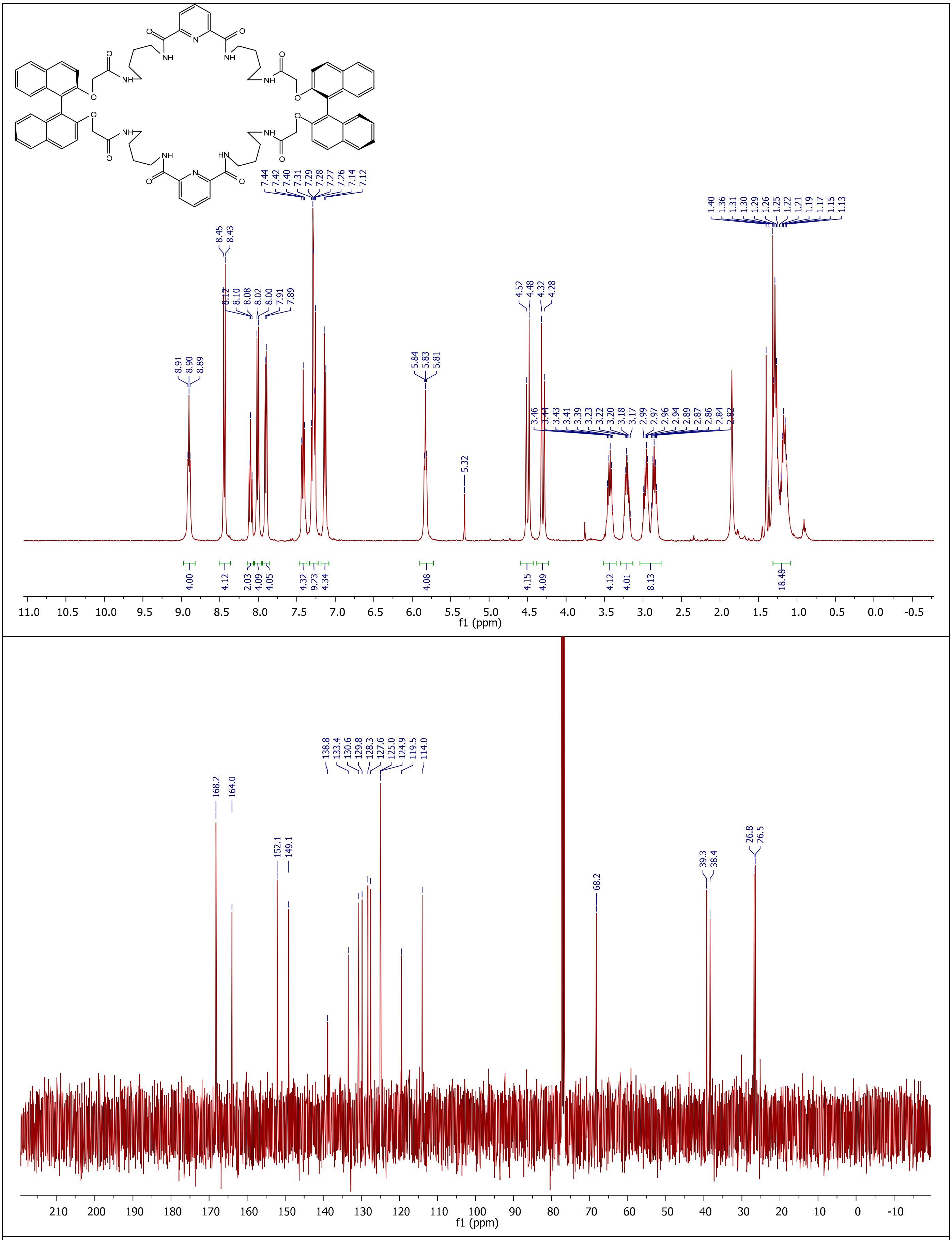

Fig. S26 ^{1}H NMR (400 MHz) and ^{13}C NMR (100 MHz) spectra of compound (*R*)-**10** in $CDCl_3$.

2. Binding Studies

2.1. General remarks

Tetrabutylammonium (TBA) salts of examinate anions were prepared before every titration experiments, namely commercially available carboxylic acids was dissolved in 0.5 mL of dry methanol and 1 equivalent of TBAOH (solution in methanol, c=1.21 M) was added. Prior to the experiment, the salts were pre-dried overnight under high vacuum at 60°C. To obtain the appropriate water concentration distilled water was added to the commercially available DMSO-d_6 or acetone-d_6 of 99.9% isotopic purity (purchased from ARMAR AG), and analyzed using Karl Fisher water titration. All titration experiments was performed on Bruker (400 MHz) at 298K.

The solution of a receptor (ca. 10^{-3} M) was titrated in NMR tube with the 0.1-0.3 M solution of a respective TBA salt. The solution of the salt contained a certain amount of the receptor to keep receptor concentration constant during titration experiments. It was important to choose such volumes of aliquots so that most of the data points could occur in close proximity of the inflection point of the respective titration curve; 11 to 23 data points were recorded. Such procedure allows for more precise calculation of binding constants. A nonlinear curve fitting for the 1:1 binding model was carried out with the HypNMR2008[1] Software (Version 4.0.71) and allows the determination of the global association constant. The details are given in ESI Figure S27 –S65 and Tables S1-S38. In all cases the most reliable results were obtained using a simple 1:1 (host:guest) binding model with excellent fit of the data points to the calculations points. Also analysis of residuals clearly confirmed the stoichiometry of the complexes.

---

[1] (a) Frassineti, C.; Ghelli, S.; Gans, P.; Sabatini, A.; Moruzzi, M.S.; Vacca A. Nuclear Magnetic Resonance as a Tool for Determining Protonation Constants of Natural Polyprotic Bases in Solution. *Analytical Biochemistry*, **1995**, *231*, 374-382. (b) Frassineti, C.; Alderighi, L.; Gans, P.; Sabatini, A.; Vacca A.; Ghelli, S. Determination of protonation constants of some fluorinated polyamines by means of ^{13}C NMR data processed by the new computer program HypNMR2000. Protonation sequence in polyamines. *Anal. Bioanal. Chem.*, **2003**, *376*,1041-1052. (c)Rodríguez-Barrientos, D.; Rojas-Hernández, A.; Gutiérrez, A.; Moya-Hernández, R.; Gómez-Balderas, R.; Ramírez-Silva, M.T. Determination of pKa Values of Tenoxicam from 1H NMR Chemical Shifts and of Oxicams from Electrophoretic Mobilities (CZE) with the Aid of Programs SQUAD and HYPNMR. *Talanta* **2009**, *80*, 754–762.

2.2. ^{1}H NMR titration experiments with achiral anions

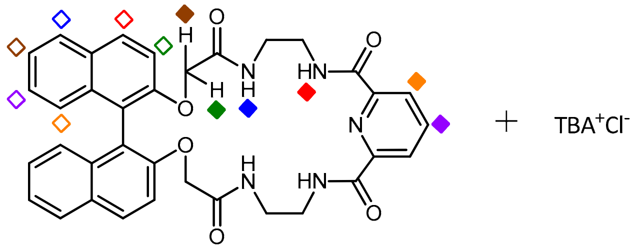

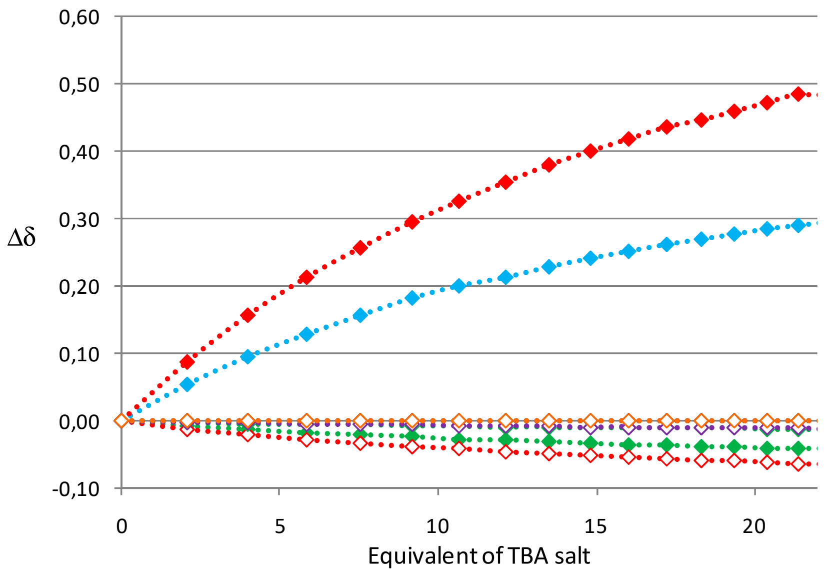

K=13 mol^{-1}

C_H=0,0042 mol/dm^3

C_G=0,2666 mol/dm^3

Range of chemical shift Δδ [ppm]

H◆ 0,31
H◆ 0,52
H◆ -0,045
H◆ no changes
H◆ no changes
H◆ no changes
H◇ no changes
H◇ -0,068
H◇ -0,01
H◇ no changes
H◇ -0,01
H◇ no changes

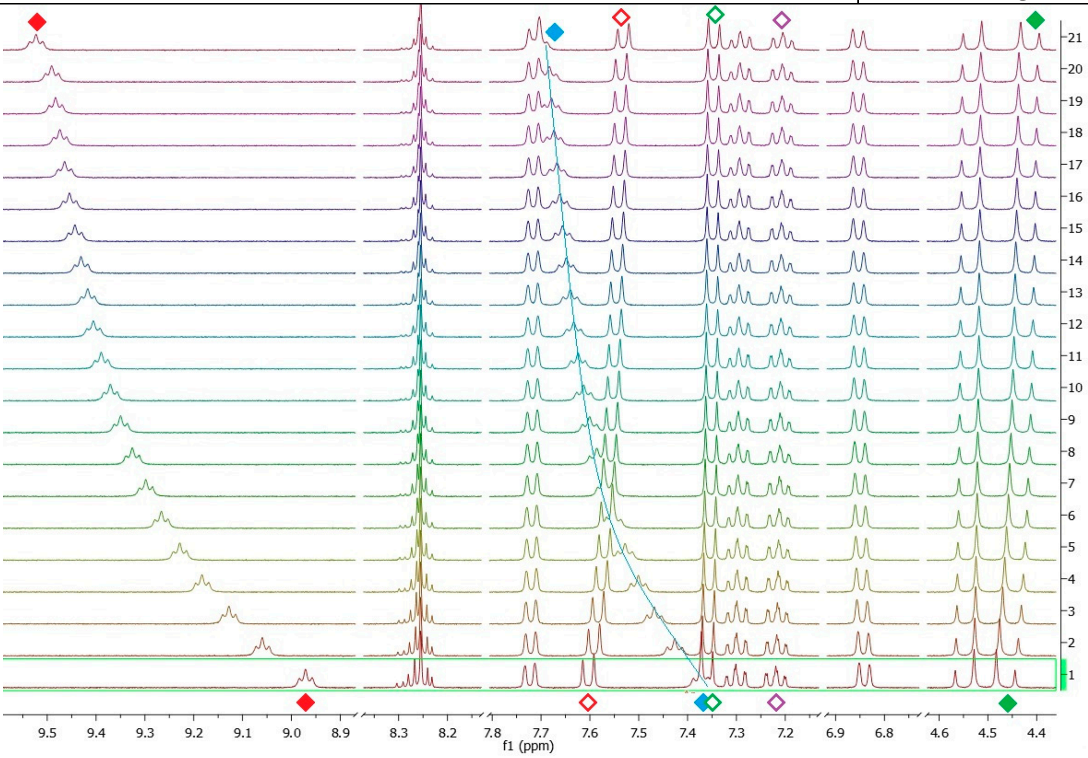

Fig. S27 The details of ^{1}H NMR titration of receptor ***(rac)*-1** with **Cl^-** in DMSO-d_6+0,5% water

Table S1. Experimental data used to determine binding constant of receptor ***(rac)*-1** with **Cl**$^-$ in DMSO-d$_6$+0,5% water

Point	Eq of guest	C_{host} [M]	C_{guest}[M]	Range of chemical shift Δδ [ppm]					
				H1◆	H2◆	H3◆	HAr1◇	HAr2◇	HAr3◇
1	0,00	0,0042	0,0000	0,0000	0,0000	0,0000	0,0000	0,0000	0,0000
2	2,07		0,0086	0,0540	0,0880	-0,0070	-0,0120	-0,0020	-0,0020
3	4,01		0,0167	0,0960	0,1570	-0,0130	-0,0200	-0,0040	-0,0030
4	5,83		0,0242	0,1290	0,2130	-0,0170	-0,0280	-0,0050	-0,0040
5	7,54		0,0314	0,1560	0,2570	-0,0210	-0,0340	-0,0060	-0,0050
6	9,16		0,0381	0,1820	0,2950	-0,0240	-0,0380	-0,0070	-0,0060
7	10,68		0,0444	0,2000	0,3270	-0,0270	-0,0420	-0,0080	-0,0070
8	12,13		0,0504	0,2130	0,3550	-0,0290	-0,0460	-0,0090	-0,0080
9	13,49		0,0561	0,2280	0,3790	-0,0320	-0,0490	-0,0090	-0,0080
10	14,79		0,0615	0,2400	0,4000	-0,0340	-0,0520	-0,0100	-0,0090
11	16,02		0,0666	0,2520	0,4180	-0,0360	-0,0540	-0,0100	-0,0090
12	17,20		0,0715	0,2610	0,4350	-0,0370	-0,0560	-0,0110	-0,0100
13	18,31		0,0762	0,2690	0,4460	-0,0380	-0,0580	-0,0110	-0,0100
14	19,38		0,0806	0,2760	0,4600	-0,0390	-0,0590	-0,0110	-0,0100
15	20,39		0,0848	0,2840	0,4730	-0,0410	-0,0610	-0,0120	-0,0110
16	21,36		0,0889	0,2900	0,4840	-0,0420	-0,0630	-0,0120	-0,0110
17	22,29		0,0927	0,2960	0,4850	-0,0420	-0,0640	-0,0130	-0,0120
18	23,18		0,0964	0,3020	0,5030	-0,0440	-0,0650	-0,0130	-0,0120
19	24,03		0,1000	0,3060	0,5120	-0,0450	-0,0660	-0,0130	-0,0120
20	24,85		0,1034	0,3110	0,5200	-0,0450	-0,0680	-0,0130	-0,0130

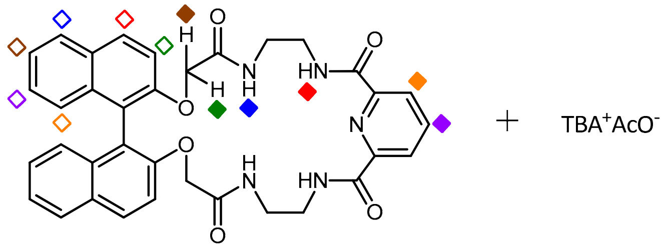

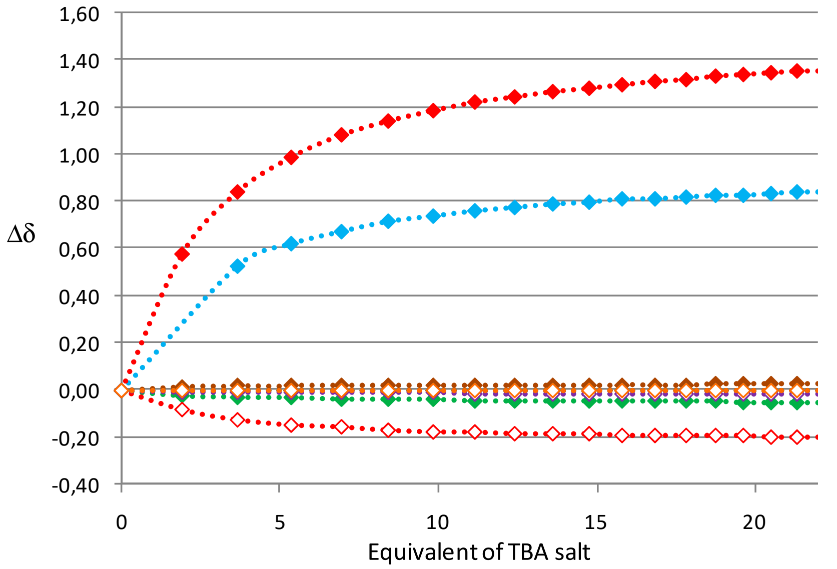

K=90 mol[-1]
C_H=0,0050 mol/dm^3
C_G=0,2680 mol/dm^3
Range of chemical shift δ [ppm]
H (blue filled diamond) 0,84
H (red filled diamond) 1,36
H (green filled diamond) -0,05
H (brown filled diamond) 0,025
H (violet filled diamond) no changes
H (orange filled diamond) no changes
H (blue open diamond) no changes
H (red open diamond) -0,20
H (green open diamond) no changes
H (brown open diamond) no changes
H (violet open diamond) -0,02
H (orange open diamond) no changes

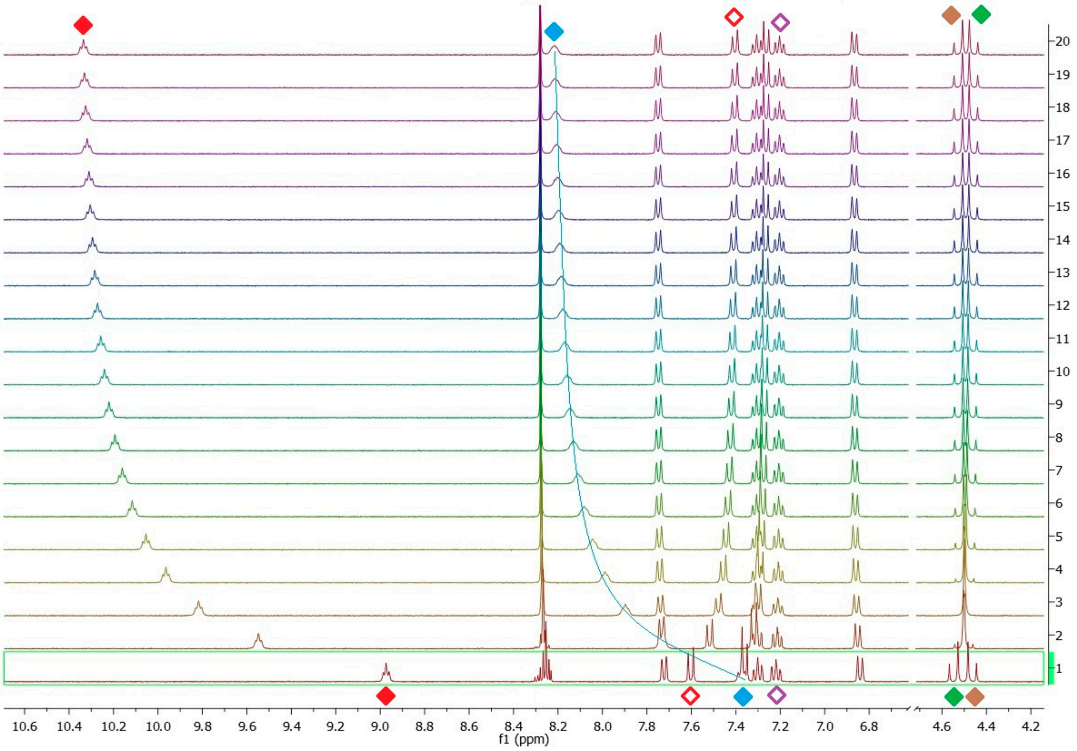

Fig. S29 The details of ^{1}H NMR titration of receptor ***(rac)*-1** with **AcO$^-$** in DMSO-d$_6$+0,5% water

**Table S2.** Experimental data used to determine binding constant of receptor **(*rac*)-1** with **AcO**$^-$ in DMSO-d_6+0,5% water										
Point	Eq of guest	C_{host} [M]	C_{guest}[M]	Range of chemical shift Δδ [ppm]						
				H1◆	H2◆	H3◆	HAr1◇	HAr2◇	HAr3◇
1	0,00	0,0050	0,0000	0,0000	0,0000	0,0000	0,0000	0,0000	0,0000
2	1,73		0,0086	x	0,5740	-0,0250	0,0160	-0,0850	-0,0060
3	3,35		0,0167	0,5270	0,8430	-0,0280	0,0170	-0,1240	-0,0080
4	4,87		0,0244	0,6180	0,9900	-0,0320	0,0170	-0,1460	-0,0100
5	6,31		0,0315	0,6740	1,0800	-0,0360	0,0190	-0,1590	-0,0110
6	7,66		0,0383	0,7130	1,1420	-0,0380	0,0200	-0,1680	-0,0120
7	8,93		0,0447	0,7400	1,1860	-0,0410	0,0210	-0,1740	-0,0120
8	10,14		0,0507	0,7610	1,2190	-0,0430	0,0210	-0,1790	-0,0130
9	11,29		0,0564	0,7760	1,2460	-0,0440	0,0230	-0,1830	-0,0130
10	12,37		0,0618	0,7880	1,2680	-0,0450	0,0230	-0,1860	-0,0130
11	13,40		0,0670	0,7990	1,2840	-0,0460	0,0230	-0,1880	-0,0140
12	14,38		0,0719	0,8090	1,2970	-0,0470	0,0240	-0,1900	-0,0140
13	15,32		0,0766	0,8140	1,3110	-0,0470	0,0240	-0,1920	-0,0140
14	16,21		0,0810	0,8220	1,3210	-0,0480	0,0240	-0,1930	-0,0150
15	17,06		0,0853	0,8270	1,3310	-0,0490	0,0250	-0,1940	-0,0150
16	17,87		0,0893	0,8290	1,3360	-0,0500	0,0250	-0,1940	-0,0150
17	18,65		0,0932	0,8360	1,3440	-0,0500	0,0250	-0,1970	-0,0150
18	19,39		0,0969	0,8390	1,3510	-0,0510	0,0250	-0,1980	-0,0150
19	20,10		0,1005	0,8420	1,3570	-0,0510	0,0250	-0,1980	-0,0150
20	20,79		0,1039	0,8430	1,3610	-0,0510	0,0250	-0,1990	-0,0160

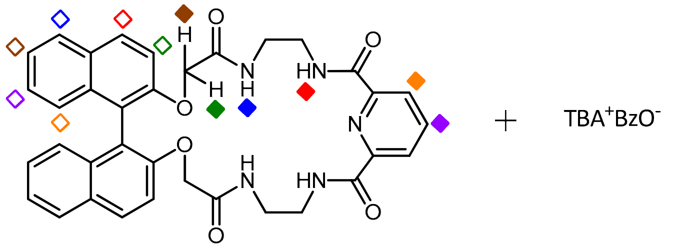

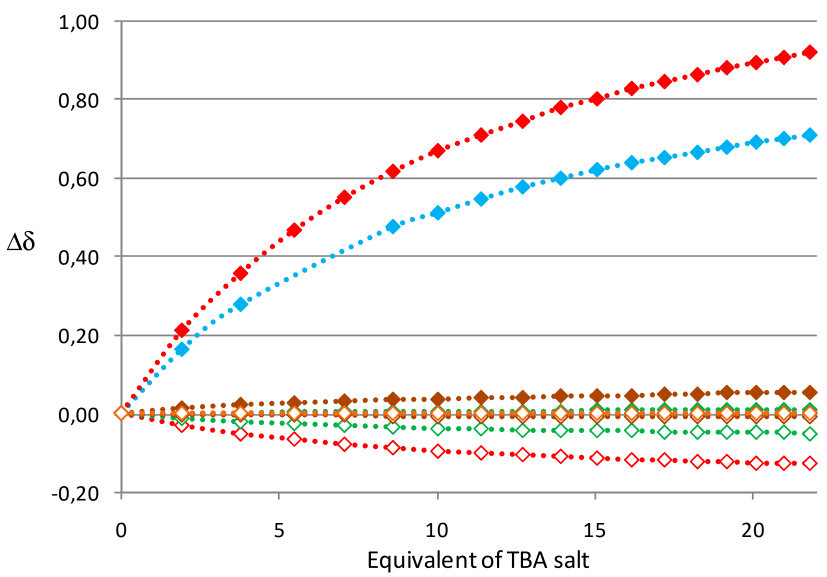

K=25 mol^{-1}
C_H=0,0042 mol/dm^3
C_G=0,2529 mol/dm^3
Range of chemical shift δ [ppm]
H◆ 0,72
H◆ 0,94
H◆ -0,01
H◆ 0,05
H◆ no changes
H◆ no changes
H◇ no changes
H◇ -0,13
H◇ -0,05
H◇ -0,01
H◇ covered
H◇ no changes

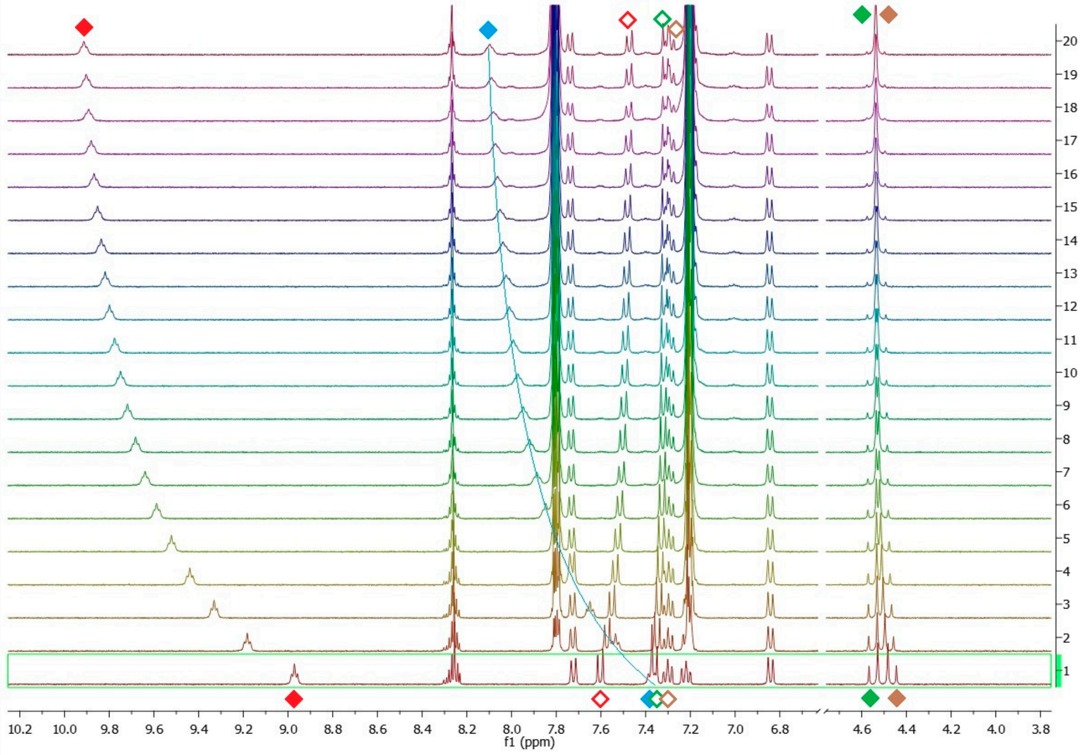

Fig. S30 The details of ^{1}H NMR titration of receptor ***(rac)*-1** with **BzO$^-$** in DMSO-d_6+0,5% water

**Table S3.** Experimental data used to determine binding constant of receptor **(*rac*)-1** with **BzO**$^-$ in DMSO-d_6+0,5% water									
Point	Eq of guest	C_{host} [M]	C_{guest}[M]	Range of chemical shift Δδ [ppm]					
				H1◆	H2◆	H3◆	HAr1◇	HAr2◇	HAr3◇
1	0,00	0,0042	0,0000	0,0000	0,0000	0,0000	0,0000	0,0000	0,0000
2	1,94		0,0082	0,1630	0,2110	0,0020	0,0130	-0,0300	-0,0120
3	3,76		0,0158	0,2780	0,3590	0,0040	0,0220	-0,0510	-0,0200
4	5,48		0,0230	x	0,4680	0,0050	0,0280	-0,0660	-0,0260
5	7,09		0,0298	x	0,5500	0,0060	0,0320	-0,0780	-0,0300
6	8,60		0,0361	0,4750	0,6160	0,0060	0,0370	-0,0870	-0,0340
7	10,04		0,0422	0,5140	0,6680	0,0070	0,0390	-0,0940	-0,0370
8	11,39		0,0479	0,5470	0,7100	0,0070	0,0410	-0,1000	-0,0390
9	12,68		0,0532	0,5760	0,7460	0,0080	0,0430	-0,1050	-0,0400
10	13,90		0,0584	0,6000	0,7780	0,0080	0,0440	-0,1090	-0,0420
11	15,06		0,0632	0,6200	0,8040	0,0100	0,0460	-0,1120	-0,0430
12	16,16		0,0679	0,6380	0,8270	0,0090	0,0470	-0,1150	-0,0440
13	17,21		0,0723	0,6530	0,8460	0,0090	0,0480	-0,1180	-0,0450
14	18,21		0,0765	0,6670	0,8640	0,0090	0,0490	-0,1200	-0,0460
15	19,16		0,0805	0,6800	0,8800	0,0090	0,0540	-0,1220	-0,0470
16	20,08		0,0843	0,6910	0,8960	0,0090	0,0540	-0,1250	-0,0470
17	20,95		0,0880	0,6990	0,9080	0,0090	0,0540	-0,1260	-0,0480
18	21,79		0,0915	0,7100	0,9200	0,0090	0,0540	-0,1270	-0,0490
19	22,59		0,0948	0,7190	0,9310	0,0090	0,0540	-0,1280	-0,0490
20	23,35		0,0981	0,7240	0,9420	0,0090	0,0540	-0,1300	-0,0490

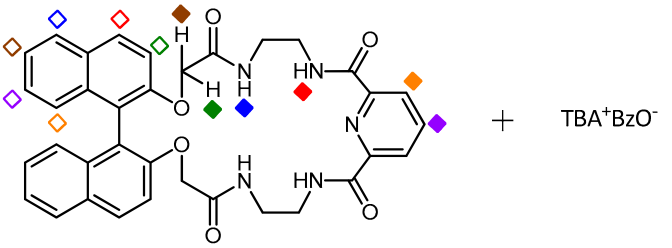

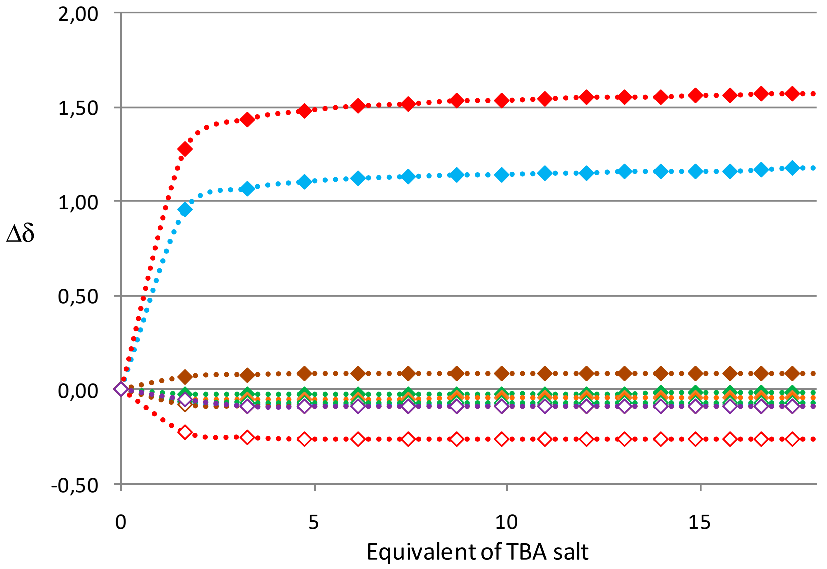

K=916 mol^{-1}
C_H=0,0051 mol/dm^3
C_G=0,2659 mol/dm^3
Range of chemical shift δ [ppm]
H◆ 1,17
H◆ 1,57
H◆ -0,02
H◆ 0,08
H◆ no changes
H◆ -0,04
H◇ -0,07
H◇ -0,26
H◇ -0,07
H◇ -0,08
H◇ -0,09
H◇ covered

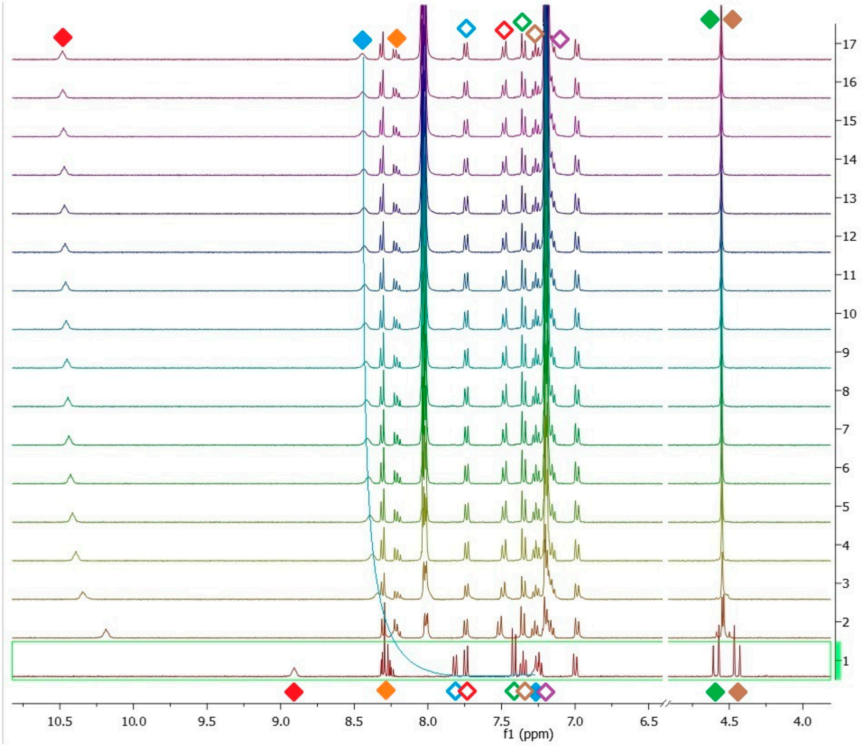

Fig. S31 The details of ^{1}H NMR titration of receptor **(*rac*)-1** with **BzO**$^-$ in acetone-d$_6$+0,5% water

**Table S4.** Experimental data used to determine binding constant of receptor **(*rac*)-1** with **BzO$^-$** in acetone-d$_6$+0,5%													
Point	Eq of guest	C_{host} [M]	C_{guest} [M]	Range of chemical shift Δδ [ppm]									
				H1◆	H2◆	H3◆	HAr1◆	HAr2◆	HAr3◇	HAr4◇	HAr5◇	HAr6◇	HAr4◇
1	0,00	0,0051	0,0000	0,0000	0,0000	0,0000	0,0000	0,0000	0,0000	0,0000	0,0000	0,0000	0,0000
2	1,73		0,0086	0,9530	1,2800	- 0,0230	0,0710	-0,0470	-0,0730	-0,2280	-0,0600	-0,0800	-0,0550
3	3,35		0,0166	1,0700	1,4370	- 0,0240	0,0810	-0,0480	-0,0790	-0,2520	-0,0650	-0,0850	-0,0870
4	4,88		0,0242	1,1060	1,4830	- 0,0220	0,0830	-0,0480	-0,0800	-0,2580	-0,0660	-0,0870	-0,0890
5	6,31		0,0313	1,1210	1,5060	- 0,0210	0,0840	-0,0480	-0,0790	-0,2600	-0,0660	-0,0870	-0,0890
6	7,67		0,0380	1,1300	1,5200	- 0,0200	0,0850	-0,0470	-0,0790	-0,2610	-0,0670	-0,0870	-0,0890
7	8,95		0,0443	1,1380	1,5310	- 0,0200	0,0850	-0,0460	-0,0780	-0,2620	-0,0670	-0,0870	-0,0890
8	10,15		0,0503	1,1440	1,5370	- 0,0190	0,0860	-0,0450	-0,0770	-0,2620	-0,0670	-0,0860	-0,0890
9	11,30		0,0560	1,1460	1,5440	- 0,0190	0,0860	-0,0450	-0,0770	-0,2620	-0,0660	-0,0860	-0,0890
10	12,39		0,0614	1,1510	1,5490	- 0,0190	0,0860	-0,0440	-0,0760	-0,2620	-0,0660	-0,0860	-0,0890
11	13,42		0,0665	1,1570	1,5520	- 0,0190	0,0860	-0,0430	-0,0700	-0,2620	-0,0660	-0,0860	-0,0890
12	14,40		0,0713	1,1570	1,5560	- 0,0180	0,0870	-0,0430	-0,0750	-0,2620	-0,0660	-0,0850	-0,0890
13	15,34		0,0760	1,1610	1,5600	- 0,0180	0,0870	-0,0420	-0,0750	-0,2620	-0,0660	-0,0850	-0,0890
14	16,23		0,0804	1,1620	1,5620	- 0,0180	0,0870	-0,0410	-0,0750	-0,2610	-0,0660	-0,0850	-0,0890
15	17,89		0,0886	1,1660	1,5670	- 0,0180	0,0870	-0,0400	-0,0740	-0,2600	-0,0650	-0,0850	-0,0890
16	19,41		0,0962	1,1730	1,5720	- 0,0170	0,0880	-0,0390	-0,0730	-0,2600	-0,0650	-0,0840	-0,0890
17	20,81		0,1031	1,1730	1,5740	- 0,0170	0,0880	-0,0380	-0,0730	-0,2600	-0,0650	-0,0840	-0,0890

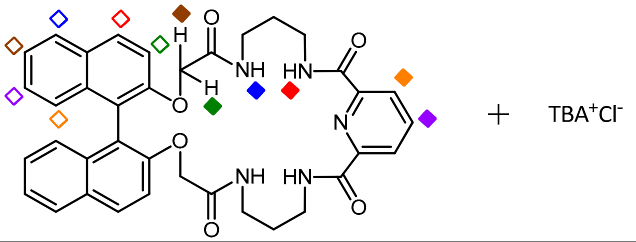

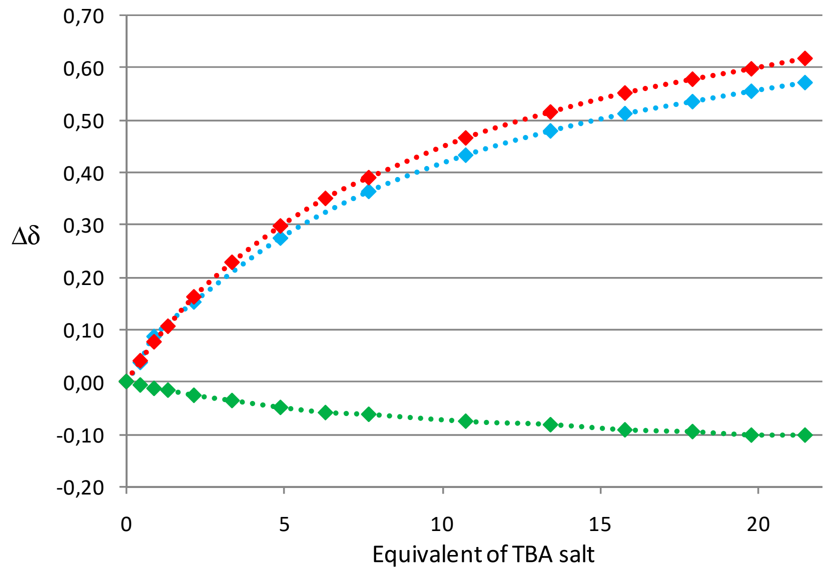

K=35 mol^{-1}
C_H=0,0049 mol/dm^3
C_G=0,1835 mol/dm^3
Range of chemical shift δ [ppm]
H◆ 0,57
H◆ 0,62
H◆ -0,10
H◆ no changes
H◆ no changes
H◆ no changes
H◇ no changes
H◇ no changes
H◇ no changes
H◇ no changes
H◇ no changes
H◇ no changes

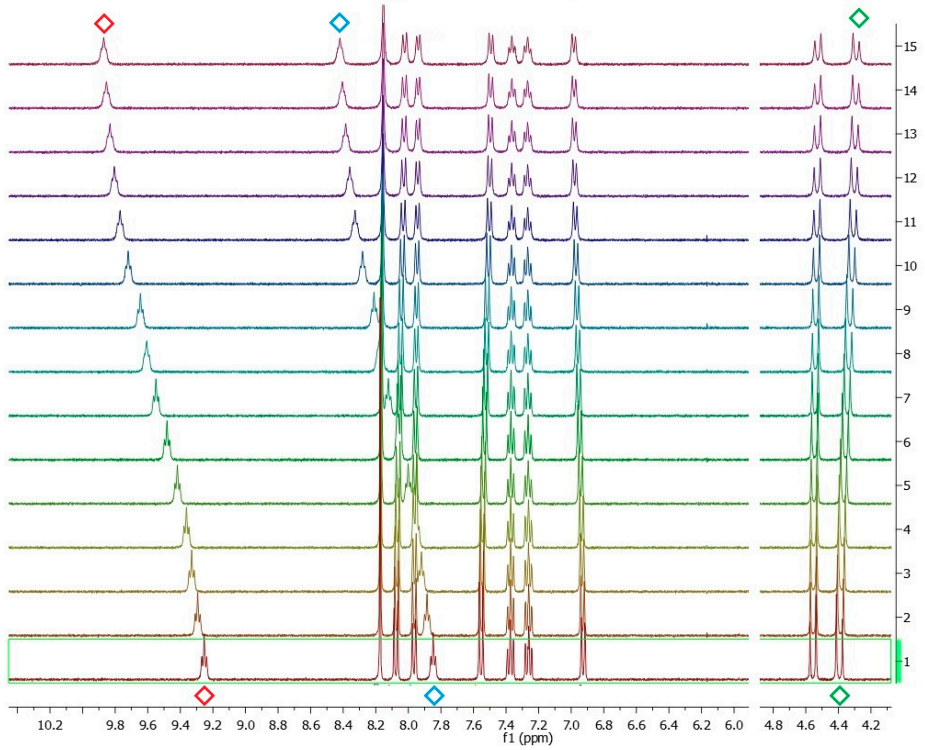

Fig. S32 The details of ^{1}H NMR titration of receptor ***(rac)*-2** with **Cl^-** in DMSO-d_6+0,5% water

**Table S5.** Experimental data used to determine binding constant of receptor ***(rac)*-2** with **Cl^-** in DMSO-d_6+0,5% water						
Point	Eq of guest	C_{host} [M]	C_{guest}[M]	Range of chemical shift Δδ [ppm]		
				H1◆	H2◆	H3◆
1	0,00	0,0049	0,0000	0,0000	0,0000	0,0000
2	0,40		0,0015	0,0380	0,0390	-0,0060
3	0,79		0,0030	0,0860	0,0760	-0,0120
4	1,17		0,0045	x	0,1080	-0,0170
5	1,92		0,0073	0,1530	0,1640	-0,0260
6	3,00		0,0115	x	0,2270	-0,0360
7	4,37		0,0167	0,2750	0,2960	-0,0470
8	5,66		0,0216	x	0,3520	-0,0570
9	6,87		0,0262	0,3620	0,3910	-0,0630
10	9,62		0,0367	0,4330	0,4670	-0,0760
11	12,02		0,0459	0,4790	0,5150	-0,0800
12	14,14		0,0540	0,5120	0,5520	-0,0910
13	16,03		0,0612	0,5360	0,5770	-0,0960
14	17,71		0,0676	0,5560	0,5990	-0,1000
15	19,23		0,0734	0,5720	0,6170	-0,1020

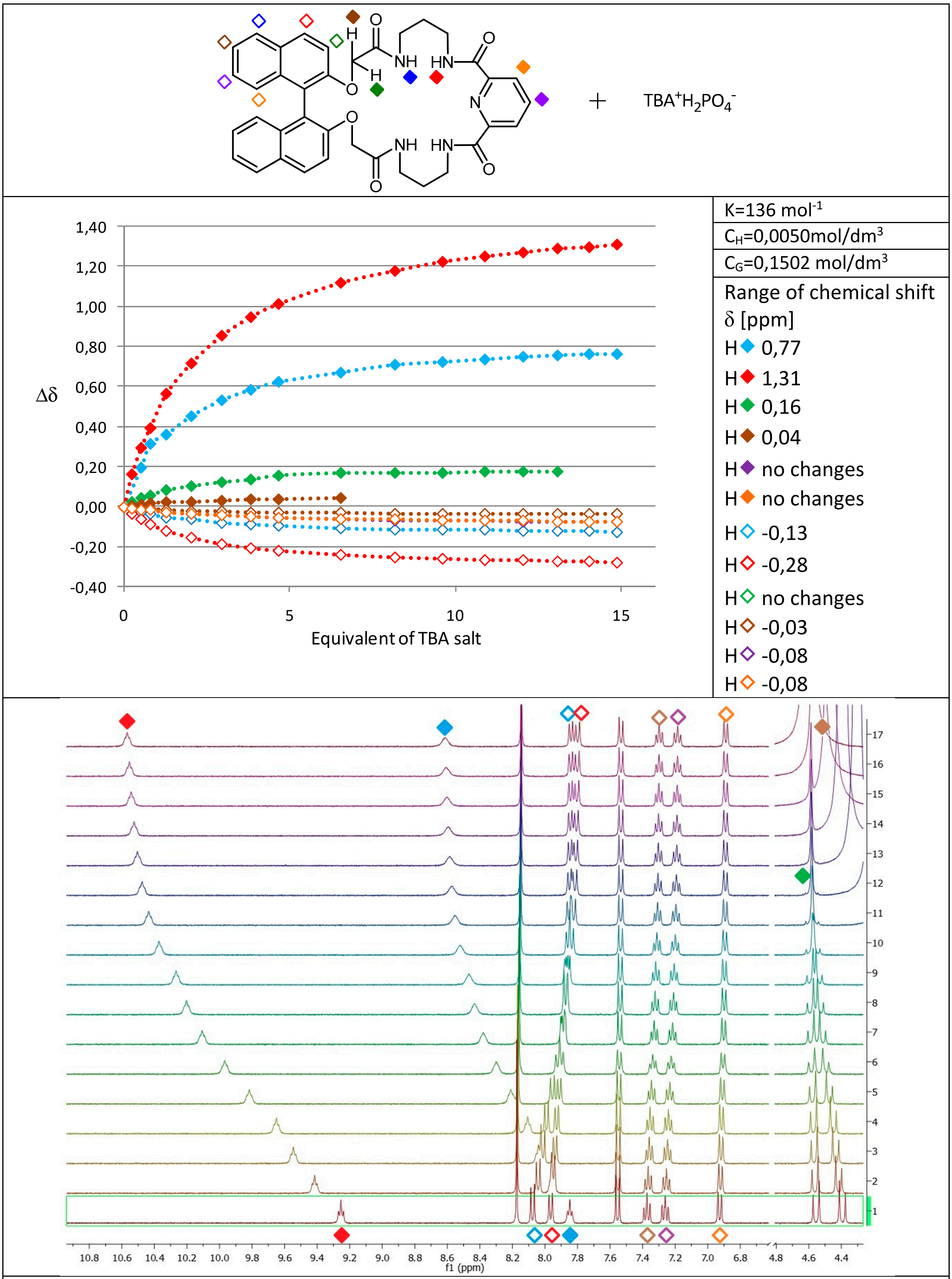

Fig. S33 The details of ^{1}H NMR titration of receptor **(*rac*)-2** with **$H_2PO_4^-$** in DMSO-d_6+0,5% water

**Table S6.** Experimental data used to determine binding constant of receptor **(*rac*)-2** with $\mathbf{H_2PO_4^-}$ in DMSO-d_6+0,5% water													
Point	Eq of guest	C_{host} [M]	C_{guest} [M]	Range of chemical shift Δδ [ppm]									
				H1◆	H2◆	H3◆	H4◆	HAr1◇	HAr2◇	HAr3◇	HAr4◇	HAr5◇
1	0,00	0,0050	0,0000	0,0000	0,0000	0,0000	0,0000	0,0000	0,0000	0,0000	0,0000	0,0000
2	0,27		0,0013	x	0,1610	0,0220	0,0060	x	-0,0350	-0,0050	-0,0070	-0,0070
3	0,54		0,0027	0,1930	0,2920	0,0410	0,0110	-0,0270	-0,0630	-0,0080	-0,0140	-0,0140
4	0,80		0,0040	x	0,3960	0,0560	0,0150	-0,0370	-0,0860	-0,0110	-0,0190	-0,0190
5	1,31		0,0065	0,3630	0,5630	0,0800	0,0210	-0,0530	-0,1220	-0,0160	-0,0280	-0,0280
6	2,04		0,0101	0,4540	0,7140	0,1020	0,0270	-0,0650	-0,1550	-0,0200	-0,0360	-0,0350
7	2,97		0,0148	0,5320	0,8530	0,1210	0,0310	-0,0800	-0,1850	-0,0250	-0,0450	-0,0440
8	3,85		0,0191	0,5860	0,9490	0,1350	0,0350	-0,0910	-0,2040	-0,0280	-0,0510	-0,0500
9	4,67		0,0232	0,6210	1,0130	0,1580	0,0400	-0,0960	-0,2180	-0,0290	-0,0550	-0,0540
10	6,54		0,0325	0,6730	1,1190	0,1670	0,0420	-0,1070	-0,2400	-0,0320	-0,0610	-0,0610
11	8,18		0,0406	0,7070	1,1810	0,1700	x	-0,1120	-0,2530	-0,0330	-0,0660	-0,0650
12	9,62		0,0478	0,7250	1,2220	0,1720	x	-0,1160	-0,2600	-0,0350	-0,0690	-0,0680
13	10,90		0,0541	0,7370	1,2500	0,1740	x	-0,1180	-0,2660	-0,0350	-0,0710	-0,0700
14	12,05		0,0598	0,7460	1,2720	0,1750	x	-0,1200	-0,2690	-0,0360	-0,0720	-0,0710
15	13,08		0,0650	0,7570	1,2900	0,1750	x	-0,1220	-0,2720	-0,0370	-0,0740	-0,0730
16	14,01		0,0696	0,7610	1,3000	x	x	-0,1230	-0,2750	-0,0370	-0,0750	-0,0740
17	14,86		0,0733	0,7650	1,3130	x	x	-0,1250	-0,2770	-0,0370	-0,0760	-0,0750

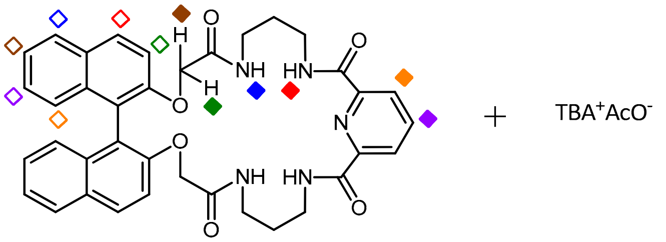

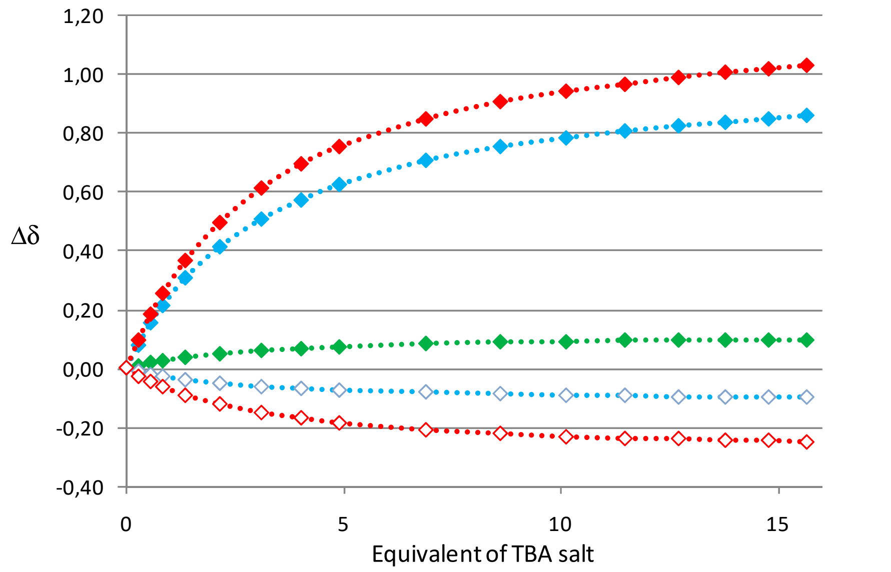

K=82 mol $^{-1}$
C_H= 0,0049 mol/dm^3
C_G=0,1694 mol/dm^3
Range of chemical shift δ [ppm]
H ◆ 0,86
H ◆ 1,03
H ◆ 0,10
H ◆ no changes
H ◆ no changes
H ◆ no changes
H ◇ -0,10
H ◇ -0,25
H ◇ no changes
H ◇ no changes
H ◇ no changes
H ◇ no changes

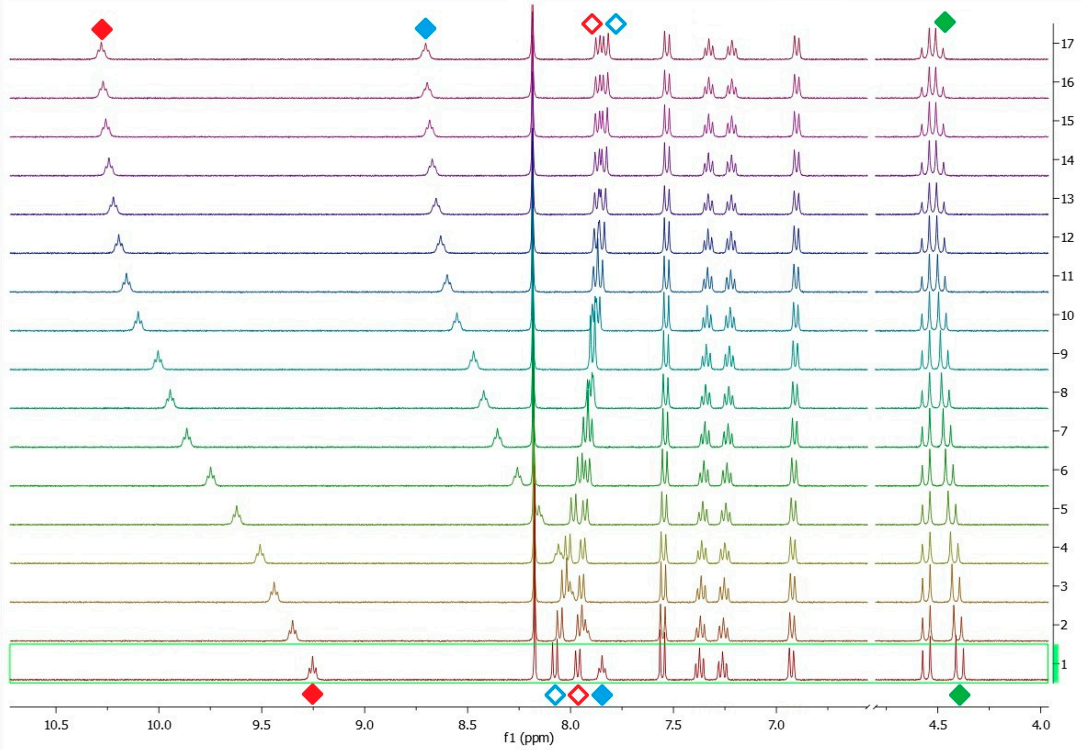

Fig. S34 The details of ^{1}H NMR titration of receptor ***(rac)*-2** with **AcO$^-$** in DMSO-d_6+0,5% water

Table S7. Experimental data used to determine binding constant of receptor **(*rac*)-2** with **AcO**$^-$ in DMSO-d_6+0,5% water

Point	Eq of guest	C_{host} [M]	C_{guest}[M]	Range of chemical shift Δδ [ppm]				
				H1◆	H2◆	H3◆	HAr1◇	HAr2◇
1	0,00	0,0049	0,0000	0,0000	0,0000	0,0000	0,0000	0,0000
2	0,28		0,0014	0,0820	0,0970	0,0100	-0,0100	-0,0240
3	0,56		0,0028	0,1560	0,1870	0,0190	-0,0180	-0,0460
4	0,84		0,0041	0,2120	0,2550	0,0260	-0,0250	-0,0630
5	1,38		0,0068	0,3060	0,3690	0,0380	-0,0360	-0,0910
6	2,15		0,0106	0,4110	0,4960	0,0510	-0,0470	-0,1210
7	3,13		0,0154	0,5090	0,6110	0,0620	-0,0600	-0,1500
8	4,05		0,0199	0,5740	0,6920	0,0700	-0,0660	-0,1700
9	4,92		0,0242	0,6240	0,7510	0,0750	-0,0720	-0,1830
10	6,89		0,0339	0,7050	0,8470	0,0840	-0,0810	-0,2070
11	8,61		0,0424	0,7520	0,9040	0,0900	-0,0870	-0,2210
12	10,13		0,0498	0,7830	0,9420	0,0930	-0,0900	-0,2290
13	11,48		0,0565	0,8060	0,9670	0,0940	-0,0920	-0,2350
14	12,69		0,0624	0,8250	0,9890	0,0960	-0,0940	-0,2400
15	13,78		0,0678	0,8370	1,0050	0,0970	-0,0960	-0,2440
16	14,77		0,0726	0,8500	1,0190	0,0980	-0,0970	-0,2460
17	15,66		0,0770	0,8560	1,0270	0,0990	-0,0980	-0,2480

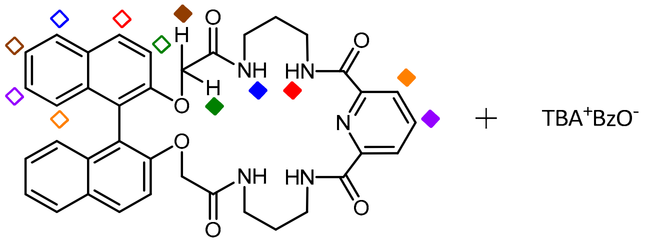

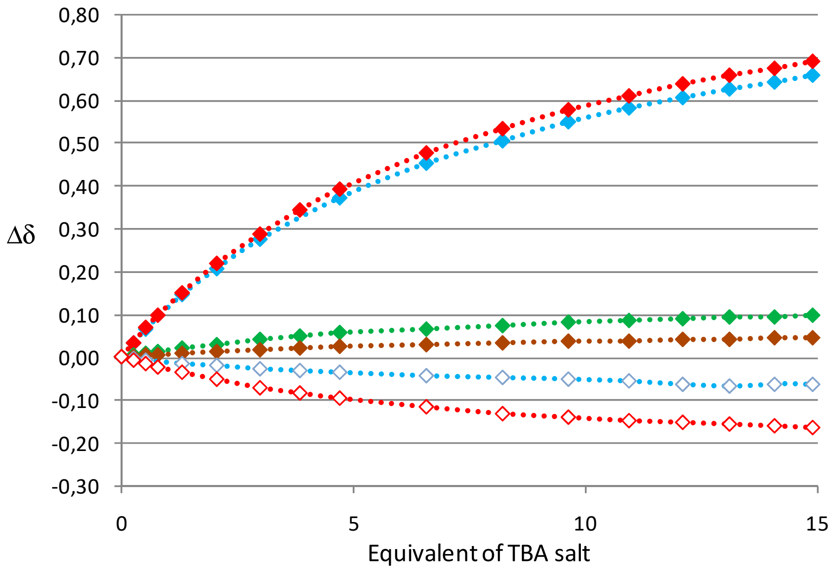

K=29 mol^{-1}
C_H=0,0050 mol/dm^3
C_G=0,048 mol/dm^3
Range of chemical shift δ [ppm]
H◆ 0,63
H◆ 0,69
H◆ 0,10
H◆ 0,04
H◆ no changes
H◆ no changes
H◇ -0,06
H◇ -0,17
H◇ no changes
H◇ no changes
H◇ no changes
H◇ no changes

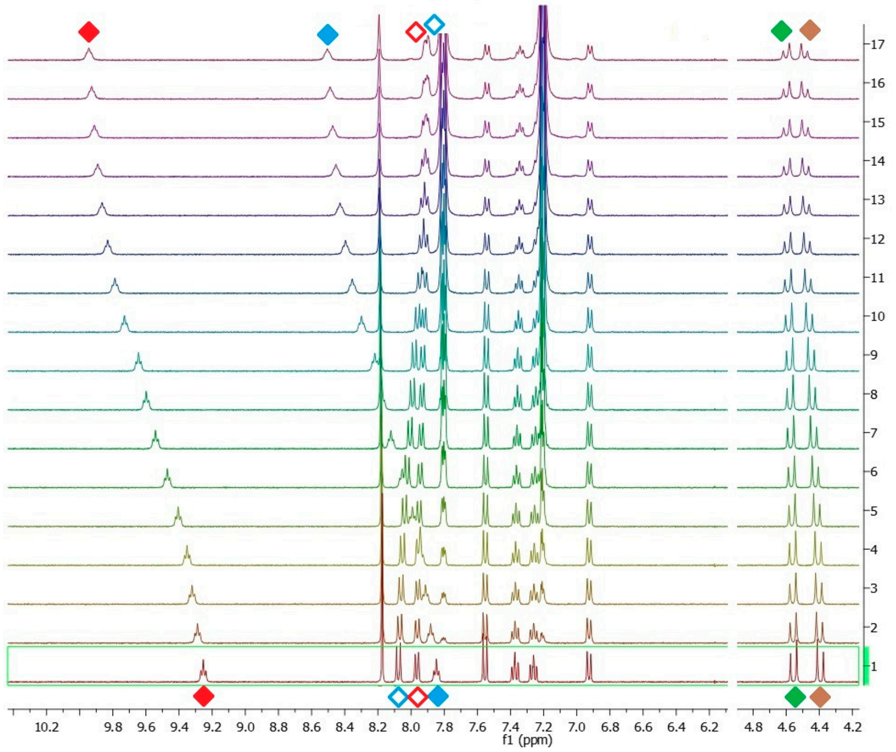

Fig. S35 The details of ^{1}H NMR titration of receptor ***(rac)*-2** with **BzO$^-$** in DMSO-d$_6$+0,5% water

**Table S8.** Experimental data used to determine binding constant of receptor **(*rac*)-2** with **BzO⁻** in DMSO-d_6+0,5% water									
Point	Eq of guest	C_{host} [M]	C_{guest}[M]	Range of chemical shift Δδ [ppm]					
				H1◆	H2◆	H3◆	H4◆	HAr1◇	HAr2◇
1	0,00	0,0050	0,0000	0,0000	0,0000	0,0000	0,0000	0,0000	0,0000
2	0,27		0,0013	0,0350	0,0350	-0,0030	-0,0080	0,0056	0,0031
3	0,54		0,0027	0,0670	0,0680	-0,0050	-0,0160	0,0103	0,0045
4	0,80		0,0040	x	0,0980	x	-0,0240	0,0143	0,0063
5	1,31		0,0065	0,1450	0,1520	-0,0140	-0,0370	0,0220	0,0095
6	2,05		0,0102	0,2070	0,2170	-0,0200	-0,0530	0,0314	0,0135
7	2,98		0,0148	0,2740	0,2880	-0,0260	-0,0700	0,0415	0,0181
8	3,86		0,0191	x	0,3440	-0,0320	-0,0840	0,0494	0,0215
9	4,68		0,0233	0,3720	0,3910	-0,0360	-0,0960	0,0558	0,0242
10	6,56		0,0326	0,4520	0,4750	-0,0440	-0,1160	0,0676	0,0298
11	8,19		0,0407	0,5070	0,5340	-0,0490	-0,1300	0,0757	0,0338
12	9,64		0,0479	0,5490	0,5760	-0,0510	-0,1410	0,0817	0,0363
13	10,93		0,0543	0,5820	0,6110	-0,0570	-0,1470	0,0870	0,0390
14	12,08		0,0600	0,6060	0,6360	-0,0620	-0,1520	0,0901	0,0404
15	13,11		0,0651	0,6250	0,6570	-0,0670	-0,1570	0,0927	0,0421
16	14,05		0,0698	0,6410	0,6730	-0,0620	-0,1620	0,0951	0,0436
17	14,90		0,0740	0,6580	0,6900	-0,0620	-0,1660	0,0974	0,0442

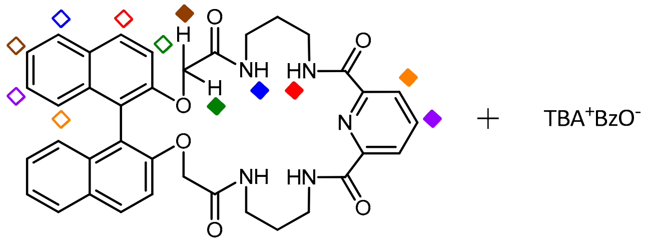

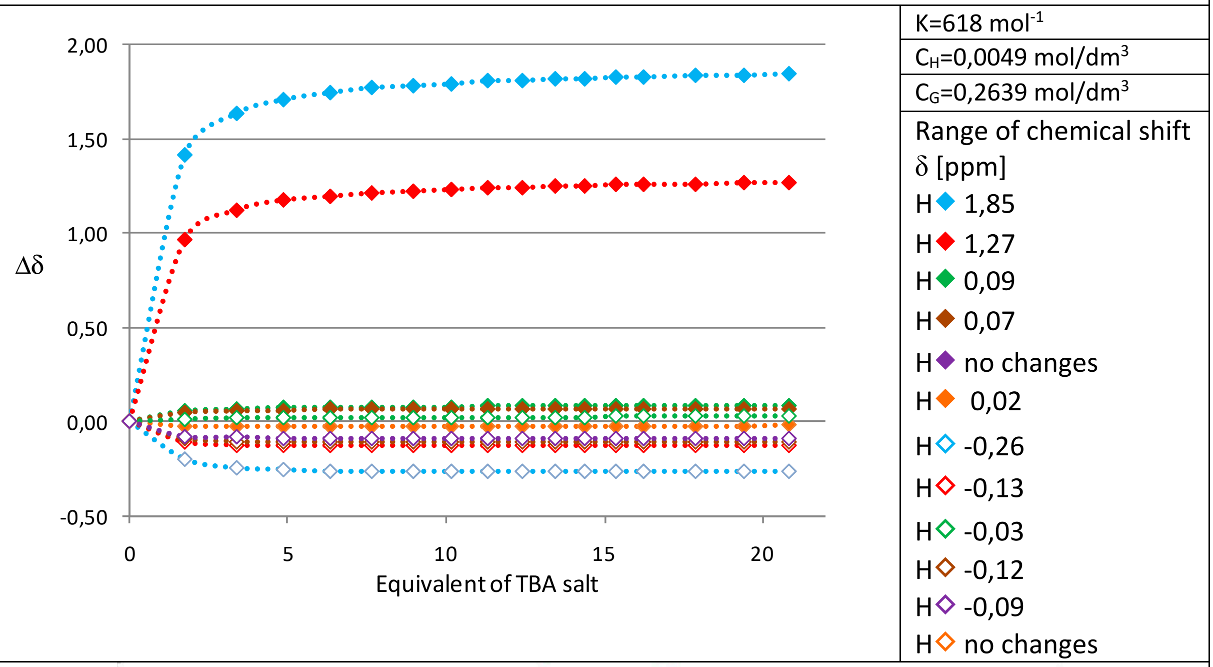

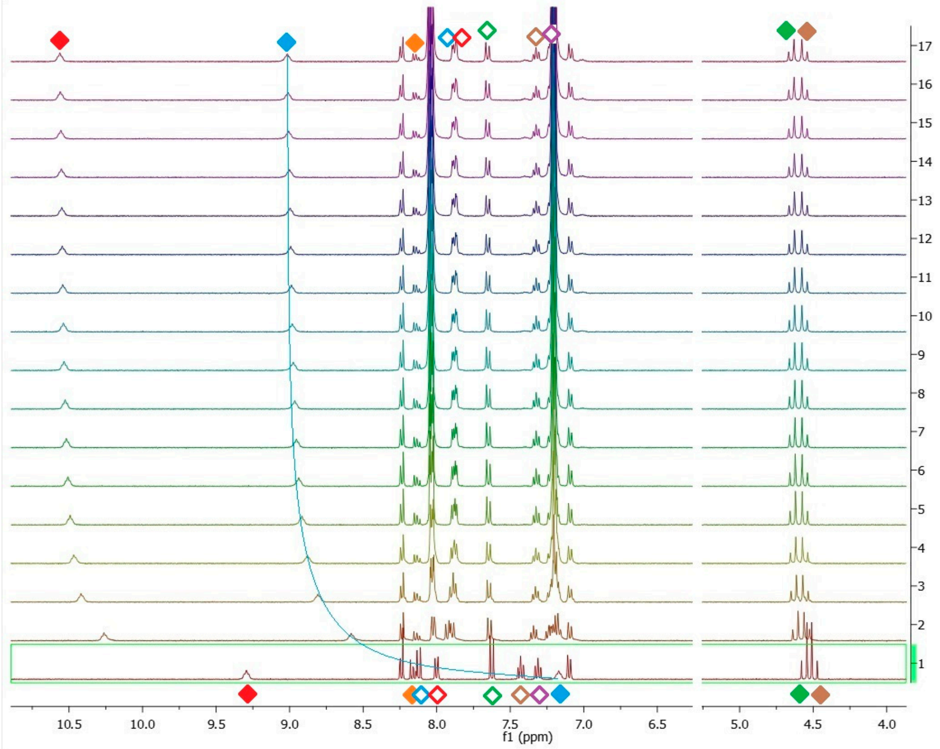

Fig. S36 The details of ^{1}H NMR titration of receptor **(*rac*)-2** with **BzO^{-}** in acetone-d_6+0,5% water

Table S9. Experimental data used to determine binding constant of receptor ***(rac)*-2** with **BzO**$^-$ in acetone-d_6+0,5%

Point	Eq of guest	C_{host} [M]	C_{guest} [M]	Range of chemical shift Δδ [ppm]									
				H1 ◆	H2 ◆	H3 ◆	H4 ◆	HAr1 ◆	HAr2 ◇	HAr3 ◇	HAr4 ◇	HAr5 ◇	HAr6 ◇
1	0,00	0,0049	0,0000	0,0000	0,0000	0,0000	0,0000	0,0000	0,0000	0,0000	0,0000	0,0000	0,0000
2	1,73		0,0085	1,4110	0,9680	0,0610	0,0520	-0,0230	-0,1940	-0,1060	0,0170	-0,0890	-0,0740
3	3,35		0,0165	1,6390	1,1250	0,0720	0,0610	-0,0260	-0,2450	-0,1230	0,0200	-0,1020	-0,0760
4	4,88		0,0240	1,7120	1,1740	0,0760	0,0640	-0,0250	-0,2530	-0,1250	0,0220	-0,1050	-0,0890
5	6,31		0,0311	1,7460	1,1980	0,0790	0,0650	-0,0260	-0,2580	-0,1270	0,0230	-0,1060	-0,0900
6	7,67		0,0377	1,7680	1,2130	0,0800	0,0650	-0,0250	-0,2600	-0,1270	0,0240	-0,1070	-0,0910
7	8,95		0,0440	1,7830	1,2250	0,0810	0,0660	-0,0240	-0,2610	-0,1280	0,0250	-0,1060	-0,0910
8	10,15		0,0499	1,7940	1,2320	0,0820	0,0660	-0,0240	-0,2620	-0,1280	0,0250	-0,1070	-0,0910
9	11,30		0,0556	1,8050	1,2400	0,0830	0,0670	-0,0230	-0,2620	-0,1280	0,0260	-0,1060	-0,0910
10	12,39		0,0609	1,8110	1,2450	0,0840	0,0670	-0,0230	-0,2620	-0,1280	0,0260	-0,1050	-0,0910
11	13,42		0,0660	1,8170	1,2490	0,0850	0,0670	-0,0220	-0,2620	-0,1270	0,0270	-0,1070	-0,0910
12	14,40		0,0708	1,8220	1,2510	0,0850	0,0670	-0,0210	-0,2620	-0,1270	0,0270	-0,1060	-0,0910
13	15,34		0,0754	1,8270	1,2550	0,0860	0,0670	-0,0210	-0,2620	-0,1270	0,0280	-0,1060	-0,0910
14	16,23		0,0798	1,8310	1,2580	0,0860	0,0670	-0,0200	-0,2620	-0,1270	0,0280	-0,1050	-0,0910
15	17,89		0,0880	1,8360	1,2610	0,0870	0,0680	-0,0190	-0,2620	-0,1260	0,0290	-0,1060	-0,0910
16	19,41		0,0955	1,8410	1,2660	0,0880	0,0680	-0,0190	-0,2620	-0,1260	0,0290	-0,1050	-0,0910
17	20,81		0,1023	1,8450	1,2690	0,0880	0,0680	-0,0180	-0,2620	-0,1260	0,0300	-0,1060	-0,0910

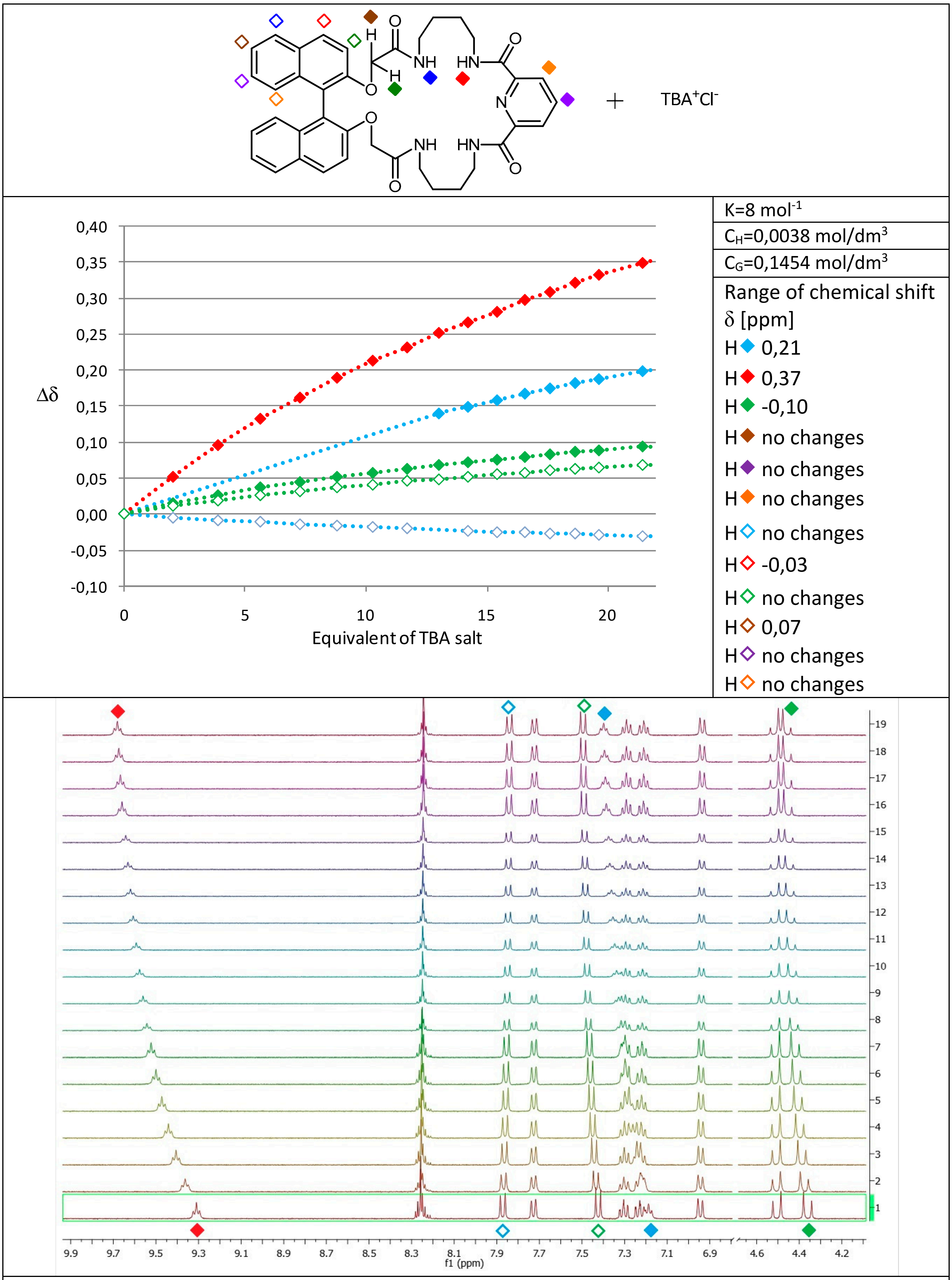

Fig. S37 The details of ^{1}H NMR titration of receptor ***(rac)*-3** with **Cl^{-}** in DMSO-d_6+0,5% water

**Table S10.** Experimental data used to determine binding constant of receptor **(*rac*)-3** with **Cl^-** in DMSO-d_6+0,5% water								
Point	Eq of guest	C_{host} [M]	C_{guest}[M]	Range of chemical shift Δδ [ppm]				
				H1◆	H2◆	H3◆	HAr1◇	HAr2◇
1	0,00	0,0038	0,0000	0,0000	0,0000	0,0000	0,0000	0,0000
2	1,99		0,0076	x	0,0530	0,0150	-0,0040	0,0110
3	3,86		0,0148	x	0,0960	0,0270	-0,0080	0,0190
4	5,61		0,0216	x	0,1320	0,0370	-0,0110	0,0270
5	7,26		0,0279	x	0,1620	0,0450	-0,0140	0,0320
6	8,82		0,0339	x	0,1900	0,0520	-0,0160	0,0380
7	10,29		0,0395	x	0,2130	0,0580	-0,0180	0,0420
8	11,68		0,0448	x	0,2320	0,0640	-0,0200	0,0460
9	12,99		0,0499	0,1400	0,2510	0,0680	x	0,0490
10	14,24		0,0547	0,1490	0,2670	0,0730	-0,0230	0,0530
11	15,43		0,0593	0,1590	0,2820	0,0760	-0,0240	0,0560
12	16,56		0,0636	0,1670	0,2970	0,0800	-0,0250	0,0580
13	17,64		0,0677	0,1740	0,3090	0,0830	-0,0260	0,0610
14	18,66		0,0717	0,1820	0,3210	0,0860	-0,0270	0,0630
15	19,64		0,0754	0,1880	0,3320	0,0880	-0,0280	0,0650
16	20,57		0,0825	0,1980	0,3490	0,0920	-0,0300	0,0680
17	21,47		0,0857	0,2020	0,3570	0,0940	-0,0300	0,0690
18	22,33		0,0889	0,2070	0,3640	0,0960	-0,0310	0,0700
19	23,15		0,0919	0,2100	0,3710	0,0970	-0,0320	0,0720

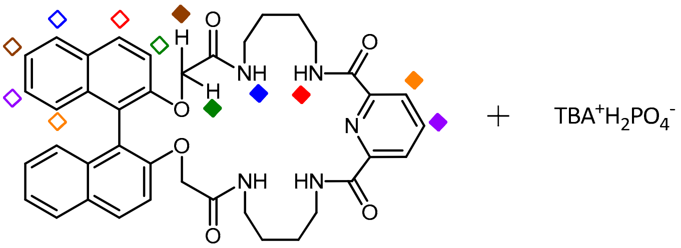

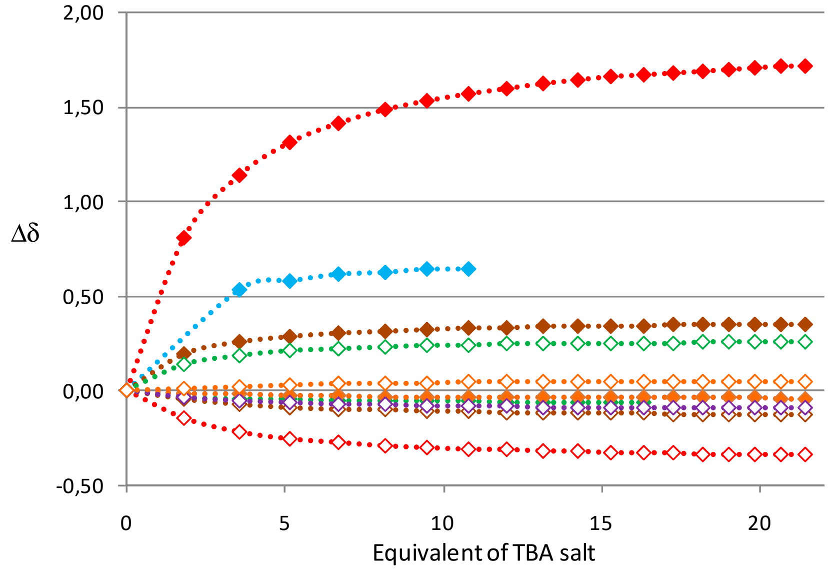

K=143 mol^{-1}
C_H=0,0037 mol/dm^3
C_G=0,2139 mol/dm^3
Range of chemical shift δ [ppm]
H◆ 0,64
H◆ 1,72
H◆ -0,06
H◆ 0,35
H◆ -0,04
H◆ -0,04
H◇ no changes
H◇ -0,34
H◇ 0,26
H◇ -0,13
H◇ -0,10
H◇ 0,05

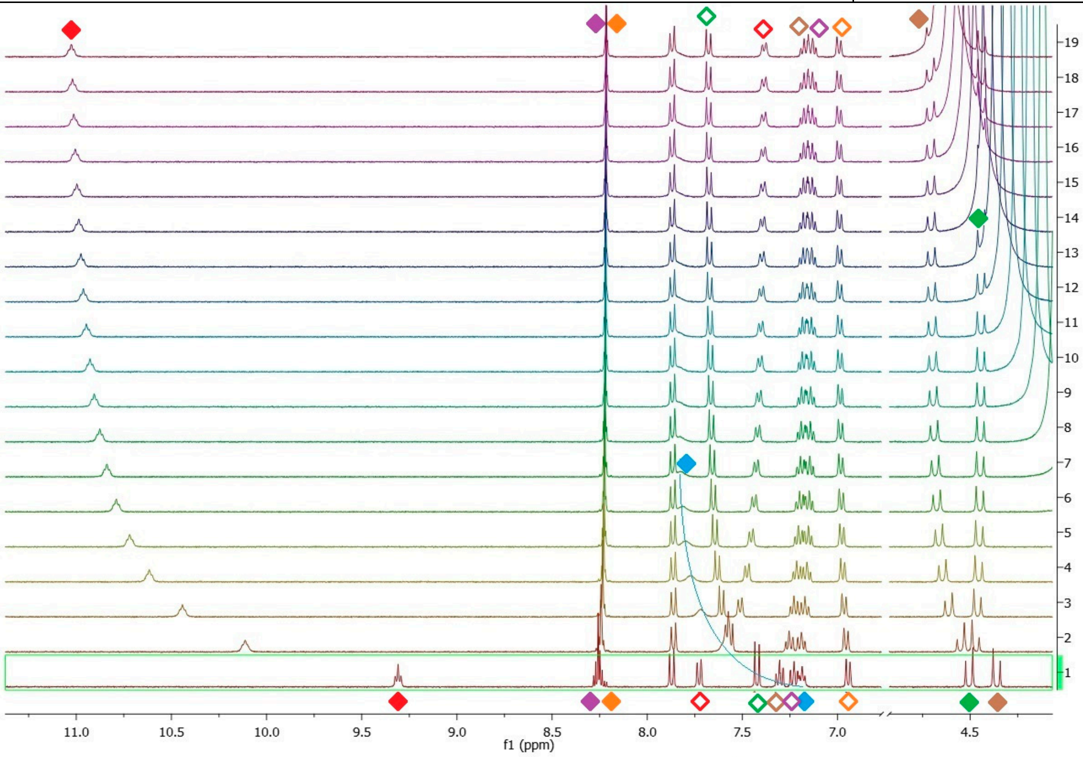

Fig. S38 The details of ^{1}H NMR titration of receptor ***(rac)*-3** with **$H_2PO_4^-$** in DMSO-d_6+0,5% water

Table S11. Experimental data used to determine binding constant of receptor ***(rac)*-3** with $\mathbf{H_2PO_4^-}$ in DMSO-d_6+0,5%

Point	Eq of guest	C_{host} [M]	C_{guest} [M]	Range of chemical shift Δδ [ppm]									
				H1 ◆	H2 ◆	H3 ◆	H4 ◆	HAr1 ◆	HAr2 ◆	HAr3 ◇	HAr4 ◇	HAr5 ◇	HAr6 ◇
1	0,00	0,0037	0,0000	0,0000	0,0000	0,0000	0,0000	0,0000	0,0000	0,0000	0,0000	0,0000	0,0000
2	1,84		0,0069	x	0,8040	-0,0340	0,1880	-0,1440	0,1390	-0,0490	-0,0380	0,0110	-0,0150
3	3,57		0,0134	0,5340	1,1330	-0,0430	0,2520	-0,2160	0,1860	-0,0750	-0,0560	0,0220	-0,0230
4	5,19		0,0195	0,5810	1,3070	-0,0500	0,2850	-0,2530	0,2090	-0,0890	-0,0670	0,0290	-0,0280
5	6,72		0,0252	0,6150	1,4100	-0,0530	0,3030	-0,2740	0,2220	-0,0980	-0,0730	0,0330	-0,0310
6	8,16		0,0306	0,6260	1,4800	-0,0560	0,3150	-0,2900	0,2300	-0,1040	-0,0780	0,0360	-0,0330
7	9,52		0,0357	0,6380	1,5290	-0,0580	0,3230	-0,3000	0,2350	-0,1090	-0,0810	0,0390	-0,0350
8	10,80		0,0405	0,6410	1,5680	-0,0590	0,3290	-0,3080	0,2390	-0,1130	-0,0840	0,0410	-0,0360
9	12,02		0,0451	x	1,5970	-0,0600	0,3330	-0,3150	0,2430	-0,1160	-0,0860	0,0420	-0,0370
10	13,18		0,0494	x	1,6180	-0,0610	0,3370	-0,3200	0,2450	-0,1180	-0,0880	0,0430	-0,0380
11	14,28		0,0535	x	1,6390	-0,0620	0,3390	-0,3240	0,2470	-0,1200	-0,0890	0,0440	-0,0380
12	15,32		0,0574	x	1,6540	-0,0620	0,3420	-0,3280	0,2490	-0,1220	-0,0910	0,0450	-0,0390
13	16,31		0,0611	x	1,6650	-0,0630	0,3430	-0,3310	0,2500	-0,1230	-0,0920	0,0450	-0,0400
14	17,26		0,0647	x	1,6770	x	0,3440	-0,3340	0,2510	-0,1240	-0,0930	0,0470	-0,0400
15	19,03		0,0713	x	1,6880	x	0,3450	-0,3360	0,2520	-0,1250	-0,0940	0,0470	-0,0400
16	19,86		0,0744	x	1,6970	x	0,3470	-0,3380	0,2530	-0,1260	-0,0940	0,0480	-4,10E-02
17	20,65		0,0774	x	1,7040	x	0,3470	-0,3390	0,2540	-0,1270	-0,0950	0,0480	-4,10E-02
18	21,41		0,0802	x	1,7110	x	0,3490	-0,3410	0,2540	-0,1280	-0,0950	0,0480	-4,20E-02
19	22,14		0,0830	x	1,7160	x	0,3490	-0,3420	0,2550	-0,1290	-0,0960	0,0490	-4,20E-02

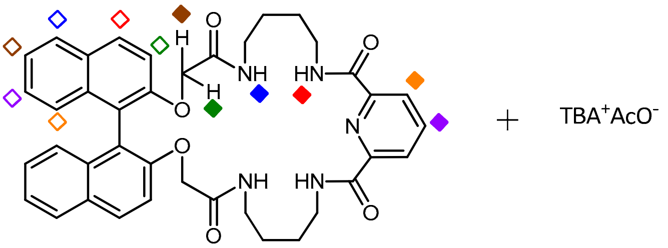

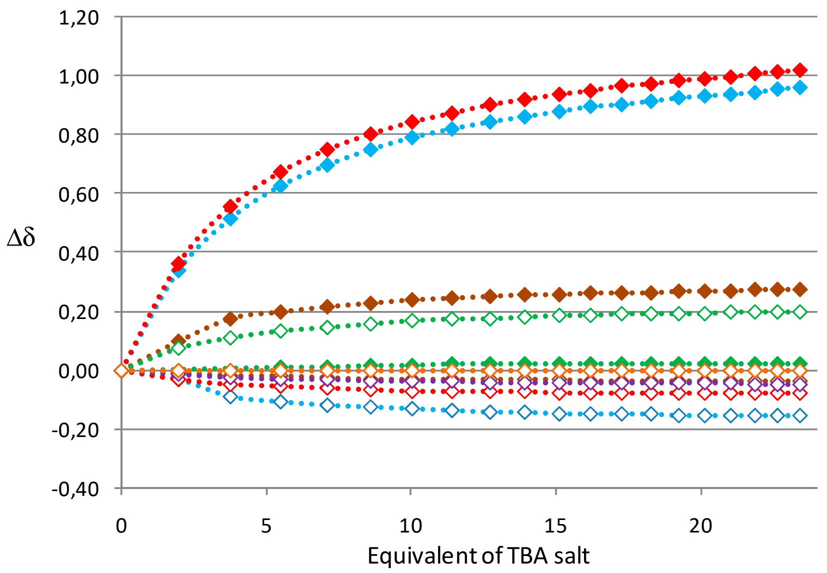

$K=60\ mol^{-1}$

$C_H=0,0043\ mol/dm^3$

$C_G=0,\ mol/dm^3$

Range of chemical shift δ [ppm]

H◆ 0,96
H◆ 1,02
H◆ 0,02
H◆ 0,27
H◆ no changes
H◆ no changes
H◇ -0,16
H◇ 0,08
H◇ 0,20
H◇ -0,04
H◇ -0,05
H◇ no changes

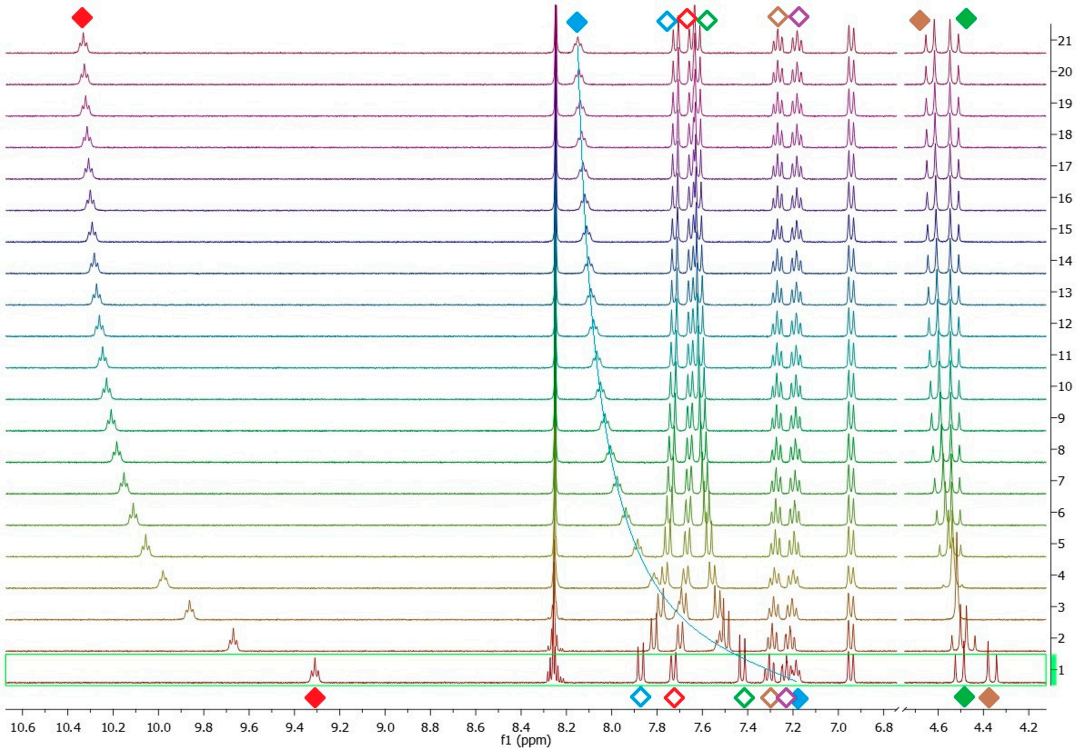

Fig. S39 The details of ^{1}H NMR titration of receptor ***(rac)*-3** with **AcO**$^-$ in DMSO-d_6+0,5% water

Table S12. Experimental data used to determine binding constant of receptor ***(rac)*-3** with **AcO**$^-$ in DMSO-d_6+0,5%

Point	Eq of guest	C_{host} [M]	C_{guest} [M]	Range of chemical shift Δδ [ppm]								
				H1◆	H2◆	H3◆	H4◆	HAr1◇	HAr2◇	HAr3◇	HAr4◇	HAr5◇
1	0,00	0,0043	0,0000	0,0000	0,0000	0,0000	0,0000	0,0000	0,0000	0,0000	0,0000	0,0000
2	1,95		0,0085	0,3360	0,3590	x	0,0960	x	-0,0310	0,0720	-0,0100	-0,0160
3	3,77		0,0164	0,5170	0,5530	x	0,1760	-0,0880	-0,0460	0,1100	-0,0190	-0,0240
4	5,49		0,0238	0,6260	0,6700	0,0100	0,1960	-0,1060	-0,0540	0,1340	x	-0,0310
5	7,10		0,0308	0,6990	0,7460	0,0130	0,2130	-0,1190	-0,0620	0,1470	-0,0260	-0,0330
6	8,62		0,0374	0,7510	0,8010	0,0160	0,2260	-0,1270	-0,0660	0,1580	-0,0280	-0,0360
7	10,06		0,0437	0,7890	0,8420	0,0180	0,2360	-0,1330	-0,0690	0,1660	-0,0300	-0,0380
8	11,42		0,0496	0,8190	0,8740	0,0190	0,2430	-0,1370	-0,0710	0,1720	-0,0310	-0,0390
9	12,71		0,0552	0,8430	0,8990	0,0200	0,2490	-0,1410	-0,0730	0,1770	-0,0320	-0,0410
10	13,93		0,0605	0,8630	0,9190	0,0210	0,2540	-0,1430	-0,0740	0,1800	-0,0320	-0,0410
11	15,09		0,0655	0,8790	0,9360	0,0220	0,2570	-0,1460	-0,0760	0,1840	-0,0330	-0,0420
12	16,19		0,0703	0,8930	0,9510	0,0220	0,2610	-0,1480	-0,0760	0,1860	-0,0340	-0,0430
13	17,24		0,0749	0,9040	0,9630	0,0220	0,2630	-0,1490	-0,0770	0,1890	-0,0340	-0,0440
14	18,25		0,0792	0,9150	0,9730	0,0230	0,2650	-0,1500	-0,0770	0,1910	-0,0340	-0,0440
15	19,20		0,0834	0,9230	0,9830	0,0230	0,2670	-0,1520	-0,0790	0,1920	-0,0350	-0,0450
16	20,12		0,0873	0,9320	0,9910	0,0230	0,2690	-0,1520	-0,0790	0,1930	-0,0360	-0,0450
17	20,99		0,0911	0,9390	0,9980	0,0230	0,2700	-0,1530	-0,0790	0,1950	-0,0360	-0,0450
18	21,83		0,0948	0,9450	1,0050	0,0240	0,2720	-0,1530	-0,0790	0,1960	-0,0360	-0,0460
19	22,63		0,0983	0,9520	1,0110	0,0240	0,2730	-0,1540	-0,0800	0,1970	-0,0360	-0,0460
20	23,40		0,1016	0,9570	1,0170	0,0240	0,2740	-0,1550	-0,0800	0,1980	-0,0360	-0,0470
21	24,14		0,1048	0,9630	1,0220	0,0240	0,2750	-0,1550	-0,0800	0,1990	-0,0370	-0,0470

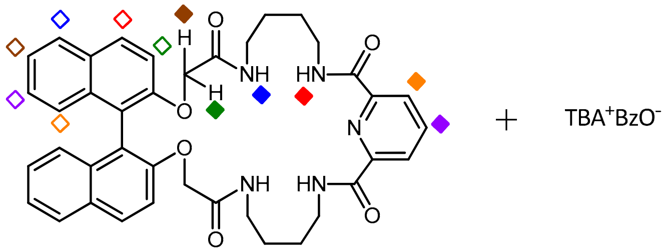

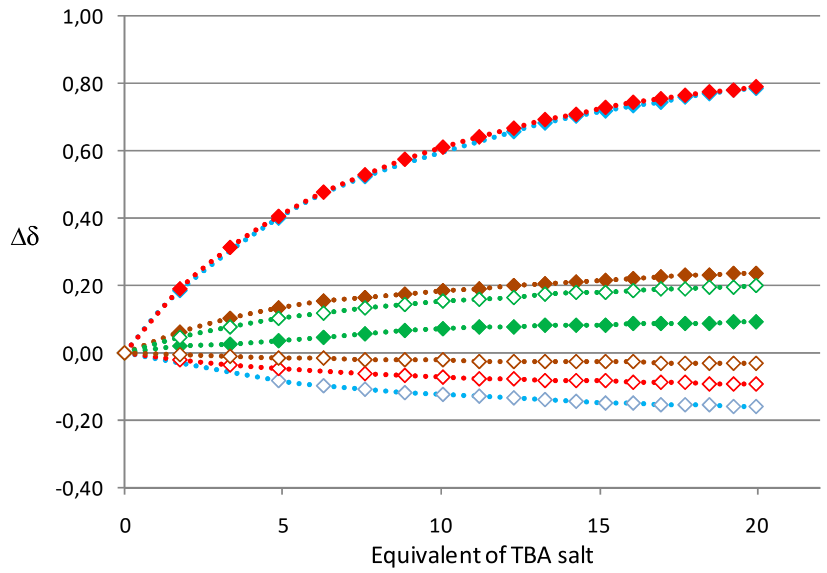

K=25 mol^{-1}
C_H=0, 0043mol/dm^3
C_G=0, mol/dm^3
Range of chemical shift δ [ppm]
H◆ 0,78
H◆ 0,78
H◆ 0,09
H◆ 0,24
H◆ covered
H◆ no changes
H◇ -0,16
H◇ -0,09
H◇ 0,20
H◇ no changes
H◇ no changes
H◇ no changes

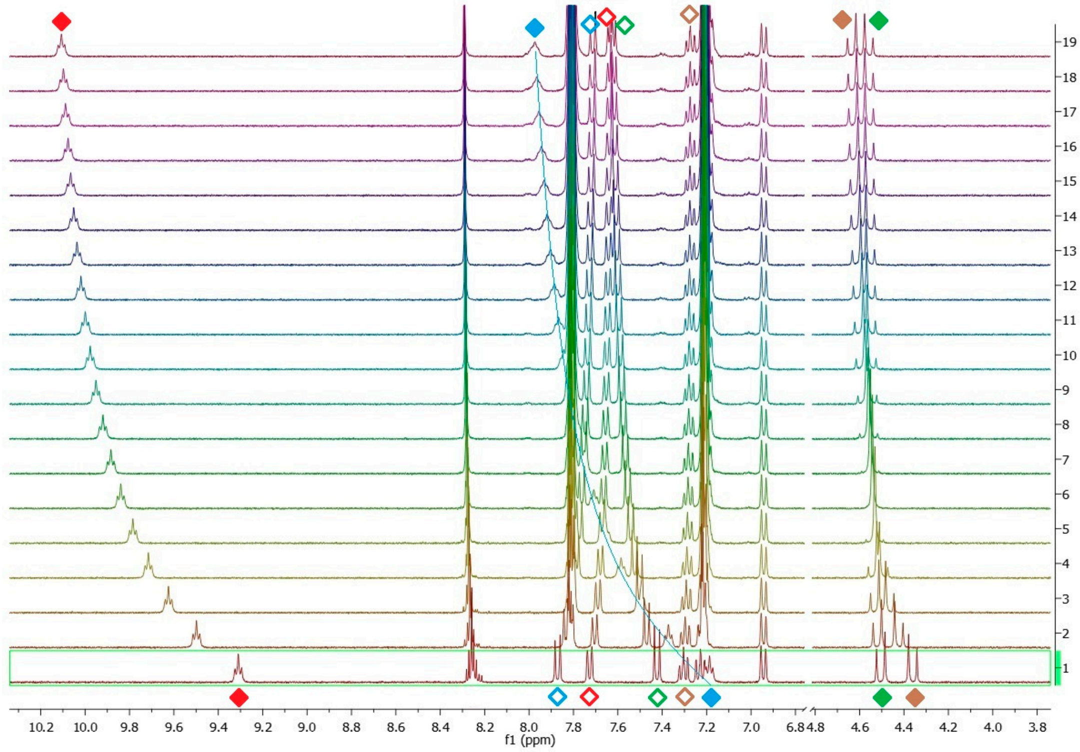

Fig. S40 The details of ^{1}H NMR titration of receptor ***(rac)*-3** with **BzO^-** in DMSO-d_6+0,5% water

Table S13. Experimental data used to determine binding constant of receptor ***(rac)*-3** with **BzO$^-$** in DMSO-d_6+0,5% water

Point	Eq of guest	C_{host} [M]	C_{guest} [M]	Range of chemical shift Δδ [ppm]						
				H1◆	H2◆	H3◆	H4◆	HAr1◇	HAr2◇	HAr3◇
1	0,00	0,0043	0,0000	H1	H2	H3	H4	HAr1	HAr2	HAr3
2	1,90		0,0085	0,0000	0,0000	0,0000	0,0000	0,0000	0,0000	0,0000
3	3,69		0,0164	0,1860	0,1880	0,0180	0,0630	x	-0,0230	0,0470
4	5,36		0,0238	x	0,3140	0,0270	0,1040	x	-0,0380	0,0780
5	6,94		0,0308	0,3990	0,4050	0,0370	0,1320	-0,0840	-0,0490	0,1010
6	8,43		0,0374	x	0,4750	0,0450	0,1520	-0,0980	x	0,1180
7	9,83		0,0437	0,5220	0,5300	0,0560	0,1630	-0,1090	-0,0640	0,1320
8	11,16		0,0496	x	0,5740	0,0650	0,1720	-0,1180	-0,0690	0,1430
9	12,42		0,0552	x	0,6090	0,0710	0,1820	-0,1250	-0,0720	0,1520
10	13,61		0,0605	x	0,6410	0,0750	0,1910	-0,1310	-0,0770	0,1600
11	14,74		0,0655	0,6570	0,6670	0,0780	0,1980	-0,1350	-0,0790	0,1660
12	15,82		0,0703	0,6800	0,6900	0,0800	0,2050	-0,1400	-0,0820	0,1720
13	16,85		0,0749	0,7000	0,7090	0,0820	0,2110	-0,1430	-0,0840	0,1770
14	17,83		0,0792	0,7180	0,7270	0,0840	0,2160	-0,1470	-0,0850	0,1810
15	19,66		0,0834	0,7310	0,7410	0,0860	0,2200	-0,1490	-0,0870	0,1850
16	20,51		0,0873	0,7440	0,7550	0,0870	0,2250	-0,1520	-0,0890	0,1880
17	21,33		0,0911	0,7570	0,7650	0,0890	0,2290	-0,1530	-0,0900	0,1910
18	22,12		0,0948	0,7680	0,7720	0,0890	0,2320	-0,1560	-0,0920	0,1930
19	22,87		0,0983	0,7790	0,7800	0,0900	0,2350	-0,1570	-0,0920	0,1960

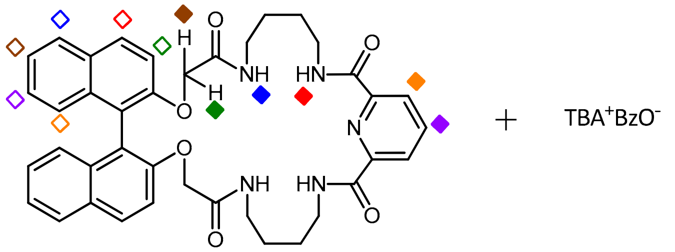

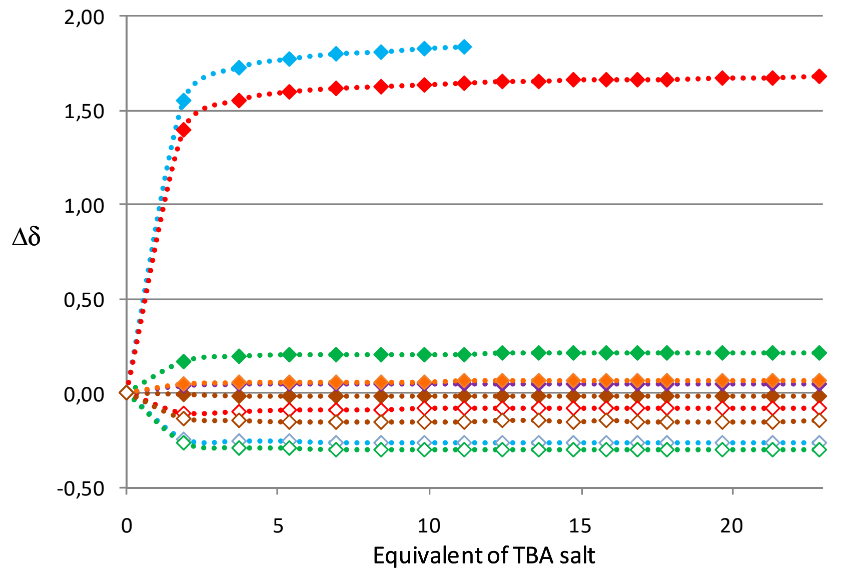

K=972 mol^{-1}
C_H=0,046 mol/dm^3
C_G=0, mol/dm^3
Range of chemical shift δ [ppm]
H◆ 1,83
H◆ 1,65
H◆ 0,21
H◆ -0,01
H◆ 0,05
H◆ 0,07
H◇ -0,184
H◇ -0,24
H◇ -0,14
H◇ no changes
H◇ no changes
H◇ no changes

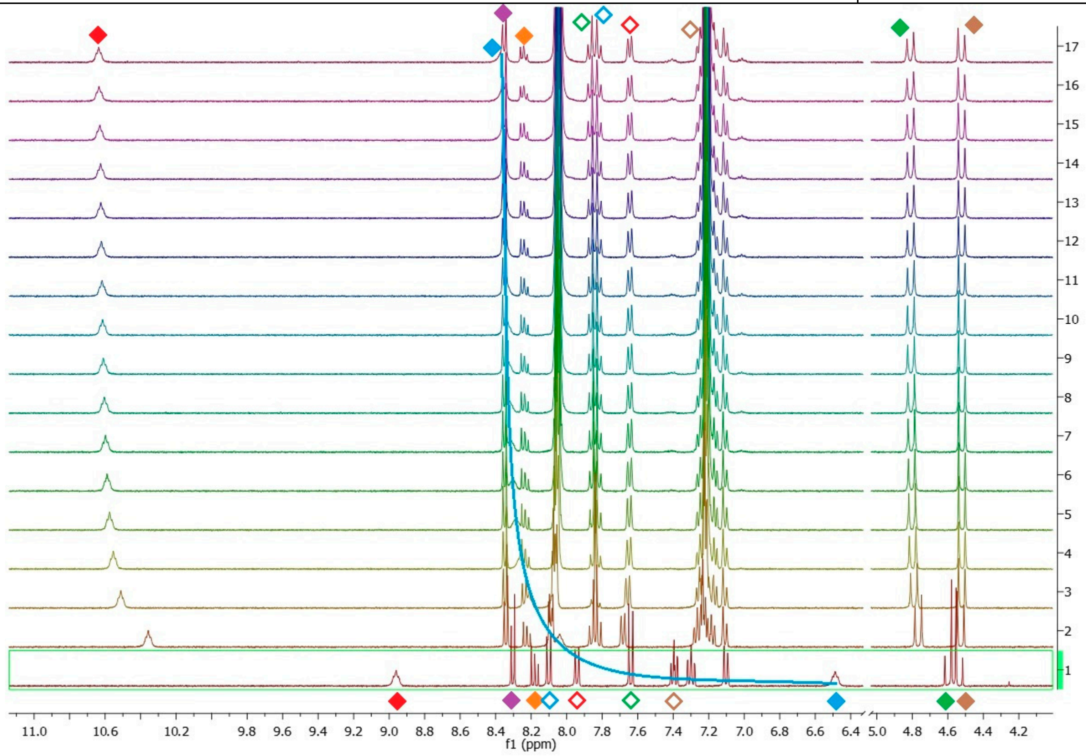

Fig. S41 The details of ^{1}H NMR titration of receptor ***(rac)*-3** with **BzO$^-$** in acetone-d_6+0,5% water

Table S14. Experimental data used to determine binding constant of receptor **(*rac*)-3** with **BzO**$^-$ in acetone-d_6+0,5%

Point	Eq of guest	C_{host} [M]	C_{guest} [M]	Range of chemical shift Δδ [ppm]								
				H1◆	H2◆	H3◆	H4◆	HAr1◆	HAr2◆	HAr3◇	HAr4◇	HAr5◇
1	0,00	0,0046	0,0000	0,0000	0,0000	0,0000	0,0000	0,0000	0,0000	0,0000	0,0000	0,0000
2	1,90		0,0088	1,5520	1,3970	0,1690	-0,0080	0,0510	-0,2410	-0,1020	-0,2590	-0,1310
3	3,69		0,0171	1,7230	1,5510	0,1940	-0,0140	0,0570	-0,2520	-0,0940	-0,2860	-0,1440
4	5,36		0,0248	1,7710	1,5940	0,2010	-0,0150	0,0590	-0,2570	-0,0890	-0,2930	-0,1480
5	6,94		0,0321	1,7980	1,6150	0,2040	-0,0150	0,0600	-0,2590	-0,0870	-0,2950	-0,1480
6	8,43		0,0390	1,8120	1,6290	0,2060	-0,0150	0,0610	-0,2600	-0,0850	-0,2960	-0,1480
7	9,83		0,0455	1,8260	1,6380	0,2080	-0,0150	0,0620	-0,2610	-0,0830	-0,2970	-0,1490
8	11,16		0,0517	1,8340	1,6450	0,2090	-0,0150	0,0630	-0,2610	-0,0820	-0,2970	-0,1490
9	12,42		0,0575	x	1,6510	0,2100	-0,0150	0,0640	-0,2610	-0,0810	-0,2980	-0,1470
10	13,61		0,0630	x	1,6540	0,2100	-0,0150	0,0640	-0,2610	-0,0800	-0,2980	-0,1470
11	14,74		0,0683	x	1,6570	0,2110	-0,0140	0,0640	-0,2610	-0,0790	-0,2980	-0,1480
12	15,82		0,0732	x	1,6620	0,2120	-0,0140	0,0650	-0,2610	-0,0780	-0,2980	-0,1470
13	16,85		0,0780	x	1,6640	0,2120	-0,0140	0,0660	-0,2610	-0,0780	-0,2980	-0,1480
14	17,83		0,0825	x	1,6660	0,2120	-0,0140	0,0660	-0,2600	-0,0770	-0,2980	-0,1480
15	19,66		0,0910	x	1,6700	0,2130	-0,0130	0,0670	-0,2600	-0,0760	-0,2970	-0,1480
16	21,33		0,0987	x	1,6740	0,2140	-0,0120	0,0680	-0,2600	-0,0750	-0,2970	-0,1480
17	22,87		0,1059	x	1,6760	0,2140	-0,0120	0,0680	-0,2600	-0,0750	-0,2970	-0,1470

2.3. ^{1}H NMR titration data from experiments with macrocyclic compound (*R*)-1

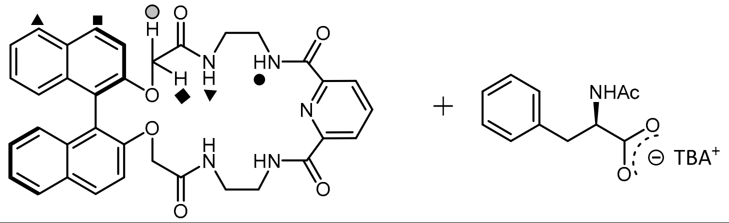

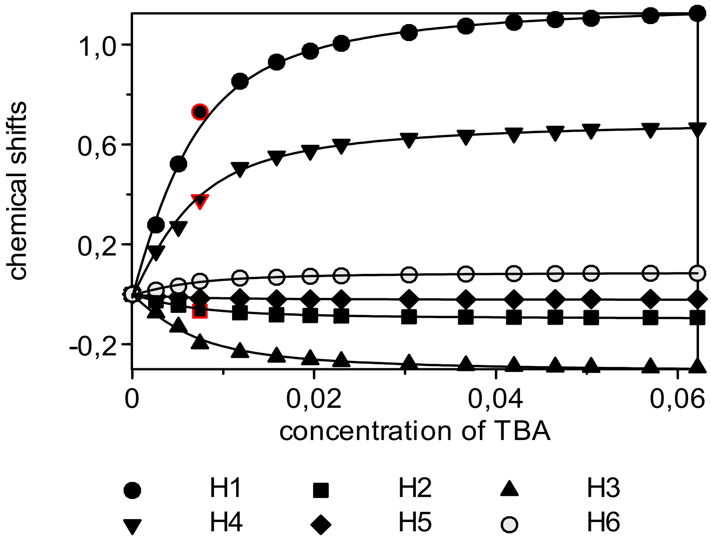

Changes in chemical shifts of the receptor (R)-**1** protons upon addition of anion

K=318 M^{-1}

C_H=5,80·10^{-3} M

C_G=0,110 M

Range of chemical shift
δ [ppm]

H1	1,125
H2	-0,93
H3	-0,294
H4	0,666
H5	-0,019
H6	0,084

Eq of anion	
10,70	15
9,81	14
8,69	13
8,00	12
7,23	11
6,31	10
5,24	9
3,96	8
3,37	7
2,73	6
2,04	5
1,28	4
0,87	3
0,45	2
0	1

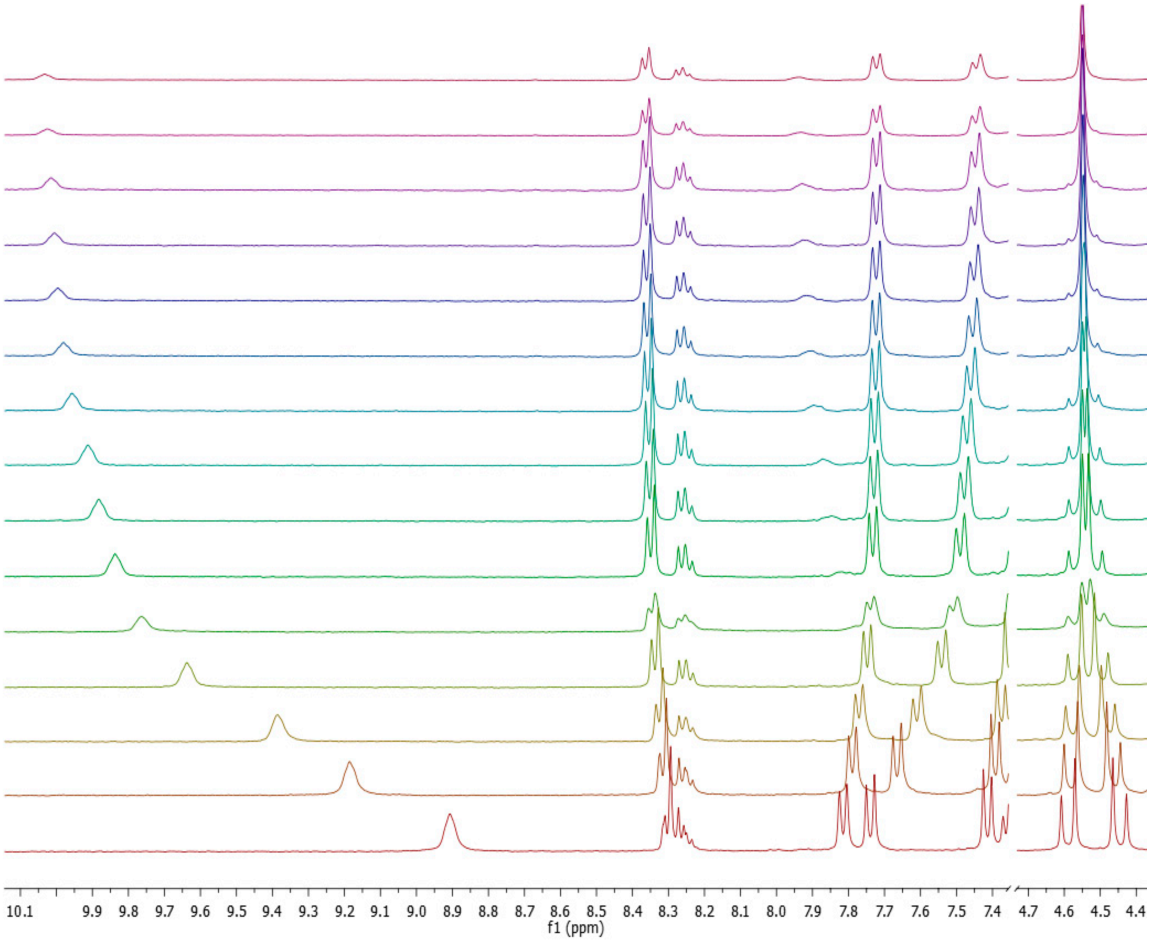

^{1}H NMR spectra changes upon titration of the receptor (*R*)-**1** with anion

Fig. S42 The details of ^{1}H NMR titration experiments of receptor **(*R*)-1** with **D-14** in acetone-d_6+0,5% H_2O

**Table S15.** Experimental data used to determine binding constant of receptor **(*R*)-1** with **D-14** in acetone-d_6+0,5% water										
Point	Eq of guest	C_{host} [M]	C_{guest} [M]	Range of chemical shift Δδ [ppm]						
				H1	H2	H3	H4	H5	H6	H7
1	0,00	0,0058	0,0000	0,000	0,000	0,000	0,000	0,000	0,000	0,000
2	0,45		0,0026	0,278	-0,026	-0,074	0,173	-0,008	0,017	0,278
3	0,87		0,0051	0,522	-0,044	-0,130	0,270	-0,011	0,033	0,522
4	1,28		0,0074	0,730	-0,067	-0,198	0,378	-0,017	0,052	0,730
5	2,04		0,0118	0,853	-0,074	-0,232	0,507	-0,017	0,065	0,853
6	2,73		0,0159	0,930	-0,082	-0,251	0,553	-0,021	0,068	0,930
7	3,37		0,0196	0,974	-0,085	-0,261	0,576	-0,021	0,072	0,974
8	3,96		0,0230	1,005	-0,087	-0,269	0,600	-0,021	0,074	1,005
9	5,24		0,0304	1,048	-0,090	-0,279	0,624	-0,020	0,078	1,048
10	6,31		0,0367	1,074	-0,091	-0,285	0,636	-0,021	0,081	1,074
11	7,23		0,0420	1,089	-0,092	-0,288	0,645	-0,021	0,083	1,089
12	8,01		0,0465	1,100	-0,092	-0,291	0,652	-0,020	0,083	1,100
13	8,69		0,0505	1,105	-0,093	-0,292	0,660	-0,019	0,084	1,105
14	9,81		0,0570	1,116	-0,093	-0,294	0,664	-0,019	0,084	1,116
15	10,70		0,0622	1,125	-0,093	-0,294	0,666	-0,019	0,084	1,125

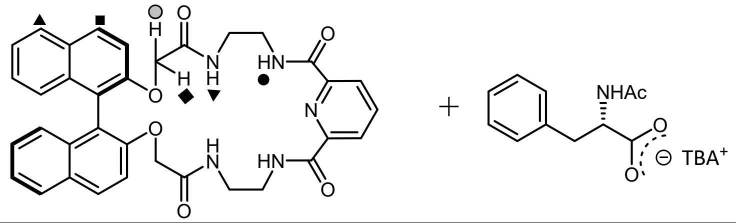

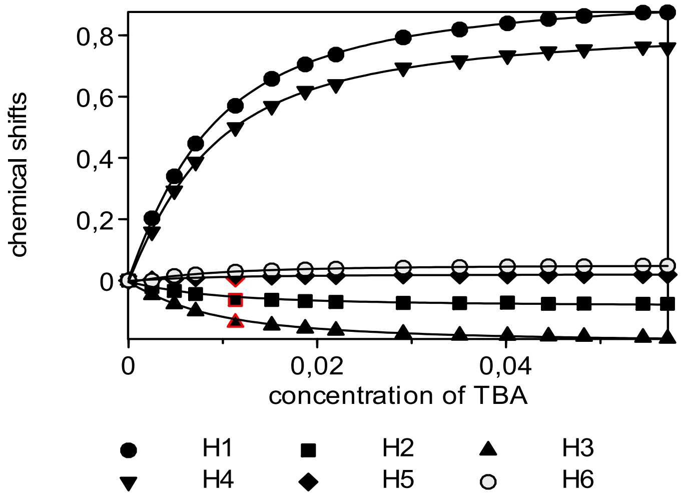

Changes in chemical shifts of the receptor (R)-**1** protons upon addition of anion

**K=187 M^{-1}**
C_H= 5,80·10^{-3} M
C_G=0,102 M
Range of chemical shift<br>δ [ppm]<br>H1 0,875<br>H2 -0,076<br>H3 -0,189<br>H4 0,760<br>H5 0,019<br>H6 0,049

Eq of anion	
9,83	15
9,38	14
8,31	13
7,66	12
6,91	11
6,04	10
5,01	9
3,78	8
3,22	7
2,61	6
1,95	5
1,22	4
0,84	3
0,43	2
0	1

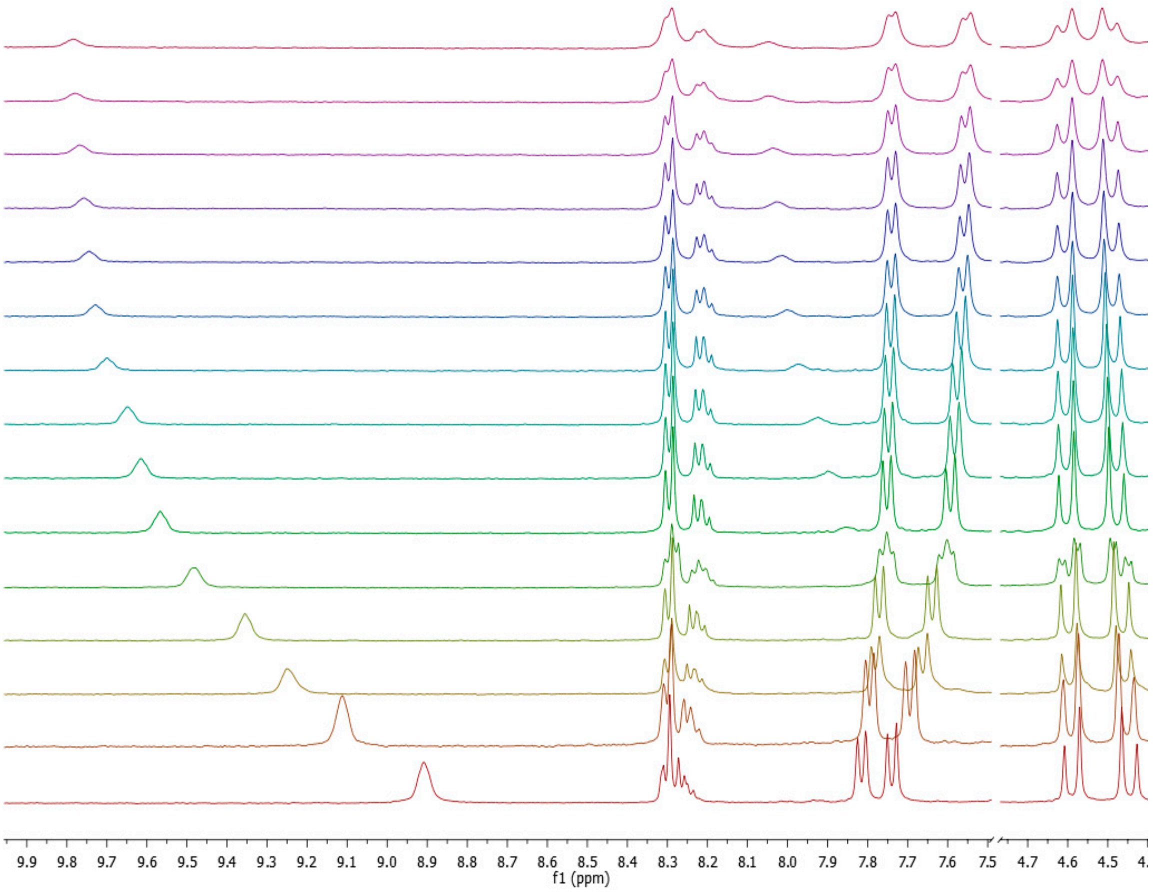

^{1}H NMR spectra changes upon titration of the receptor (R)-**1** with anion

Fig. S43 The details of ^{1}H NMR titration experiments of receptor **(*R*)-1** with **L-14** in acetone-d_6+0,5% H_2O

Table S16. Experimental data used to determine binding constant of receptor **(*R*)-1** with **L-14** in acetone-d_6+0,5% water

Point	Eq of guest	C_{host} [M]	C_{guest} [M]	Range of chemical shift Δδ [ppm]						
				H1	H2	H3	H4	H5	H6	H7
1	0,00	0,0058	0,0000	0,000	0,000	0,000	0,000	0,000	0,000	0,000
2	0,43		0,0025	0,203	-0,021	-0,047	0,159	0,002	0,000	0,203
3	0,84		0,0049	0,340	-0,034	-0,077	0,293	0,005	0,015	0,340
4	1,22		0,0071	0,447	-0,044	-0,100	0,387	0,009	0,020	0,447
5	1,95		0,0113	0,570	-0,063	-0,137	0,499	0,008	0,029	0,570
6	2,61		0,0152	0,658	-0,063	-0,146	0,569	0,014	0,033	0,658
7	3,22		0,0187	0,705	-0,067	-0,156	0,618	0,016	0,036	0,705
8	3,78		0,0220	0,737	-0,070	-0,163	0,640	0,016	0,039	0,737
9	5,01		0,0291	0,793	-0,073	-0,173	0,694	0,018	0,042	0,793
10	6,04		0,0351	0,819	-0,074	-0,179	0,718	0,018	0,044	0,819
11	6,91		0,0401	0,839	-0,072	-0,180	0,734	0,018	0,046	0,839
12	7,66		0,0445	0,853	-0,076	-0,182	0,747	0,019	0,047	0,853
13	8,31		0,0483	0,863	-0,075	-0,183	0,754	0,019	0,047	0,863
14	9,38		0,0545	0,874	-0,076	-0,186	0,763	0,019	0,049	0,874
15	9,83		0,0571	0,875	-0,076	-0,189	0,760	0,019	0,048	0,875

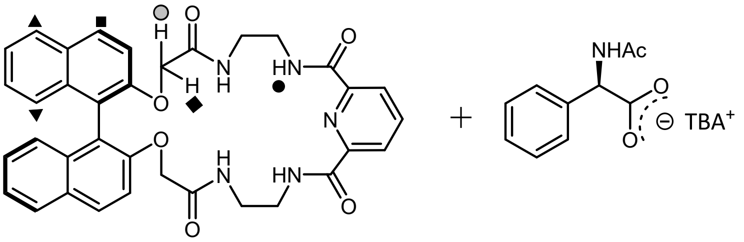

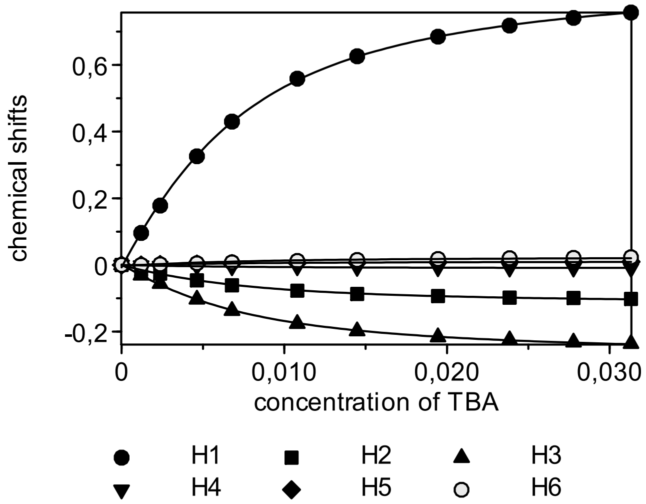

Changes in chemical shifts of the receptor (*R*)-**1** protons upon addition of anion

K=229 M^{-1}

C_H=5,25·10^{-3} M

C_G=0,0972 M

Range of chemical shift

δ [ppm]

H1 0,815

H2 -0,106

H3 -0,249

H4 -0,010

H5 0,011

H6 0,027

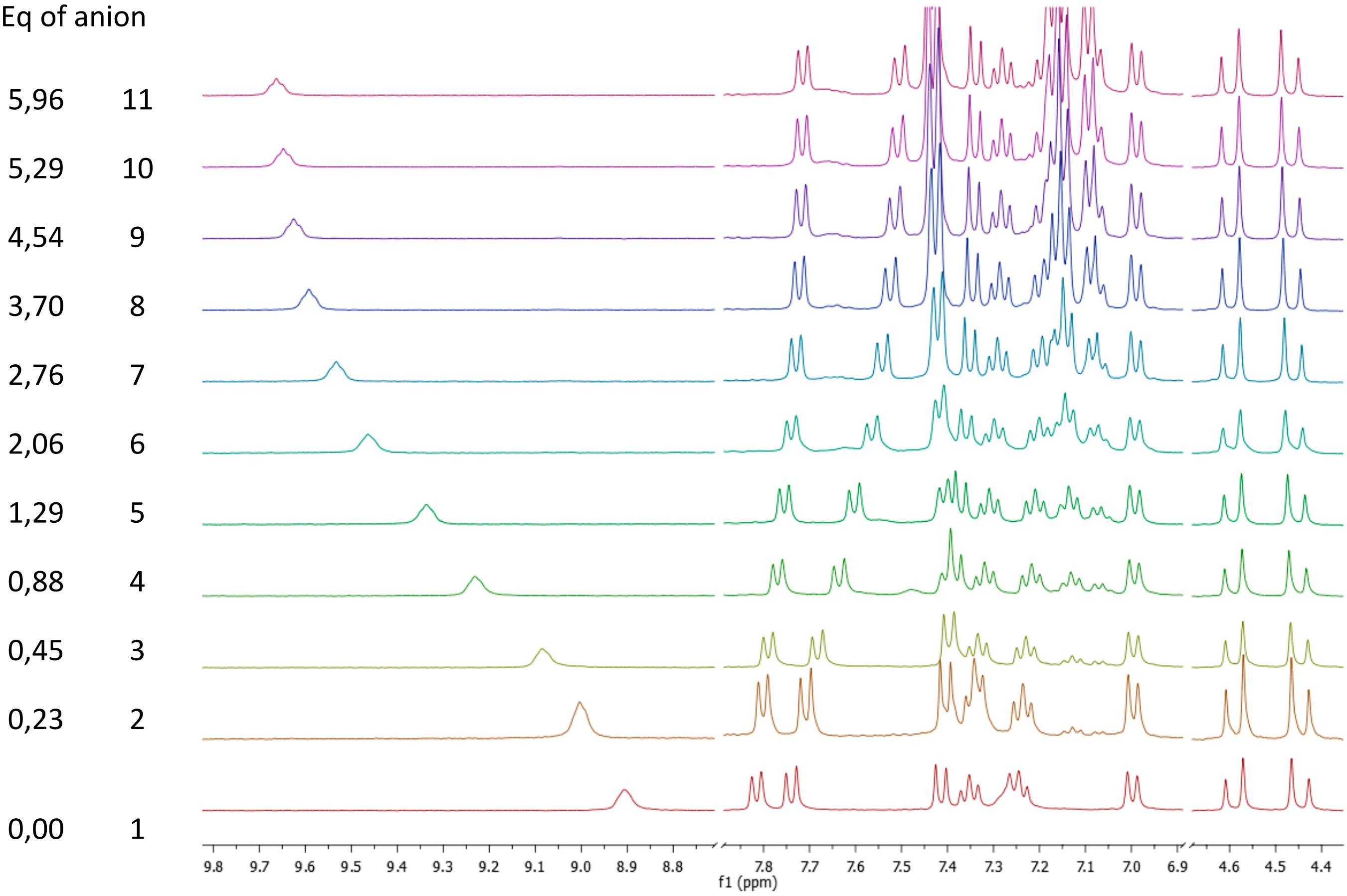

^{1}H NMR spectra changes upon titration of the receptor (*R*)-**1** with anion

Fig. S44 The details of ^{1}H NMR titration experiments of receptor **(*R*)-1** with **D-12** in acetone-d_6+0,5% H_2O

**Table S17.** Experimental data used to determine binding constant of receptor **(*R*)-1** with **D-12** in acetone-d_6+0,5% water										
Point	Eq of guest	C_{host} [M]	C_{guest} [M]	Range of chemical shift Δδ [ppm]						
				H1	H2	H3	H4	H5	H6	H7
1	0,00	0,0053	0,0000	0,000	0,000	0,000	0,000	0,000	0,000	0,000
2	0,23		0,0012	0,096	-0,015	-0,031	-0,002	0,000	0,000	0,096
3	0,45		0,0024	0,178	-0,026	-0,056	-0,003	0,001	0,002	0,178
4	0,88		0,0046	0,326	-0,046	-0,103	-0,003	0,003	0,005	0,326
5	1,29		0,0068	0,430	-0,061	-0,137	-0,005	0,004	0,008	0,430
6	2,06		0,0108	0,559	-0,077	-0,176	-0,005	0,006	0,012	0,559
7	2,76		0,0145	0,626	-0,087	-0,198	-0,007	0,007	0,015	0,626
8	3,70		0,0194	0,685	-0,093	-0,216	-0,008	0,008	0,018	0,685
9	4,54		0,0238	0,718	-0,098	-0,225	-0,009	0,009	0,020	0,718
10	5,29		0,0278	0,741	-0,099	-0,232	-0,009	0,009	0,021	0,741
11	5,96		0,0313	0,757	-0,102	-0,236	-0,010	0,010	0,023	0,757

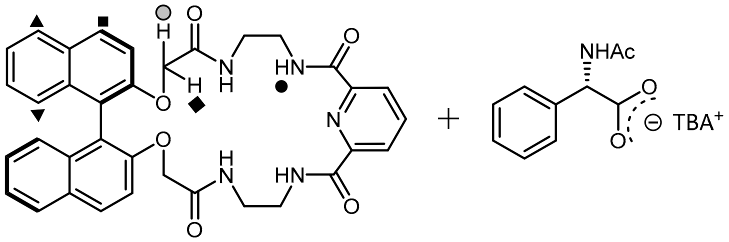

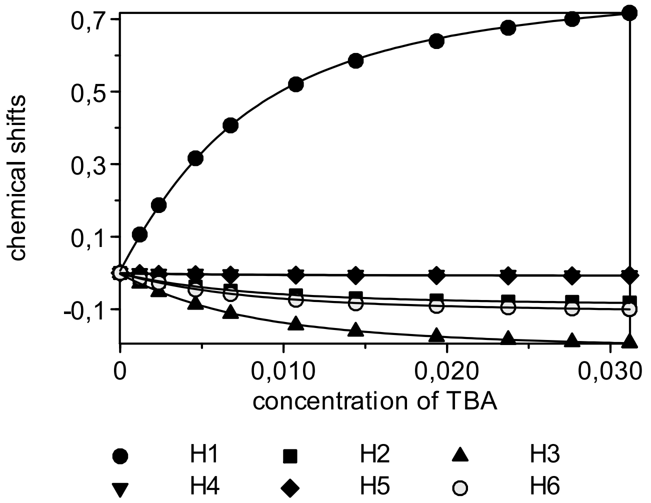

Changes in chemical shifts of the receptor (*R*)-**1** protons upon addition of anion

K=221 M^{-1}

C_H=5,25·10^{-3} M

C_G=0,0968 M

Range of chemical shift δ [ppm]

H1 0,717

H2 -0,082

H3 -0,193

H4 -0,007

H5 -0,007

H6 -0,100

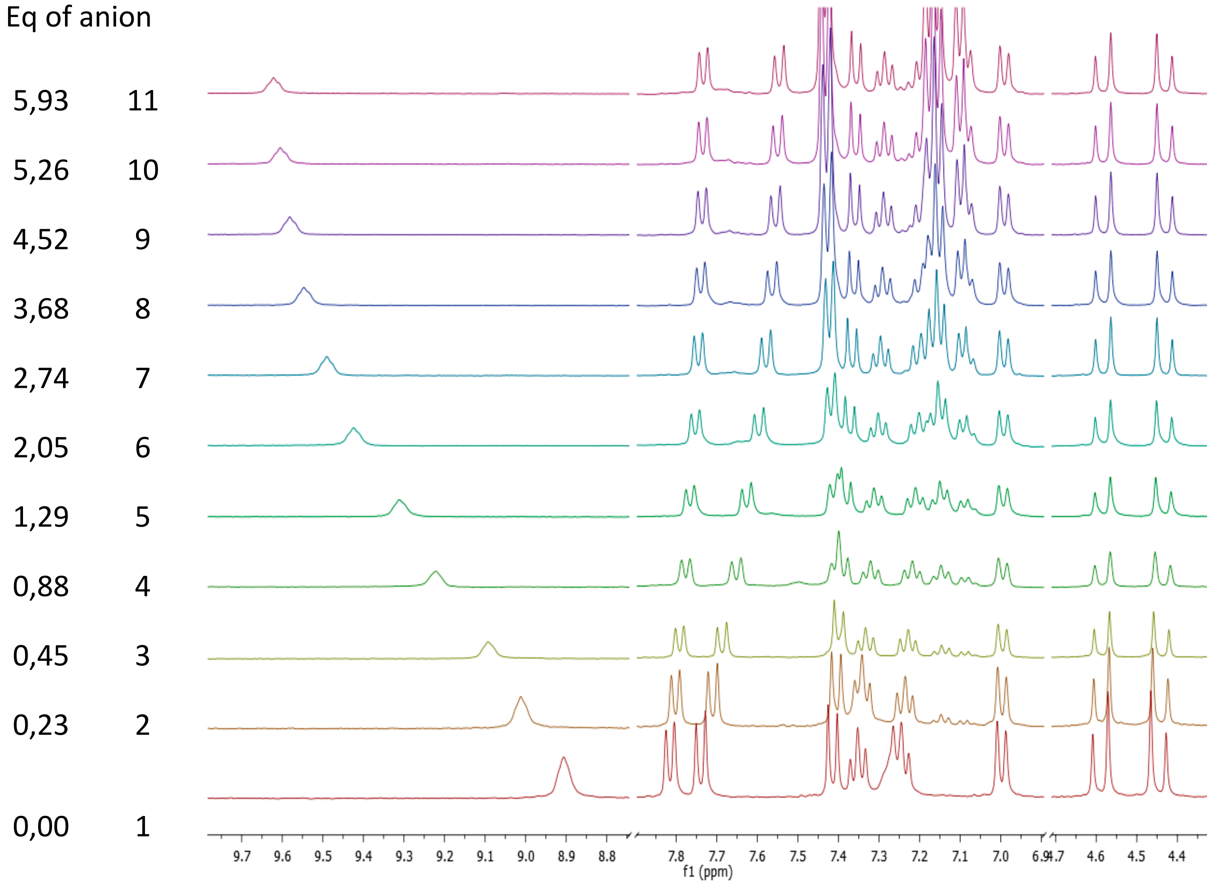

^{1}H NMR spectra changes upon titration of the receptor (*R*)-**1** with anion

Fig. S45 The details of ^{1}H NMR titration experiments of receptor **(*R*)-1** with **L-12** in acetone-d_6+0,5% H_2O

**Table S18.** Experimental data used to determine binding constant of receptor **(*R*)-1** with **L-12** in acetone-d_6+0,5% water										
Point	Eq of guest	C_{host} [M]	C_{guest} [M]	Range of chemical shift Δδ [ppm]						
				H1	H2	H3	H4	H5	H6	H7
1	0,00	0,0053	0,0000	0,000	0,000	0,000	0,000	0,000	0,000	0,000
2	0,23		0,0012	0,106	-0,013	-0,029	-0,001	-0,002	-0,015	0,106
3	0,45		0,0024	0,187	-0,024	-0,052	-0,003	-0,004	-0,027	0,187
4	0,88		0,0046	0,316	-0,038	-0,087	-0,003	-0,005	-0,046	0,316
5	1,29		0,0068	0,407	-0,049	-0,112	-0,004	-0,006	-0,058	0,407
6	2,05		0,0108	0,520	-0,062	-0,144	-0,005	-0,006	-0,074	0,520
7	2,74		0,0144	0,585	-0,070	-0,161	-0,006	-0,007	-0,084	0,585
8	3,68		0,0194	0,639	-0,076	-0,176	-0,006	-0,007	-0,091	0,639
9	4,52		0,0237	0,676	-0,079	-0,184	-0,006	-0,008	-0,095	0,676
10	5,26		0,0277	0,700	-0,080	-0,190	-0,007	-0,008	-0,098	0,700
11	5,93		0,0312	0,717	-0,082	-0,193	-0,007	-0,007	-0,100	0,717

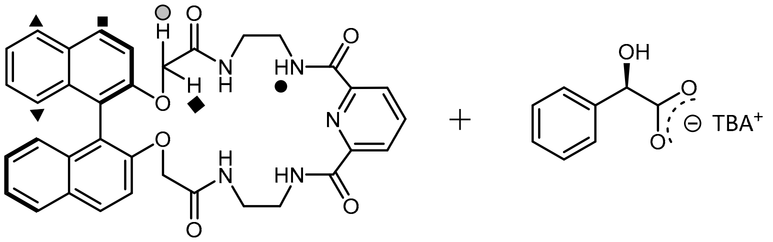

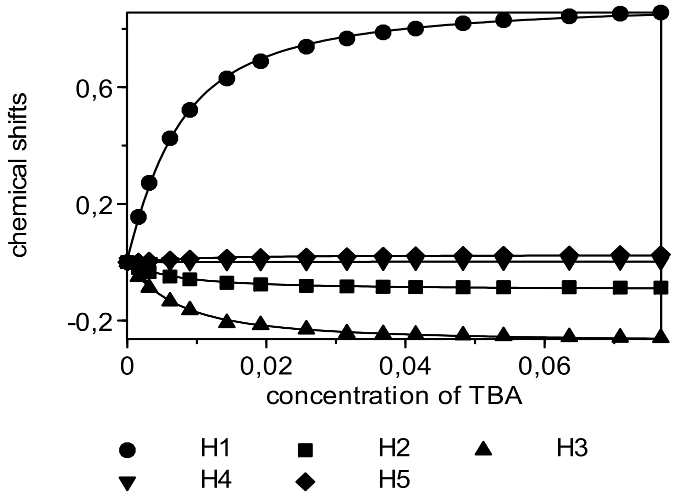

Changes in chemical shifts of the receptor (*R*)-**1** protons upon addition of anion

**K=215 M^{-1}**
C_H=4,76·10^{-3} M
C_G=0,129 M
Range of chemical shift δ [ppm]<br>H1 0,856<br>H2 -0,087<br>H3 -0,259<br>H4 0,003<br>H5 0,026

Eq of anion	
16,11	16
14,88	15
13,35	14
11,36	13
10,14	12
8,71	11
7,72	10
6,63	9
5,41	8
4,03	7
3,00	6
1,89	5
1,29	4
0,66	3
0,33	2
0,00	1

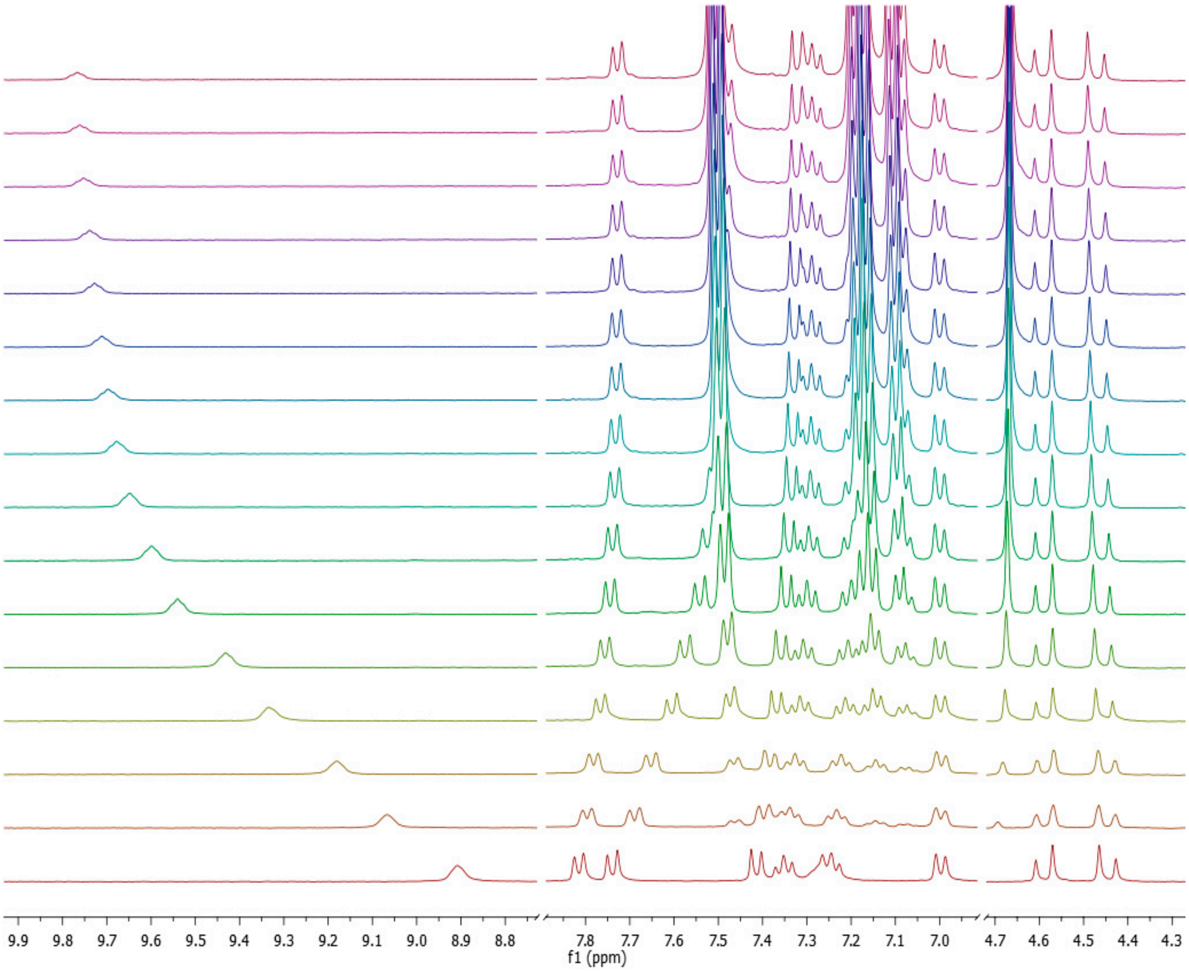

^{1}H NMR spectra changes upon titration of the receptor (*R*)-**1** with anion

Fig. S46 The details of ^{1}H NMR titration experiments of receptor **(*R*)-1** with **(*R*)-11** in acetone-d_6+0,5% H_2O

**Table S19.** Experimental data used to determine binding constant of receptor **(*R*)-1** with **(*R*)-11** in acetone-d_6+0,5% water								
Point	Eq of guest	C_{host} [M]	C_{guest} [M]	Range of chemical shift Δδ [ppm]				
				H1	H2	H3	H4	H5
1	0,00	0,0048	0,0000	0,000	0,000	0,000	0,000	0,000
2	0,33		0,0016	0,155	-0,019	-0,050	-0,002	0,001
3	0,66		0,0031	0,272	-0,034	-0,087	-0,001	0,004
4	1,29		0,0061	0,425	-0,049	-0,134	0,000	0,008
5	1,89		0,0090	0,522	-0,059	-0,164	0,000	0,011
6	3,00		0,0143	0,630	-0,070	-0,208	0,001	0,014
7	4,03		0,0192	0,689	-0,076	-0,215	0,001	0,016
8	5,41		0,0257	0,739	-0,081	-0,230	0,001	0,018
9	6,63		0,0316	0,767	-0,083	-0,243	0,002	0,019
10	7,72		0,0368	0,788	-0,084	-0,246	0,002	0,021
11	8,71		0,0415	0,801	-0,085	-0,248	0,002	0,022
12	10,14		0,0483	0,819	-0,086	-0,250	0,002	0,023
13	11,36		0,0541	0,830	-0,086	-0,253	0,002	0,024
14	13,35		0,0635	0,843	-0,086	-0,256	0,003	0,025
15	14,88		0,0709	0,852	-0,087	-0,258	0,003	0,026
16	16,11		0,0767	0,856	-0,087	-0,259	0,003	0,026

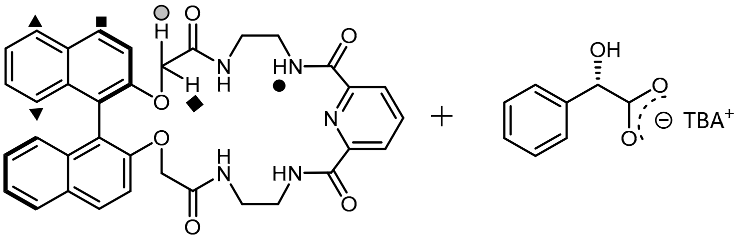

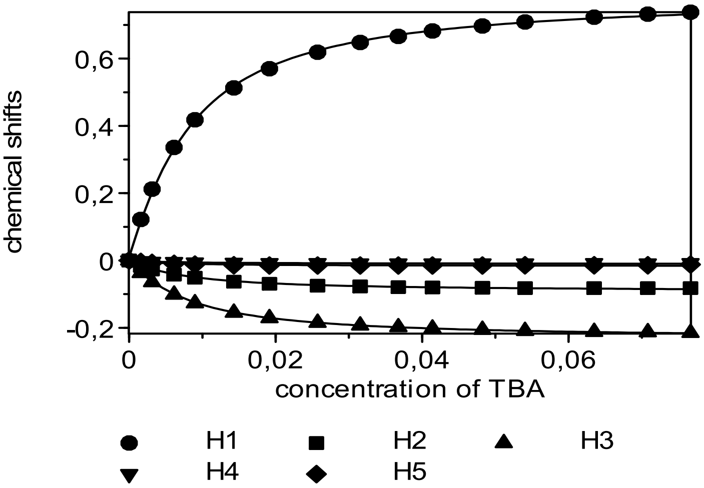

**K=167 M^{-1}**
C_H=4,76·10^{-3} M
C_G=0,129 M
Range of chemical shift δ [ppm]<br>H1 0,738<br>H2 -0,082<br>H3 -0,215<br>H4 -0,008<br>H5 -0,012

Changes in chemical shifts of the receptor (*R*)-**1** protons upon addition of anion

Eq of anion	
16,10	16
14,87	15
13,33	14
11,35	13
10,13	12
8,70	11
7,72	10
6,63	9
5,40	8
4,02	7
3,00	6
1,88	5
1,29	4
0,66	3
0,33	2
0,00	1

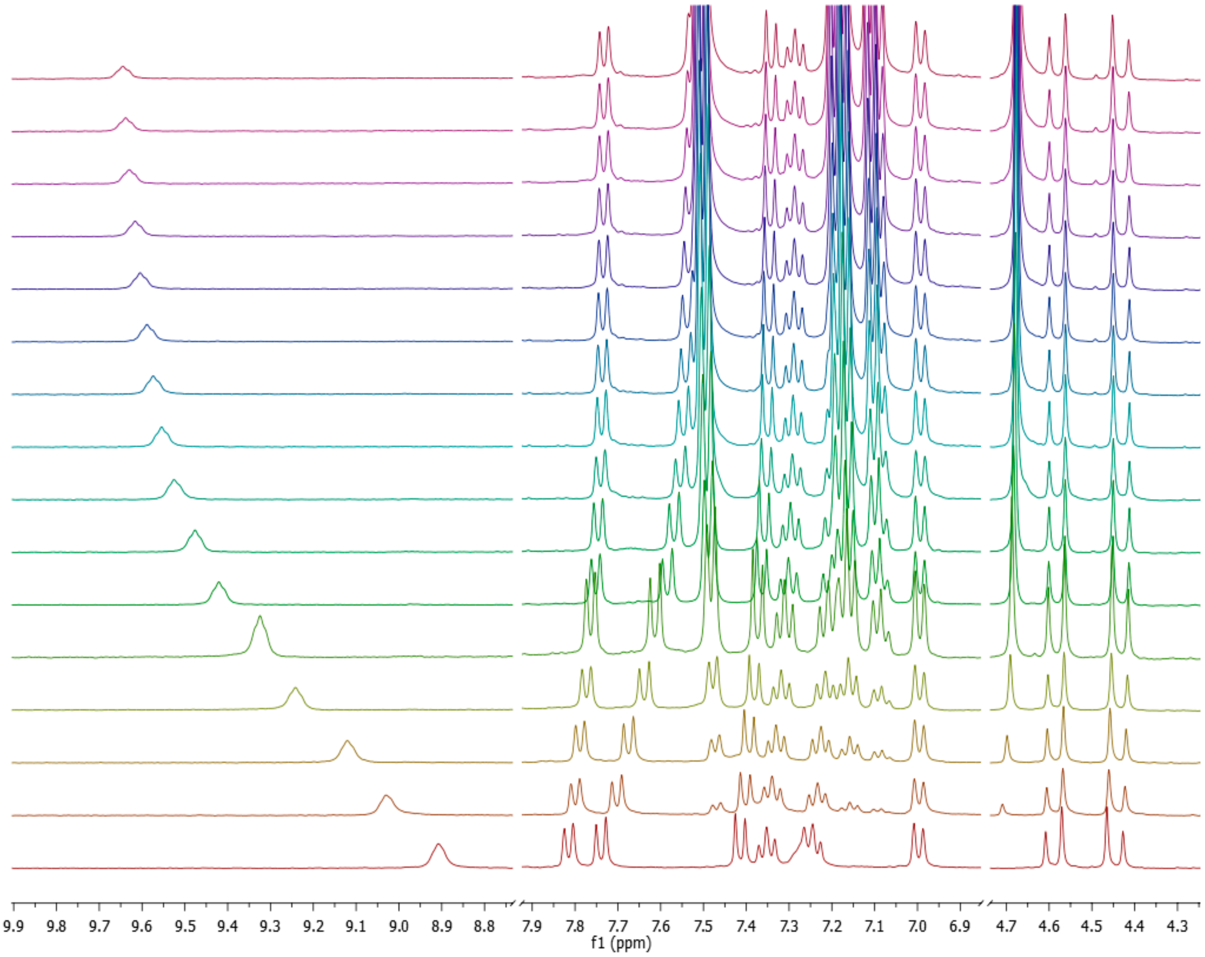

^{1}H NMR spectra changes upon titration of the receptor (*R*)-**1** with anion

Fig. S47 The details of ^{1}H NMR titration experiments of receptor **(*R*)-1** with **(*S*)-11** in acetone-d_6+0,5% H_2O

**Table S20.** Experimental data used to determine binding constant of receptor **(*R*)-1** with **(*S*)-11** in acetone-d_6+0,5% water								
Point	Eq of guest	C_{host} [M]	C_{guest} [M]	Range of chemical shift Δδ [ppm]				
				H1	H2	H3	H4	H5
1	0,00	0,0048	0,0000	0,000	0,000	0,000	0,000	0,000
2	0,33		0,0016	0,122	-0,016	-0,037	-0,002	-0,004
3	0,66		0,0031	0,212	-0,027	-0,064	-0,004	-0,007
4	1,29		0,0061	0,336	-0,042	-0,101	-0,005	-0,010
5	1,88		0,0090	0,418	-0,051	-0,127	-0,006	-0,012
6	3,00		0,0143	0,513	-0,063	-0,155	-0,007	-0,014
7	4,02		0,0192	0,570	-0,069	-0,171	-0,008	-0,015
8	5,40		0,0257	0,619	-0,075	-0,185	-0,008	-0,015
9	6,63		0,0315	0,648	-0,077	-0,193	-0,009	-0,015
10	7,72		0,0367	0,666	-0,079	-0,198	-0,009	-0,015
11	8,70		0,0414	0,682	-0,080	-0,202	-0,009	-0,015
12	10,13		0,0482	0,697	-0,081	-0,206	-0,009	-0,015
13	11,35		0,0541	0,709	-0,082	-0,209	-0,009	-0,014
14	13,33		0,0635	0,723	-0,082	-0,212	-0,008	-0,014
15	14,87		0,0708	0,732	-0,082	-0,213	-0,009	-0,014
16	16,10		0,0766	0,738	-0,082	-0,215	-0,008	-0,012

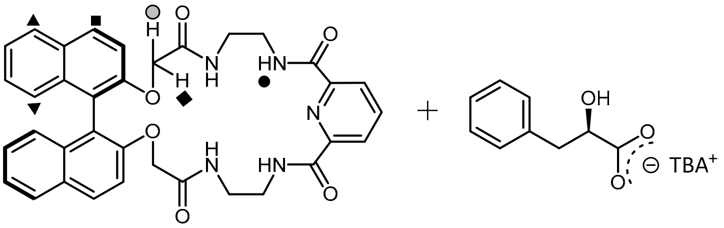

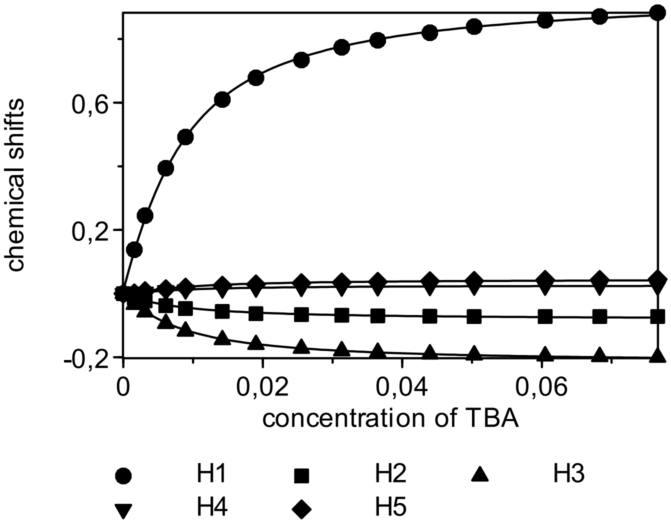

Changes in chemical shifts of the receptor (*R*)-**1** protons upon addition of anion

**K=234 M^{-1}**
C_H=4,89·10^{-3} M
C_G=0,126 M
Range of chemical shift δ [ppm]
H1 1,178<br>H2 -0,078<br>H3 -0,291<br>H4 -0,015<br>H5 0,089

Eq of anion	
15,46	15
13,78	14
12,20	13
10,15	12
8,87	11
7,36	10
6,32	9
5,15	8
3,84	7
2,86	6
1,80	5
1,23	4
0,63	3
0,32	2
0,00	1

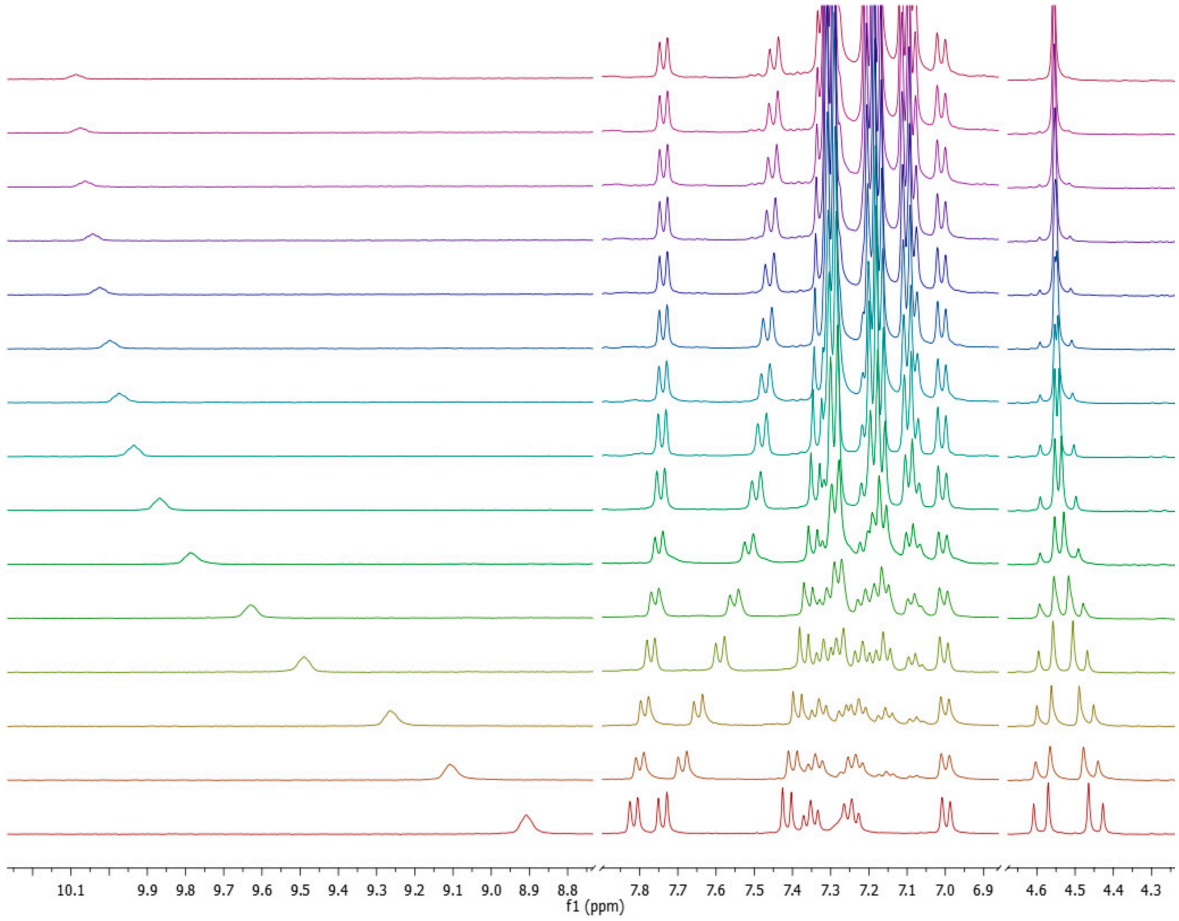

^{1}H NMR spectra changes upon titration of the receptor (*R*)-**1** with anion

Fig. S48 The details of ^{1}H NMR titration experiments of receptor **(*R*)-1** with **D-13** in acetone-d_6+0,5% H_2O

**Table S21.** Experimental data used to determine binding constant of receptor **(*R*)-1** with **D-13** in acetone-d_6+0,5% water								
Point	Eq of guest	C_{host} [M]	C_{guest} [M]	Range of chemical shift Δδ [ppm]				
				H1	H2	H3	H4	H5
1	0,00	0,0049	0,0000	0,000	0,000	0,000	0,000	0,000
2	0,32		0,0016	0,199	-0,016	-0,051	-0,004	0,013
3	0,63		0,0031	0,360	-0,028	-0,093	-0,008	0,025
4	1,23		0,0060	0,584	-0,045	-0,150	-0,012	0,041
5	1,80		0,0088	0,722	-0,055	-0,187	-0,015	0,053
6	2,86		0,0140	0,879	-0,066	-0,227	-0,016	0,065
7	3,84		0,0187	0,961	-0,070	-0,246	-0,027	0,071
8	5,15		0,0252	1,027	-0,074	-0,259	-0,017	0,077
9	6,32		0,0309	1,066	-0,076	-0,269	-0,016	0,080
10	7,36		0,0360	1,090	-0,076	-0,273	-0,016	0,082
11	8,87		0,0433	1,115	-0,077	-0,280	-0,016	0,085
12	10,15		0,0496	1,132	-0,077	-0,284	-0,017	0,087
13	12,20		0,0596	1,154	-0,078	-0,287	-0,015	0,089
14	13,78		0,0673	1,168	-0,078	-0,289	-0,015	0,089
15	15,46		0,0755	1,178	-0,078	-0,291	-0,015	0,089

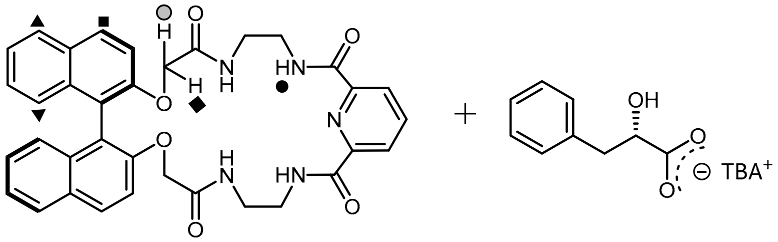

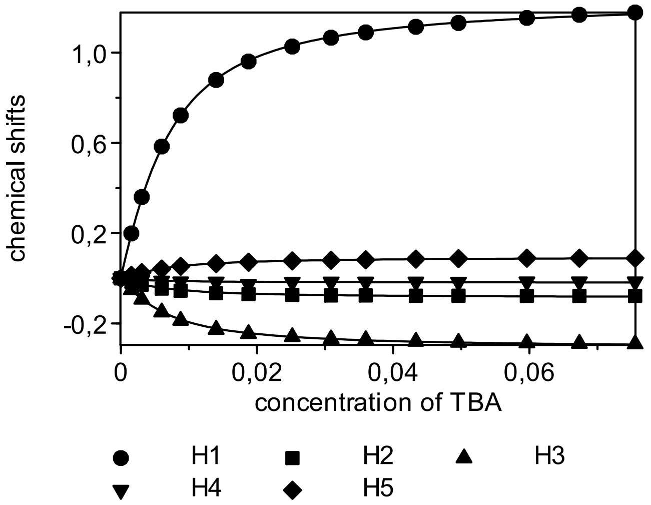

Changes in chemical shifts of the receptor (*R*)-**1** protons upon addition of anion

K=164 M^{-1}

C_H=4,89·10^{-3} M

C_G=0,128 M

Range of chemical shift

δ [ppm]

H1 0,883

H2 -0,072

H3 -0,200

H4 0,024

H5 0,045

Eq of anion	
15,68	15
13,98	14
12,38	13
10,29	12
9,00	11
7,47	10
6,41	9
5,23	8
3,89	7
2,90	6
1,82	5
1,24	4
0,64	3
0,32	2
0,00	1

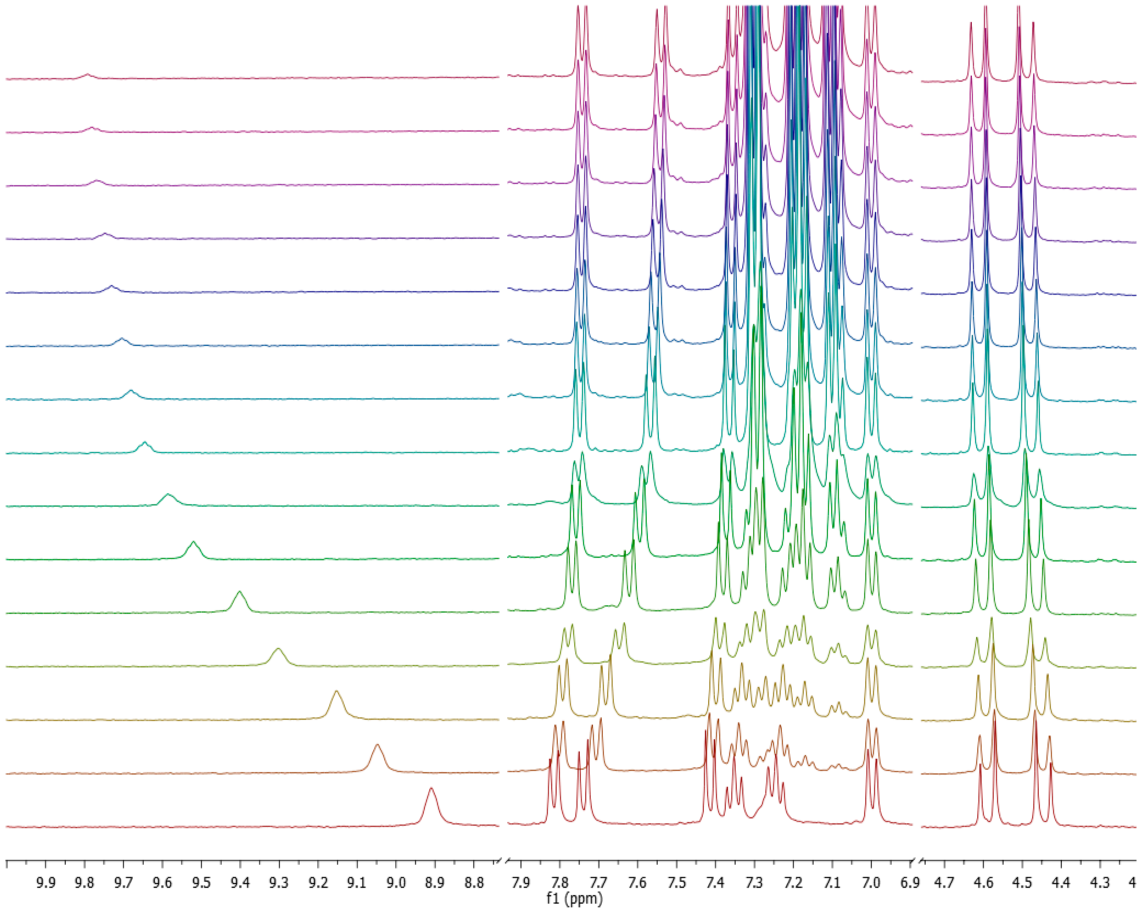

^{1}H NMR spectra changes upon titration of the receptor (*R*)-**1** with anion

Fig. S49 The details of ^{1}H NMR titration experiments of receptor **(*R*)-1** with **L-13** in acetone-d_6+0,5% H_2O

**Table S22.** Experimental data used to determine binding constant of receptor **(*R*)-1** with **L-13** in acetone-d_6+0,5% water								
Point	Eq of guest	C_{host} [M]	C_{guest} [M]	Range of chemical shift Δδ [ppm]				
				H1	H2	H3	H4	H5
1	0,00	0,0049	0,0000	0,000	0,000	0,000	0,000	0,000
2	0,32		0,0016	0,138	-0,014	-0,034	0,000	0,001
3	0,64		0,0031	0,245	-0,023	-0,058	0,005	0,008
4	1,24		0,0061	0,394	-0,038	-0,094	0,009	0,014
5	1,82		0,0089	0,492	-0,047	-0,118	0,012	0,019
6	2,90		0,0142	0,610	-0,056	-0,145	0,015	0,025
7	3,89		0,0190	0,678	-0,063	-0,160	0,018	0,029
8	5,23		0,0255	0,734	-0,066	-0,172	0,020	0,033
9	6,41		0,0313	0,774	-0,068	-0,180	0,021	0,035
10	7,47		0,0365	0,796	-0,070	-0,185	0,022	0,037
11	9,00		0,0440	0,820	-0,071	-0,190	0,023	0,039
12	10,29		0,0503	0,839	-0,072	-0,193	0,024	0,040
13	12,38		0,0605	0,859	-0,072	-0,196	0,024	0,043
14	13,98		0,0683	0,871	-0,072	-0,198	0,024	0,043
15	15,68		0,0766	0,883	-0,072	-0,200	0,024	0,045

2.4. ^{1}H NMR titration data from experiments with macrocyclic compound (*R*)-2

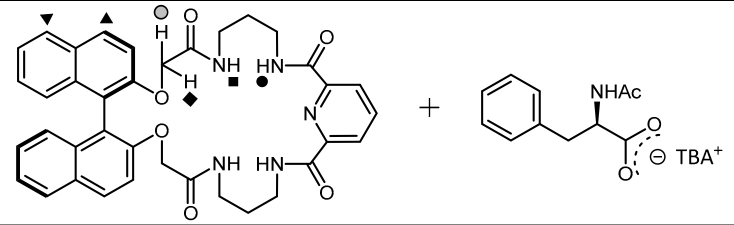

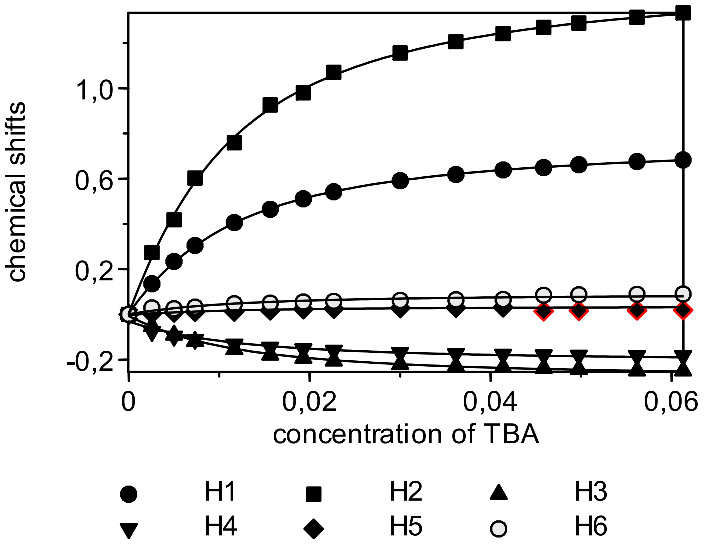

Changes in chemical shifts of the receptor (*R*)-**2** protons upon addition of anion

**K=122 M^{-1}**	
C_H=5,84·10^{-3} M	
C_G=0,105 M	
Range of chemical shift δ [ppm]	
H1	0,683
H2	1,334
H3	-0,249
H4	-0,183
H5	0,019
H6	0,090

Eq of anion	
10,51	15
9,64	14
8,53	13
7,87	12
7,10	11
6,20	10
5,15	9
3,89	8
3,31	7
2,68	6
2,00	5
1,26	4
0,86	3
0,44	2
0,00	1

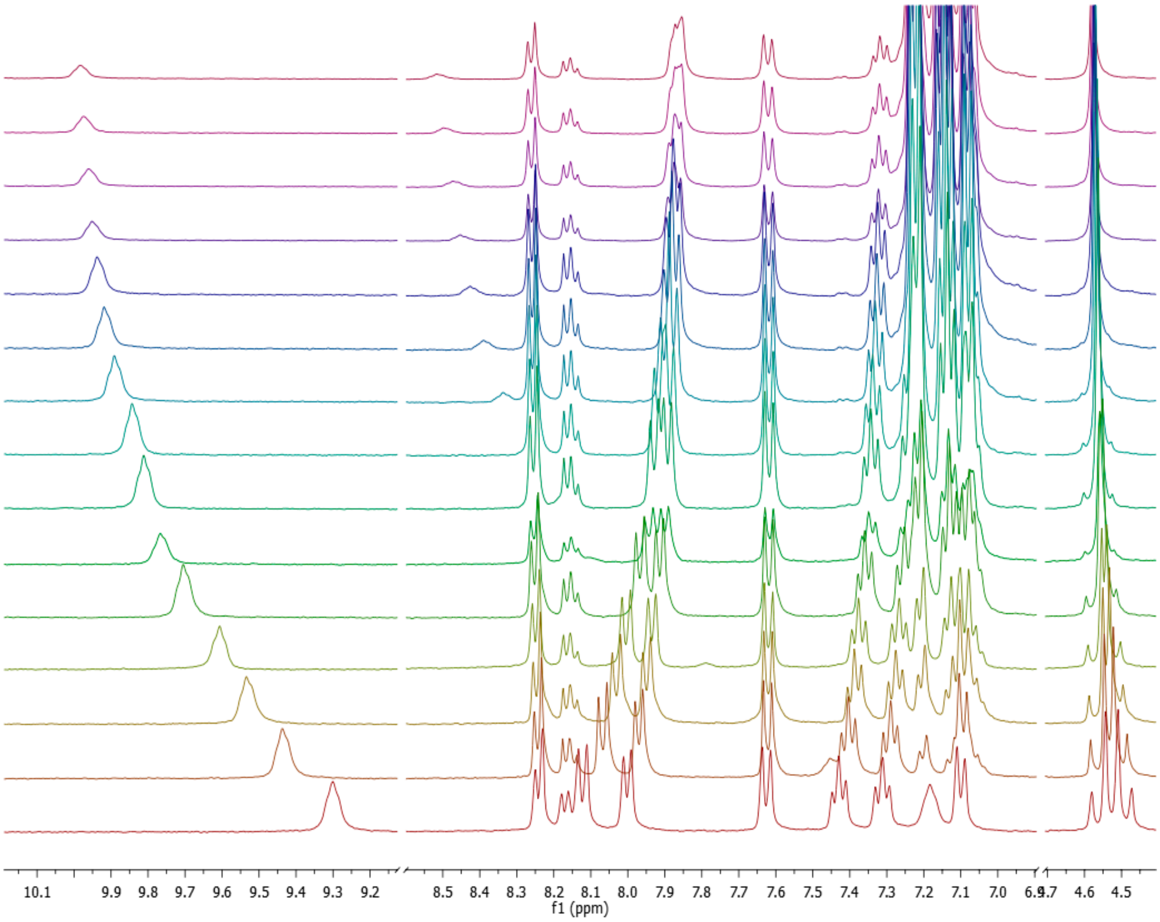

1H NMR spectra changes upon titration of the receptor (*R*)-**2** with anion

Fig. S50 The details of ^{1}H NMR titration experiments of receptor **(*R*)-2** with D-**14** in acetone-d_6+0,5% H_2O

**Table S23.** Experimental data used to determine binding constant of receptor **(*R*)-2** with **D-14** in acetone-d_6+0,5% water									
Point	Eq of guest	C_{host} [M]	C_{guest} [M]	Range of chemical shift Δδ [ppm]					
				H1	H2	H3	H4	H5	H6
1	0,00	0,0058	0,0000	0,000	0,000	0,000	0,000	0,000	0,000
2	0,44		0,0026	0,135	0,274	-0,053	-0,076	0,005	0,029
3	0,86		0,0050	0,234	0,419	-0,091	-0,097	0,009	0,025
4	1,26		0,0073	0,305	0,603	-0,118	-0,111	0,011	0,032
5	2,00		0,0117	0,406	0,759	-0,155	-0,132	0,013	0,047
6	2,68		0,0156	0,465	0,926	-0,179	-0,146	0,018	0,050
7	3,31		0,0193	0,511	0,980	-0,193	-0,153	0,023	0,055
8	3,89		0,0227	0,542	1,071	-0,205	-0,159	0,025	0,058
9	5,15		0,0300	0,591	1,156	-0,221	-0,169	0,028	0,062
10	6,20		0,0362	0,619	1,206	-0,230	-0,174	0,030	0,065
11	7,10		0,0414	0,639	1,242	-0,234	-0,177	0,032	0,066
12	7,87		0,0459	0,649	1,269	-0,238	-0,180	0,014	0,085
13	8,53		0,0498	0,662	1,289	-0,243	-0,181	0,016	0,087
14	9,64		0,0562	0,676	1,314	-0,249	-0,183	0,018	0,089
15	10,51		0,0613	0,683	1,334	-0,249	-0,183	0,019	0,090

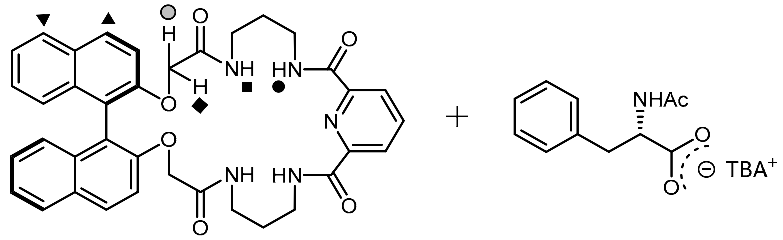

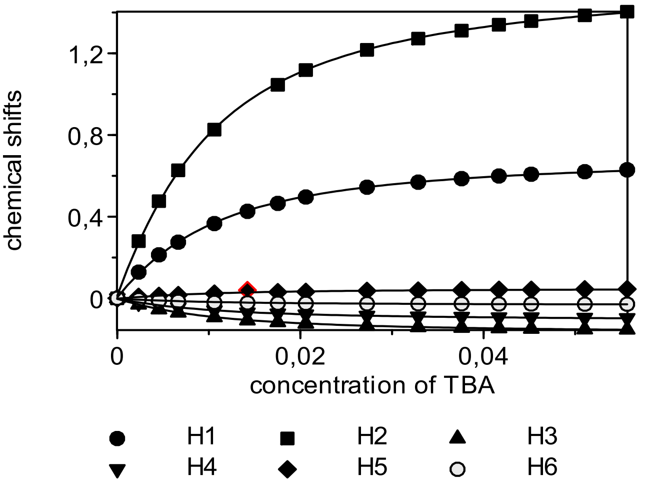

Changes in chemical shifts of the receptor (*R*)-**2** protons upon addition of anion

K=141 M^{-1}

C_H=5,84·10^{-3} M

C_G=0,095 M

Range of chemical shift

δ [ppm]

H1 0,629

H2 1,404

H3 -0,153

H4 -0,097

H5 0,045

H6 -0,029

Eq of anion

9,53	15
8,74	14
7,74	13
7,14	12
6,44	11
5,63	10
4,67	9
3,53	8
3,00	7
2,43	6
1,82	5
1,14	4
0,78	3
0,40	2
0,00	1

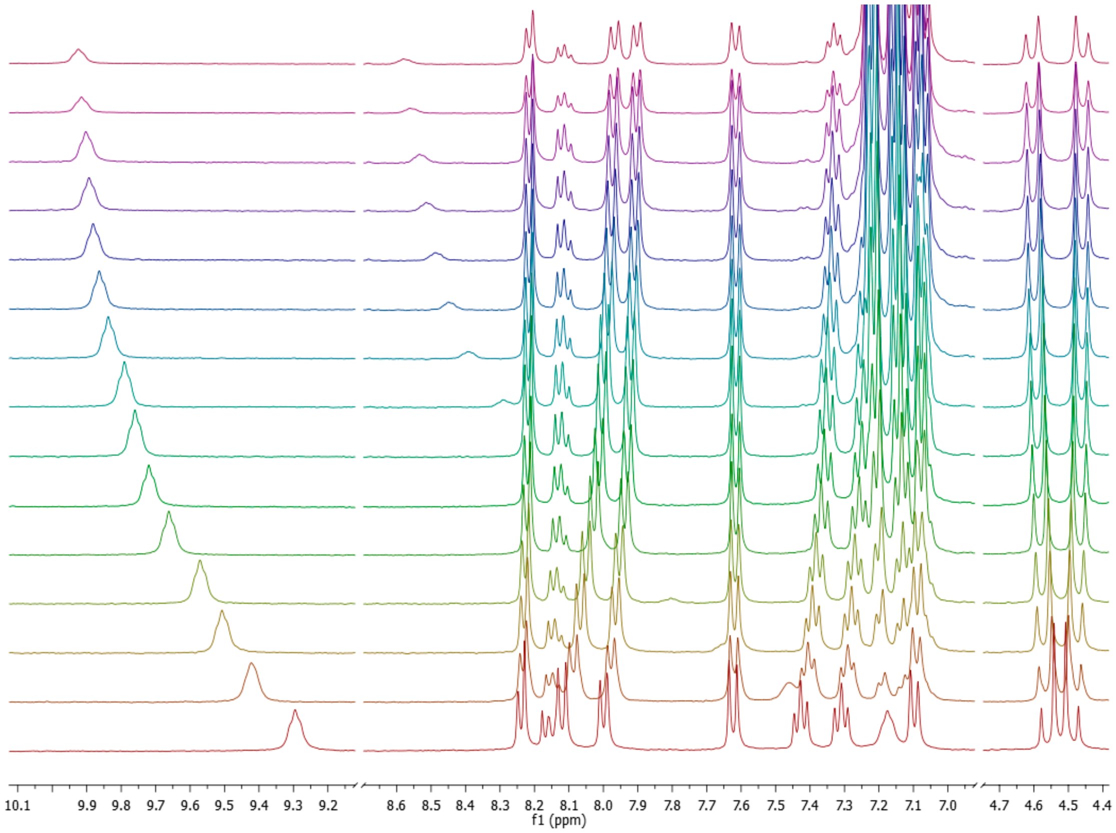

^{1}H NMR spectra changes upon titration of the receptor (*R*)-**2** with anion

Fig. S51 The details of ^{1}H NMR titration experiments of receptor **(*R*)-2** with **L-14** in acetone-d_6+0,5% H_2O

**Table S24.** Experimental data used to determine binding constant of receptor **(*R*)-2** with **L-14** in acetone-d_6+0,5% water									
Point	Eq of guest	C_{host} [M]	C_{guest} [M]	Range of chemical shift Δδ [ppm]					
				H1	H2	H3	H4	H5	H6
1	0,00	0,0058	0,0000	0,000	0,000	0,000	0,000	0,000	0,000
2	0,40		0,0023	0,128	0,280	-0,031	-0,022	0,007	-0,007
3	0,78		0,0045	0,213	0,476	-0,054	-0,034	0,012	-0,012
4	1,14		0,0067	0,275	0,627	-0,070	-0,045	0,016	-0,015
5	1,82		0,0106	0,366	0,826	-0,093	-0,059	0,023	-0,020
6	2,43		0,0142	0,426	x	-0,108	-0,070	0,040	-0,022
7	3,00		0,0175	0,465	1,046	-0,117	-0,075	0,030	-0,024
8	3,53		0,0206	0,496	1,118	-0,124	-0,079	0,032	-0,025
9	4,67		0,0273	0,544	1,217	-0,135	-0,086	0,036	-0,027
10	5,63		0,0329	0,569	1,272	-0,141	-0,090	0,038	-0,027
11	6,44		0,0376	0,586	1,311	-0,144	-0,091	0,040	-0,027
12	7,14		0,0417	0,599	1,340	-0,147	-0,094	0,042	-0,028
13	7,74		0,0452	0,608	1,358	-0,149	-0,094	0,042	-0,028
14	8,74		0,0510	0,620	1,386	-0,152	-0,096	0,044	-0,029
15	9,53		0,0557	0,629	1,404	-0,153	-0,097	0,045	-0,029

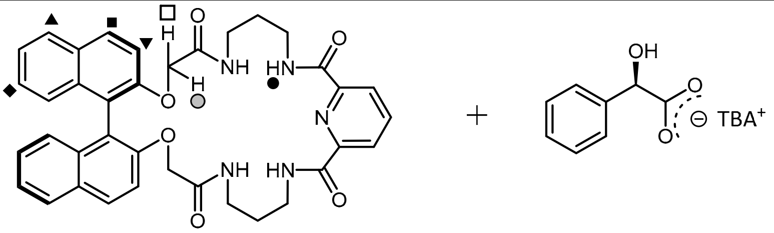

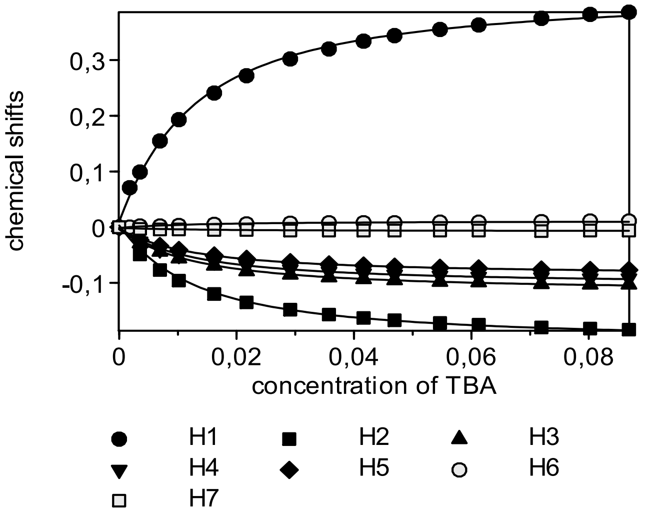

Changes in chemical shifts of the receptor (*R*)-**2** protons upon addition of anion

**K=96 M^{-1}**
C_H=4,36·10^{-3} M
C_G=0,146 M
Range of chemical shift δ [ppm]
H1 0,386
H2 -0,184
H3 -0,103
H4 -0,092
H5 -0,077
H6 0,011
H7 -0,006

Eq of anion	
19,89	16
18,38	15
16,48	14
14,03	13
12,52	12
10,75	11
9,54	10
8,19	9
6,68	8
4,97	7
3,71	6
2,33	5
1,59	4
0,81	3
0,41	2
0,00	1

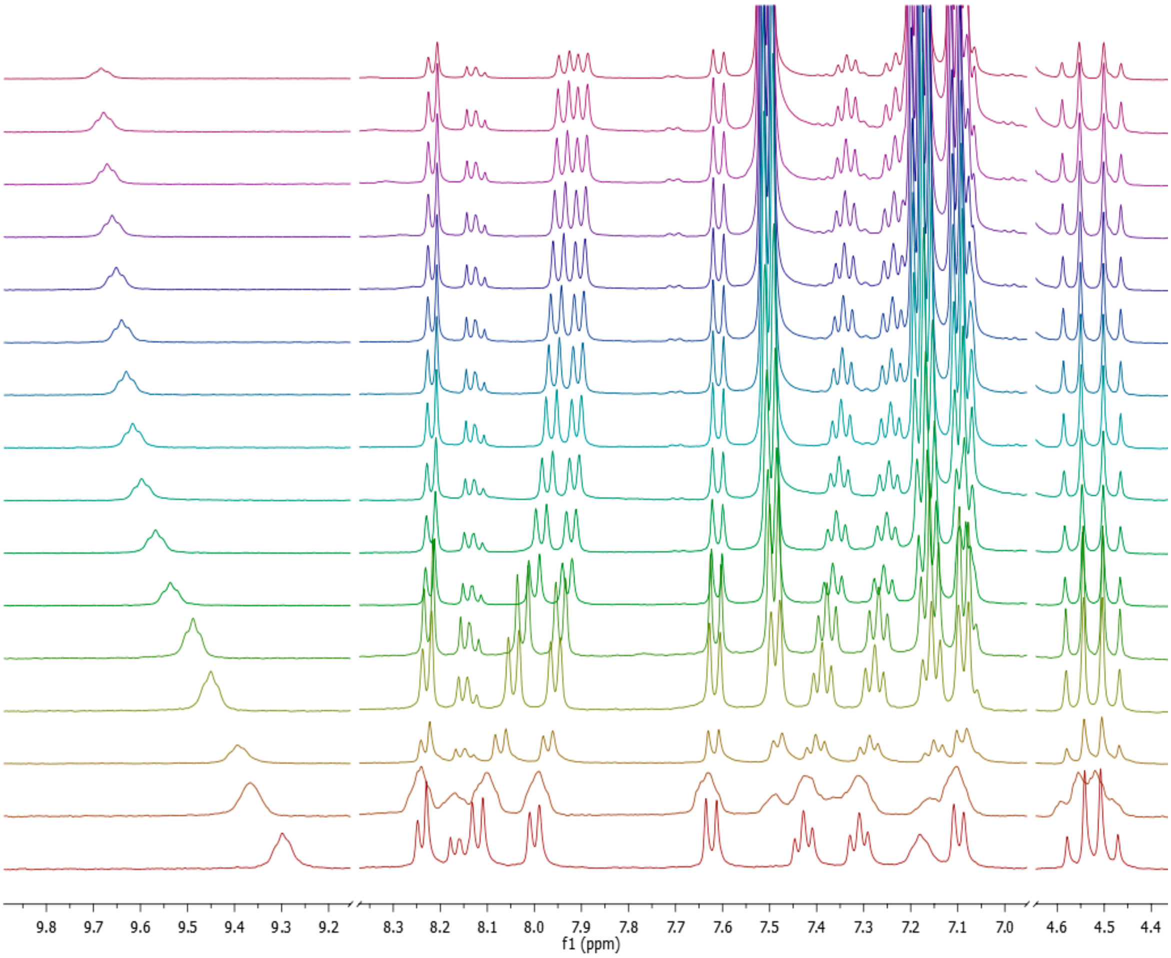

^{1}H NMR spectra changes upon titration of the receptor (*R*)-**2** with anion

Fig. S52 The details of ^{1}H NMR titration experiments of receptor **(*R*)-2** with **(*R*)-11** in acetone-d_6+0,5% H_2O

**Table S25.** Experimental data used to determine binding constant of receptor **(*R*)-2** with （***R***）**-11** in acetone-d_6+0,5% water										
Point	Eq of guest	C_{host} [M]	C_{guest} [M]	Range of chemical shift Δδ [ppm]						
				H1	H2	H3	H4	H5	H6	H7
1	0,00	0,0044	0,0000	0,000	0,000	0,000	0,000	0,000	0,000	0,000
2	0,41		0,0018	0,071	x	x	x	x	x	x
3	0,81		0,0036	0,099	-0,049	-0,029	-0,026	-0,023	0,002	-0,003
4	1,59		0,0069	0,155	-0,077	-0,044	-0,040	-0,034	0,002	-0,004
5	2,33		0,0102	0,193	-0,096	-0,056	-0,050	-0,041	0,003	-0,004
6	3,71		0,0162	0,241	-0,120	-0,069	-0,062	-0,051	0,005	-0,005
7	4,97		0,0217	0,272	-0,135	-0,079	-0,069	-0,057	0,006	-0,005
8	6,68		0,0291	0,302	-0,148	-0,085	-0,076	-0,063	0,007	-0,006
9	8,19		0,0357	0,320	-0,157	-0,090	-0,080	-0,067	0,008	-0,006
10	9,54		0,0416	0,334	-0,163	-0,093	-0,083	-0,069	0,008	-0,006
11	10,75		0,0469	0,344	-0,167	-0,095	-0,085	-0,071	0,008	-0,006
12	12,52		0,0546	0,355	-0,172	-0,098	-0,087	-0,073	0,009	-0,006
13	14,03		0,0612	0,363	-0,175	-0,099	-0,089	-0,074	0,009	-0,006
14	16,48		0,0719	0,375	-0,180	-0,102	-0,090	-0,076	0,010	-0,007
15	18,38		0,0802	0,382	-0,182	-0,103	-0,092	-0,077	0,011	-0,006
16	19,89		0,0868	0,386	-0,184	-0,103	-0,092	-0,077	0,011	-0,006

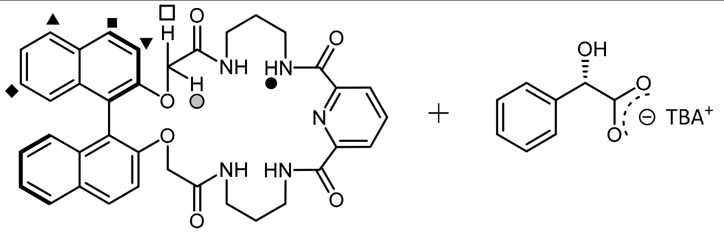

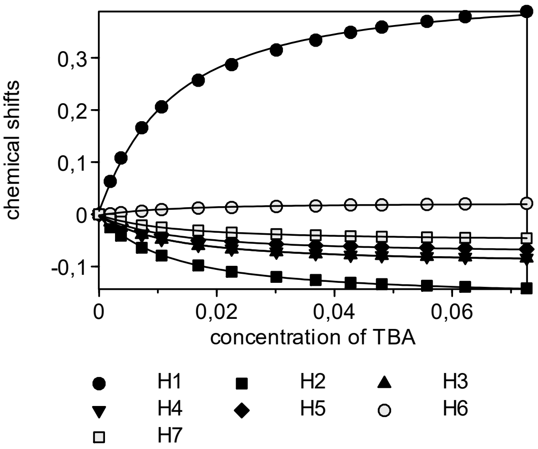

Changes in chemical shifts of the receptor (*R*)-**2** protons upon addition of anion

K=101 M^{-1}

C_H=4,36·10^{-3} M

C_G=0,142 M

Range of chemical shift
δ [ppm]

H1	0,389
H2	-0,142
H3	-0,084
H4	-0,084
H5	-0,067
H6	0,021
H7	-0,046

Eq of anion

19,94	16
18,48	15
16,65	14
14,25	13
12,76	12
11,01	11
9,79	10
8,43	9
6,90	8
5,16	7
3,86	6
2,43	5
1,66	4
0,85	3
0,43	2
0,00	1

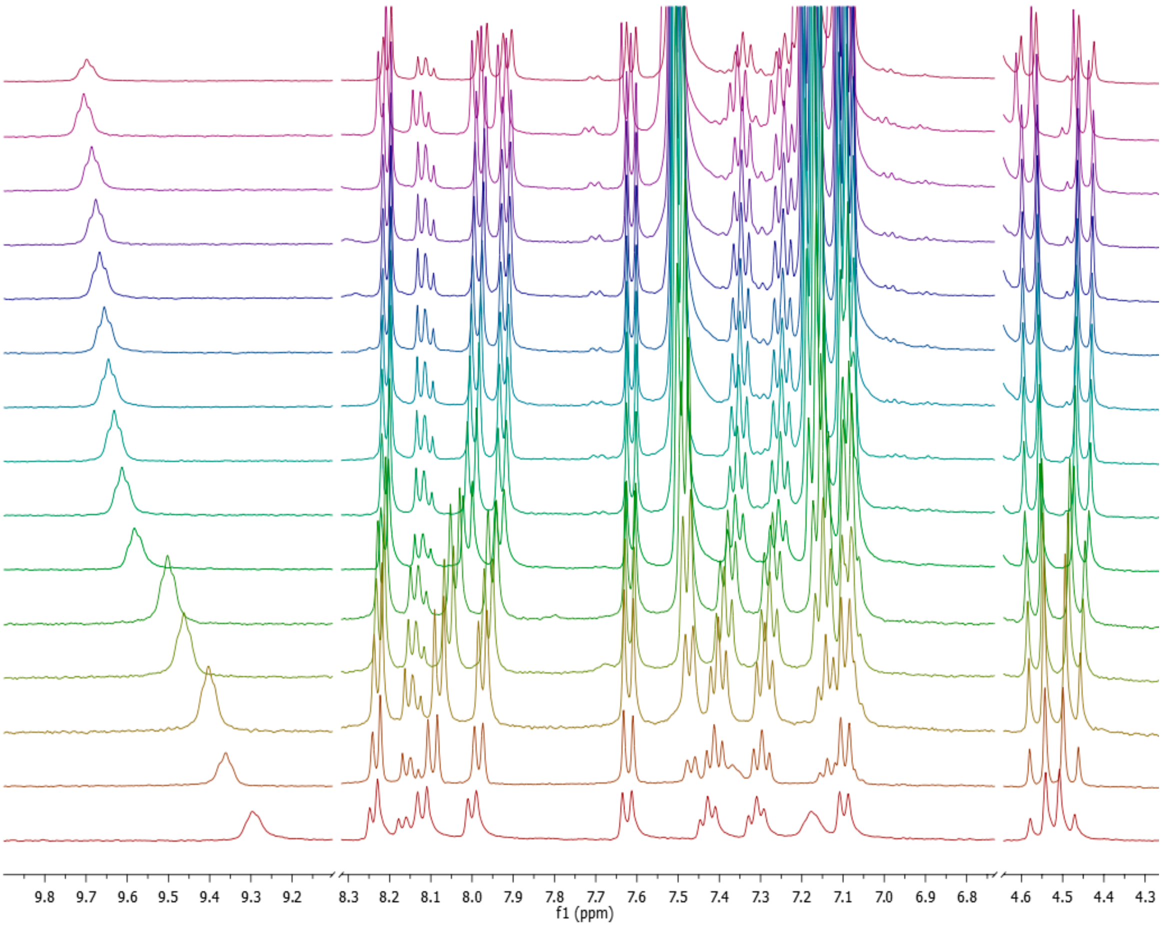

^{1}H NMR spectra changes upon titration of the receptor (*R*)-**2** with anion

Fig. S53 The details of ^{1}H NMR titration experiments of receptor **(*R*)-2** with **(*R*)-11** in acetone-d_6+0,5% H_2O

**Table S26.** Experimental data used to determine binding constant of receptor **(*R*)-2** with **(*S*) -11** in acetone-d_6+0,5% water										
Point	Eq of guest	C_{host} [M]	C_{guest} [M]	Range of chemical shift Δδ [ppm]						
				H1	H2	H3	H4	H5	H6	H7
1	0,00	0,0044	0,0000	0,000	0,000	0,000	0,000	0,000	0,000	0,000
2	0,43		0,0019	0,063	-0,025	-0,015	-0,015	-0,012	0,001	-0,009
3	0,85		0,0037	0,108	-0,041	-0,025	-0,026	-0,021	0,003	-0,014
4	1,66		0,0073	0,166	-0,064	-0,039	-0,040	-0,031	0,006	-0,021
5	2,43		0,0106	0,206	-0,079	-0,048	-0,048	-0,039	0,009	-0,025
6	3,86		0,0169	0,257	-0,098	-0,060	-0,059	-0,050	0,012	-0,031
7	5,16		0,0225	0,287	-0,110	-0,067	-0,066	-0,052	0,013	-0,035
8	6,90		0,0301	0,315	-0,120	-0,072	-0,072	-0,057	0,015	-0,038
9	8,43		0,0368	0,334	-0,126	-0,076	-0,075	-0,060	0,016	-0,040
10	9,79		0,0427	0,349	-0,131	-0,078	-0,078	-0,061	0,018	-0,042
11	11,01		0,0480	0,359	-0,134	-0,079	-0,079	-0,063	0,018	-0,043
12	12,76		0,0557	0,370	-0,137	-0,081	-0,081	-0,064	0,019	-0,044
13	14,25		0,0622	0,379	-0,139	-0,082	-0,082	-0,065	0,020	-0,045
14	16,65		0,0727	0,389	-0,142	-0,084	-0,084	-0,067	0,021	-0,046
15	18,48		0,0807	0,410	-0,132	-0,072	-0,072	-0,055	0,034	-0,034
16	19,94		0,0870	0,403	-0,145	-0,085	-0,085	-0,068	0,022	-0,047

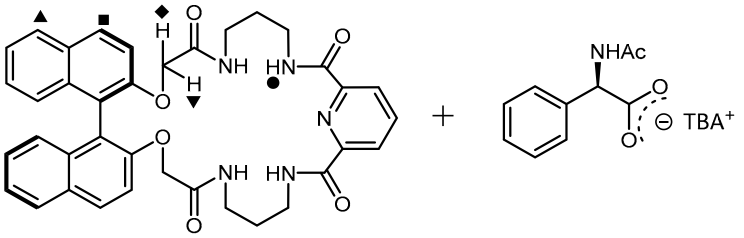

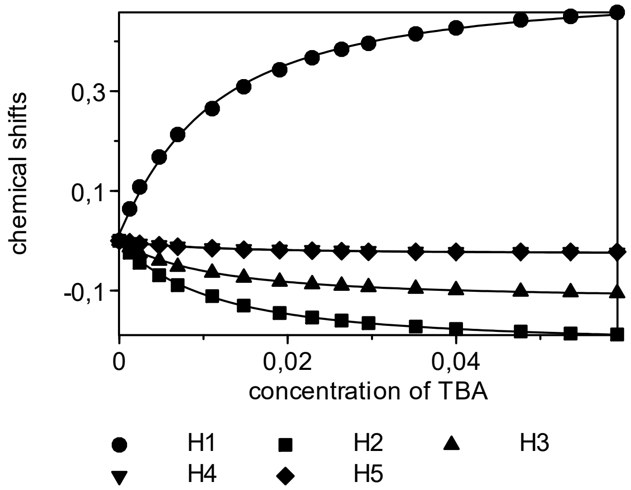

Changes in chemical shifts of the receptor (*R*)-**2** protons upon addition of anion

**K=124 M^{-1}**
C_H=4,89·10^{-3} M
C_G=0,099 M
Range of chemical shift δ[ppm]<br>H1 0,458<br>H2 -0,188<br>H3 -0,105<br>H4 -0,024<br>H5 -0,022

Eq of anion	
12,10	16
10,97	15
9,76	14
8,18	13
7,21	12
6,06	11
5,40	10
4,69	9
3,90	8
3,02	7
2,26	6
1,42	5
0,97	4
0,50	3
0,25	2
0,00	1

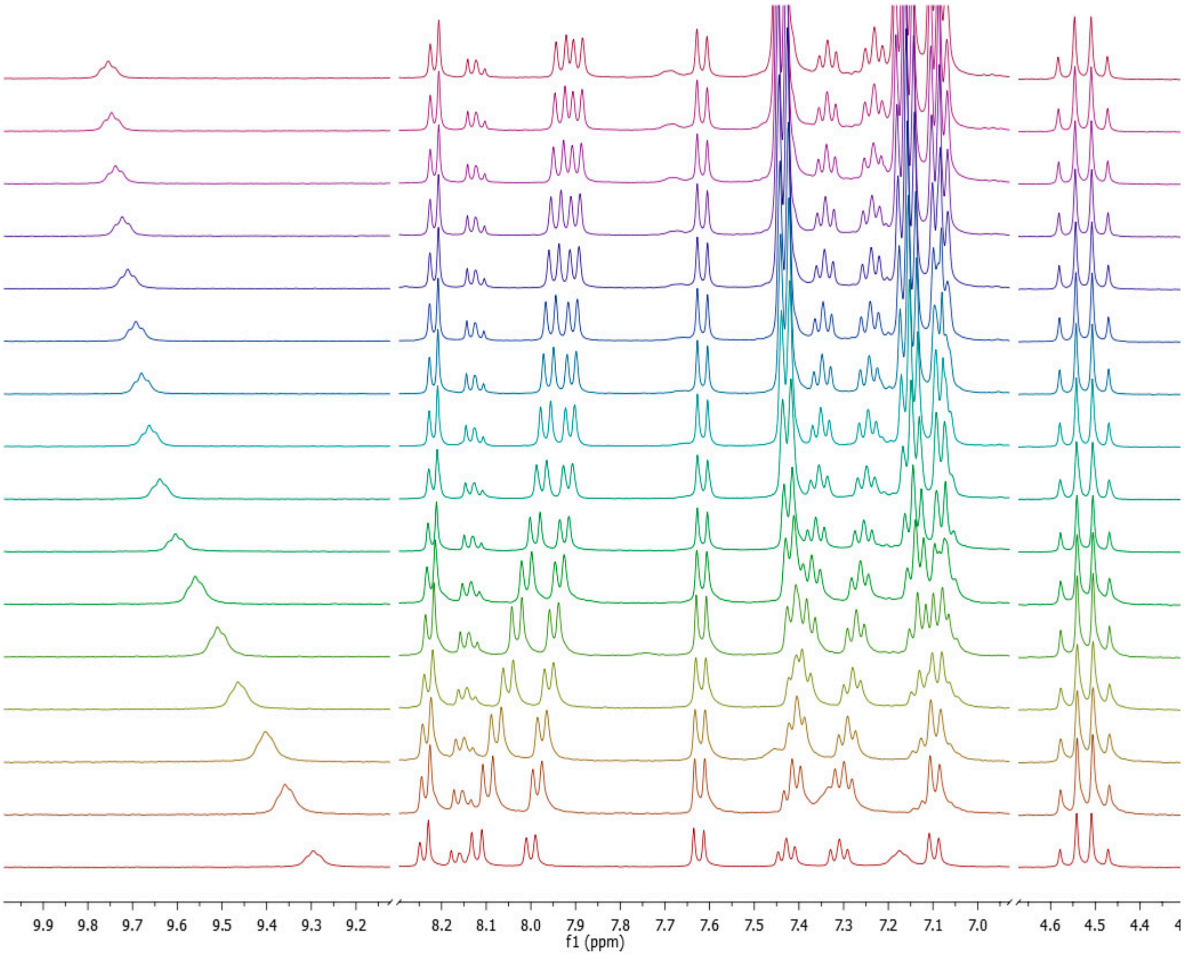

^{1}H NMR spectra changes upon titration of the receptor (*R*)-**2** with anion

Fig. S54 The details of ^{1}H NMR titration experiments of receptor **(*R*)-2** with **D-12** in acetone-d_6+0,5% H_2O

Table S27. Experimental data used to determine binding constant of receptor **(*R*)-2** with **D-12** in acetone-d_6+0,5% water

Point	Eq of guest	C_{host} [M]	C_{guest} [M]	Range of chemical shift Δδ [ppm]				
				H1	H2	H3	H4	H5
1	0,00	0,0049	0,0000	0,000	0,000	0,000	0,000	0,000
2	0,25		0,0012	0,064	-0,024	-0,013	-0,003	-0,003
3	0,50		0,0024	0,108	-0,044	-0,025	-0,006	-0,006
4	0,97		0,0047	0,168	-0,069	-0,040	-0,009	-0,009
5	1,42		0,0069	0,213	-0,089	-0,052	-0,012	-0,012
6	2,26		0,0110	0,265	-0,111	-0,064	-0,015	-0,015
7	3,02		0,0148	0,309	-0,130	-0,074	-0,017	-0,018
8	3,90		0,0190	0,343	-0,145	-0,082	-0,018	-0,019
9	4,69		0,0229	0,367	-0,154	-0,087	-0,019	-0,020
10	5,40		0,0264	0,384	-0,160	-0,090	-0,020	-0,021
11	6,06		0,0296	0,396	-0,165	-0,093	-0,021	-0,022
12	7,21		0,0352	0,415	-0,172	-0,096	-0,022	-0,022
13	8,18		0,0400	0,427	-0,177	-0,099	-0,023	-0,022
14	9,76		0,0477	0,443	-0,182	-0,102	-0,023	-0,022
15	10,97		0,0536	0,450	-0,186	-0,103	-0,023	-0,022
16	12,10		0,0591	0,458	-0,188	-0,105	-0,024	-0,022

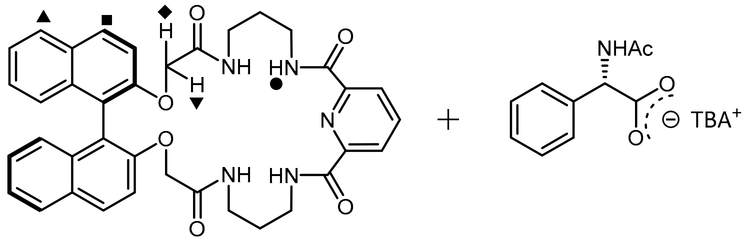

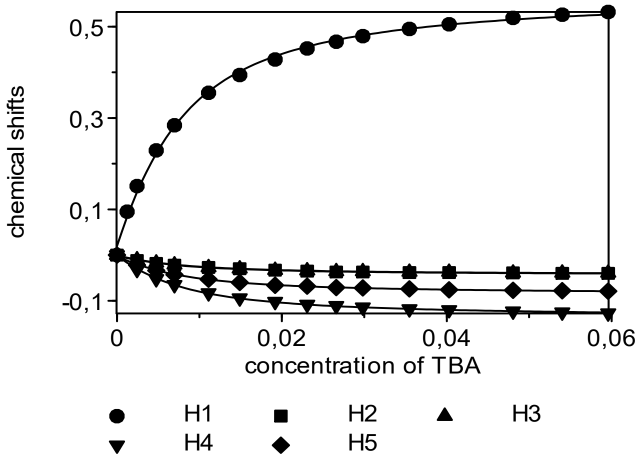

Changes in chemical shifts of the receptor (R)-**2** protons upon addition of anion

K=192 M^{-1}

C_H=4,89·10^{-3} M

C_G=0,099 M

Range of chemical shift
δ [ppm]
H1 0,532
H2 -0,040
H3 -0,039
H4 -0,127
H5 -0,079

Eq of anion	
12,20	16
11,06	15
9,83	14
8,25	13
7,26	12
6,10	11
5,44	10
4,72	9
3,93	8
3,05	7
2,27	6
1,43	5
0,97	4
0,50	3
0,25	2
0,00	1

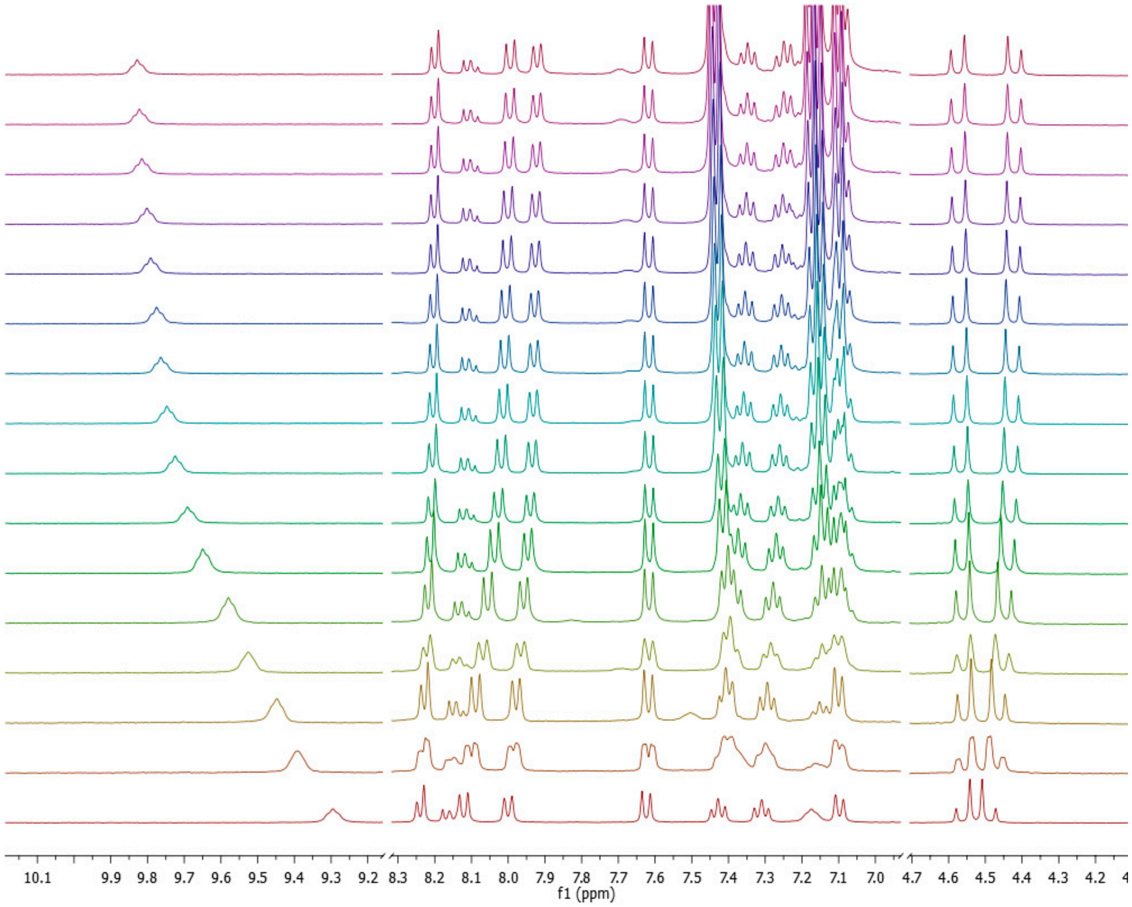

^{1}H NMR spectra changes upon titration of the receptor (R)-**2** with anion

Fig. S55 The details of ^{1}H NMR titration experiments of receptor **(*R*)-2** with **L-12** in acetone-d_6+0,5% H_2O

**Table S28.** Experimental data used to determine binding constant of receptor **(*R*)-2** with **L-12** in acetone-d$_6$+0,5% water								
Point	Eq of guest	C_{host} [M]	C_{guest} [M]	Range of chemical shift Δδ [ppm]				
				H1	H2	H3	H4	H5
1	0,00	0,0049	0,0000	0,000	0,000	0,000	0,000	0,000
2	0,25		0,0012	0,095	x	x	x	x
3	0,50		0,0024	0,151	-0,012	-0,010	-0,032	-0,022
4	0,97		0,0048	0,229	-0,018	-0,017	-0,052	-0,034
5	1,43		0,0070	0,284	-0,022	-0,021	-0,065	-0,043
6	2,27		0,0111	0,355	-0,027	-0,027	-0,083	-0,053
7	3,05		0,0149	0,394	-0,030	-0,030	-0,094	-0,060
8	3,93		0,0192	0,428	-0,033	-0,033	-0,102	-0,066
9	4,72		0,0231	0,452	-0,035	-0,034	-0,108	-0,068
10	5,44		0,0266	0,467	-0,037	-0,036	-0,111	-0,071
11	6,10		0,0298	0,479	-0,037	-0,036	-0,114	-0,072
12	7,26		0,0355	0,495	-0,038	-0,037	-0,118	-0,074
13	8,25		0,0403	0,505	-0,038	-0,037	-0,120	-0,076
14	9,83		0,0480	0,519	-0,039	-0,039	-0,123	-0,077
15	11,06		0,0540	0,526	-0,040	-0,039	-0,125	-0,078
16	12,20		0,0596	0,532	-0,040	-0,039	-0,127	-0,079

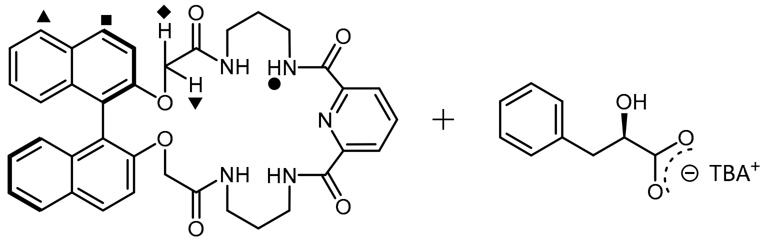

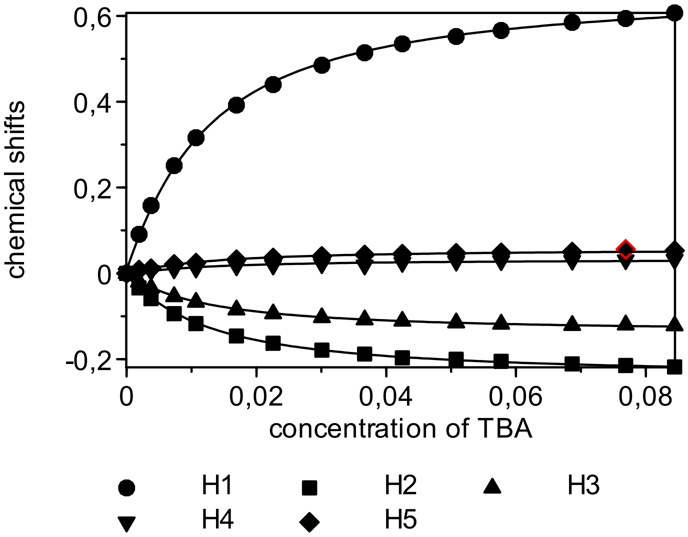

Changes in chemical shifts of the receptor (*R*)-**2** protons upon addition of anion

**K=96 M^{-1}**
C_H=4,67·10^{-3} M
C_G=0,135 M
Range of chemical shift δ [ppm]<br>H1 0,607<br>H2 -0,218<br>H3 -0,122<br>H4 0,032<br>H5 0,053

Eq of anion	
18,07	15
16,45	14
14,69	13
12,35	12
10,86	11
9,09	10
7,85	9
6,44	8
4,83	7
3,62	6
2,29	5
1,57	4
0,80	3
0,41	2
0,00	1

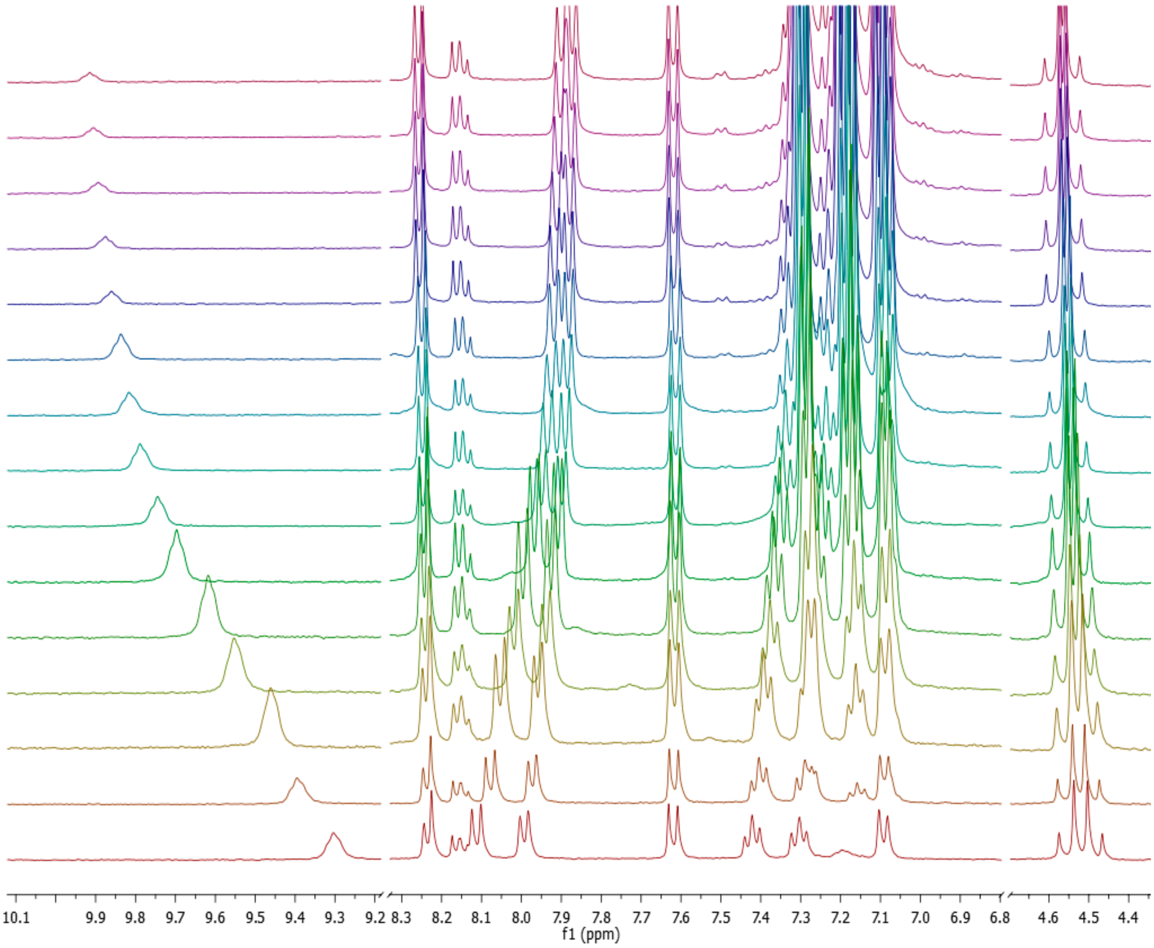

^{1}H NMR spectra changes upon titration of the receptor (*R*)-**2** with anion

Fig. S56 The details of ^{1}H NMR titration experiments of receptor **(*R*)-2** with **D-13** in acetone-d_6+0,5% H_2O

**Table S29.** Experimental data used to determine binding constant of receptor **(*R*)-2** with **D-13** in acetone-d_6+0,5% water								
Point	Eq of guest	C_{host} [M]	C_{guest} [M]	Range of chemical shift Δδ [ppm]				
				H1	H2	H3	H4	H5
1	0,00	0,0047	0,0000	0,000	0,000	0,000	0,000	0,000
2	0,41		0,0019	0,091	-0,034	-0,020	0,003	0,008
3	0,80		0,0038	0,158	-0,059	-0,034	0,006	0,011
4	1,57		0,0073	0,251	-0,094	-0,054	0,010	0,021
5	2,29		0,0107	0,316	-0,117	-0,067	0,012	0,024
6	3,62		0,0169	0,392	-0,146	-0,085	0,017	0,032
7	4,83		0,0226	0,440	-0,163	-0,094	0,019	0,036
8	6,44		0,0301	0,485	-0,179	-0,103	0,022	0,040
9	7,85		0,0367	0,514	-0,188	-0,108	0,019	0,043
10	9,09		0,0425	0,535	-0,197	-0,111	0,025	0,045
11	10,86		0,0508	0,552	-0,200	-0,115	0,028	0,047
12	12,35		0,0577	0,566	-0,205	-0,118	0,029	0,048
13	14,69		0,0687	0,585	-0,211	-0,121	0,030	0,050
14	16,45		0,0769	0,594	-0,215	-0,120	0,031	0,066
15	18,07		0,0844	0,607	-0,218	-0,122	0,032	0,053

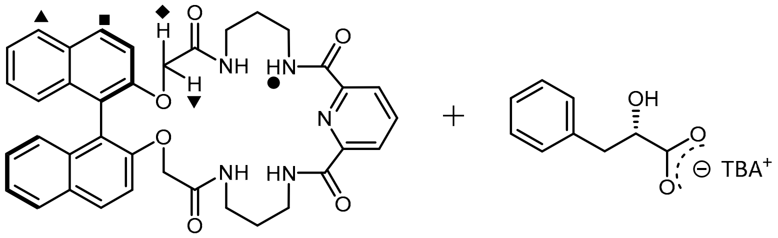

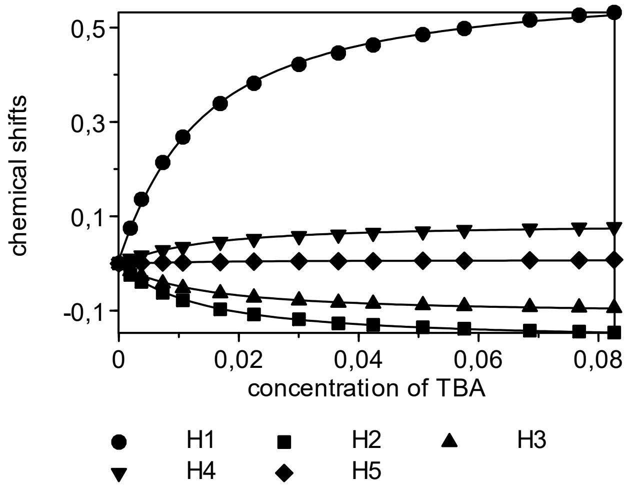

Changes in chemical shifts of the receptor (*R*)-**2** protons upon addition of anion

K=91 M^{-1}

C_H=4,67·10^{-3} M

C_G=0,135 M

Range of chemical shift δ [ppm]

H1 0,532

H2 -0,146

H3 -0,094

H4 0,077

H5 0,008

Eq of anion	
17,68	15
16,43	14
14,67	13
12,33	12
10,85	11
9,07	10
7,83	9
6,43	8
4,82	7
3,62	6
2,28	5
1,56	4
0,80	3
0,41	2
0,00	1

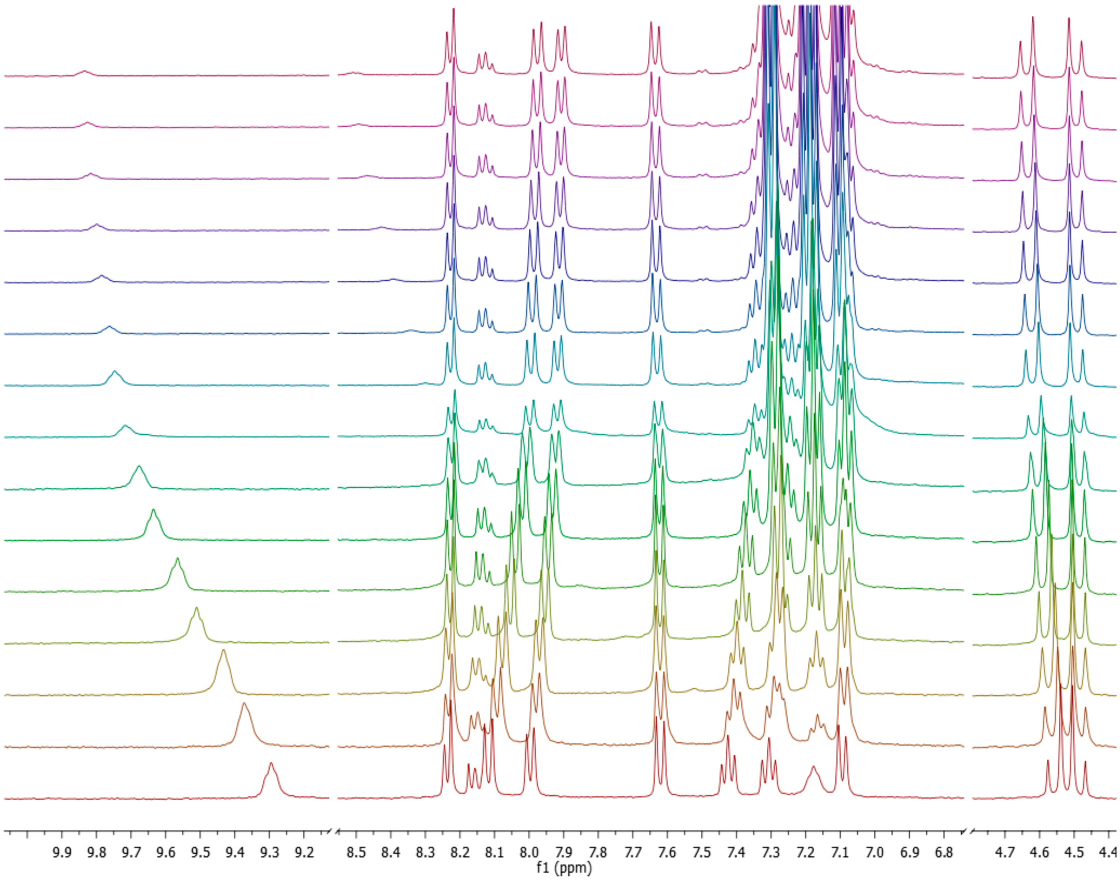

^{1}H NMR spectra changes upon titration of the receptor (*R*)-**2** with anion

Fig. S57 The details of ^{1}H NMR titration experiments of receptor **(*R*)-2** with **L-12** in acetone-*d*$_6$+0,5% H_2O

**Table S30.** Experimental data used to determine binding constant of receptor **(*R*)-2** with **L-13** in acetone-d_6+0,5% water								
Point	Eq of guest	C_{host} [M]	C_{guest} [M]	Range of chemical shift Δδ [ppm]				
				H1	H2	H3	H4	H5
1	0,00	0,0047	0,0000	0,000	0,000	0,000	0,000	0,000
2	0,41		0,0019	0,075	-0,024	-0,016	0,010	0,000
3	0,80		0,0038	0,136	-0,039	-0,026	0,017	0,001
4	1,56		0,0073	0,214	-0,062	-0,042	0,028	0,002
5	2,28		0,0107	0,268	-0,078	-0,052	0,035	0,002
6	3,62		0,0169	0,339	-0,097	-0,063	0,046	0,003
7	4,82		0,0225	0,382	-0,108	-0,072	0,052	0,004
8	6,43		0,0300	0,422	-0,118	-0,078	0,058	0,005
9	7,83		0,0366	0,446	-0,127	-0,083	0,061	0,005
10	9,07		0,0424	0,463	-0,130	-0,085	0,065	0,005
11	10,85		0,0507	0,485	-0,135	-0,088	0,068	0,006
12	12,33		0,0576	0,498	-0,138	-0,090	0,071	0,006
13	14,67		0,0686	0,516	-0,142	-0,092	0,074	0,007
14	16,43		0,0768	0,526	-0,144	-0,093	0,076	0,007
15	17,68		0,0826	0,532	-0,146	-0,094	0,077	0,008

2.5. ^{1}H NMR titration data from experiments with macrocyclic compound (*R*)-3

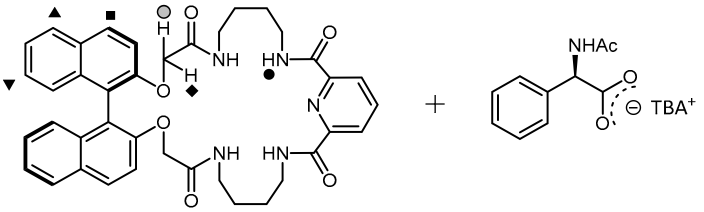

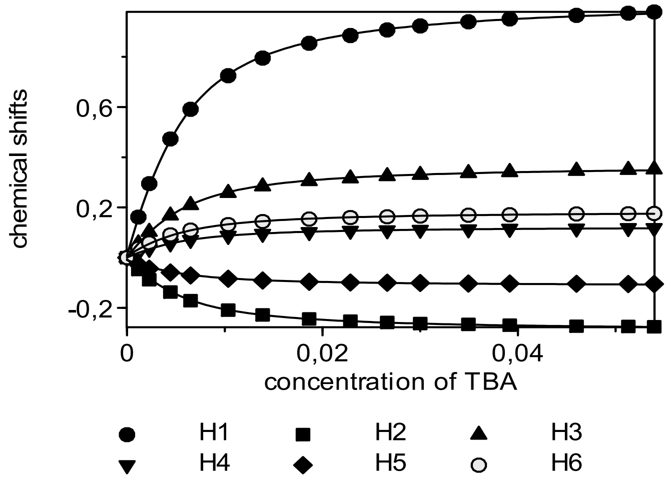

Changes in chemical shifts of the receptor (*R*)-**3** protons upon addition of anion

K=329 M^{-1}

C_H=4,38·10^{-3} M

C_G=0,093 M

Range of chemical shift δ[ppm]

H1 0,979

H2 -0,275

H3 0,352

H4 0,118

H5 -0,105

H6 0,176

Eq of anion	
12,32	16
11,72	15
10,50	14
8,94	13
7,98	12
6,85	11
6,08	10
5,22	9
4,26	8
3,17	7
2,36	6
1,48	5
1,01	4
0,52	3
0,26	2
0,00	1

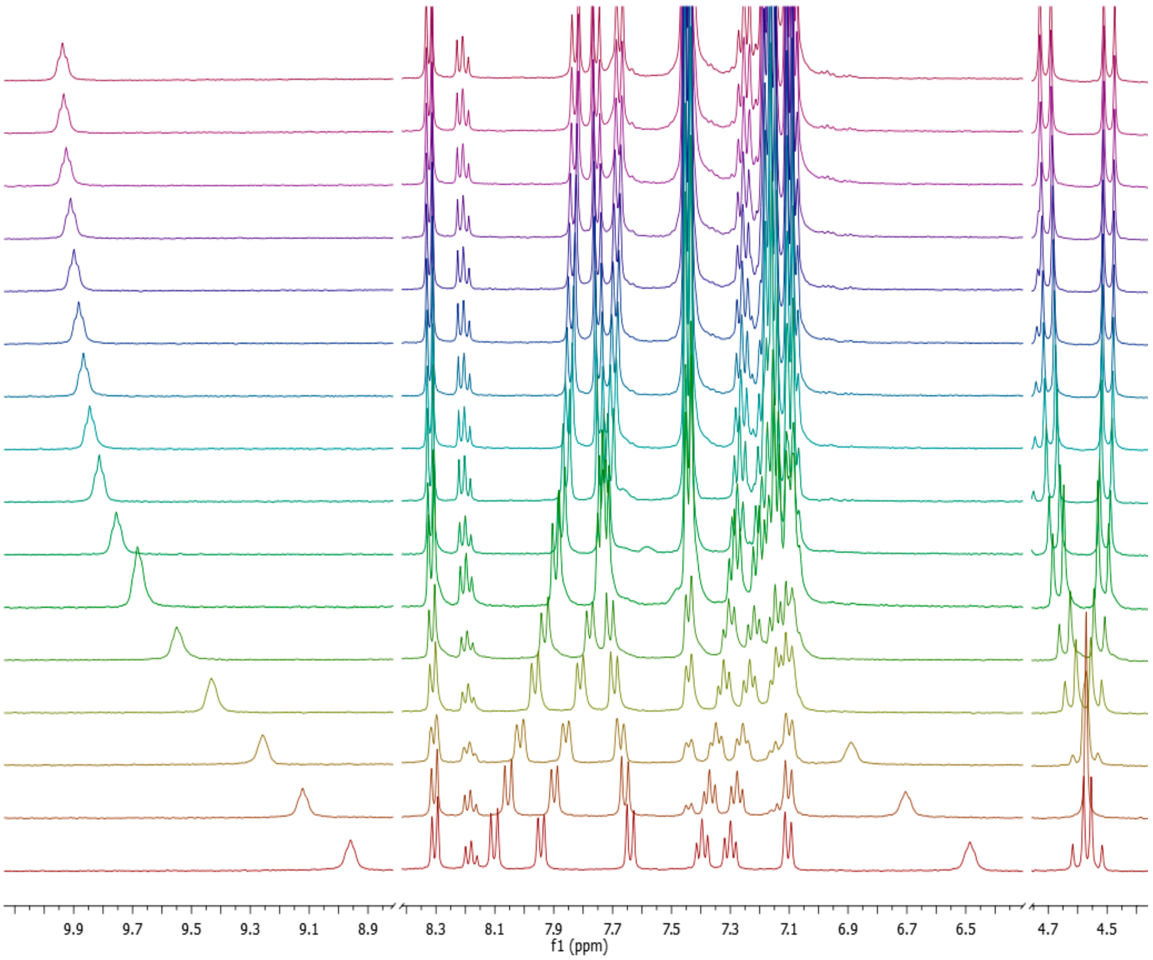

^{1}H NMR spectra changes upon titration of the receptor (*R*)-**3** with anion

Fig. S58 The details of ^{1}H NMR titration experiments of receptor **(*R*)-3** with **D-12** in acetone-*d$_6$*+0,5% H_2O

**Table S31.** Experimental data used to determine binding constant of receptor **(*R*)-2** with **D-12** in acetone-d_6+0,5% water									
Point	Eq of guest	C_{host} [M]	C_{guest} [M]	Range of chemical shift Δδ [ppm]					
				H1	H2	H3	H4	H5	H6
1	0,00	0,0044	0,0000	0,000	0,000	0,000	0,000	0,000	0,000
2	0,26		0,0012	0,162	-0,047	0,057	0,019	-0,027	0,037
3	0,52		0,0023	0,295	-0,088	0,103	0,035	-0,044	0,059
4	1,01		0,0044	0,473	-0,137	0,168	0,057	-0,059	0,091
5	1,48		0,0065	0,591	-0,171	0,210	0,071	-0,071	0,110
6	2,36		0,0104	0,725	-0,209	0,258	0,087	-0,084	0,132
7	3,17		0,0139	0,795	-0,228	0,284	0,095	-0,091	0,144
8	4,26		0,0186	0,854	-0,244	0,305	0,102	-0,096	0,154
9	5,22		0,0229	0,885	-0,253	0,316	0,107	-0,099	0,160
10	6,08		0,0266	0,907	-0,258	0,325	0,109	-0,100	0,163
11	6,85		0,0300	0,923	-0,262	0,330	0,111	-0,101	0,166
12	7,98		0,0349	0,940	-0,266	0,337	0,113	-0,103	0,169
13	8,94		0,0392	0,951	-0,269	0,341	0,115	-0,103	0,171
14	10,50		0,0460	0,965	-0,273	0,346	0,116	-0,104	0,174
15	11,72		0,0513	0,974	-0,274	0,350	0,118	-0,105	0,175
16	12,32		0,0539	0,979	-0,275	0,352	0,118	-0,105	0,176

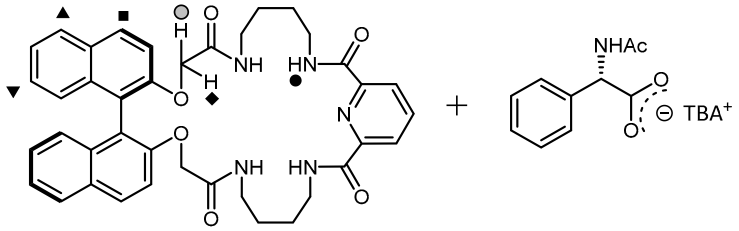

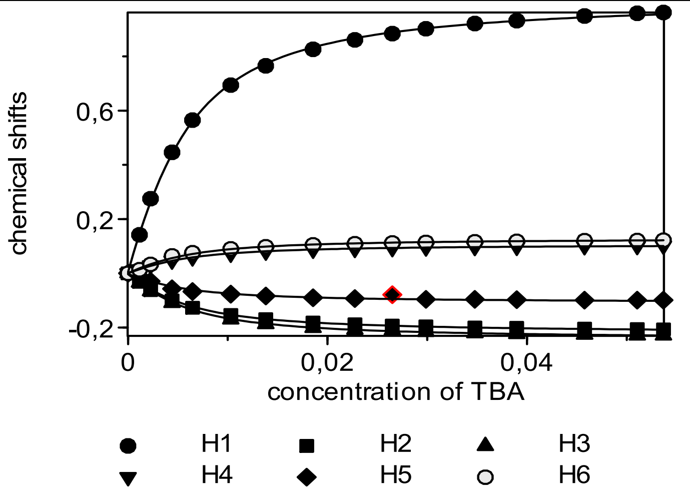

Changes in chemical shifts of the receptor (R)-**3** protons upon addition of anion

K=297 M^{-1}

C_H=4,38·10^{-3} M

C_G=0,093 M

Range of chemical shift

δ [ppm]

H1 0,962
H2 -0,207
H3 -0,229
H4 0,102
H5 -0,099
H6 0,122

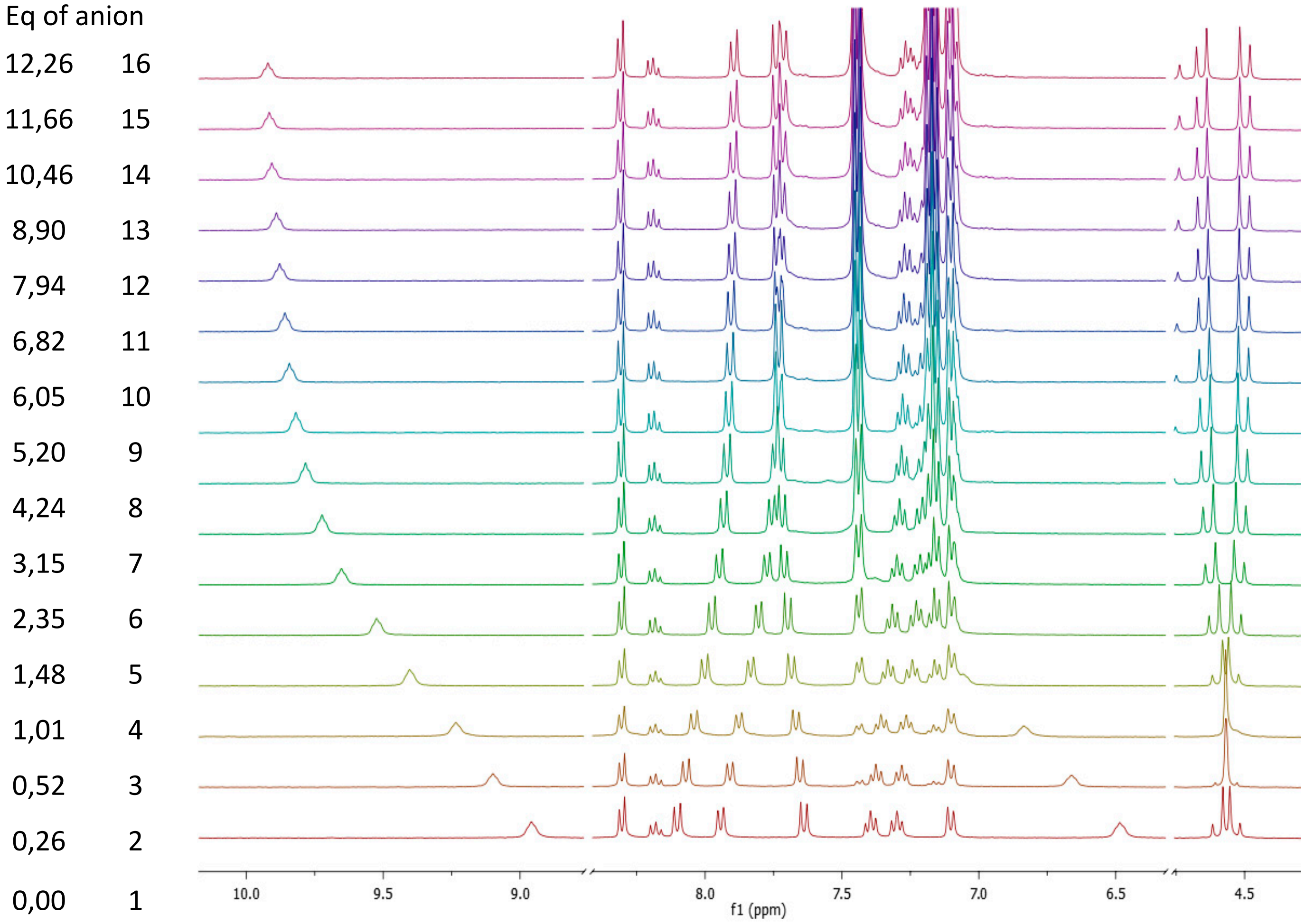

^{1}H NMR spectra changes upon titration of the receptor (R)-**3** with anion

Fig. S59 The details of ^{1}H NMR titration experiments of receptor **(R)-3** with **L-12** in acetone-d_6+0,5% H_2O

**Table S32.** Experimental data used to determine binding constant of receptor ***(R)*-2** with **L-12** in acetone-d_6+0,5% water									
Point	Eq of guest	C_{host} [M]	C_{guest} [M]	Range of chemical shift Δδ [ppm]					
				H1	H2	H3	H4	H5	H6
1	0,00	0,0044	0,0000	0,000	0,000	0,000	0,000	0,000	0,000
2	0,26		0,0012	0,142	-0,031	-0,034	0,014	-0,012	0,013
3	0,52		0,0023	0,275	-0,062	-0,066	0,029	-0,029	0,033
4	1,01		0,0044	0,446	-0,100	-0,109	0,047	-0,057	0,064
5	1,48		0,0065	0,565	-0,127	x	0,059	-0,067	0,076
6	2,35		0,0104	0,694	-0,154	-0,170	0,072	-0,077	0,090
7	3,15		0,0139	0,765	-0,169	-0,187	0,080	-0,084	0,098
8	4,24		0,0186	0,826	-0,182	-0,201	0,087	-0,090	0,105
9	5,20		0,0229	0,861	-0,188	-0,208	0,090	-0,093	0,109
10	6,05		0,0266	0,884	-0,193	-0,210	0,093	-0,079	0,112
11	6,82		0,0300	0,902	-0,196	-0,216	0,095	-0,096	0,114
12	7,94		0,0349	0,921	-0,200	-0,221	0,097	-0,096	0,116
13	8,90		0,0392	0,932	-0,201	-0,224	0,098	-0,097	0,118
14	10,46		0,0460	0,949	-0,204	-0,226	0,100	-0,099	0,120
15	11,66		0,0513	0,959	-0,206	-0,229	0,101	-0,099	0,121
16	12,26		0,0539	0,962	-0,207	-0,229	0,102	-0,099	0,122

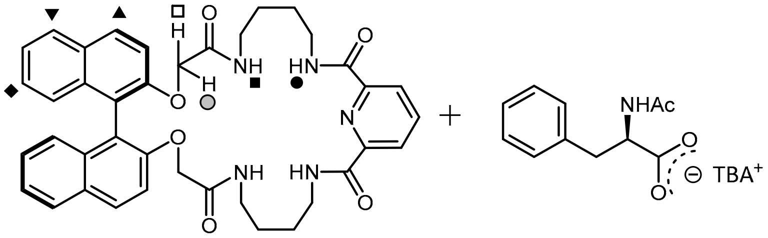

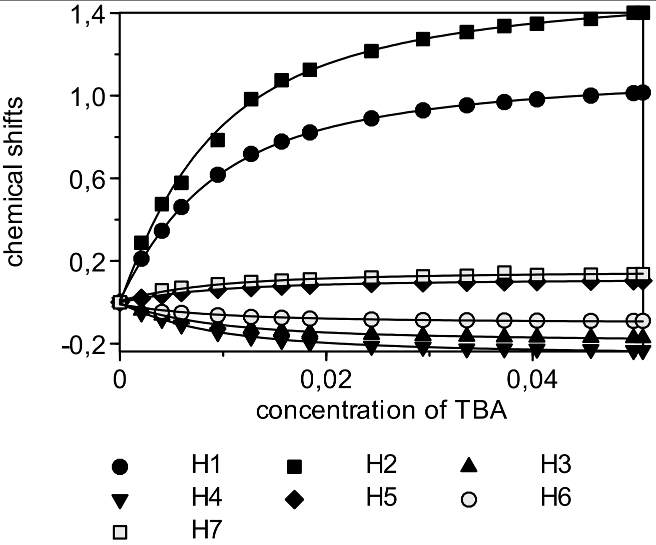

Changes in chemical shifts of the receptor (*R*)-**3** protons upon addition of anion

K=221 M^{-1}

C_H=5,37·10^{-3} M

C_G=0,091 M

Range of chemical shift δ [ppm]

H1 1,091
H2 1,463
H3 -0,205
H4 -0,252
H5 0,140
H6 0,116
H7 -0,034

Eq of anion	
9,05	15
8,56	14
8,01	13
7,39	12
6,67	11
5,83	10
4,83	9
3,65	8
3,11	7
2,52	6
1,88	5
1,18	4
0,81	3
0,41	2
0,00	1

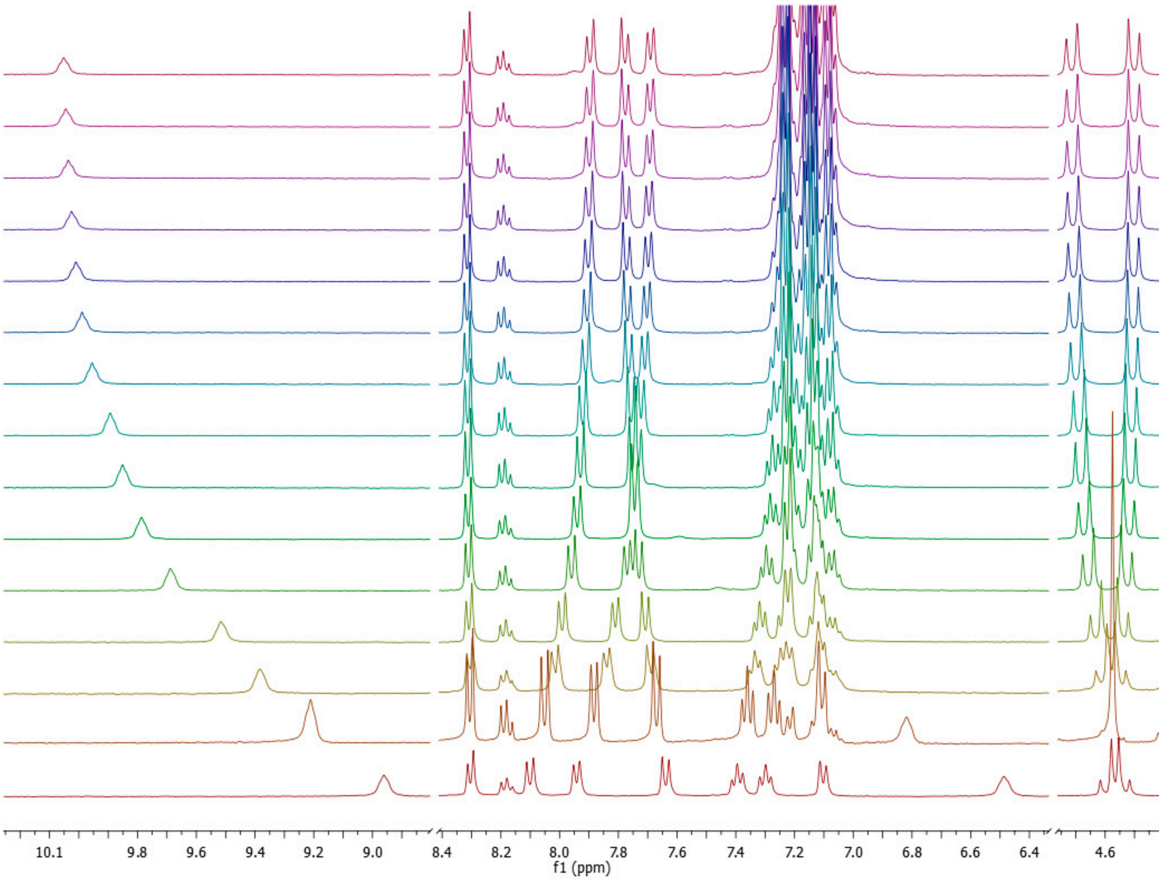

^{1}H NMR spectra changes upon titration of the receptor (*R*)-**3** with anion

Fig. S60 The details of ^{1}H NMR titration experiments of receptor **(*R*)-3** with **D-14** in acetone-d_6+0,5% H_2O

**Table S33.** Experimental data used to determine binding constant of receptor **(*R*)-3** with **D-14** in acetone-d_6+0,5% water										
Point	Eq of guest	C_{host} [M]	C_{guest} [M]	Range of chemical shift Δδ [ppm]						
				H1	H2	H3	H4	H5	H6	H7
1	0,00	0,0054	0,0000	0,000	0,000	0,000	0,000	0,000	0,000	0,000
2	0,41		0,0022	0,250	0,333	-0,049	-0,059	0,031	x	x
3	0,81		0,0043	0,420	0,587	-0,083	-0,101	0,052	0,015	0,013
4	1,18		0,0063	0,558	0,716	-0,108	-0,131	0,069	0,034	0,004
5	1,88		0,0101	0,728	0,974	-0,141	-0,171	0,092	0,060	-0,009
6	2,52		0,0135	0,825	1,110	-0,159	-0,198	0,105	0,075	-0,016
7	3,11		0,0167	0,891	1,198	-0,172	-0,210	0,113	0,085	-0,021
8	3,65		0,0196	0,932	1,257	-0,179	-0,219	0,119	0,092	-0,024
9	4,83		0,0260	0,994	1,337	-0,189	-0,232	0,126	0,102	-0,028
10	5,83		0,0313	1,028	1,381	-0,195	-0,240	0,131	0,107	-0,030
11	6,67		0,0358	1,048	1,414	-0,198	-0,243	0,134	0,110	-0,032
12	7,39		0,0397	1,065	1,429	-0,200	-0,246	0,136	0,112	-0,032
13	8,01		0,0431	1,076	1,449	-0,202	-0,249	0,138	0,114	-0,033
14	8,56		0,0460	1,083	1,463	-0,203	-0,251	0,139	0,115	-0,033
15	9,05		0,0486	1,091	1,463	-0,205	-0,252	0,140	0,116	-0,034

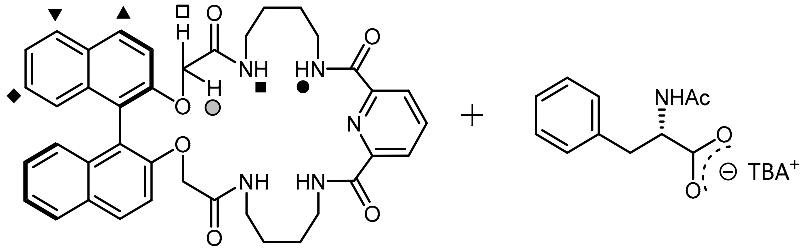

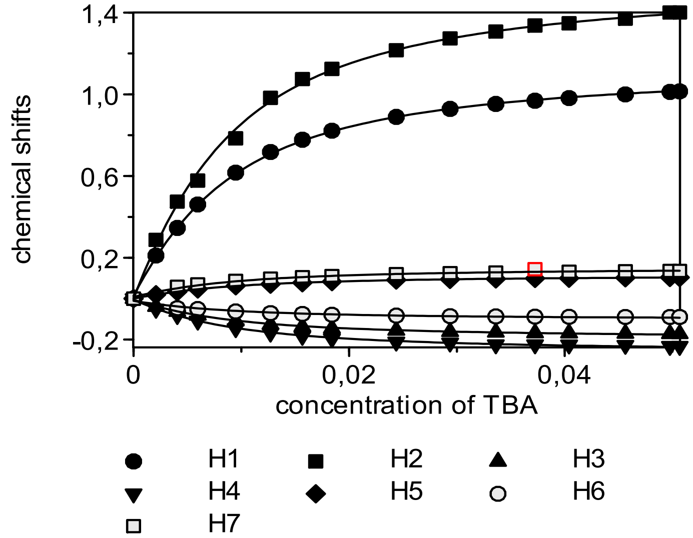

**K=167 M^{-1}**
C_H=5,37·10^{-3} M
C_G=0,085 M
Range of chemical shift δ[ppm]<br>H1 1,015<br>H2 1,401<br>H3 -0,172<br>H4 -0,233<br>H5 0,104<br>H6 -0,090<br>H7 0,136

Changes in chemical shifts of the receptor (*R*)-**3** protons upon addition of anion

Eq of anion	
9,43	16
9,26	15
8,49	14
7,52	13
6,93	12
6,25	11
5,47	10
4,54	9
3,42	8
2,92	7
2,36	6
1,76	5
1,11	4
0,76	3
0,39	2
0,00	1

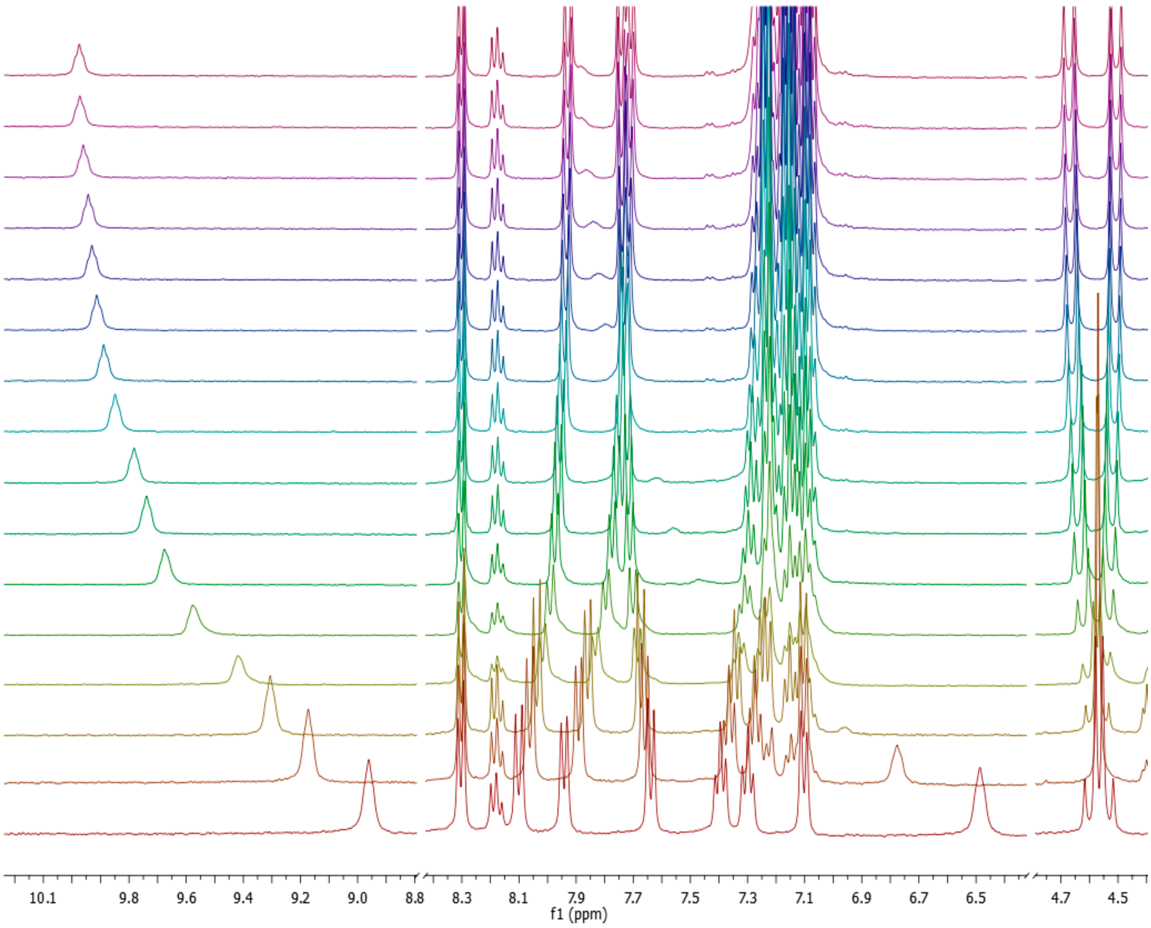

^{1}H NMR spectra changes upon titration of the receptor (*R*)-**3** with anion

Fig. S61 The details of ^{1}H NMR titration experiments of receptor ***(R)*-3** with **L-14** in acetone-d_6+0,5% H_2O

**Table S34.** Experimental data used to determine binding constant of receptor **(*R*)-3** with **L-14** in acetone-d$_6$+0,5% water										
Point	Eq of guest	C_{host} [M]	C_{guest} [M]	Range of chemical shift Δδ [ppm]						
				H1	H2	H3	H4	H5	H6	H7
1	0,00	0,0054	0,0000	0,000	0,000	0,000	0,000	0,000	0,000	0,000
2	0,39		0,0021	0,211	0,288	-0,039	-0,051	0,019	x	x
3	0,76		0,0041	0,346	0,475	-0,063	-0,082	0,034	-0,047	0,058
4	1,11		0,0060	0,461	0,578	-0,082	-0,109	0,047	-0,051	0,070
5	1,76		0,0095	0,617	0,785	-0,110	-0,146	0,063	-0,063	0,087
6	2,36		0,0127	0,718	0,983	-0,127	-0,168	0,071	-0,071	0,098
7	2,92		0,0157	0,778	1,075	-0,138	-0,184	0,078	-0,075	0,105
8	3,42		0,0184	0,822	1,125	-0,146	-0,196	0,083	-0,079	0,111
9	4,54		0,0244	0,890	1,216	-0,156	-0,212	0,091	-0,083	0,120
10	5,47		0,0294	0,929	1,274	-0,162	-0,217	0,094	-0,086	0,125
11	6,25		0,0336	0,953	1,308	-0,165	-0,225	0,097	-0,087	0,128
12	6,93		0,0373	0,969	1,337	-0,168	-0,225	0,099	-0,088	0,144
13	7,52		0,0404	0,982	1,348	-0,169	-0,226	0,100	-0,089	0,132
14	8,49		0,0456	1,000	1,371	-0,171	-0,228	0,102	-0,090	0,134
15	9,26		0,0498	1,012	1,401	-0,172	-0,233	0,104	-0,090	0,136
16	9,43		0,0507	1,015	1,401	-0,172	-0,233	0,104	-0,090	0,136

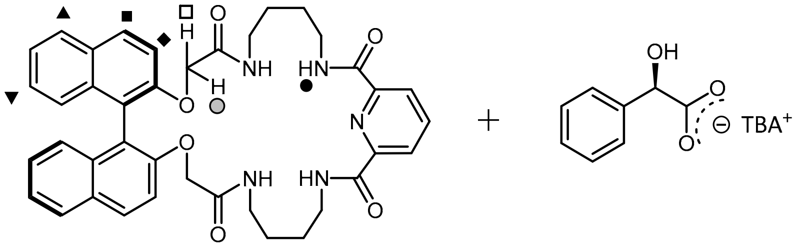

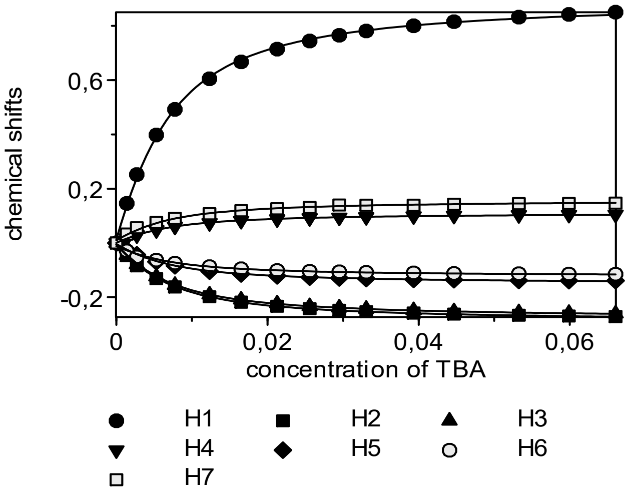

Changes in chemical shifts of the receptor (*R*)-**3** protons upon addition of anion

K=227 M^{-1}

C_H=4,66·10^{-3} M

C_G=0,111M

Range of chemical shift δ [ppm]

H1	0,850
H2	-0,271
H3	-0,260
H4	0,105
H5	-0,139
H6	-0,115
H7	0,147

Eq of anion

14,17	16
12,85	15
11,43	14
9,58	13
8,44	12
7,09	11
6,33	10
5,49	9
4,56	8
3,54	7
2,64	6
1,66	5
1,13	4
0,58	3
0,29	2
0,00	1

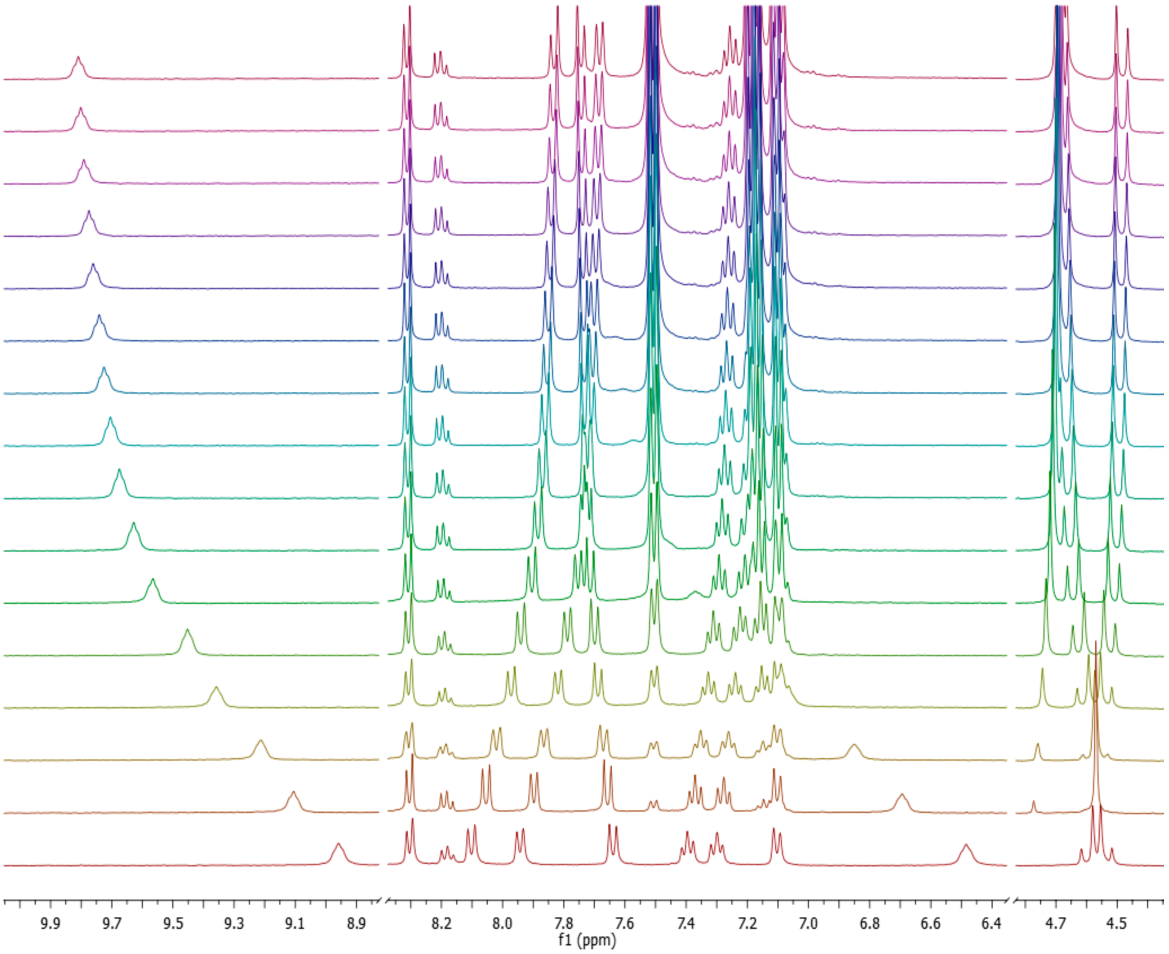

^{1}H NMR spectra changes upon titration of the receptor (*R*)-**3** with anion

Fig. S62 The details of ^{1}H NMR titration experiments of receptor **(*R*)-3** with **(*R*)-14** in acetone-d_6+0,5% H_2O

**Table S35.** Experimental data used to determine binding constant of receptor **(*R*)-3** with **(*R*)-14** in acetone-d_6+0,5% water										
Point	Eq of guest	C_{host} [M]	C_{guest} [M]	Range of chemical shift Δδ [ppm]						
				H1	H2	H3	H4	H5	H6	H7
1	0,00	0,0047	0,0000	0,000	0,000	0,000	0,000	0,000	0,000	0,000
2	0,29		0,0014	0,146	-0,047	-0,045	0,018	-0,025	-0,029	0,033
3	0,58		0,0027	0,252	-0,084	-0,079	0,029	-0,043	-0,051	0,056
4	1,13		0,0053	0,398	-0,131	-0,124	0,048	-0,069	-0,063	0,076
5	1,66		0,0077	0,492	-0,162	-0,155	0,061	-0,084	-0,074	0,091
6	2,64		0,0123	0,605	-0,198	-0,190	0,074	-0,104	-0,087	0,108
7	3,54		0,0165	0,667	-0,218	-0,209	0,082	-0,114	-0,095	0,119
8	4,56		0,0213	0,714	-0,233	-0,223	0,088	-0,121	-0,101	0,126
9	5,49		0,0256	0,744	-0,242	-0,233	0,092	-0,126	-0,104	0,131
10	6,33		0,0295	0,765	-0,248	-0,238	0,094	-0,129	-0,106	0,140
11	7,09		0,0331	0,781	-0,253	-0,243	0,096	-0,131	-0,108	0,139
12	8,44		0,0393	0,800	-0,258	-0,248	0,099	-0,134	-0,110	0,139
13	9,58		0,0447	0,815	-0,262	-0,252	0,101	-0,135	-0,112	0,143
14	11,43		0,0533	0,832	-0,266	-0,256	0,103	-0,138	-0,113	0,145
15	12,85		0,0599	0,842	-0,269	-0,258	0,104	-0,139	-0,114	0,146
16	14,17		0,0661	0,850	-0,271	-0,260	0,105	-0,139	-0,115	0,147

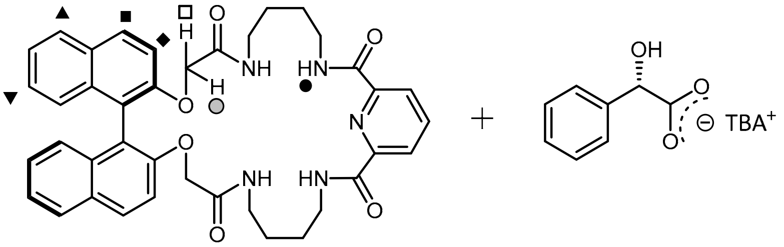

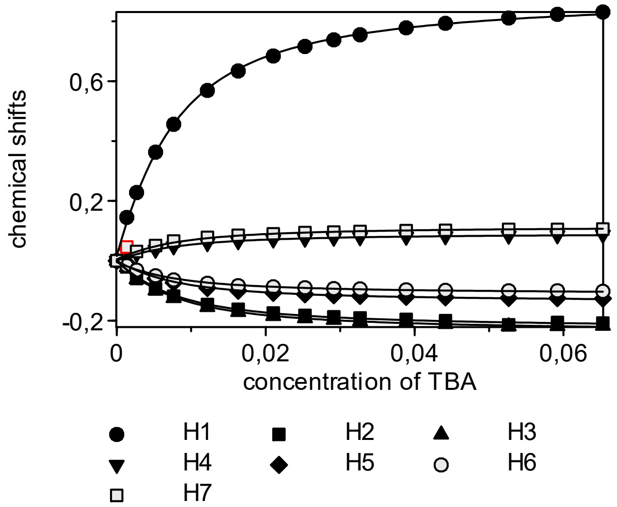

Changes in chemical shifts of the receptor (*R*)-**3** protons upon addition of anion

K=191 M^{-1}

C_H=4,66·10^{-3} M

C_G=0,110 M

Range of chemical shift δ [ppm]

H1 0,831
H2 -0,208
H3 -0,217
H4 0,087
H5 -0,126
H6 -0,102
H7 0,106

Eq of anion	
14,00	16
12,69	15
11,29	14
9,47	13
8,34	12
7,01	11
6,25	10
5,42	9
4,51	8
3,50	7
2,61	6
1,64	5
1,12	4
0,57	3
0,29	2
0,00	1

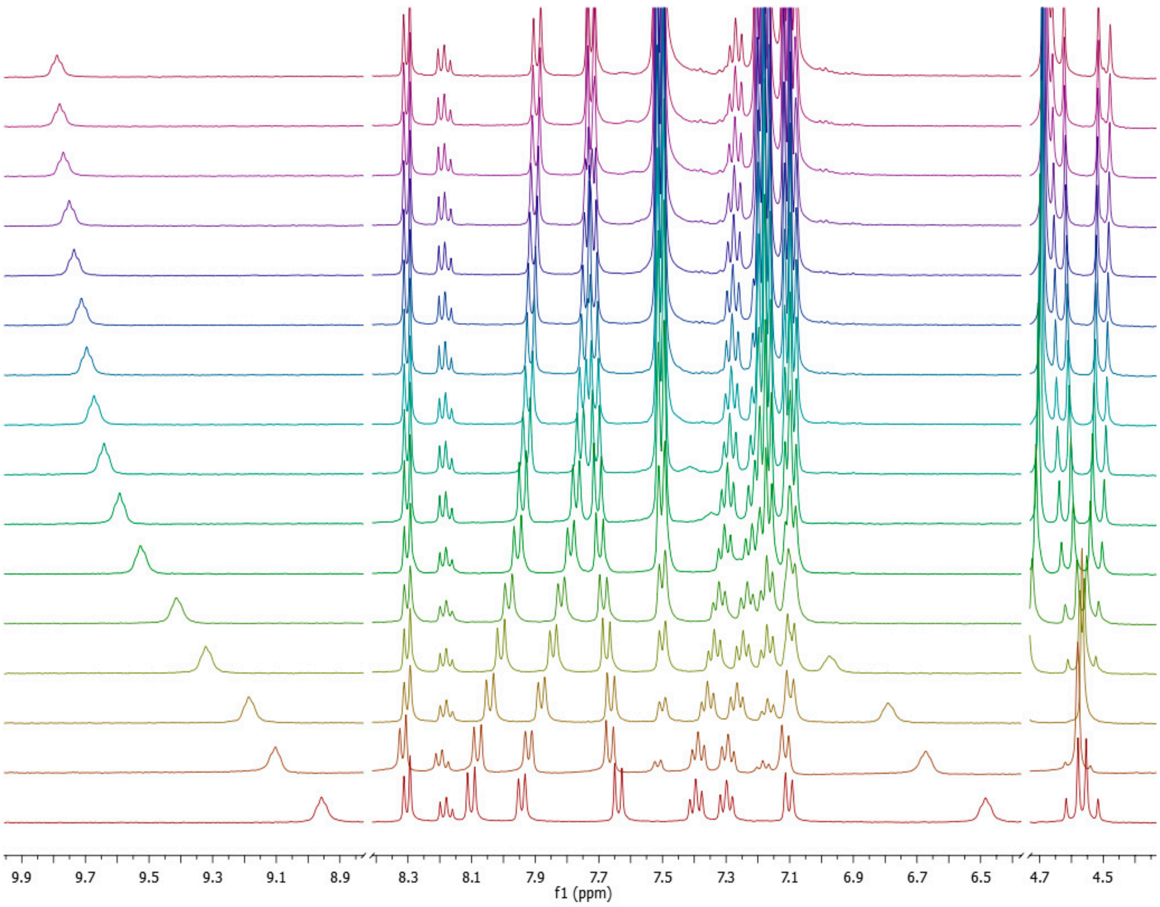

^{1}H NMR spectra changes upon titration of the receptor (*R*)-**3** with anion

Fig. S63 The details of ^{1}H NMR titration experiments of receptor **(*R*)-3** with **(*S*)-14** in acetone-d_6+0,5% H_2O

**Table S36.** Experimental data used to determine binding constant of receptor **(*R*)-3** with **(*S*)-14** in acetone-d_6+0,5% water										
Point	Eq of guest	C_{host} [M]	C_{guest} [M]	Range of chemical shift Δδ [ppm]						
				H1	H2	H3	H4	H5	H6	H7
1	0,00	0,0047	0,0000	0,000	0,000	0,000	0,000	0,000	0,000	0,000
2	0,29		0,0014	0,145	-0,020	-0,021	0,026	-0,008	-0,016	0,046
3	0,57		0,0027	0,228	-0,059	-0,062	0,023	-0,037	-0,031	0,031
4	1,12		0,0052	0,363	-0,094	-0,099	0,037	-0,059	-0,050	0,051
5	1,64		0,0076	0,456	-0,118	-0,124	0,048	-0,073	-0,064	0,066
6	2,61		0,0122	0,569	-0,146	-0,154	0,059	-0,091	-0,075	0,078
7	3,50		0,0163	0,634	-0,162	-0,171	0,065	-0,100	-0,083	0,085
8	4,51		0,0210	0,684	-0,174	-0,184	0,071	-0,107	-0,087	0,090
9	5,42		0,0253	0,716	-0,182	-0,192	0,074	-0,112	-0,090	0,094
10	6,25		0,0291	0,738	-0,187	-0,197	0,077	-0,115	-0,093	0,096
11	7,01		0,0327	0,755	-0,191	-0,202	0,079	-0,117	-0,094	0,098
12	8,34		0,0389	0,778	-0,196	-0,207	0,080	-0,120	-0,097	0,101
13	9,47		0,0441	0,793	-0,200	-0,211	0,082	-0,122	-0,099	0,102
14	11,29		0,0526	0,811	-0,214	-0,219	0,084	-0,124	-0,100	0,105
15	12,69		0,0592	0,823	-0,206	-0,218	0,085	-0,125	-0,100	0,105
16	14,00		0,0653	0,831	-0,208	-0,217	0,087	-0,126	-0,102	0,106

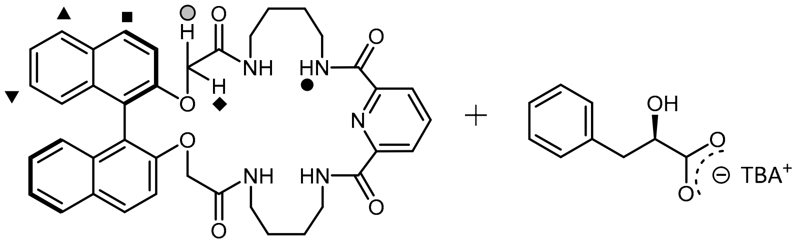

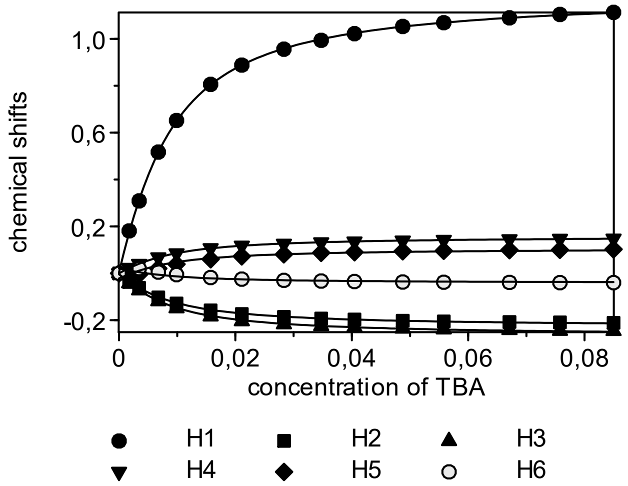

**K=169 M^{-1}**
C_H=5,29·10^{-3} M
C_G=0,142 M
Range of chemical shift δ [ppm]<br>H1 1,113<br>H2 -0,211<br>H3 -0,246<br>H4 0,148<br>H5 0,102<br>H6 -0,040

Changes in chemical shifts of the receptor (*R*)-**3** protons upon addition of anion

Eq of anion

16,07	16
14,32	15
12,68	14
10,55	13
9,22	12
7,65	11
6,57	10
5,36	9
3,99	8
2,98	7
1,87	6
1,28	5
0,65	4
0,33	3
0,00	2
16,07	1

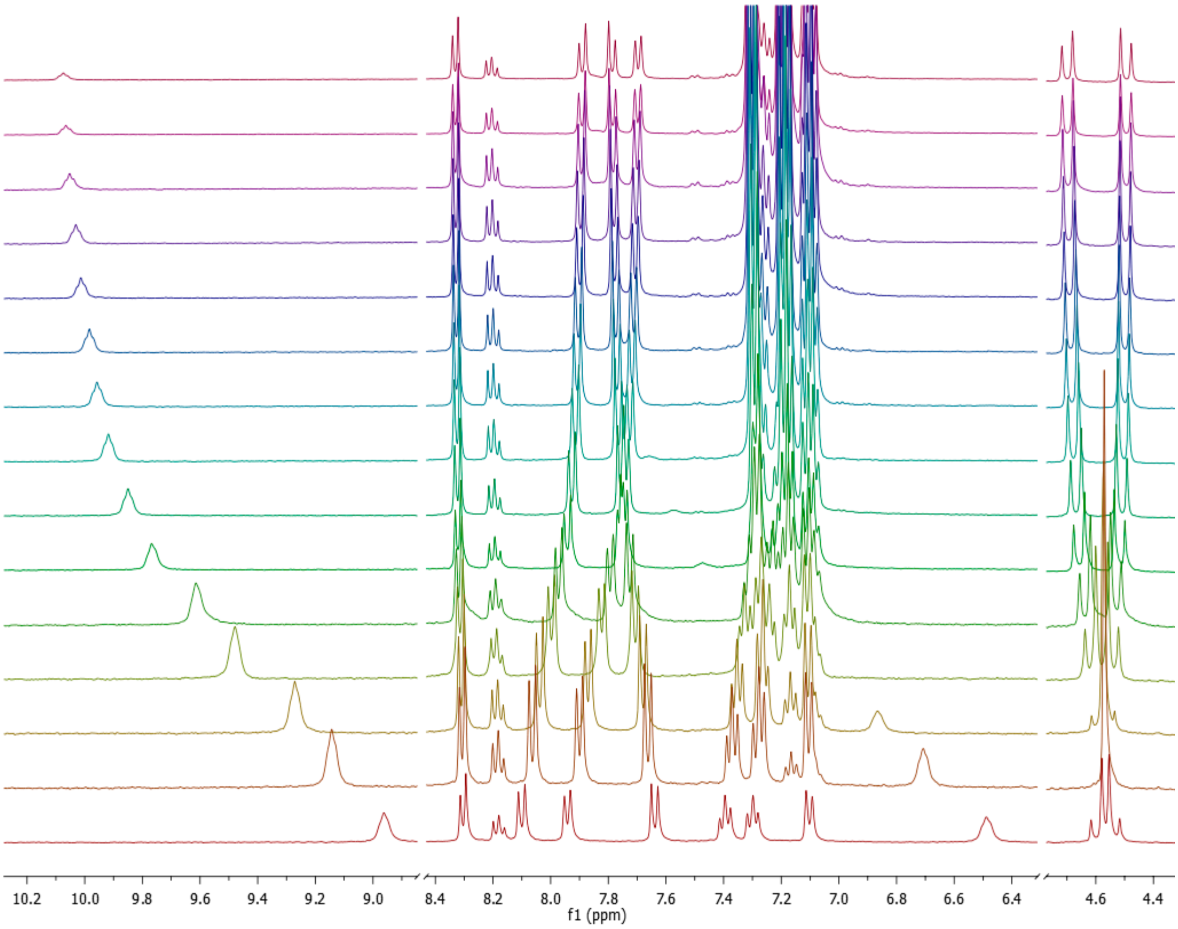

^{1}H NMR spectra changes upon titration of the receptor (*R*)-**3** with anion

Fig. S64 The details of ^{1}H NMR titration experiments of receptor **(*R*)-3** with **D-13** in acetone-d_6+0,5% H_2O

Table S37. Experimental data used to determine binding constant of receptor **(*R*)-2** with **D-13** in acetone-d_6+0,5% water

Point	Eq of guest	C_{host} [M]	C_{guest} [M]	Range of chemical shift Δδ [ppm]					
				H1	H2	H3	H4	H5	H6
1	0,00	0,0053	0,0000	0,000	0,000	0,000	0,000	0,000	0,000
2	0,33		0,0017	0,181	-0,037	-0,042	0,023	x	x
3	0,65		0,0035	0,309	-0,062	-0,072	0,040	-0,004	0,020
4	1,28		0,0067	0,517	-0,103	-0,119	0,068	0,021	0,005
5	1,87		0,0099	0,652	-0,129	-0,149	0,085	0,039	-0,006
6	2,98		0,0157	0,806	-0,158	-0,184	0,107	0,060	-0,018
7	3,99		0,0211	0,888	-0,174	-0,204	0,118	0,072	-0,025
8	5,36		0,0283	0,956	-0,186	-0,217	0,126	0,081	-0,030
9	6,57		0,0347	0,994	-0,193	-0,225	0,132	0,087	-0,033
10	7,65		0,0405	1,022	-0,197	-0,230	0,135	0,090	-0,035
11	9,22		0,0488	1,053	-0,202	-0,236	0,139	0,094	-0,037
12	10,55		0,0558	1,069	-0,205	-0,239	0,142	0,096	-0,038
13	12,68		0,0671	1,090	-0,208	-0,242	0,145	0,099	-0,039
14	14,32		0,0758	1,104	-0,209	-0,245	0,147	0,100	-0,040
15	16,07		0,0850	1,113	-0,211	-0,246	0,148	0,102	-0,040
16	0,00		0,0000	0,000	0,000	0,000	0,000	0,000	0,000

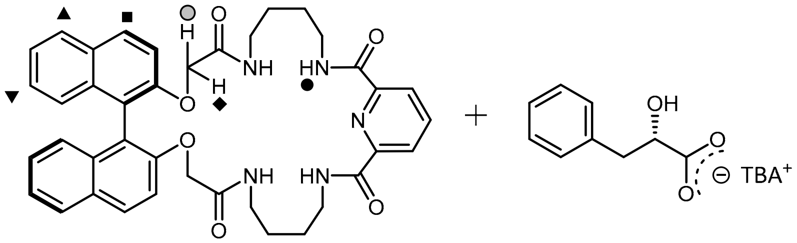

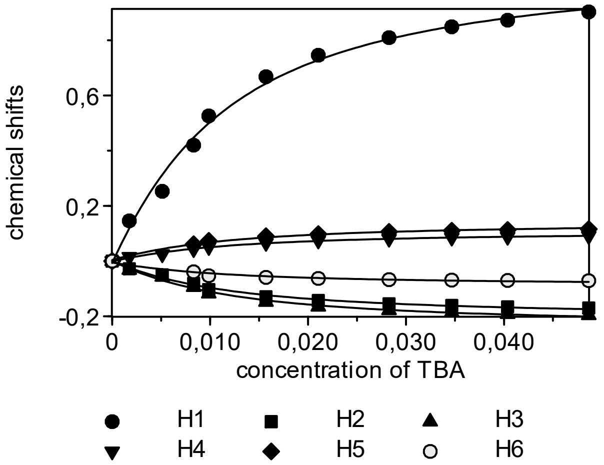

Changes in chemical shifts of the receptor (*R*)-**3** protons upon addition of anion

**K=114 M^{-1}**
C_H=5,29·10^{-3} M
C_G=0,142 M
Range of chemical shift<br>δ [ppm]<br>H1 0,967<br>H2 -0,178<br>H3 -0,205<br>H4 0,097<br>H5 0,089<br>H6 -0,072

Eq of anion	
15,59	15
14,29	14
12,66	13
10,53	12
9,20	11
7,63	10
6,55	9
5,34	8
3,98	7
2,97	6
1,86	5
1,57	4
0,97	3
0,33	2
0,00	1

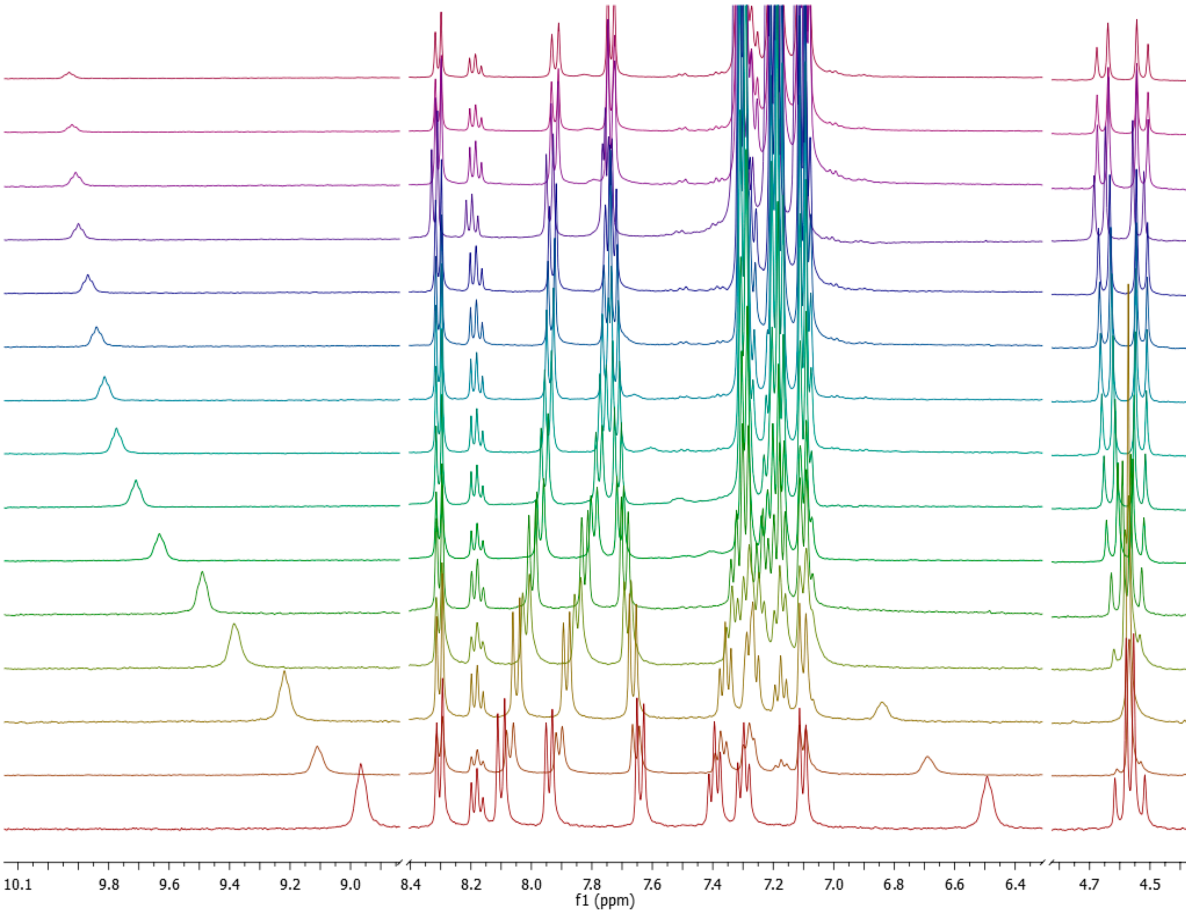

^{1}H NMR spectra changes upon titration of the receptor (*R*)-**3** with anion

Fig. S65 The details of ^{1}H NMR titration experiments of receptor ***(R)*-3** with **L-13** in acetone-d_6+0,5% H_2O

**Table S38.** Experimental data used to determine binding constant of receptor **(*R*)-2** with **L-13** in acetone-d_6+0,5% water									
Point	Eq of guest	C_{host} [M]	C_{guest} [M]	Range of chemical shift Δδ [ppm]					
				H1	H2	H3	H4	H5	H6
1	0,00	0,0053	0,0000	0,000	0,000	0,000	0,000	0,000	0,000
2	0,33		0,0017	0,146	-0,028	-0,032	0,015	x	x
3	0,97		0,0051	0,253	-0,050	-0,057	0,025	x	x
4	1,57		0,0083	0,420	-0,081	-0,094	0,043	0,061	-0,039
5	1,86		0,0099	0,526	-0,103	-0,118	0,051	0,073	-0,053
6	2,97		0,0157	0,668	-0,129	-0,148	0,066	0,090	-0,059
7	3,98		0,0210	0,746	-0,143	-0,165	0,075	0,098	-0,063
8	5,34		0,0283	0,810	-0,155	-0,178	0,081	0,106	-0,067
9	6,55		0,0347	0,849	-0,161	-0,186	0,085	0,110	-0,069
10	7,63		0,0404	0,873	-0,166	-0,192	0,088	0,113	-0,070
11	9,20		0,0487	0,903	-0,170	-0,196	0,091	0,116	-0,071
12	10,53		0,0557	0,935	-0,160	-0,186	0,105	0,131	-0,059
13	12,66		0,0669	0,944	-0,176	-0,205	0,095	0,088	-0,072
14	14,29		0,0756	0,956	-0,177	-0,205	0,096	0,089	-0,072
15	15,59		0,0824	0,967	-0,178	-0,205	0,097	0,089	-0,072
